# scSuperAnnotator: a platform for benchmarking comparison and visualizing automated cellular annotation methods for scRNA-seq data

**DOI:** 10.1093/nar/gkaf1470

**Published:** 2026-01-06

**Authors:** Qi Qi, Yanchi Su, Yi Fan, Zhuohan Yu, Yujian Huang, Ka-Chun Wong, Xiangtao Li

**Affiliations:** School of Artificial Intelligence, Jilin University, 2699 Qianjin Street, Chaoyang District, Changchun, Jilin, 130012, China; School of Information Science and Technology, Northeast Normal University, 5268 Renmin Street, Nanguan District, Changchun, Jilin, 130024, China; School of Artificial Intelligence, Jilin University, 2699 Qianjin Street, Chaoyang District, Changchun, Jilin, 130012, China; School of Artificial Intelligence, Jilin University, 2699 Qianjin Street, Chaoyang District, Changchun, Jilin, 130012, China; College of Computer Science and Cyber Security, Chengdu University of Technology, No. 1, East Third Road, Erxianqiao, Chenghua District, Chengdu, Sichuan, 610059, China; Department of Computer Science, City University of Hong Kong, Tat Chee Avenue, Kowloon Tong, Hong Kong SAR, 000000, China; School of Artificial Intelligence, Jilin University, 2699 Qianjin Street, Chaoyang District, Changchun, Jilin, 130012, China

## Abstract

The advent of single-cell RNA-seq has revolutionized the study of gene expression profiles with unparalleled resolution. Accurate identification of cell types from single-cell RNA-seq data is crucial to advance our understanding of disease progression and tumor microenvironments. Although various methods have been proposed to facilitate cell-type annotation, complementing traditional manual approaches, a comprehensive platform that integrates these methods for automated identification is still lacking. To address this gap, we developed scSuperAnnotator, the first online platform that integrates a variety of cell-type identification methods, including both marker gene-based and reference-based approaches, for the automated identification of cell types from single-cell RNA-seq data. A key feature of scSuperAnnotator is its user-friendly interface, which allows researchers to perform one-stop annotation and analyses of single-cell RNA-seq without needing programming expertise. The platform enables users to select appropriate methods and conduct downstream analyses through intuitive, multi-perspective comparisons, streamlining the entire process for greater convenience and efficiency. Furthermore, our platform provides a comprehensive and systematic comparison of existing annotation methods, offering valuable information to researchers.

## Graphical Abstract

**Graphical Abstract:**
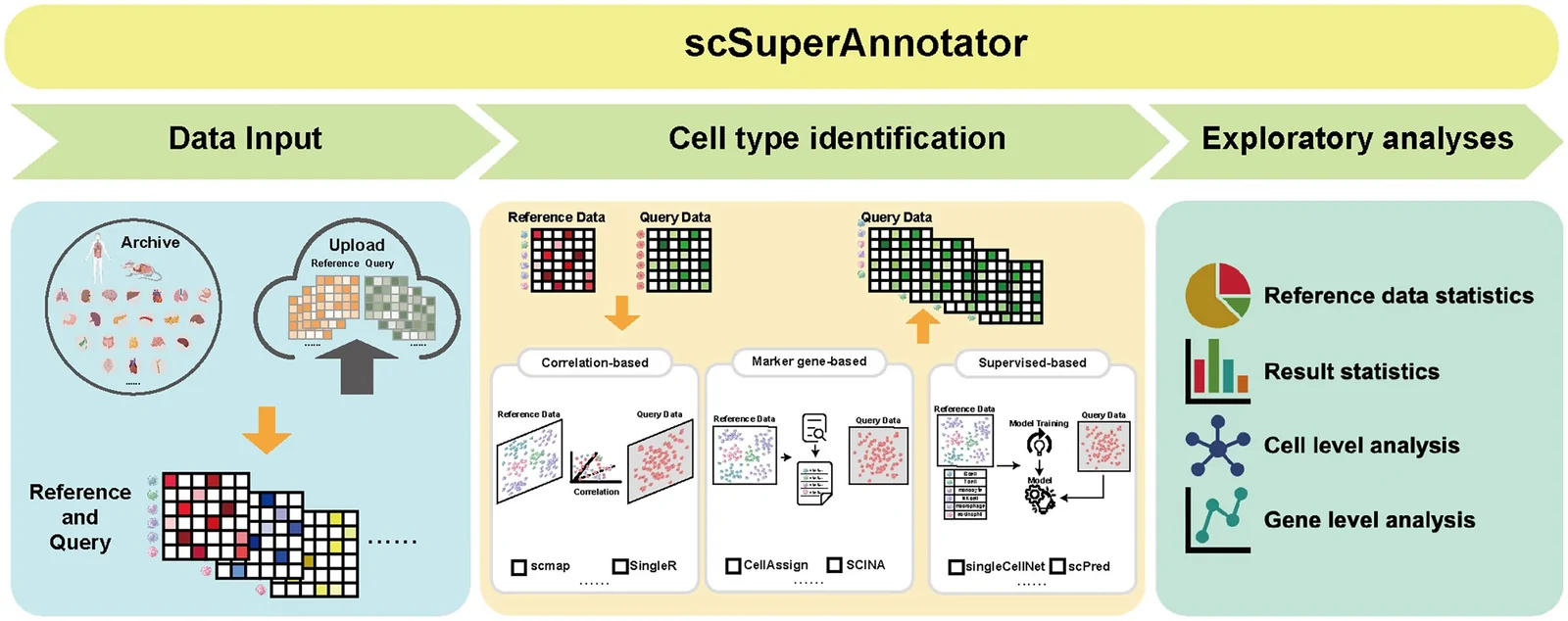

## Introduction

The advent of single-cell RNA sequencing (scRNA-seq) technologies has illuminated the heterogeneity and complexity of RNA transcripts at the cellular level, revealing gene expression patterns with greater resolution than traditional bulk RNA sequencing [1]. Identifying and characterizing cell types through unsupervised clustering and manual annotation, often relying on marker genes, is a common but challenging task due to potential biases from unknown cell type numbers and the labor-intensive, subjective nature of manual labeling, which can impact the reliability of results [2–4].

Cell type identification is a pivotal step in scRNA-seq data analysis, where computational tools significantly mitigate the time and subjectivity associated with manual annotation [5–7]. These tools are divided into reference-based methods and marker gene-based methods [8]. With the increasing availability of scRNA-seq datasets featuring reliable labels, the development of reference-based methods has accelerated. These methods are subdivided into correlation-based approaches, such as scmap [9], SingleR [10], and CHETAH [11], which align unlabeled datasets with annotated references. In contrast, supervised classification methods leverage machine learning techniques to propagate labels from labeled to unlabeled datasets, with notable implementations including singleCellNet [12] utilizing random forests, and others such as ACTINN [13], which employ artificial neural networks. Furthermore, other methods, such as scPred [14], use support vector machines, while Garnett [15] adopts hierarchical models predicated on user-defined cell type labels.

Building upon the advancements in machine learning and deep learning tools, the proliferation of transcriptomic datasets with reliable labels has further enhanced the refinement of cell-type-specific marker gene libraries. Libraries such as CellMarker [16] and PanglaoDB [17] provide essential reference markers crucial for the accurate identification of cell types, bridging the divide between empirical data and computational analysis. Marker gene-based methods take advantage of these resources, employing either scoring systems or statistical models to assign cell types. For instance, tools such as scCATCH [18] utilize reference marker genes along with scoring systems to classify cell types, while SCINA [19] and CellAssign [20] apply advanced statistical methods to assign probabilities to reference cell types, thus increasing both the precision and reliability of cell type identification. Although many cell-type identification methods perform well in various situations, their accessibility is limited for users without programming skills, and there is a lack of platforms for comprehensive integration and comparison of these state-of-the-art methods, necessitating more systematic and comprehensive experiments to address this issue.

Here, we present scSuperAnnotator, the first computational platform and a user-friendly web-based portal that facilitates scRNA-seq cell type identification and downstream comparative analyses without the need for programming knowledge. scSuperAnnotator integrates a range of cutting-edge marker gene-based and reference-based cell type identification methods, complemented by downstream analyses, including differentially expressed genes (DEGs), Gene Ontology (GO) enrichment, Kyoto Encyclopedia of Genes and Genomes (KEGG) enrichment, cell trajectory inference, and cell–cell communication. Additionally, scSuperAnnotator offers a suite of diverse visualization tools such as bar plots, uniform-manifold approximation and projection (UMAP) plots, and heatmaps. These tools assist users in selecting the optimal methods for their datasets and guide the necessary subsequent analyses based on the cell type identification results. scSuperAnnotator also provides valuable references and insights to help users identify the most suitable methods for their research needs. We hope that this platform will be particularly beneficial for researchers lacking a background in computer science or programming, as it simplifies complex data analysis processes. It is crucial to emphasize that our primary objective is to showcase the platform’s ability to visualize and quantify diverse analytical results, rather than endorse specific algorithms.

## Materials and methods

### The overall framework of scSuperAnnotator

**Figure 1.**
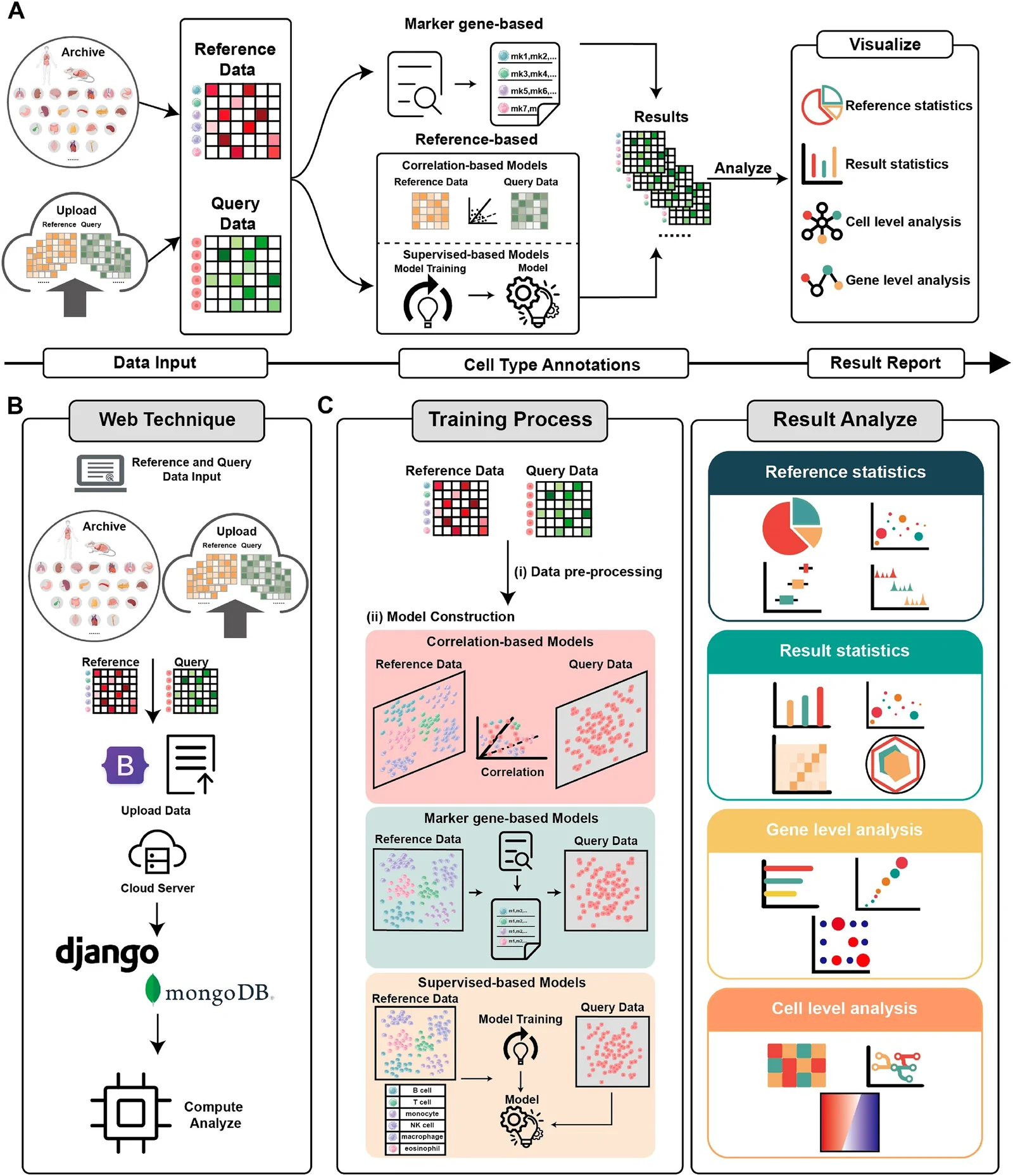
**(A**) The overall framework of the proposed scSuperAnnotator, which consists of three modules: (i) Data Input module; (ii) Cell Type Annotations module; and (iii) Result Report module. (**B**) The web technique of scSuperAnnotator. (**C**) The workflow of the training process of Cell Type Annotations module and Result Report module.

scSuperAnnotator is a user-friendly web portal designed to help users leverage mature scRNA-seq methods for cell type identification. In addition to enabling powerful downstream functional analyses of single-cell genomic and transcriptomic data, scSuperAnnotator also serves as an objective benchmarking framework for quantitatively and qualitatively evaluating cell annotation methods applied to scRNA-seq data. Using this framework, we have conducted a comprehensive evaluation of the performance of multiple annotation algorithms across multiple datasets and analysis scenarios. The general overview of scSuperAnnotator is shown in Fig. 1A. scSuperAnnotator consists of three main modules: (i) Data Input Module: This module organizes and processes reference datasets in a modular, hierarchical structure to enable efficient and accurate cell-type annotation, with particular emphasis on disease-related applications. The archive spans human and mouse tissues across healthy and diseased conditions, providing broad anatomical coverage. The module also supports user-uploaded datasets (Supplementary Fig. S2). (ii) Cell Type Annotations Module: scSuperAnnotator autonomously trains models for cell type identification by integrating reference-based methods, such as correlation-based and supervised classification models, with marker gene-based approaches. It processes input data to develop robust models tailored to its specific characteristics; (iii) Result Report Module: This module provides visualization and analysis output in multiple formats, enabling in-depth analysis at the cell and gene levels. It also benchmarks the performance of cell annotation methods through intuitive visualization methods such as bar plots and heatmaps.

### Data Input Module

scSuperAnnotator is a comprehensive solution for scRNA-seq data annotation, designed to enable accurate cell type identification and to facilitate downstream analyses through advanced annotation methodologies. To support both reference-based and marker gene-based annotation approaches, scSuperAnnotator provides pre-curated reference datasets and standardized marker gene resources, providing comprehensive and flexible annotation capabilities, allowing researchers to perform robust and context-specific cell type annotations across a wide range of biological conditions (Supplementary Fig. S80).

The reference datasets in scSuperAnnotator are organized using a modular and hierarchical structure, specifically designed to facilitate efficient and accurate cell type annotation, particularly in disease-related applications. These datasets are curated from nearly 30 public resources, including the Human Cell Landscape [21], The Tabula Sapiens [22], Tabula Muris Senis [23], Human Lung Cell Atlas [24], healthy human liver [25], the Mouse Cell Atlas (MCA) [26], CZ CELLxGENE Discover [27], and DISCO [28], as well as literature-curated datasets focused on key pathological cell types across various disease contexts. Collectively, these datasets encompass a wide range of physiological and pathological conditions and cover multiple organs such as the brain, liver, pancreas, and blood, thereby providing comprehensive and reliable data support for diverse annotation tasks. As illustrated in Supplementary Fig. S1A, the hierarchical structure of the Data Input Module allows users to systematically select reference datasets based on a three-tiered classification system: species (e.g. human or mouse), health status (healthy or diseased), and tissue of origin (e.g. pancreas, liver, or lung). This structure effectively distinguishes species-specific and disease-specific transcriptomic variations, minimizing annotation errors caused by heterogeneous reference datasets. Furthermore, the separation of healthy and diseased samples enhances the resolution and accuracy of annotations in disease-related studies, particularly in cases involving disease-induced changes in cell states. Lastly, the organ-level organization enables high-resolution, tissue-specific analyses, allowing users to precisely select datasets that align with their specific research objectives.

In addition to reference-based annotation methods, scSuperAnnotator also integrates marker gene-based approaches. Unlike reference-based methods, these approaches do not rely on annotated reference datasets but instead utilize predefined marker gene sets to automatically identify cell types. To support this functionality, scSuperAnnotator incorporates a comprehensive and well-curated marker gene resource. The current marker gene files are compiled based on Cell Marker Accordion [29] and integrate information from >20 published human and mouse marker gene databases, widely adopted standard collections of cell sorting markers, and literature-curated marker genes associated with key pathological cell types across various disease contexts. To ensure interoperability and consistency, all cell type names, tissue terms, and disease annotations within the marker gene set have been systematically standardized. As a result, these marker gene-based methods offer broad applicability across both healthy and diseased conditions, providing a robust and biologically meaningful foundation for reliable cell type identification, including the detection of disease-associated cell populations.

Given the inherent limitations of public reference datasets—such as the absence of certain cell populations and technical variations across sequencing platforms—scSuperAnnotator also supports the analysis of user-provided datasets. Researchers can upload their own data to the platform, enabling greater flexibility to address specific research needs. To ensure data security and user privacy, scSuperAnnotator has implemented strict data protection measures. All uploaded datasets are temporarily stored on secure servers, are inaccessible to other users, and are permanently deleted upon completion of the analysis. These safeguards ensure the confidentiality and integrity of user-submitted data throughout the entire analytical process.

By integrating these three categories of data resources—hierarchically organized reference datasets, standardized marker gene sets, and flexible user-uploaded datasets—scSuperAnnotator offers a highly adaptable and comprehensive framework for accurate and biologically interpretable cell type annotation across a wide range of research applications (Supplementary Fig. S1B).

### Cell Type Annotations Module

The process of identifying and analyzing cell types via scSuperAnnotator generally involves three systematic steps: (i) data selection or upload; (ii) task selection; and (iii) task implementation and visualization, which are described in detail below.

#### 1st step: Data selection or upload

Users primarily initiate cell type annotation tasks in the Identification tab. To support efficient organization and retrieval, scSuperAnnotator’s dataset archive is a tree-structured hierarchy ordered by species, then health status (health or disease), and finally tissue type. This design mirrors common biomedical data-portal practice—progressing from broad species-level separation to specific anatomical context—and provides biologically meaningful navigation of large single-cell collections. Users can rapidly drill down (e.g. Human → Healthy versus Disease → Liver) to locate relevant datasets. The data selection workflow is shown in Supplementary Fig. S1. Recognizing the inherent limitations of public references—such as missing cell populations and platform-specific technical differences—scSuperAnnotator also supports analysis of user-supplied datasets, allowing researchers to upload their own data to meet study-specific needs. Requirements for data upload are detailed in Supplementary Note S1.

#### 2nd step: Task selection

After selecting or uploading the desired datasets, users can customize their task by selecting specific cell type identification methods and exploratory analysis techniques. To ensure data privacy, users can choose to mark their submitted tasks as “Private,” which conceals them from the public task list. Upon task submission, a unique Task ID is generated, which users should save to retrieve and view the task later via the search box on the home page. Users may also choose to provide an email address to receive updates on task status from scSuperAnnotator, although this is optional.

#### 3rd step: Task implementation and visualization

Once the “Submit” button is clicked, the back-end program begins processing the input data using the chosen methods. Initially, data undergo preprocessing based on the protocols associated with the selected cell type annotation method. The preprocessed data are then input into the selected model(s) for cell type identification. scSuperAnnotator supports a wide range of state-of-the-art cell type identification models, including marker gene-based methods such as CellAssign, correlation-based methods such as SingleR, and supervised classification-based methods such as singleCellNet (Supplementary Table S1). The platform allows for the simultaneous selection of multiple models and provides visualization results that intuitively display the outcomes of each annotation method (Fig. 1C).

### Performance evaluation

To evaluate and compare identification results and differential gene expression outcomes, we employ five commonly used metrics: Accuracy (Acc), F1-score, Matthews correlation coefficient (MCC), normalized mutual information (NMI), and adjusted mutual information (ARI). Detailed descriptions of these metrics are provided below.

#### Accuracy (Acc)

Accuracy is a fundamental metric for evaluating the performance of a classification model. It measures the proportion of correctly classified instances out of the total number of instances, offering a straightforward assessment of model effectiveness. This metric is particularly suitable for balanced datasets, where class distributions are relatively even. Accuracy is defined as:


(1) \begin{eqnarray*}
Acc = \frac{TP+TN}{TP+TN+FP+FN}.
\end{eqnarray*}

*TP* (True Positives) refers to instances correctly classified as positive, and *TN* (True Negatives) denotes those accurately identified as negative. Conversely, *FP* (False Positives) represents instances incorrectly predicted as positive, while *FN* (False Negatives) indicates instances misclassified as negative.

#### F1-score

The F1-score is a crucial metric for evaluating classification models, particularly in scenarios with imbalanced class distributions. It is defined as the harmonic mean of precision and recall, providing a balanced measure that accounts for the trade-off between these two metrics. The F1-score is especially valuable when both false positives and false negatives carry significant consequences, as it condenses their impact into a single performance measure. Using the same definitions for TP, TN, FP, and FN as outlined above, the formula for the F1-score is:


(2) \begin{eqnarray*}
F1-score = \frac{2TP}{2TP+FP+FN}.
\end{eqnarray*}

The F1-score ranges from 0 to 1, with higher values indicating better performance, particularly in tasks where both types of errors need to be minimized.

#### Matthews correlation coefficient

The MCC is a versatile metric applicable to both binary and multi-class classification tasks. In multi-class settings, it evaluates overall performance by accounting for the entire confusion matrix, making it particularly useful for imbalanced datasets. The MCC ranges from −1 to 1, where 1 indicates perfect predictions, 0 reflects random guessing, and −1 signifies total disagreement. The generalized MCC for multi-class classification is calculated as:


(3) \begin{eqnarray*}
MCC=\frac{\sum _{k,l,m}(C_{kk}C_{lm}-C_{kl}C_{mk})}{\sqrt{\left(\sum _k(\sum _l C_{kl})(\sum _{l\ne k}C_{lk})\right) \left(\sum _k(\sum _l C_{lk})(\sum _{l\ne k}C_{kl})\right)}} .\\
\end{eqnarray*}

Here, $C_{ij}$ represents elements of the confusion matrix.

#### Normalized mutual information

NMI is a statistical metric used to measure the similarity between two sets of labels. It quantifies the amount of shared information between the two label sets, with values ranging from 0 to 1. A value of 1 indicates perfect agreement, while 0 signifies no relationship. Let $L_t$ denote the true label set and $L_p$ the predicted label set obtained from cell type identification. The NMI is calculated as follows:


(4) \begin{eqnarray*}
NMI = \frac{2 \times I(L_{t}, L_{p})}{H(L_{t}) + H(L_{p})}.
\end{eqnarray*}

Where the mutual information $I(L_t, L_p)$ and entropy $H(L_t)$ are defined as:


(5) \begin{eqnarray*}
I(L_{t}, L_{p}) = \sum _{l_{t} \in L_{t}} \sum _{l_{p} \in L_{p}} P(l_{t},l_{p})\log \frac{P(l_{t},l_{p})}{P(l_{t})P(l_{p})},
\end{eqnarray*}

(6) \begin{eqnarray*}
H(L_{t}) = -\sum _{l_{t} \in L_{t}}P(l_{t}) \log P(l_{t}).
\end{eqnarray*}

Here, $I(L_t, L_p)$ measures the shared information between $L_t$ and $L_p$, with $H(L_t)$ and $H(L_p)$ representing the entropy of the true and predicted label sets, respectively. $P(l_t, l_p)$ is the joint probability of $l_t$ in $L_t$ and $l_p$ in $L_p$, while $P(l_t)$ and $P(l_p)$ are the marginal probabilities of $l_t$ and $l_p$.

#### Adjusted mutual information

ARI is also a widely used metric for evaluating the similarity between two clustering label assignments, accounting for the possibility of chance agreement. Unlike the traditional Rand Index, ARI adjusts for random labeling, ensuring that random cluster assignments yield a score close to zero. The ARI score ranges from *-1* to 1, where 1 indicates perfect agreement between the two clustering results, 0 represents a similarity equivalent to random clustering, and negative values imply clustering outcomes that are worse than random. The ARI is computed using the following formula:


(7) \begin{eqnarray*}
ARI = \frac{\sum _{ij}(\frac{n_{ij}}{2})-[\sum _{i}(\frac{a_{i}}{2}) \sum _{j}(\frac{b_{j}}{2})]/(\frac{n}{2})}{\frac{1}{2}[\sum _{i}(\frac{a_{i}}{2})+\sum _{j}(\frac{b_{j}}{2})]-[\sum _{i}(\frac{a_{i}}{2})\sum _{j}(\frac{b_{j}}{2})]/(\frac{n}{2})}.
\end{eqnarray*}

Where $n_{ij}$ represents the number of elements shared between cluster *i* in the true labels and cluster *j* in the predicted labels. The term $a_i$ denotes the total number of elements in cluster *i* of the true labels, $b_j$ corresponds to the total number of elements in cluster *j* of the predicted labels, and *n* is the total number of elements across all clusters.

Furthermore, the Ranking Index (RI) was employed to evaluate the overall performance of each algorithm, as proposed by [30]. The RI for a given algorithm *i* is calculated as:


(8) \begin{eqnarray*}
RI_i = \sum _j B(v_{ij}),
\end{eqnarray*}

where *B(· )* is the Heaviside step function, defined as *B(x) = 0* if *x < 0* and *B(x) = 1* if *x ≥ 0*. In this context, $B(v_{ij})$ indicates whether algorithm *i* is among the top-performing algorithms for metric *j*. Specifically, it reflects whether the algorithm ranks within the top 50% for that metric. The dataset-specific RI provides a measure of an algorithm’s relative performance on a particular dataset by counting the number of metrics for which it ranks in the top 50%. For instance, if an algorithm ranks in the top 50% for three different metrics on dataset A, its dataset-specific RI for that dataset would be 3. The RI offers a straightforward yet effective way to summarize and compare the performance of different algorithms across multiple evaluation metrics.

### Downstream analysis tools

Following the annotation of the single-cell dataset, scSuperAnnotator can conduct downstream analyses at both the gene level and cell level to comprehensively investigate the studied conditions. At the gene level, differential expression genes (DEGs) and enrichment analyses were performed to identify key genes and pathways underlying functional changes. At the cell level, trajectory inference was used to reconstruct developmental lineages, while cell–cell communication analysis explored intercellular signaling networks. Together, these analyses provide a holistic view of the molecular and cellular mechanisms driving the observed phenotypic changes. To implement these analyses, we utilized a range of specialized tools and algorithms tailored to each task. Below, we provide a detailed description of the methods and software applied in each analysis.

#### Differential expression gene analysis

scRNA-seq provides crucial insights into the stochastic nature of gene expression and its role in cell type differentiation. For DEG analysis, we utilized Scanpy’s *sc.tl.rank_genes_groups* function to group cells based on their cell type annotations derived from the respective identification methods [31]. The Wilcoxon test (method=’wilcoxon’) was used, with default settings for other parameters. The analysis identified genes with a log-fold change (logFC) *≥*3, highlighting the top five DEGs for each cell type compared to all other cells.

#### Gene Ontology enrichment

GO enrichment analysis identifies overrepresented biological processes, molecular functions, and cellular components within a given gene set, providing insights into the underlying biological mechanisms. We applied the *enrichGO* function from the *clusterProfiler* package to determine the top 10 enriched pathways for each category. The results of the GO enrichment analysis highlight the top 10 enriched pathways based on −log_10_(*P*-value) for biological processes, cellular components, and molecular functions for each cell type by method [32].

#### Kyoto Encyclopedia of Genes and Genomes enrichment

KEGG enrichment analysis is used to identify overrepresented pathways within a gene set, providing functional insights by linking genes to metabolic and signaling pathways. We used the *enrichKEGG* function from the *clusterProfiler* package to identify the top 20 enriched pathways based on the GeneRatio for each cell type identified by each method. An example of the KEGG results visualization in scSuperAnnotator is shown in Supplementary Fig. S81.

#### Cell–cell communication analysis

Cell–cell communication analysis reveals intercellular signaling networks by identifying ligand-receptor interactions between different cell types. We utilized the *CellChat* package to infer communication networks from scRNA-seq data [33], enabling the quantification of the number and strength of cell–cell interactions, the assessment of signaling pathway activities, and the identification of signaling patterns across cell types, providing critical insights for further biological research.

#### Trajectory inference

Trajectory inference provides a framework for reconstructing dynamic biological processes, such as cell differentiation, along a developmental continuum. We employed *Monocle2* to infer pseudotime trajectories from high-dimensional scRNA-seq data, enabling the mapping of cellular transitions and lineage relationships and providing a comprehensive view of the temporal progression of gene expression changes associated with development and differentiation processes [34].

### Result report

scSuperAnnotator is equipped with a comprehensive set of visualization and analysis tools for presenting cell type identification results and their downstream analyses. The platform provides a detailed page for each task, which includes the reference and query datasets used, the selected cell type identification method(s), the current status of the task, and a suite of powerful visualization tools. The visualization and analysis capabilities of scSuperAnnotator are organized into four main modules: (A) Reference Statistics, (B) Identification Results, (C) Gene Level Analysis, and (D) Cell Level Analysis. If the user opts for a downstream analysis when submitting a task, the visualization results of the corresponding gene or cell-level analysis will be displayed in the respective submodule.

#### (A) Reference Statistics module

In the Reference Statistics module, scSuperAnnotator conducts a comprehensive statistical analysis of the composition and distribution of the reference datasets. Through intuitive visualization tools, the module highlights key characteristics, including the presence of cell types, the degree of discretization, and gene expression patterns. These features enable users to evaluate the quality, reliability, and informational content of the dataset, enhancing their understanding of the reference data and improving their ability to evaluate the accuracy and relevance of annotation results.

#### (B) Identification Results module

The Identification Results module presents the annotation results of the query dataset through two sub-modules: Comparison and Method Evaluation. The Comparison submodule is available only when the true labels of the query dataset are known, using five metrics—Acc, F1-score, MCC, NMI, and ARI—to evaluate the performance of selected annotation methods. Results are presented quantitatively and visually through tables, bar charts, and radar charts that provide rankings of individual metrics for each method. Additionally, a comparison plot of RI is included to offer users a more comprehensive and intuitive assessment of the various annotation methods. The Method Evaluation submodule leverages UMAP plots to visualize the distribution of the query dataset after labeling by each method. When true labels for the query dataset are available, it also uses heatmaps to depict the correlation between the annotation results and the true labels, enabling a direct visual comparison of the cell type identification performance across methods. Furthermore, the submodule provides a comparison plot illustrating the correlation between cell types in the reference dataset and the annotated results from various methods, along with a plot showing the proportion of each cell type in both the reference dataset and the annotated results. A detailed example of the Identification Results Module is provided in Supplementary Note S13 and Supplementary Fig. S79. These visualizations enhance the understanding of changes in cell composition and relationships, such as those observed between diseased and healthy conditions, providing a foundation for more detailed biologically relevant analyses.

#### (C) Gene-level analysis module

The Gene-level Analysis module uses gene expression profiling to gain insight into cellular functions and biological processes. scSuperAnnotator platform provides a variety of tools, including functionality for identifying DEGs, as well as functionality for performing GO enrichment and KEGG enrichment analyses, to help users explore key biological pathways and functions, elucidate the molecular mechanisms of cellular diversity and disease, while providing insight into the genetic basis of cellular behavior and pathology.

#### (D) Cell-level analysis module

The Cell-level Analysis module allows the user to examine in detail the characteristics of individual cells and their interactions, which is essential for a comprehensive understanding of cellular heterogeneity, interrelationships between cells, and the function of different cell types in biological systems. Currently, the module provides analysis tools such as Cell Trajectory and Cell–Cell Interaction. The Cell Trajectory tool depicts the developmental and differentiation processes of cells, highlighting the dynamic changes of cells at different time points or states. By analyzing the distribution of cells at branch points and across various time points, this tool facilitates a deep exploration of the intrinsic mechanisms determining cell fate. Furthermore, the Cell–Cell Interaction Analysis examines the communication and influence between different cell types, enabling users to assess and validate the performance of various identification methods in the capture of cell–cell interactions. This contributes to a deeper understanding of intercellular interactions in biological systems, ensuring the reliability and biological relevance of cell-type identification methods.

### Web server implementation

scSuperAnnotator is empowered by a high-performance processing architecture with significant calculation capability. Specifically, scSuperAnnotator runs on the Ubuntu 24.04 Linux system, equipped with multiple AMD EPYC 7B12 64-Core Processor CPUs, 128 GB of RAM, 1 TB Solid State Drives, and NVIDIA RTX 3090 GPU clusters. Additionally, Fig. 1B shows that the front-end pages are carefully designed to provide an interactive visualization of results, with options for users to freely download each result. To be specific, we use the Bootstrap framework to set up the user interface, the Django framework for backend server implementation, Python and R programming languages for model construction and visualization, and the MongoDB database for data storage management. Moreover, to enhance the web experience, we also construct interactive graphs using Plotly’s Python graphing library (https://plotly.com/python/) to visualize the analysis results on the homepage. Our platform can be run stably on many browsers, including Internet Explorer, Mozilla Firefox, Microsoft Edge, Safari, and Google Chrome. In conclusion, with our scSuperAnnotator platform, data scientists and researchers limited by equipment resources can now employ high-performance computers to tackle their challenging work.

## Results

### General overview of reference datasets

**Figure 2.**
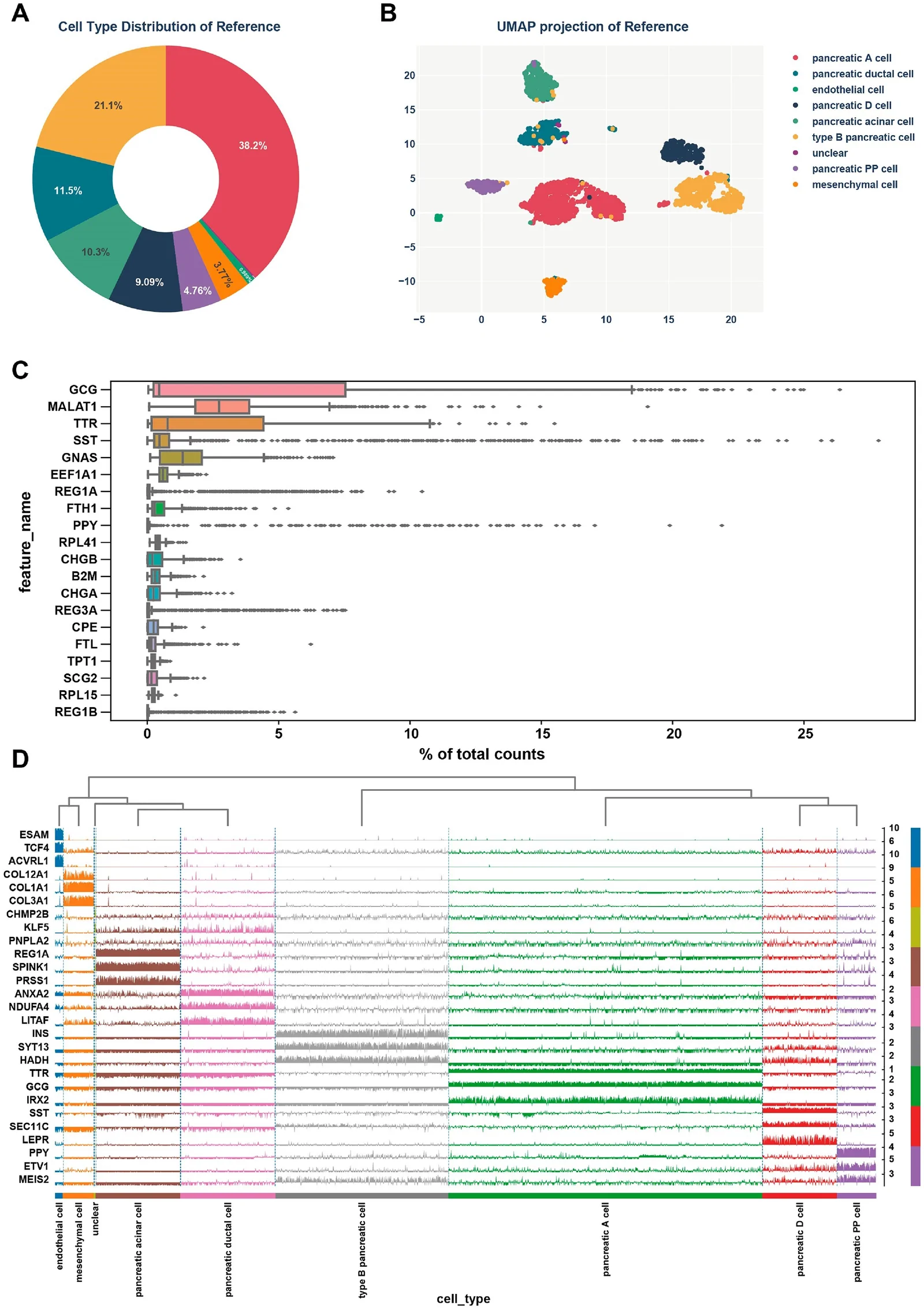
(**A**) Cell type composition of the reference dataset. (**B**) UMAP visualization of cell type clusters in the reference dataset. (**C**) Expression distributions of the top 20 genes contributing the highest fraction of total counts per cell across the dataset. (**D**) Expression profiles of representative marker genes across different cell types in the reference dataset.

The quality of reference datasets is a critical determinant in reference-based cell type identification. Key factors such as dataset composition, cell type proportions, and overall data quality substantially affect the performance of annotation models. High-quality reference datasets enable accurate cell type classification. Consequently, careful selection and evaluation of reference datasets are essential for obtaining meaningful identification results. To underscore the importance of reference quality, we use the archived human Muraro dataset as an example, providing visualizations that highlight key aspects of reference datasets from multiple perspectives [35]. Fig. 2A presents the distribution of cell types within the reference dataset, illustrating the proportions of 10 cell types in the human Muraro dataset. Pancreatic A cells constitute the largest fraction (38.2%), followed by type B pancreatic cells (21.1%) and pancreatic ductal cells (11.5%). Other notable cell types include pancreatic acinar cells (10.3%) and pancreatic D cells (9.08%), while endothelial and unclear cell types each represent <1%. This distribution reflects the dominance of specific cell types in the dataset. Fig. 2B provides a dimensionality reduction visualization, where most cell types, such as pancreatic A cell, type B pancreatic cell, and pancreatic ductal cell, form distinct clusters, indicating clear separation in feature space. In contrast, smaller clusters, such as those corresponding to endothelial and unclear cells, suggest rare or challenging-to-classify cell types. Together, Fig. 2A and B offer a detailed view of the dataset’s characteristics, confirming the high quality of the reference data and highlighting the strong biological signal captured in these scRNA-seq measurements.

In addition to visualizing cell type distributions, we further analyzed and visualized gene expression patterns, providing deeper insights into the reference dataset and a more comprehensive understanding of its characteristics and underlying biological signals. Fig. 2C illustrates the expression distribution of key genes, prominently showcasing a dominant GCG signal that reflects the high abundance of pancreatic A cells in the dataset. The expression diversity of other marker genes, such as MALAT1 and TTR, highlights the presence of multiple cell types, emphasizing the dataset’s biological complexity. This variation, combined with a strong signal-to-noise ratio in major genes, underscores the dataset’s reliability as a high-quality reference for cell type annotation and downstream analyses. Fig. 2D further demonstrates gene expression patterns across various cell types, revealing distinct clusters and cell-type-specific markers, such as GCG for pancreatic A cells and INS for type B pancreatic cells. The hierarchical clustering and clear separation of cell types highlight the dataset’s robust biological signals and authentic cellular identities, demonstrating its diversity and specificity in expression profiles and establishing it as a reliable reference for scRNA-seq analysis, cell type annotation, and other applications, while further validating its utility as a high-quality benchmark.

In conclusion, the presented figures confirm the quality and suitability of the reference dataset for downstream analyses, highlighting its diverse and accurately annotated cellular landscape and establishing it as a reliable resource for benchmarking and validating scRNA-seq identification methods.

### Benchmarking annotation methods in same-platform scenarios

Same-platform label transfers approximate a ceiling for reference-based annotation and isolate method effects from domain shift; accordingly, we benchmarked 10 annotation methods on 12 same-platform reference–query pairs spanning six platforms (10x, Drop-seq, inDrop, Microwell-seq, Seq-Well, and Smart-seq2) under a unified pipeline (Supplementary Table S2), quantifying Acc, F1-score, MCC, NMI, ARI, and inspecting UMAP label coherence. Preprocessing was standardized (QC filtering, normalization, highly variable gene selection, dimensionality reduction), healthy and diseased references were not mixed, and species/tissue were matched within each pair.

**Figure 3.**
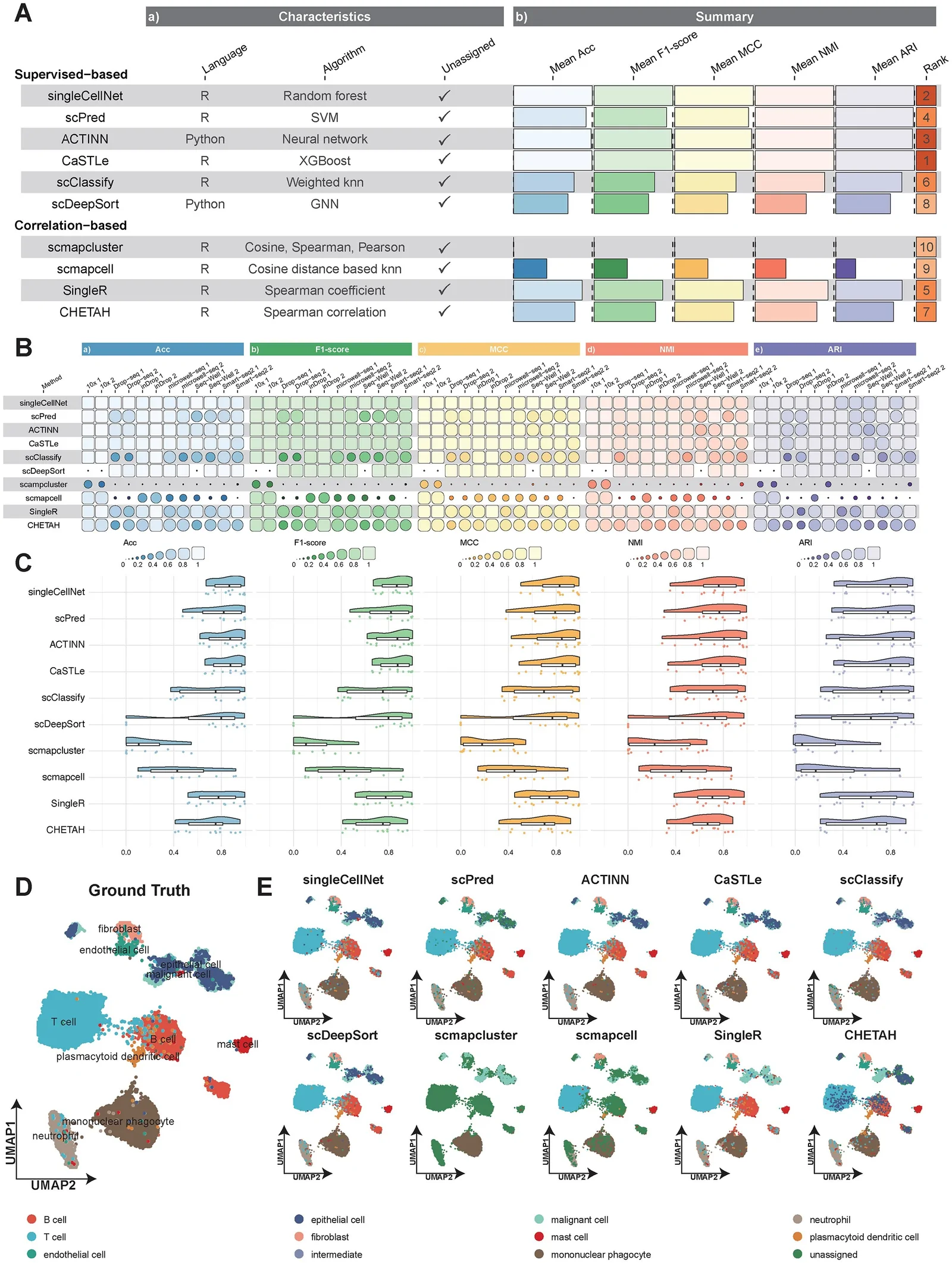
(**A**) Summary of method performance across multiple evaluation aspects under the same-platform condition. (**B**) Detailed comparison of five evaluation metrics for each method across datasets under the same-platform condition. (**C**) Distributional comparison of five evaluation metrics for each method across datasets. (**D**) Ground Truth UMAP for the inDrop 1 query dataset. (**E**) UMAP comparison of method-specific annotations on the inDrop 1 query dataset.

Fig. 3A reveals a clear stratification: CaSTLe [46] leads, followed by singleCellNet and ACTINN; scPred and SingleR form the next tier. Leaders maintain uniformly high bars across all five metrics, indicating strong central performance not driven by any single measure; mid-tier methods show metric-specific dips, and the trailing group is consistently lower across metrics. Supervised approaches dominate the upper ranks, with SingleR the strongest among correlation-based baselines. Per-dataset and distribution views in Fig. 3B and C corroborate this ordering: top methods sustain high scores with limited dispersion across datasets, whereas lower-ranked methods show recurrent, multi-metric declines—evidence of systematic deficits rather than dataset idiosyncrasies. Spatial overlays further support the ranking: on an inDrop query, the ground-truth UMAP in Fig. 3D shows well-separated clusters, and higher-ranked methods (CaSTLe, singleCellNet, ACTINN) preserve boundaries with cluster-coherent coloring and only minor bleed, while scmapcluster, scmapcell, and CHETAH exhibit cross-cluster mixing and fragmented labels (Fig. 3E); supplementary UMAPs across remaining datasets (Supplementary Figs S3–S13) reproduce this rank–coherence relationship. Overall, same-platform evidence separates methods into stable tiers—a leading tier with high metrics and coherent embeddings, a middle tier with dataset-dependent dips, and a trailing tier with recurrent deficits—implying differences are primarily method-intrinsic. For same-platform applications, we recommend prioritizing CaSTLe, singleCellNet, and ACTINN, with scPred or SingleR as alternatives, and using nearest-neighbor or hierarchical schemes (scmapcluster, scmapcell, CHETAH) with caution (Supplementary Table S3).

### Benchmarking annotation methods in cross-platform scenarios

In practice, references and queries are rarely generated using the same platform or protocol. Differences in protocol and preprocessing—UMI versus full-length profiling (10x versus Smart-seq2), capture efficiency and read depth, ambient RNA or background, chemistry and version effects, and site-specific pipelines—introduce domain shift that warps expression geometry (dropout patterns, gene-length bias), distorts distance metrics, and miscalibrates classifier probabilities. These effects inflate in-domain accuracy yet degrade transfer performance, especially in pathology, where tissue damage and inflammation amplify technical drift. To quantify generalization under these real-world conditions and to provide actionable guidance, we conducted a dedicated cross-platform evaluation.

**Figure 4.**
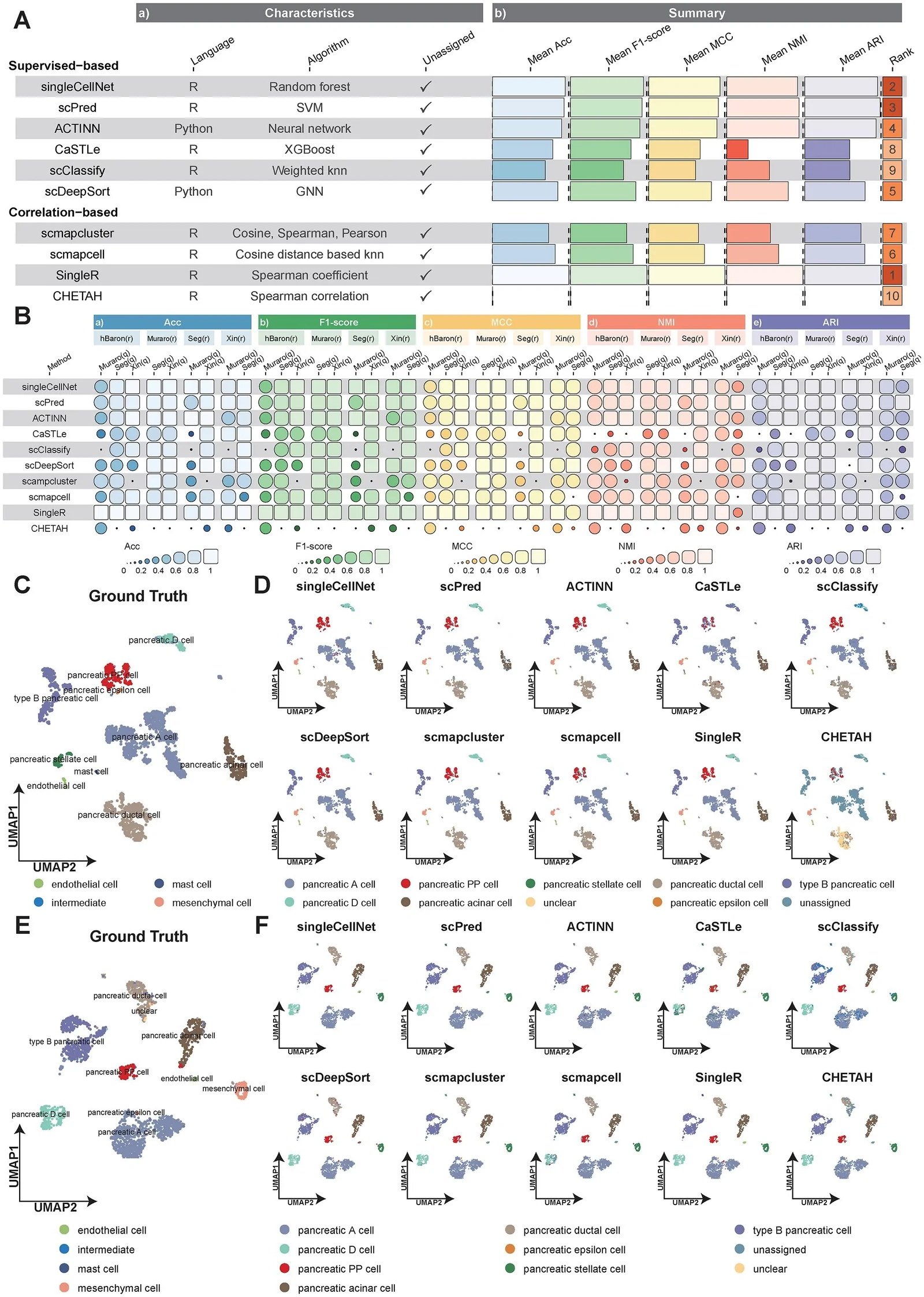
(**A**) Summary of method performance across multiple evaluation aspects under the cross-platform condition. (**B**) Detailed comparison of five evaluation metrics for each method across datasets under the cross-platform condition. (**C**) Ground truth UMAP of the Segerstolpe (Smart-seq2) query dataset. (**D**) UMAP of the Segerstolpe (Smart-seq2) query showing method-specific predicted cell type annotations using Muraro (CEL-seq2) as the reference. (**E**) Ground truth UMAP of the Muraro (CEL-seq2) query dataset. (**F**) UMAP of the Muraro (CEL-seq2) query showing method-specific predicted cell type annotations using Segerstolpe (Smart-seq2) as the reference.

We assessed 10 reference-based annotation methods under cross-platform shift using nine reference–query pairs and a unified pipeline (Supplementary Table S4), reporting Acc, F1-score, MCC, NMI, and ARI at aggregate and per-dataset levels. Fig. 4A shows a stable ordering: SingleR achieves the highest aggregates; singleCellNet and scPred form a consistent second tier; ACTINN and scDeepSort [47] are competitive but more sensitive to reference–query composition; scmap exhibits higher between-pair variance; CaSTLe and scClassify [48] rank lower; and CHETAH is lowest. The per-dataset dispersion in Fig. 4B corroborates this: top methods cluster at higher values with limited spread, mid-tier methods show moderate dispersion with pair-specific fluctuations, and the lowest group displays the widest spreads with occasional sharp drops. A representative transfer from Muraro (CEL-seq2) to Segerstolpe (Smart-seq2) [36] (Fig. 4C–F) illustrates these behaviors: all methods preserve global topology, but robustness differs in cluster compactness and boundary continuity, with SingleR closest to ground truth and lower-ranked methods showing increased within-cluster variability or fragmentation. Supplementary UMAPs across remaining datasets (Supplementary Figs S14–S20) reproduce this rank–coherence relationship. Overall, the nine evaluations indicate that methods differ chiefly in robustness to platform shift, not just peak accuracy on a single dataset (Supplementary Table S5).

### Benchmarking annotation methods in cross-species scenarios

**Figure 5.**
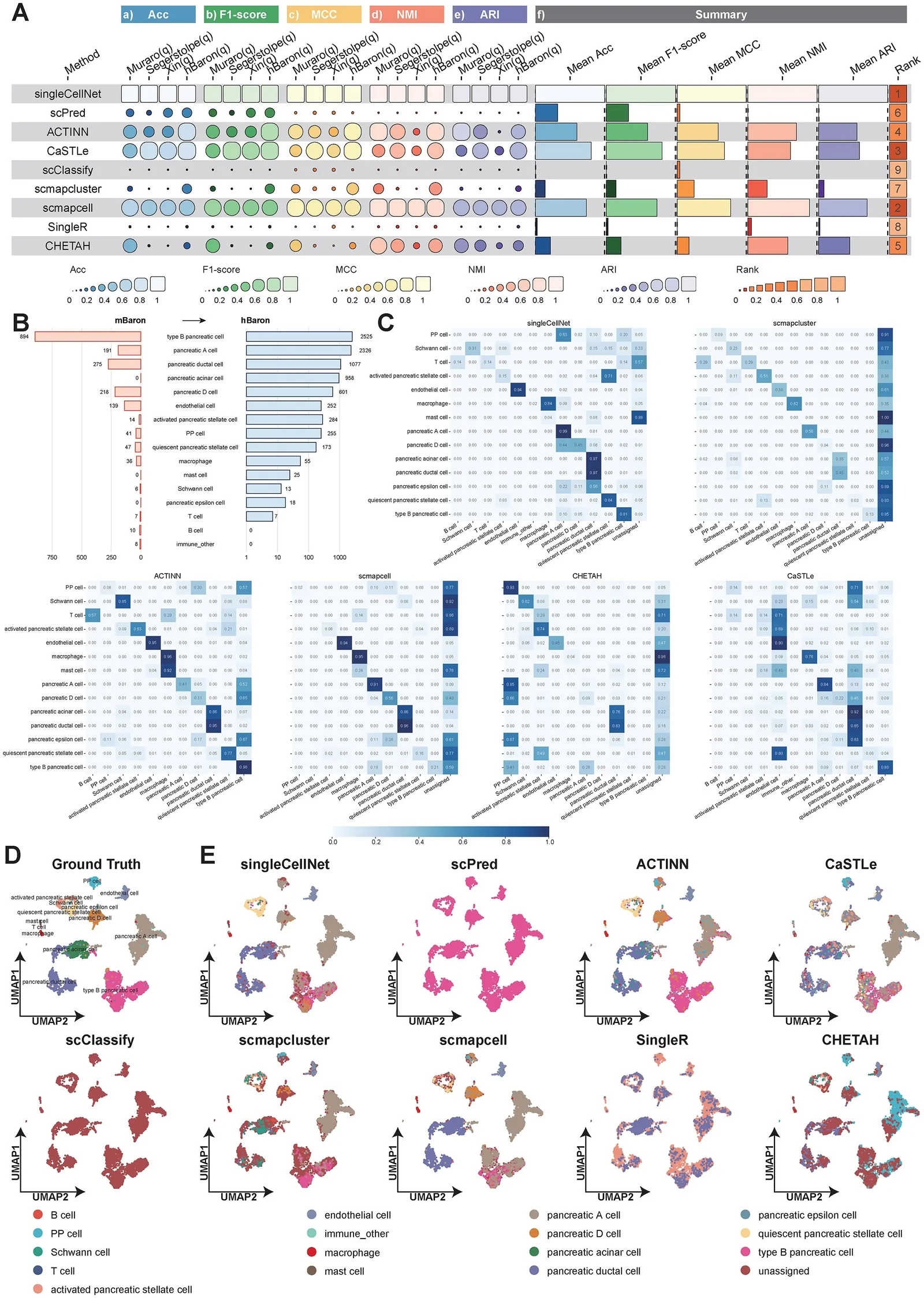
(**A**) Cross-species summary of method performance across evaluation aspects with per-dataset comparisons of five metrics for each method. (**B**) Cell-type composition of the mBaron reference and hBaron query datasets, reporting the number of cells per type in each dataset. (**C**) Comparison of method-specific annotations against the ground truth on the hBaron query using mBaron as the reference. (**D**) Ground truth UMAP of the hBaron query dataset. (**E**) UMAP of the hBaron query showing method-specific predicted cell type annotations using mBaron as the reference.

High-quality annotated human references are often limited by ethical and access constraints, while many disease studies are conducted in mouse and core cell lineages/programs are well conserved; consequently, cross-species annotation becomes necessary. Against this backdrop, we used mBaron (mouse) [37] as the reference and evaluated nine reference-based methods on four human queries under a unified pipeline (Supplementary Table S6), summarizing Acc, F1-score, MCC, NMI, and ARI. Fig. 5A integrates per-dataset results and overall means and yields a consistent hierarchy: singleCellNet ranks first; scmapcell and CaSTLe follow; ACTINN and CHETAH form a middle tier; and scPred, scmapcluster, SingleR, and scClassify rank lower. The per-dataset panels and summary columns agree that leading methods sustain high scores across metrics and across all four queries (driving higher means and top ranks), the middle tier shows query-specific declines that depress overall means and ranks, and the lowest group exhibits uniformly low scores—indicating that the ordering reflects performance reproducible across datasets and metrics rather than isolated fluctuations in the cross-species setting.

Using mBaron as reference and hBaron as query, Fig. 5B shows that the datasets share major pancreatic lineages, albeit unevenly represented in the reference; Fig. 5C confirms that greater reference support for a shared lineage yields higher agreement, concentrating correlations along the diagonal. singleCellNet exhibits the clearest diagonal among shared types, with CaSTLe showing a similar concentration. When a shared lineage has few reference cells in mBaron, correct matches decline: CHETAH and scmapcell recover many shared types but retain a prominent “unassigned” column; scmapcluster shows the strongest “unassigned” signal. These label-level patterns track the availability and counts of shared lineages and are consistent with the ranking in Fig. 5A—methods that maintain diagonal alignment across shared types and remain stable when reference counts are low occupy the higher positions.

UMAP projections in Fig. 5D and E visualize transfer from mBaron to hBaron: the ground-truth map separates endocrine and exocrine compartments; singleCellNet largely reproduces these partitions with cluster-coherent labeling, though type B pancreatic cell regions are only partially recovered and PP cells are poorly identified—consistent with limited PP support in Fig. 5B. scmapcell, CaSTLe, and ACTINN preserve the global layout but mix labels within several clusters; scClassify and scmapcluster leave most cells unassigned; scPred collapses much of the embedding onto the dominant type B pancreatic cell label; SingleR and CHETAH show patchy alignment. Across the additional datasets (Supplementary Figs S21–S23), correlation maps and UMAPs recapitulate these patterns, and the ranking remains stable. Higher-ranked methods keep predictions along the diagonal and retain cluster boundaries, indicating reliable structural transfer; lower-ranked methods exhibit two recurring errors—extensive “unassigned” or collapse to a single label—weakening the diagonal and fragmenting the spatial layout. Overall, cross-dataset consistency suggests the hierarchy is driven primarily by method-specific transfer behavior with residual variation due to dataset composition; for mouse*→*human annotation, we therefore recommend singleCellNet as default, with CaSTLe or scmapcell as alternatives (Supplementary Table S7).

### Benchmarking annotation methods for unknown cell type detection

References can lack relevant types due to cohort scope or curation gaps, causing misassignment and biased biological conclusions. To directly test Unknown (open-set) detection, we simulated class absence by removing pancreatic A cells from hBaron, then annotated Muraro, Segerstolpe, Xin, and Fasolino (Supplementary Table S8), and computed Acc, F1-score, MCC, NMI, and ARI under a unified pipeline [38, 39].

**Figure 6.**
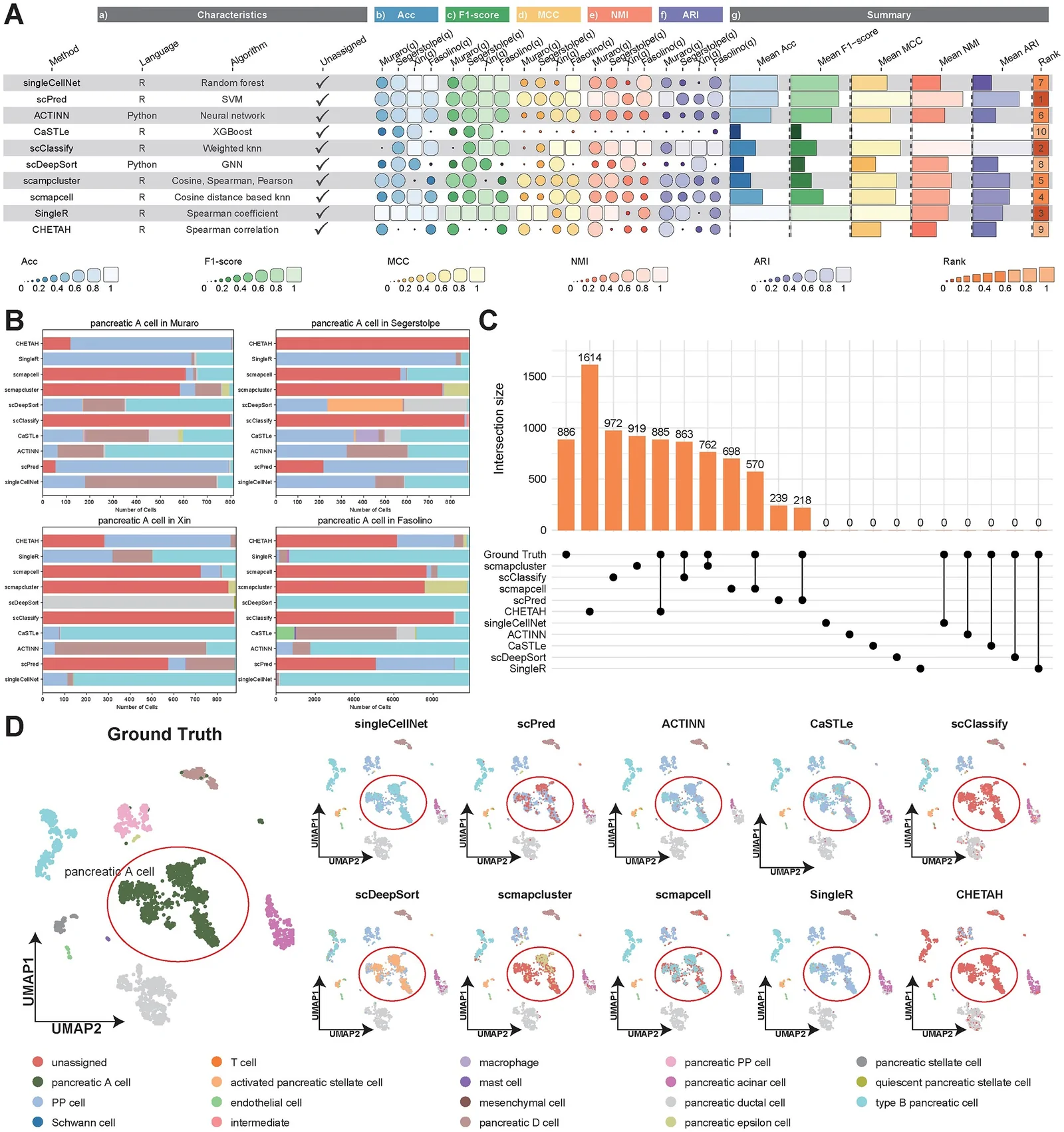
**(A**) Summary of method performance in unknown cell type detection across evaluation aspects with per-dataset comparisons of five metrics for each method. (**B**) Label-level behavior in the missing-class setting illustrated by partitioning ground-truth pancreatic A cells into unassigned and reassigned labels. (**C**) Label-level behavior in the missing-class setting was further quantified on the Segerstolpe dataset by partitioning pancreatic A cells into unassigned abstentions and reassigned labels and examining co-occurrence patterns across methods. (**D**) Ground truth and method-specific predicted cell-type annotations of the Segerstolpe query dataset with differences in counts localized to the pancreatic A-cell region.

Fig. 6A shows a coherent, reproducible ordering: scPred leads with high aggregates and low cross-dataset dispersion; scClassify and SingleR follow closely with strong aggregates and compact spreads; scmapcell and scmapcluster form an upper-middle tier with competitive aggregates but intermediate rank, reflecting moderate attenuation on some datasets; singleCellNet and ACTINN sit in a middle tier with visibly larger dispersion; scDeepSort and CHETAH fall below this tier with lower aggregates and broader spreads; CaSTLe consistently shows the smallest per-dataset markers and depressed aggregates, placing it last.

Label-level behavior of the missing class is summarized in Fig. 6B by partitioning true pancreatic A cells into “unassigned” (abstained) versus reassigned labels. scClassify yields the largest Unknown fraction on all four datasets (consistently conservative when the target class is absent). CHETAH abstains strongly on Segerstolpe but much less on Muraro, Xin, and Fasolino, highlighting dataset dependence. scmapcluster and scmapcell are intermediate, with substantial but not uniform unassigned calls. scPred makes a few unassigned calls (high precision, lower recall of the missing class). scDeepSort, SingleR, singleCellNet, ACTINN, and CaSTLe rarely abstain and predominantly reassign pancreatic A cells to existing labels. Fig. 6C quantifies these trade-offs on Segerstolpe (886 pancreatic A cells): CHETAH abstains on 885/886 pancreatic A cells but also flags 729 non-pancreatic A cells as unassigned (near-complete recall with extensive over-abstention); scClassify attains high recall (863 pancreatic A cells of 972 unassigned total) with a more favorable precision–recall balance; scmapcluster is intermediate (762/919), scmapcell more conservative (570/698), and scPred is most selective (239 unassigned capturing 218 pancreatic A cells), implying high precision but low recall. The remaining methods rarely use Unknown and instead reassign pancreatic A cells. In short, methods vary along a single trade-off: leave many cells unassigned and recover most true pancreatic A cells, or assign labels aggressively and miss many pancreatic A cells.

Spatial patterns on the Segerstolpe UMAP in Fig. 6D mirror the counts. Abstention-heavy methods (CHETAH, scClassify) label most of the pancreatic A-cell region as unassigned (with CHETAH also flagging scattered off-region areas, consistent with over-abstention in Fig. 6C). Intermediate methods (scmapcluster, scmapcell) produce mosaics of Unknown interleaved with other labels. scPred leaves small unassigned islands within a largely reassigned region. Methods that rarely abstain (SingleR, singleCellNet, ACTINN, CaSTLe) mainly reassign the pancreatic A-cell area to nearby cell types, yielding fragmented coloring. Similar behavior appears in Muraro, Xin, and Fasolino (Supplementary Figs S24–S26).

Overall, robustness to a missing class is driven by method-intrinsic open-set strategy rather than dataset idiosyncrasies: strong methods keep aggregates stable across datasets, abstain from the absent population rather than forcing mismatched labels, and preserve global embedding organization. Practically, when a relevant population may be absent from the reference, prefer scClassify when high Unknown recall is desired, scPred when high Unknown precision is preferred (Supplementary Table S9).

### Benchmarking annotation methods across marker databases

High-quality, disease-matched references are often unavailable or incomplete, so practitioners frequently turn to marker-based annotation methods for cell-type labeling. However, public marker repositories differ in curation depth, lineage granularity, and naming standards, producing non-overlapping gene lists even for the same nominal type. These discrepancies propagate to automated labels—shifting accuracy and cluster coherence—and complicate method-agnostic guidance. To make these effects explicit and provide practical recommendations, we conducted a dedicated benchmark. Specifically, to quantify how marker resources and marker-based annotators shape automated cell-type labeling, we evaluated CellAssign, Garnett, and SCINA on six public datasets using five widely used marker sources (CellMarker2.0, PanglaoDB, CellMatch, MSigDB, and CELLxGENE), plus an integrated, standardized database from Cell Marker Accordion, under a unified pipeline reporting Acc, F1-score, MCC, NMI, and ARI [40, 41] (Supplementary Table S10).

**Figure 7.**
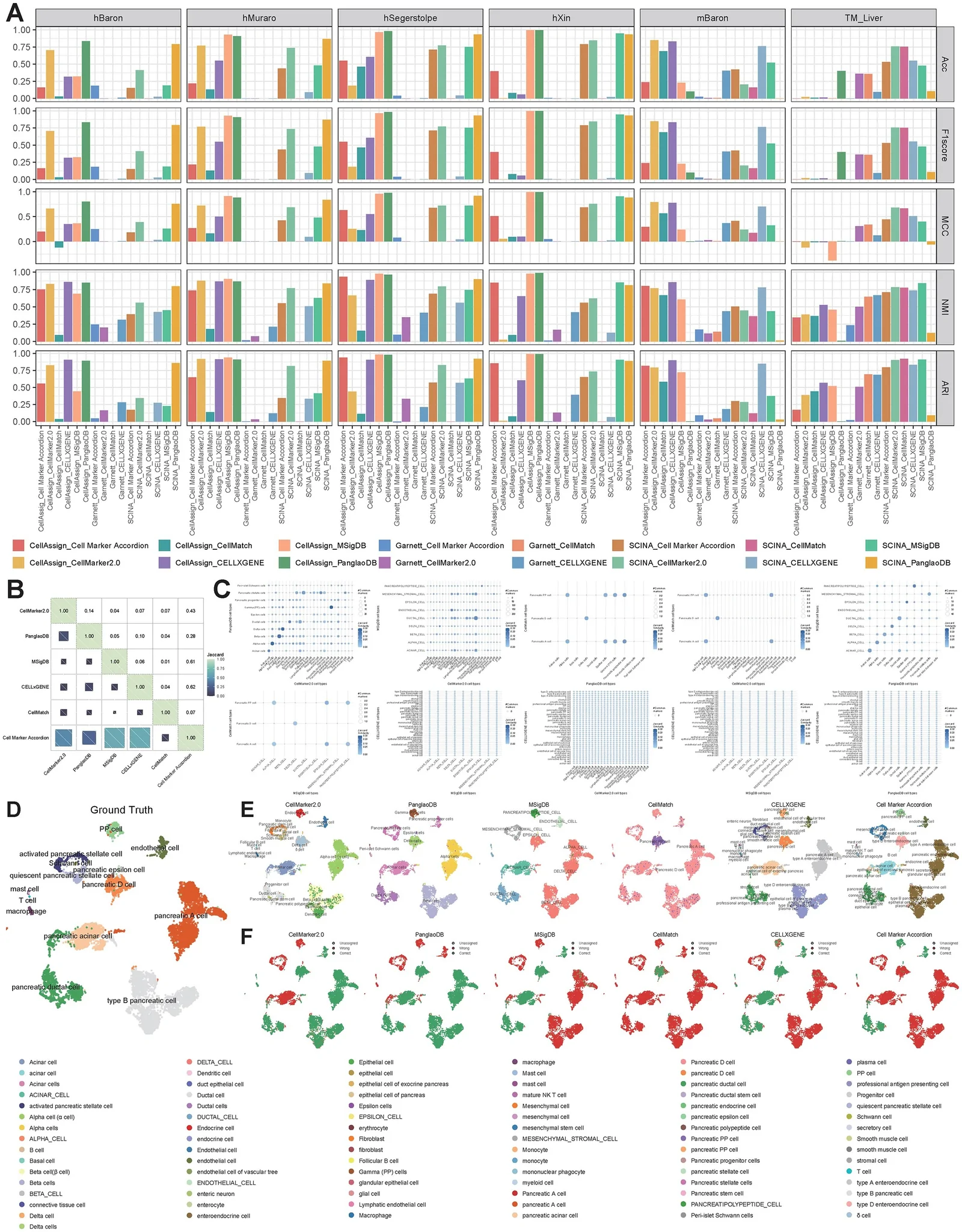
(**A**) Benchmark of marker-based annotation methods across six marker databases, evaluated by Acc, F1-score, MCC, NMI, and ARI. (**B**) Pairwise Jaccard similarity of cell-type marker sets across six databases, averaged over shared cell types. (**C**) Overlap of marker genes across databases for hBaron cell types annotated by CellAssign with different marker resources, in which dot color encodes the Jaccard similarity and dot size indicates the number of shared markers for each cell-type pair. (**D**) Ground truth UMAP of the hBaron query dataset. (**E**) UMAPs of the hBaron query showing CellAssign-predicted annotations using different marker gene sets. (**F**) UMAPs comparing CellAssign annotations from different marker gene sets with ground truth.

Fig. 7A shows that marker-set choice strongly modulates performance across datasets and methods: no single published resource is uniformly best, and a set that excels on one dataset may underperform on another. In contrast, the marker database of Cell Marker Accordion produces more stable profiles and reduces between-dataset variability. Within each dataset, the spread of bar heights indicates that marker selection explains more variance than the identity of the classifier, implying that—holding ground truth fixed—differences in curation and coverage can outweigh algorithmic differences. Content analysis in Fig. 7B confirms limited agreement among individual databases (maximum Jaccard 0.14), reflecting heterogeneous gene lists and nomenclature; integration and standardization of the marker database increases concordance with each source (mean Jaccard 0.40; maximum 0.62), recovering shared signal and attenuating source-specific idiosyncrasies. At the cell-type level, Fig. 7C examines cell-type-level effects using CellAssign on hBaron and illustrates how divergent marker content propagates to labels. We observe differences in terminology and shifts in lineage boundaries. Overlap is denser for broad and ubiquitous lineages, such as immune and endothelial, whereas overlap is sparse for tissue-specific subtypes, such as pancreatic endocrine subtypes. Consequently, the shared marker sets are smallest precisely where fine-grained annotation is most difficult, and resources tend to agree on canonical markers while diverging on subtype- or state-specific signatures.

Within-method comparisons on hBaron (Fig. 7D–F and Supplementary Figs S27 and  S28) show that curated, lineage-balanced resources (CellMarker2.0, PanglaoDB) yield cluster-coherent maps respecting ground-truth boundaries, whereas broader sets (CELLxGENE, Cell Marker Accordio database) often reduce Unassigned at the cost of within-cluster mosaics when cross-source markers conflict; less selective or lineage-biased sets (MSigDB, CellMatch) further erode coherence. With the marker set held constant, methods differ in how these effects manifest: SCINA frequently shows high Unassigned and intra-cluster inconsistency even as coverage increases, while Garnett is sensitive to coverage/completeness, with conflicts elevating incorrect or Unassigned labels and occasional training failures when required lineages lack sufficient markers; these trends recur across the remaining datasets (Supplementary Figs S29–S43).

Overall, high-quality, lineage-balanced markers are necessary but not sufficient for reliable automated annotation. Marker choice has a first-order impact on accuracy and cluster concordance, and method behavior then determines whether broader coverage stabilizes labels or instead produces mosaics and Unassigned. Cell Marker Accordion expands coverage and reduces variability across datasets, yet residual cross-source discrepancies may persist at the subtype level (Supplementary Table S11).

### Identification of potential disease-associated cell populations

**Figure 8.**
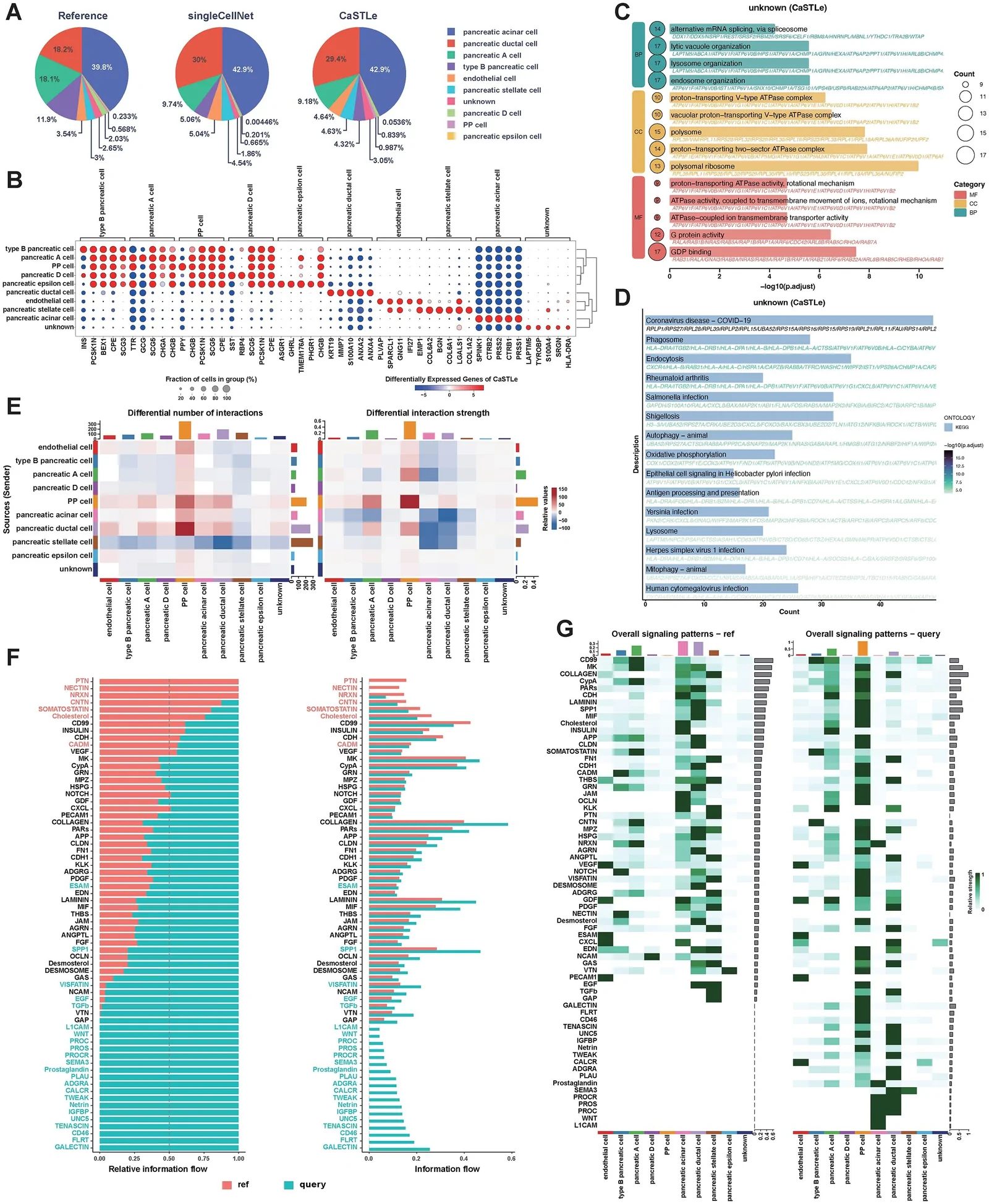
(**A**) Comparison of cell type proportions from each annotation method with those in the reference dataset. (**B**) DEGs across various cell types identified by CaSTLe. (**C**) GO enrichment in unknown cells across biological processes, cellular components, and molecular functions. (**D**) KEGG pathways enriched in unknown cells. (**E**) Depicts changes in the frequency and strength of cell–cell interactions in the T1D pancreas compared to healthy controls. (**F**) Comparison of relative and absolute information flow for specific proteins and signaling pathways between T1D (query) and healthy controls (reference), highlighting differences in intercellular communication under disease conditions. (**G**) Comparison of overall signaling patterns between the healthy reference dataset (ref) and the T1D dataset (query).

Cell type identification is critical for the discovery of disease-associated cell populations and the detection of subtle cellular differences between healthy and diseased states. By leveraging well-characterized reference datasets, supervised cell-type identification methods allow for precise annotation of scRNA-seq data, which can help identify novel or rare cell subtypes associated with disease progression or treatment response. In addition, reference-based methods standardize comparisons across methods, providing a robust framework for downstream experimental validation in the context of disease. Using the healthy pancreas dataset as a reference, we annotated the T1D pancreas dataset with singleCellNet and CaSTLe to demonstrate the role of cell type identification in disease-related biological discovery [39]. Fig. 8A compares the cell-type proportions in the T1D dataset with those in the healthy reference. The T1D pancreas shows a markedly reduced proportion of type B pancreatic cells (β cells), consistent with autoimmune β-cell destruction characteristic of T1D [42]. Conversely, pancreatic ductal cells are more abundant in T1D, likely due to challenges in isolating pure islets from T1D donors. Additionally, pancreatic A cells (α cells) are reduced in T1D, providing insights into potential glucagon dysregulation mechanisms linked to lower α-cell numbers or intrinsic defects [43].

Based on the RI (Supplementary Fig. S64), we conducted gene-level and cell-level analyses using CaSTLe identification results to demonstrate the utility of scSuperAnnotator for disease inference and analysis. The analyses based on singleCellNet identification results are also available in Supplementary Figs S69–S74. Fig. 8B illustrates gene expression patterns across pancreatic cell types, highlighting their specialized functions. Type B pancreatic cells (β cells) express high levels of INS (insulin), pancreatic A cells (α cells) show elevated GCG (glucagon) expression, pancreatic D cells (δ cells) express SST (somatostatin), and PP cells uniquely express PPY (pancreatic polypeptide), consistent with their roles in glucose regulation and hormone secretion. The “unknown” cells express immune-related markers, such as HLA-DRA, suggesting the presence of immune or antigen-presenting cells, potentially indicative of immune infiltration or non-pancreatic cell types relevant to T1D pathology. Fig. 8C (GO) and Fig. 8D (KEGG) describe a single, coherent program: high-flux vesicular trafficking with sustained acidification (endosome organization, lysosome organization, multiple V-type ATPase terms) coupled to degradative–recycling modules (Phagosome, Lysosome, Endocytosis, Autophagy, Mitophagy) and supported by metabolic/biosynthetic upshift (Oxidative phosphorylation, polysome/polysomal ribosome). The presence of Antigen processing and presentation and broad antiviral/bacterial response pathways situates this program within an immune-active inflammatory milieu rather than a purely housekeeping turnover state. In the context of T1D patient samples, such convergence is consistent with disease-relevant activity: intensified uptake, acidification, and processing create conditions that facilitate epitope generation and immune engagement in inflamed islets. Thus, the enrichment pattern should be interpreted as an immunologically engaged degradative state with metabolic reinforcement that is compatible with T1D pathophysiology while not being exclusive to it. GO and KEGG enrichment for the remaining annotations are presented in Supplementary Figs S65 and  S66. Building on these findings, disease mechanisms were further examined at the cell level. Fig. 8E highlights significant alterations in cell–cell interaction frequency and strength in the T1D pancreas compared to healthy controls, reflecting a reorganization of cellular communication networks. Notable findings include increased interactions from pancreatic ductal cells to PP cells, suggesting enhanced regulatory influences within the ductal microenvironment, and elevated autocrine PP–PP interactions, indicative of an adaptive response. Conversely, reduced interactions from pancreatic stellate cells to ductal and acinar cells, along with weakened acinar-ductal interactions, point to compromised cellular interdependence, potentially affecting pancreatic function. Fig. 8F reveals upregulated signaling in T1D through proteins and pathways linked to inflammation, immune modulation, and ECM remodeling, including GAP, VTN, TGFb, EGF, SPP1, PDGF, LAMININ, and COLLAGEN, suggesting structural changes and adaptive responses to tissue damage. Unique activation of pathways such as FLRT, TENASCIN, Netrin, and WNT in T1D underscores their roles in tissue repair and disease progression. Fig. 8G and Supplementary Figs S67 and  S68 further demonstrate enhanced signaling strength, particularly in PP cells, with the strongest correlations involving PP, acinar, and ductal cells, emphasizing their central role in T1D pathophysiology and potential as therapeutic targets.

Integrating cell-level and gene-level analyses after identifying cell types in disease-related datasets using healthy references is pivotal for uncovering disease-specific mechanisms. Cell-level analyses reveal disrupted communication networks and altered cellular interactions, reflecting changes in cellular dynamics, while gene-level analyses highlight variations in pathway activity and signaling, elucidating underlying molecular mechanisms. Together, these analyses underscore the value of cell annotation methods in advancing our understanding of disease pathophysiology and identifying potential therapeutic targets.

### Deciphering disease biology through cell signaling and trajectory analysis

**Figure 9.**
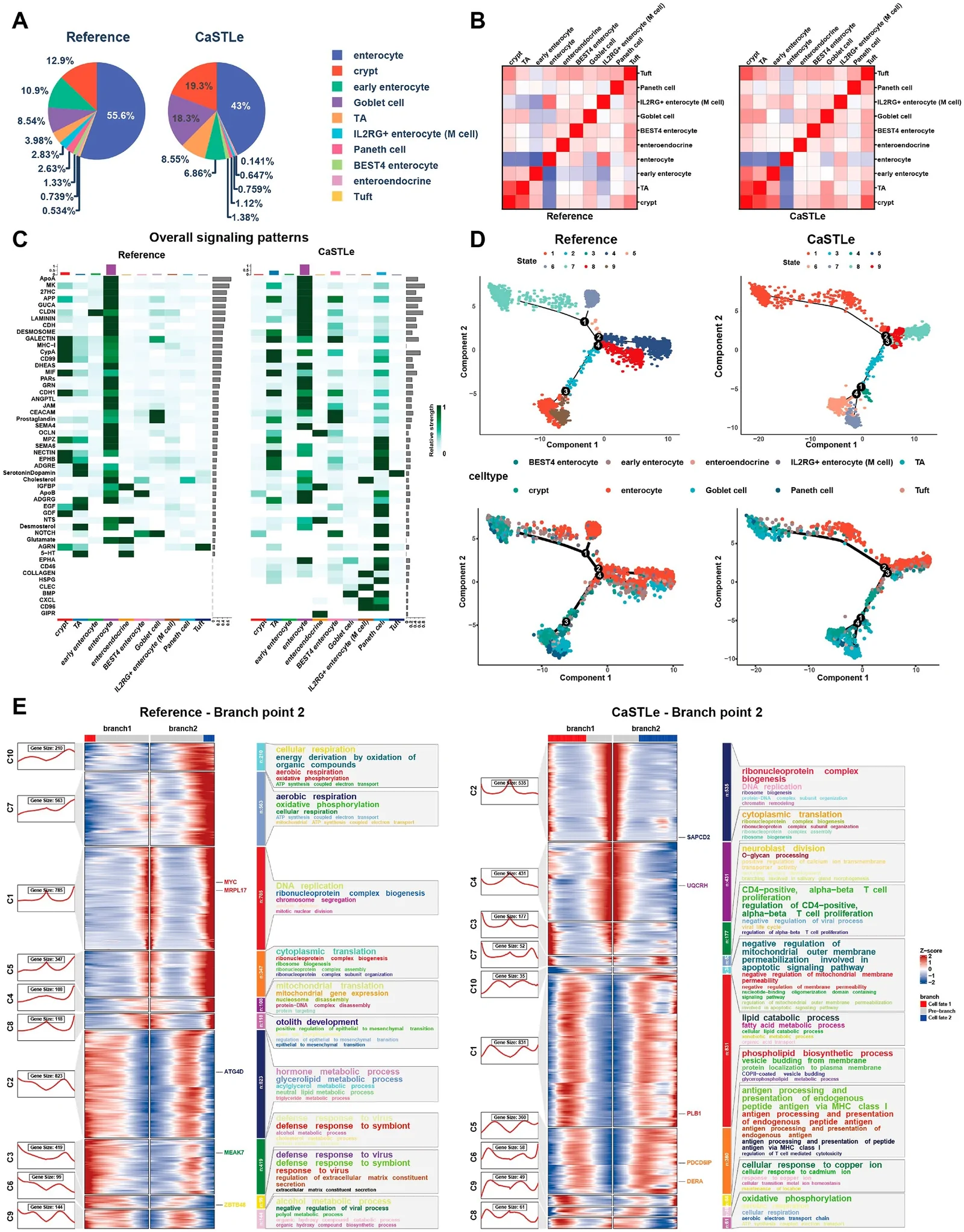
(**A**) Comparison of cell type proportions identified by CaSTLe with those in the reference dataset. (**B**) Comparison of correlations between intestinal epithelial cell subtypes in healthy individuals (left) and CD patients (right). (**C**) Comparison of overall signaling patterns between intestinal epithelial cell subtypes in healthy individuals (left) and CD patients (right). (**D**) Significant disruptions in epithelial differentiation. (**E**) Comparison of BEAM analysis at branch point 2.

The intestinal epithelium, a monolayer of cells, serves as a critical barrier, mediating neuro-immune-epithelial interactions with the gut microbiota to protect the host from luminal contents. In Crohn’s disease (CD), an inflammatory bowel disease, this barrier is impaired, resulting in increased intestinal permeability, immune dysregulation, and chronic inflammation [44]. Using the CaSTLe method, we annotated and analyzed small intestinal epithelial cells from children newly diagnosed with CD, comparing them to healthy children aged 4–12 years as a reference, to demonstrate the utility of scSuperAnnotator in elucidating disease mechanisms and guiding therapeutic strategies. Fig. 9A illustrates the epithelial cell composition in healthy (left) and CD (right) samples, highlighting a significant reduction in fully differentiated enterocytes (55.6% to 43%) in CD, indicative of impaired differentiation. Concurrently, there is an increase in TA cells (3.98% to 8.55%), Goblet cells (8.54% to 18.3%), and crypt cells (12.9% to 19.3%), consistent with crypt hyperplasia and secretory cell expansion driven by chronic inflammation. Fig. 9B compares epithelial subtype correlations, revealing altered interaction patterns in CD, particularly between BEST4 enterocytes and early enterocytes, suggesting disrupted nutrient absorption and epithelial regeneration. Increased correlations involving Goblet cells and IL2RG^+^ enterocytes reflect their heightened inflammatory response. Fig. 9C shows significant shifts in epithelial signaling pathways in CD, with GALECTIN signaling broadly upregulated, reflecting its role in inflammation and impaired communication. Elevated CEACAM and CXCL signaling in Paneth cells indicate immune activation and barrier dysfunction, while BMP and COLLAGEN pathways suggest compensatory repair mechanisms. These findings underscore key epithelial remodeling and signaling disruptions in CD, providing insights into pathogenesis and potential therapeutic targets.

Trajectory inference analyses, as shown in Fig. 9D, reveal significant alterations in epithelial differentiation in CD, driven by chronic inflammation and metabolic dysregulation. In the reference dataset, branch node 1 represents normal differentiation, while its absence in CD indicates impaired progenitor cell maturation. CD is marked by fragmented differentiation pathways, with branch node 3 in the reference dataset corresponding to stress-adapted and inflammatory phenotypes in CD. The emergence of a new branch node 1 in CD suggests an alternative inflammation-driven trajectory, while branch node 2 reflects skewed differentiation, and branch node 4 in the reference dataset aligns with branch node 3 in CD, indicating further pathway disruptions. BEAM analysis of branch node 2, depicted in Fig. 9E, shows that the reference dataset supports efficient differentiation through pathways related to oxidative phosphorylation, mitochondrial translation, and DNA replication. Conversely, CD exhibits mitochondrial metabolic reprogramming, lipid catabolic process, and immune-related processes such as CD4+ T cell proliferation and antigen presentation, reflecting stress adaptation and inflammation-induced reprogramming. Attenuation of DNA replication and ribosome biogenesis further underscores compromised cell cycle progression and epithelial maturation in CD. These findings highlight the profound effects of inflammation and metabolic dysregulation on epithelial differentiation, contributing to barrier dysfunction and disease progression. BEAM analyses of the rest branch points are provided in the Supplementary Figs S75–S78 [45].

Trajectory inference as a downstream analysis of cell annotation methods can reveal key points of divergence between disease and normal datasets, highlighting disrupted differentiation trajectories and altered branching nodes in disease, providing insights into the underlying mechanisms of disease progression and the impact of inflammation on cell development.

## Conclusion

In this study, we developed scSuperAnnotator, a one-stop system for scRNA-seq cell-type identification. scSuperAnnotator integrates marker-based and reference-based methods and provides downstream analyses at both gene and cell levels. Its interface supports in-depth analyses and method comparisons without programming, offering practical guidance for selecting approaches suited to a given dataset. In addition, scSuperAnnotator includes a rich catalog of built-in datasets. The dataset collection is actively expanded and spans multiple sequencing platforms, diverse human and mouse tissues, pathology cohorts for cross-disease evaluation, and custom sets for assessing marker-based annotation under different marker resources. All datasets are standardized with structured provenance, making the platform more comprehensive and robust.

We assembled six complementary benchmark suites reflecting common analysis settings and reported method rankings for each, spanning twelve same-platform pairs, nine cross-platform pairs, four cross-species transfers, ten pathology cohorts (Supplementary Note S9, Supplementary Figs S44–S53, and Supplementary Tables S12 and  S13), six marker-based benchmarks, and four unknown-class stress tests. To strengthen the platform’s coverage of upstream clustering paradigms that impact downstream annotation quality, we additionally conducted an in-depth comparison of two representative clustering methods (Supplementary Note S10, Supplementary Figs S54–S63, and Supplementary Table S14).

Within a single platform, methods are stratified into stable tiers—leaders with high metrics and consistent embeddings, a middle tier with increasing dataset dependence, and a trailing tier with recurrent shortcomings—indicating that performance differences are driven by method-intrinsic properties rather than dataset idiosyncrasies; for this setting, we recommend CaSTLe, singleCellNet, and ACTINN, with scPred/SingleR as alternatives and lower-ranked methods used cautiously. Under platform/center shifts, separation among methods reflects robustness to transfer rather than peak scores on individual datasets: robust approaches retain high means and low variance with limited pair-specific drops; SingleR is the default choice, with singleCellNet and scPred as strong alternatives, while ACTINN and scDeepSort require caution when reference–query divergence is large and nearest-neighbor or hierarchical schemes (scmapcell/scmapcluster, CHETAH, scClassify) are best avoided unless the reference tightly matches and coverage is verified. In cross-species scenarios, discrimination hinges on preserving true boundaries while maintaining intra-cluster coherence; singleCellNet is preferred for mouse-to-human transfer, with scmapcell and CaSTLe viable when moderate abstention is acceptable.

Across pathology cohorts, rankings were consistent: ACTINN was the strongest overall, followed by scPred, CaSTLe, and singleCellNet; SingleR and scClassify were intermediate; scDeepSort ranked lower, and nearest-neighbor/hierarchical methods were less robust to disease heterogeneity. Marker-based benchmarking showed that marker-set choice is the primary driver across datasets and methods, no single database dominates, and the integrated Cell Marker Accordion yields more stable maps and reduced between-dataset variability; within datasets, marker selection explains differences more than the annotator. In unknown class tests, robustness reflected an open-set strategy: strong methods maintained aggregate stability, abstained rather than forced mismatches, and preserved global organization; when relevant classes may be absent, prioritize scClassify for high unknown recall, use scPred for high-precision unknown recall, and prefer SingleR, singleCellNet, or ACTINN when minimizing abstention is required, acknowledging their tendency to reassign missing classes.

Further, we demonstrate the value of integrating cell-level and gene-level analyses following cell type identification to uncover disease-associated signals and provide valuable biological insights. Cell-level analyses reveal disrupted communication networks, altered interactions, and differentiation trajectories, highlighting the impact of inflammation and disease progression on cellular dynamics. Gene-level analyses identify changes in pathway activities and molecular signaling, offering insights into the underlying mechanisms of disease pathophysiology. Trajectory inference further pinpoints critical divergence points between disease and normal states, shedding light on disrupted differentiation and developmental processes. Together, these approaches underscore the potential of cell annotation methods to advance our understanding of disease mechanisms and facilitate the discovery of therapeutic targets.

Finally, it should be noted that the comparison results and corresponding conclusions in this study are based on sample datasets and example methods, and may not be fully applicable to all situations. Those who wish to further explore the performance of cell-type identification methods in different datasets can use our scSuperAnnotator to conduct experiments.

In future work, we will expand scSuperAnnotator’s method library and data resources beyond scRNA-seq by adding multi-omics capabilities such as scATAC-seq and cross-modal annotation, together with curated references for scATAC-seq and other single-cell modalities. These enhancements are intended to improve practical utility for researchers handling increasingly complex datasets and to ensure scSuperAnnotator keeps pace with rapid advances in single-cell analysis. In parallel, we will continue to strengthen downstream analysis to deliver more actionable insights. Performance optimization will remain a core priority: we will pursue software-level improvements and targeted hardware upgrades to further improve speed and scalability, making scSuperAnnotator a more powerful and broadly usable tool.

## Supplementary Material

gkaf1470_Supplemental_File

## Data Availability

As an online platform, scSuperAnnotator can be freely accessed without registration via https://sciden.aibio-lab.com/scIDEN/index/.
