# Correction to ‘scSuperAnnotator: A platform for benchmarking comparison and visualizing automated cellular annotation methods for scRNA-seq data’

**DOI:** 10.1093/nar/gkag684

**Published:** 2026-07-02

This is a correction to: Qi Qi, Yanchi Su, Yi Fan, Zhuohan Yu, Yujian Huang, Ka-Chun Wong, Xiangtao Li, scSuperAnnotator: a platform for benchmarking comparison and visualizing automated cellular annotation methods for scRNA-seq data, *Nucleic Acids Research*, Volume 54, Issue 1, 13 January 2026, gkaf1470, https://doi.org/10.1093/nar/gkaf1470

In the originally published version of this manuscript, an incorrect hyperlink was published in the Data Availability section. The correct link is https://sciden.aibio-lab.com/scIDEN/index/.

This error has been corrected.

**Addendum** 14 September 2026: in addition to the correction above, the following three references were omitted from the paper, and have been inserted as ^46, 47^, and ^48^ within the body of the text, with the full citations placed at the end of the originally-published **References** list.

46. Lieberman Y, Rokach L, Shay T. CaSTLe–classification of single cells by transfer learning: harnessing the power of publicly available single cell RNA sequencing experiments to annotate new experiments. PloS one, 2018, 13(10): e0205499.

47. Shao X, Yang H, Zhuang X, et al. scDeepSort: a pre-trained cell-type annotation method for single-cell transcriptomics using deep learning with a weighted graph neural network. Nucleic acids research, 2021, 49(21): e122-e122.

48. Lin Y, Cao Y, Kim H J, et al. scClassify: sample size estimation and multiscale classification of cells using single and multiple reference. Molecular systems biology, 2020, 16(6): MSB199389.

