# Supplementary material for: scSuperAnnotator: a platform for benchmarking comparison and visualizing automated cellular annotation methods for scRNA-seq data
Source: Nucleic Acids Res. 2026 Jan 6;54(1):gkaf1470. doi: 10.1093/nar/gkaf1470 (PMC12774656; doi:10.1093/nar/gkaf1470)
Supplement: gkaf1470_Supplemental_File [file gkaf1470_supplemental_file.pdf]

Supplementary Material to "scSuperAnnotator: a platform for benchmarking comparison and visualizing automated cellular annotation methods for scRNA-seq data"

Contents

1 Supplementary Note 1: Summary of Methods 2

2 Supplementary Note 2: Supplementary for "Data Input Module" 3

3 Supplementary Note 3: Data Upload Requirements 4

4 Supplementary Note 4: Supplementary for "Benchmarking Annotation Methods in Same-Platform Scenarios" 5

5 Supplementary Note 5: Supplementary for "Benchmarking Annotation Methods in Cross-Platform Scenarios" 13

6 Supplementary Note 6: Supplementary for "Benchmarking Annotation Methods in Cross-Species Scenarios" 19

7 Supplementary Note 7: Supplementary for "Benchmarking Annotation Methods for Unknown Cell Type Detection" 24

8 Supplementary Note 8: Supplementary for "Benchmarking Annotation Methods Across Marker Database" 29

9 Supplementary Note 9: Benchmarking Annotation Methods on Pathology Datasets 45

10 Supplementary Note 10: Benchmarking of scTPC and scDFN with Cell Population Identification Methods 54

11 Supplementary Note 11: Supplementary for "Identification of potential disease-associated cell populations" 66

12 Supplementary Note 12: Supplementary for "Deciphering disease biology through cell signaling and trajectory analysis" 76

13 Supplementary Note 13: Example Outputs from the Web Server 80

14 Supplementary Note 14: Web Server Overview and Screenshot 82

1 Supplementary Note 1: Summary of Methods

Supplementary Table S1. Summary of methods

Methods	Algorithm	Prior knowledge	Rejection	Language	Reference
SingleCellNet	Random forest	Reference data	yes	R	[1]
scPred	SVM	Reference data	yes	R	[2]
ACTINN	Neural network	Reference data	yes	Python	[3]
CaSTLe	XGBoost	Reference data	yes	R	[4]
scClassify	Ensemble learning and cell type hierarchical tree	Reference data	yes	R	[5]
scDeepSort	GNN	Reference data	yes	Python	[6]
scTPC	semisupervised deep clustering			Python	[7]
scDFN	Deep fusion networks			Python	[8]
scmap-cluster	Cluster-level similarity matching	Reference data	yes	R	[9]
scmap-cell	Cell-level nearest neighbor voting	Reference data	yes	R	[9]
SingleR	Spearman coefficient	Reference data	yes	R	[10]
CHETAH	Spearman correlation based classification tree	Reference data	yes	R	[11]
scCATCH	Evidence-based scoring	Marker genes data		R	[12]
SCINA	EM	Marker genes data	yes	R	[13]
CellAssign	Hierarchical statistical framework	Marker genes data	yes	R	[14]
Garnett	hierarchical classification	Marker genes data	yes	R	[15]

2 Supplementary Note 2: Supplementary for "Data Input Module"

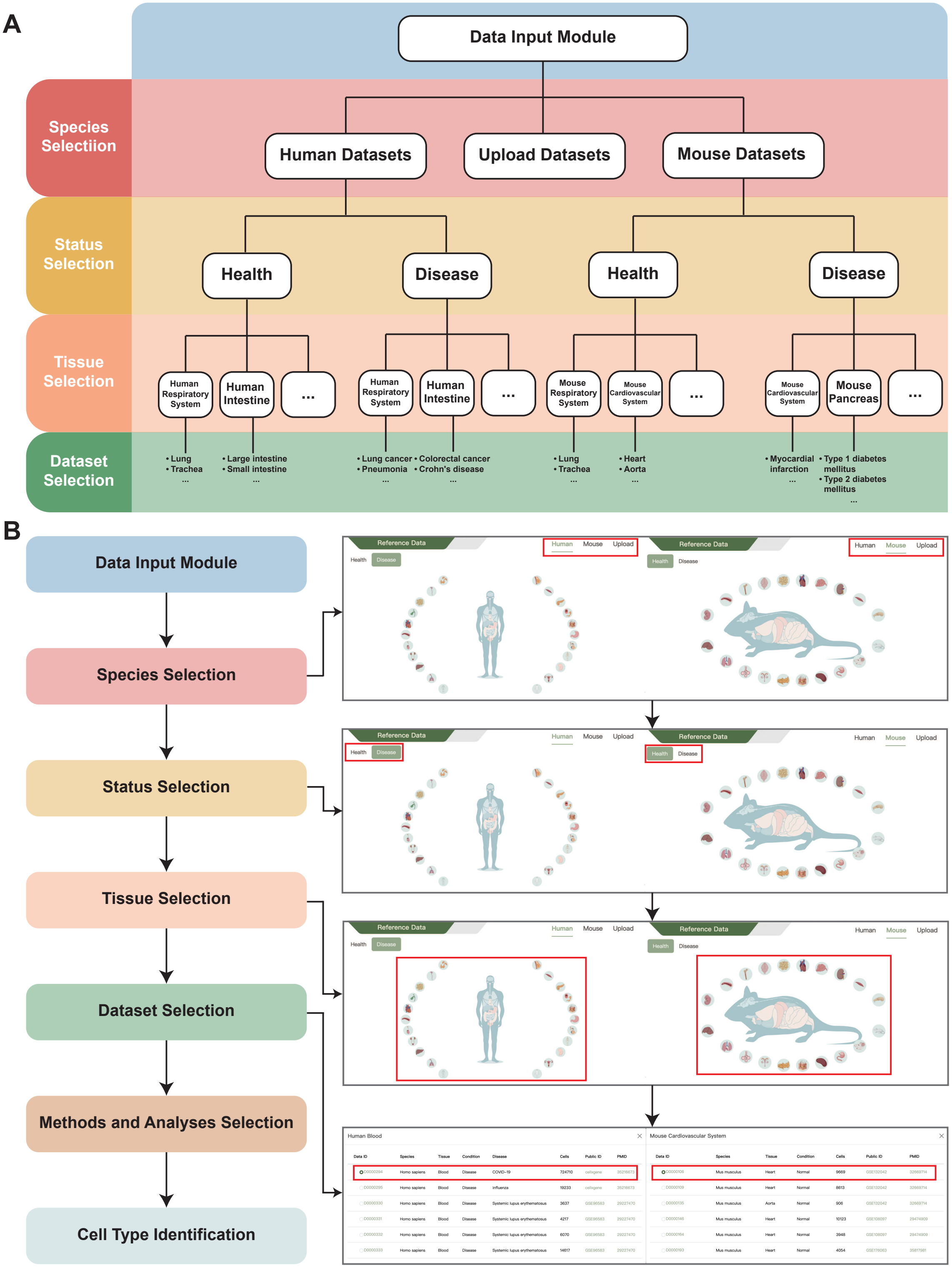

Supplementary Figure S1. (**A**) Schematic overview of the hierarchical data organization in Data Input module. (**B**) The correspondence between the hierarchical structure of Data Input module and its operational interface.

3 Supplementary Note 3: Data Upload Requirements

In addition to providing a comprehensive reference database, scSuperAnnotator also allows users to upload their own datasets as either references or queries. Under the Upload tab, users can submit data by clicking on the designated upload area (Supplementary Fig. S2). Currently, scSuperAnnotator supports only the *.h5ad* format. Each uploaded file must meet the following conditions:

- The AnnData object's *.X* attribute must be a sparse matrix where rows correspond to cells (observations) and columns correspond to genes (variables).
- For any dataset used in annotation performance evaluation, the *.obs* attribute must include a *cell_type* column. To ensure semantic interoperability, it is recommended that all cell type labels follow standardized terminology defined by the Cell Ontology.

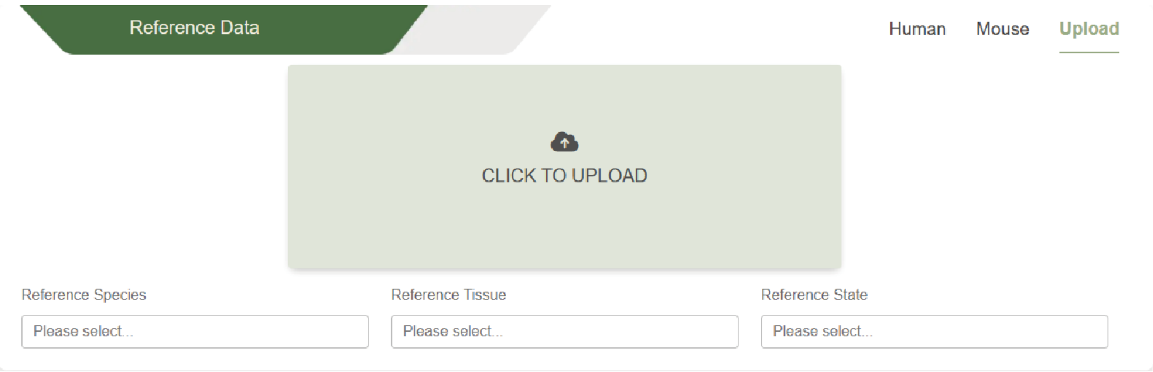

Supplementary Figure S2. The file upload interface.

4 Supplementary Note 4: Supplementary for "Benchmarking Annotation Methods in Same-Platform Scenarios"

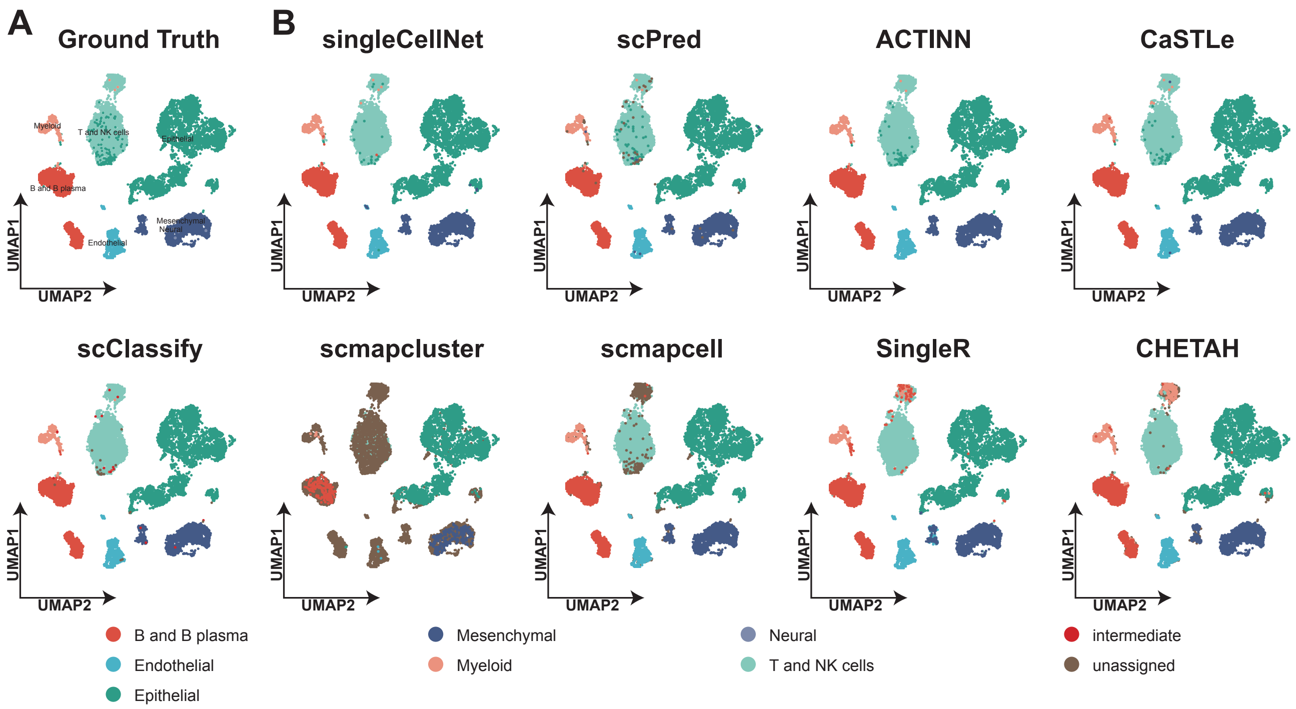

Supplementary Figure S3. (**A**) Ground Truth UMAP for the 10x 1 query dataset. (**B**) UMAP comparison of method-specific annotations on the 10x 1 query dataset.

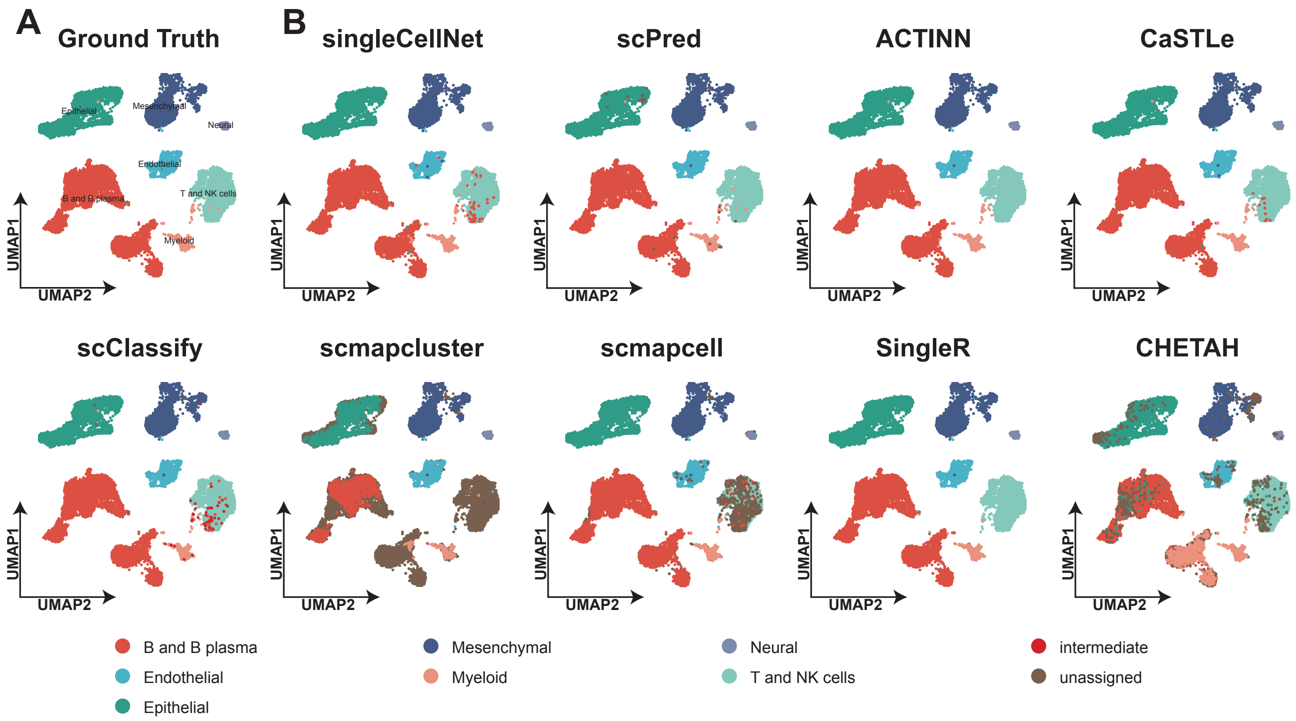

Supplementary Figure S4. (**A**) Ground Truth UMAP for the 10x 2 query dataset. (**B**) UMAP comparison of method-specific annotations on the 10x 2 query dataset.

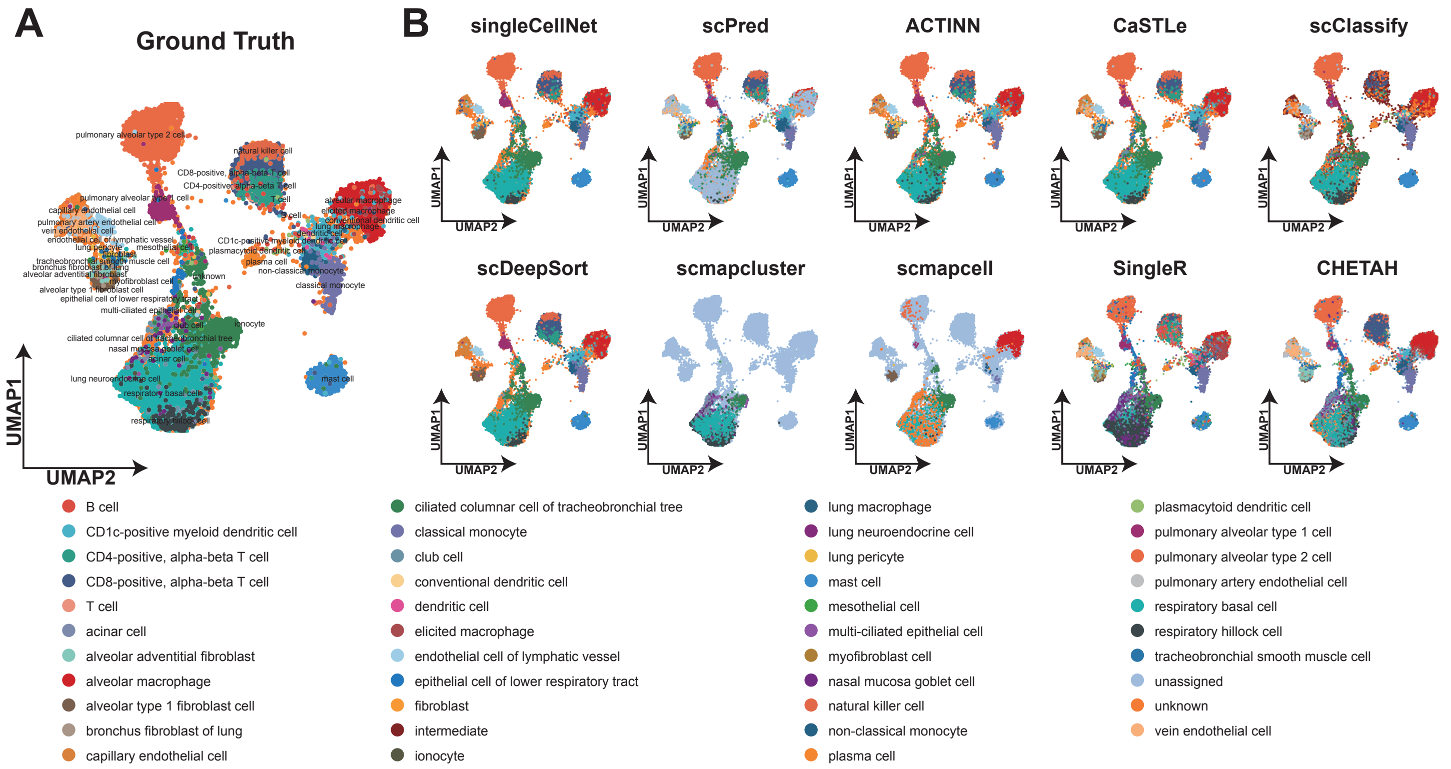

Supplementary Figure S5. (**A**) Ground Truth UMAP for the Drop-seq 1 query dataset. (**B**) UMAP comparison of method-specific annotations on the Drop-seq 1 query dataset.

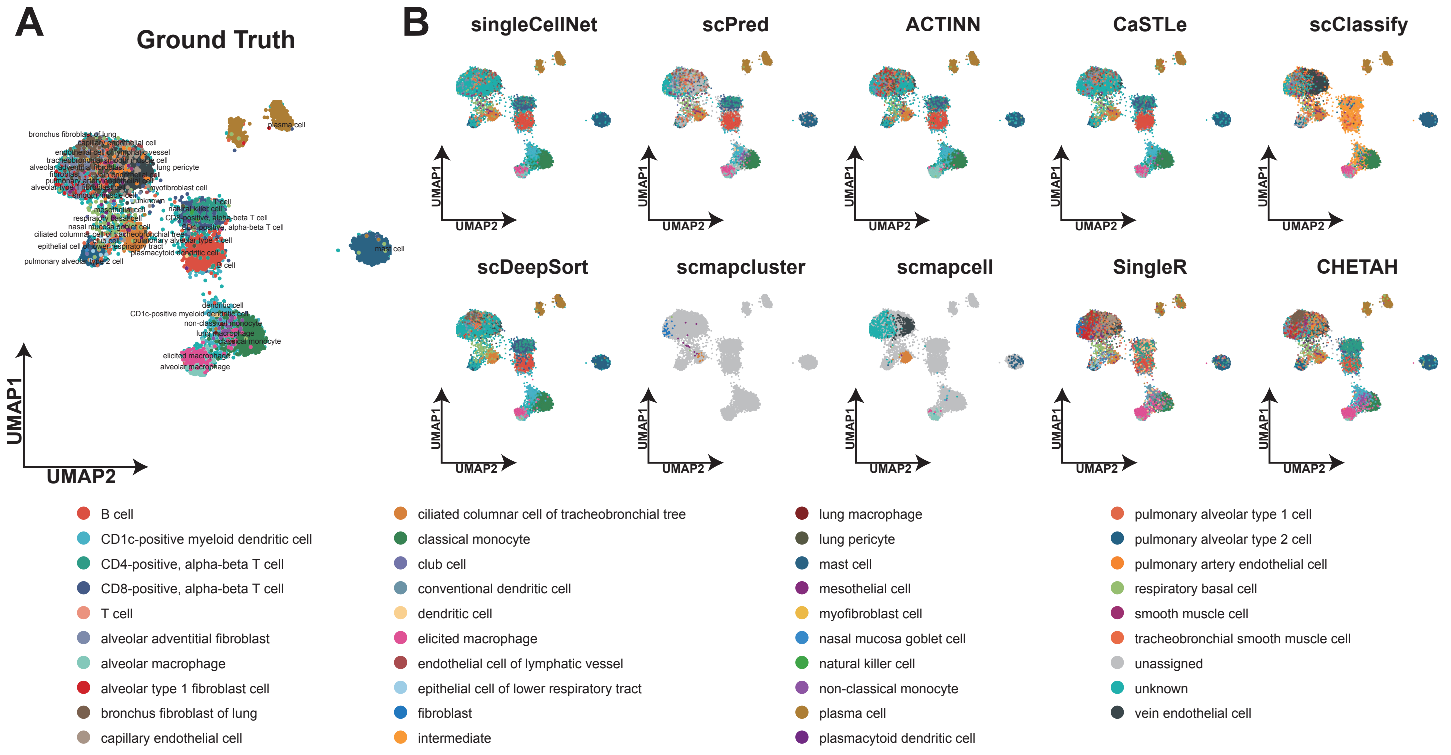

Supplementary Figure S6. (**A**) Ground Truth UMAP for the Drop-seq 2 query dataset. (**B**) UMAP comparison of method-specific annotations on the Drop-seq 2 query dataset.

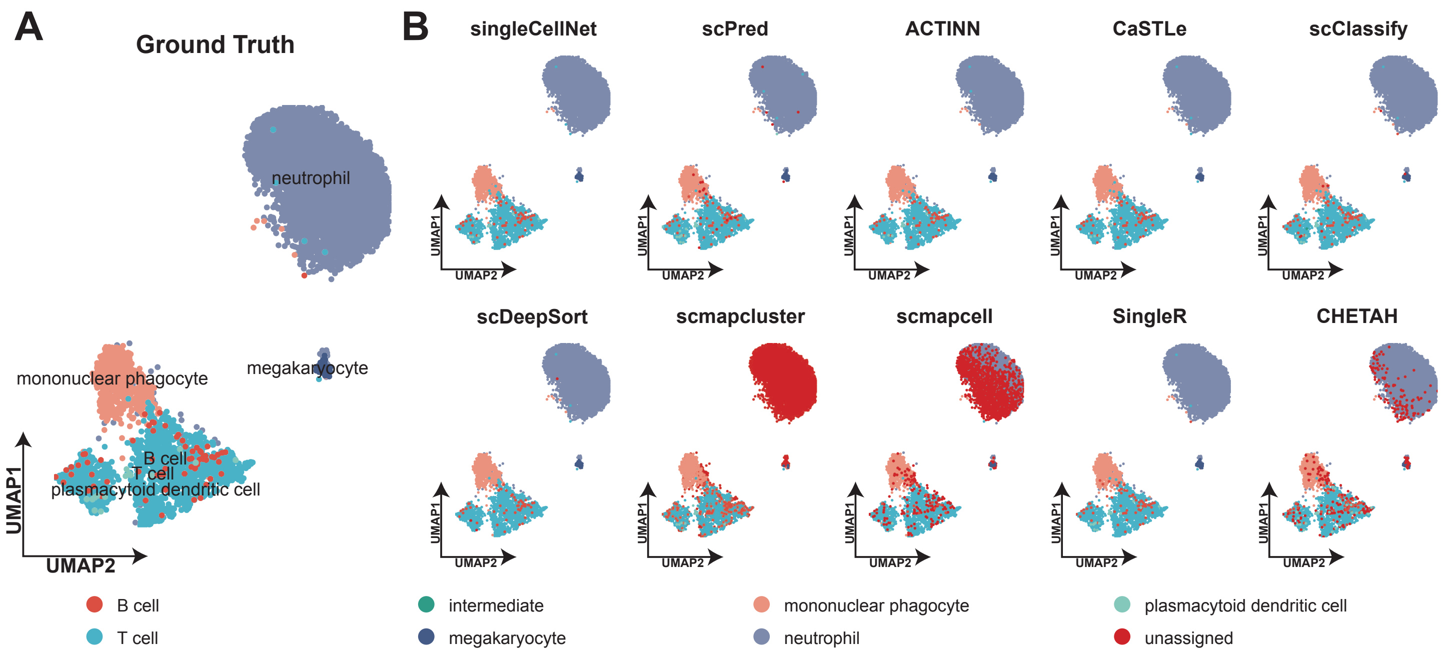

Supplementary Figure S7. (**A**) Ground Truth UMAP for the inDrop 1 query dataset. (**B**) UMAP comparison of method-specific annotations on the inDrop 1 query dataset.

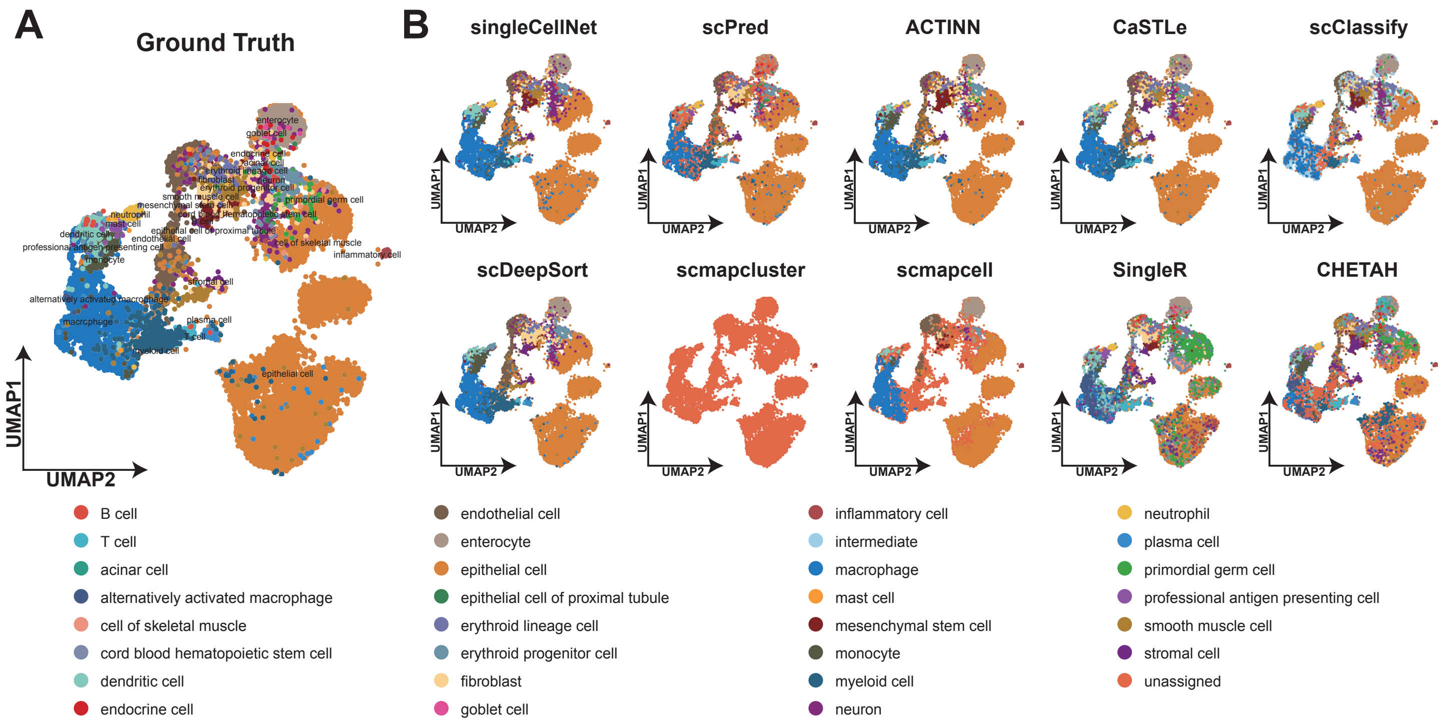

Supplementary Figure S8. (**A**) Ground Truth UMAP for the microwell-seq 1 query dataset. (**B**) UMAP comparison of method-specific annotations on the microwell-seq 1 query dataset.

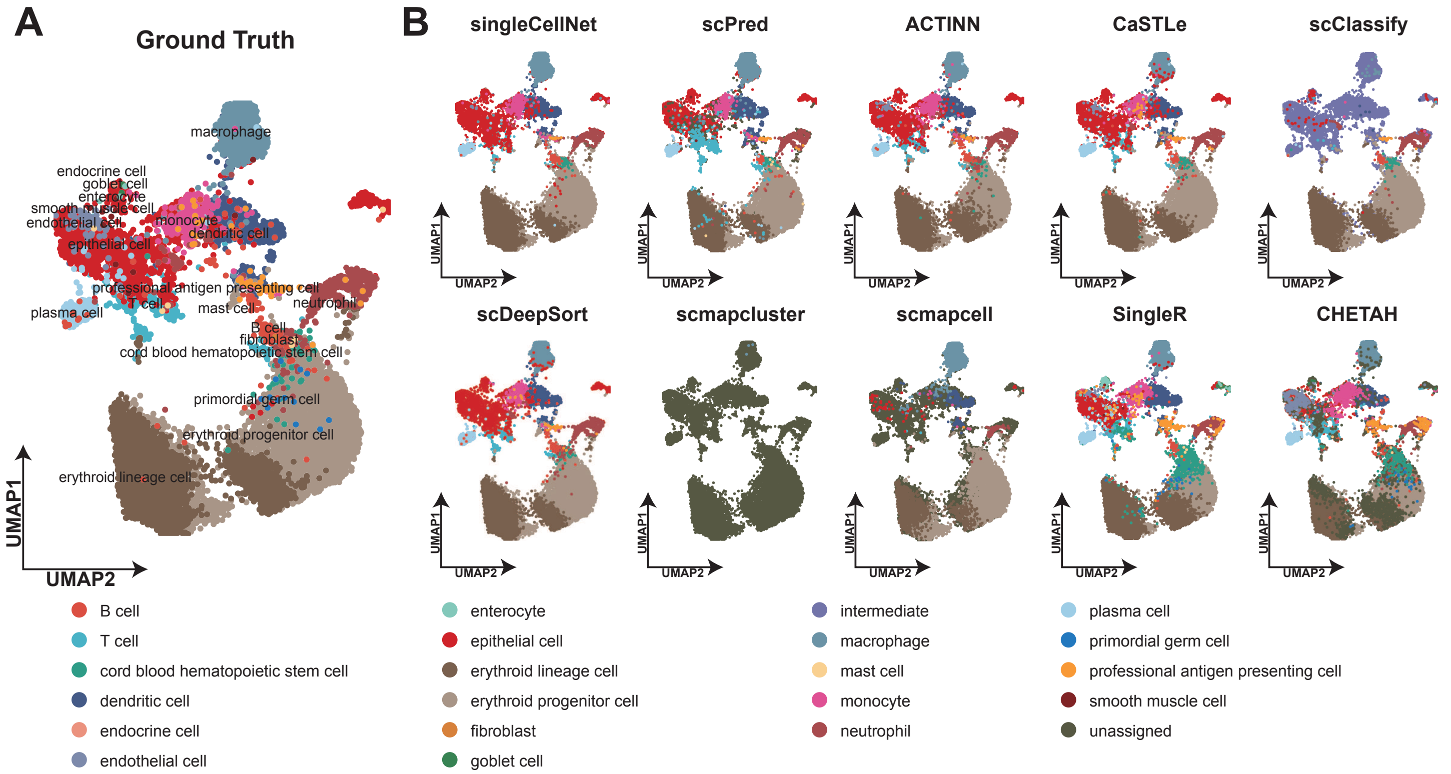

Supplementary Figure S9. (**A**) Ground Truth UMAP for the microwell-seq 2 query dataset. (**B**) UMAP comparison of method-specific annotations on the microwell-seq 2 query dataset.

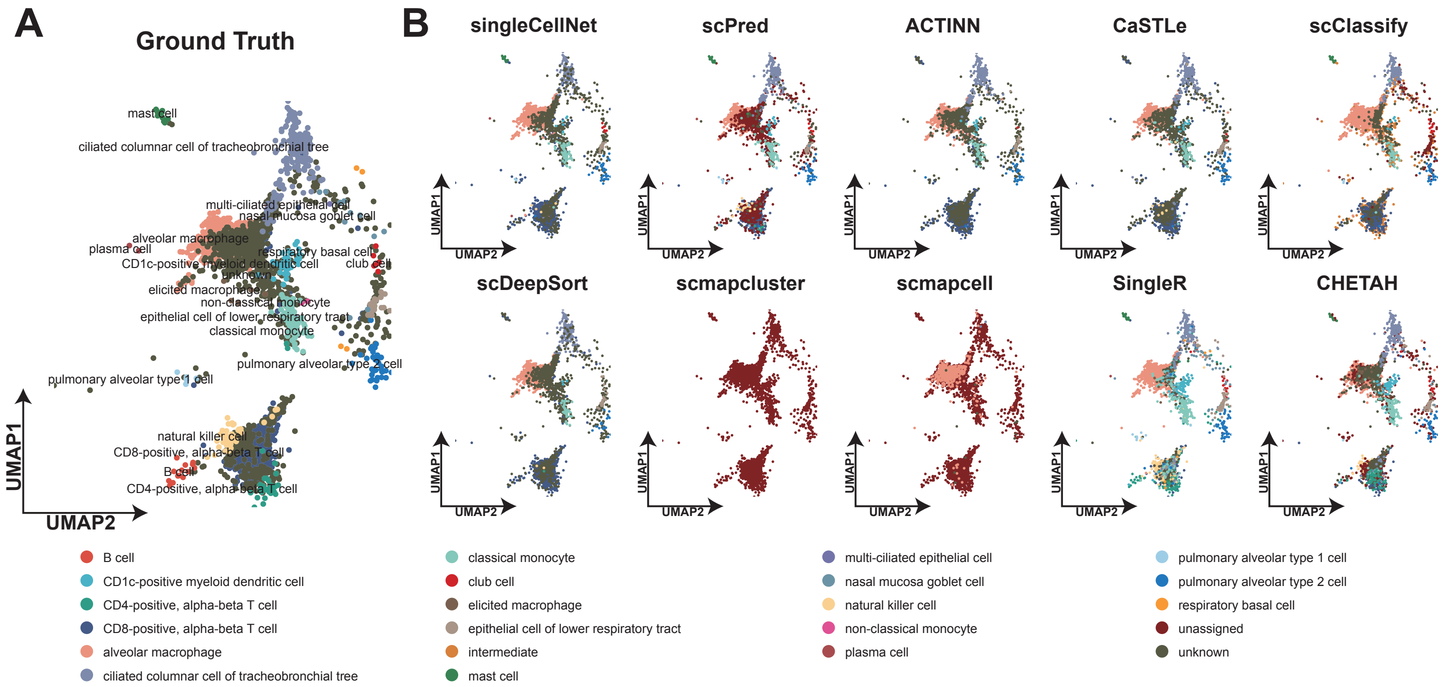

Supplementary Figure S10. (**A**) Ground Truth UMAP for the Seq-Well 1 query dataset. (**B**) UMAP comparison of method-specific annotations on the Seq-Well 1 query dataset.

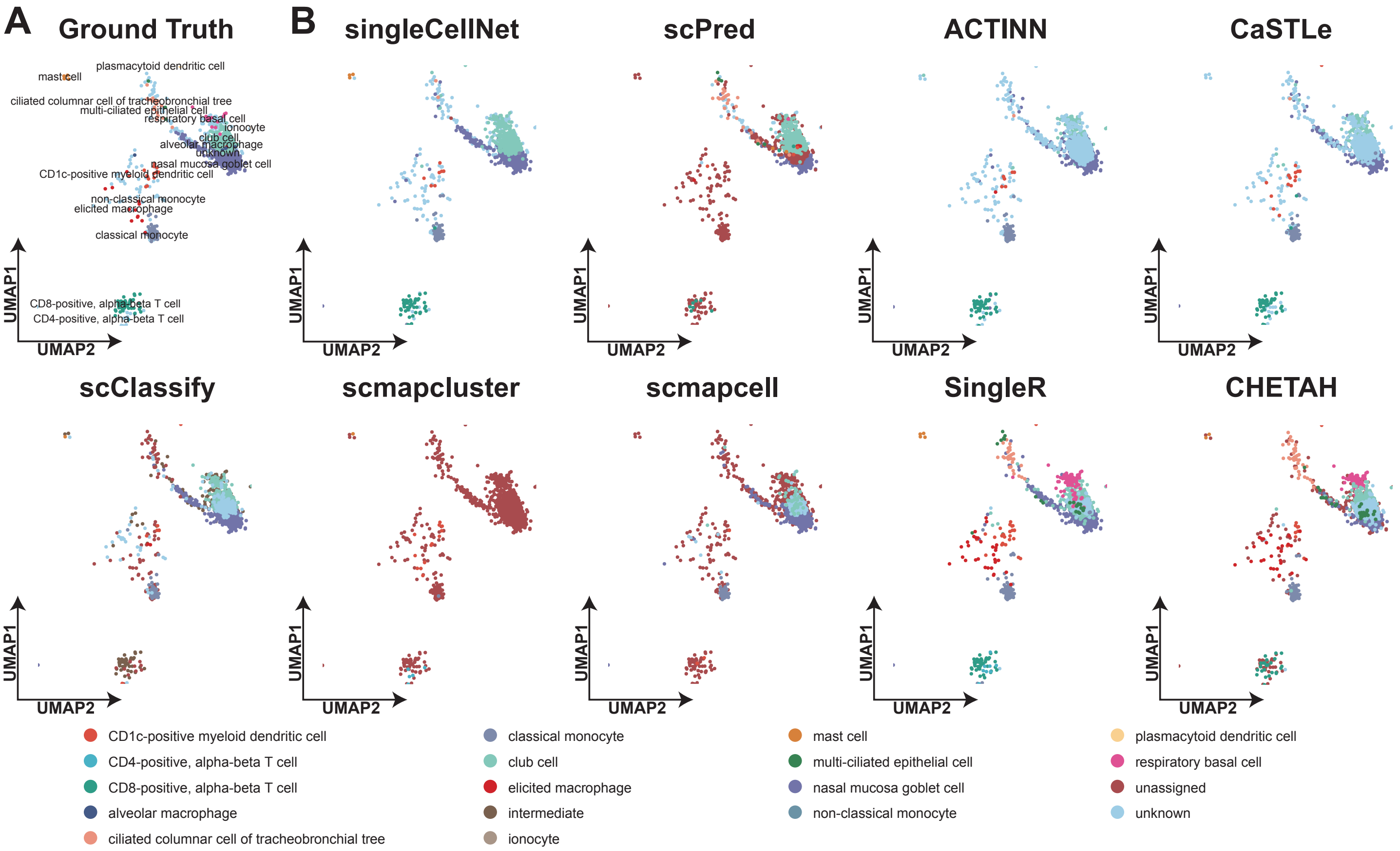

Supplementary Figure S11. (**A**) Ground Truth UMAP for the Seq-Well 2 query dataset. (**B**) UMAP comparison of method-specific annotations on the Seq-Well 2 query dataset.

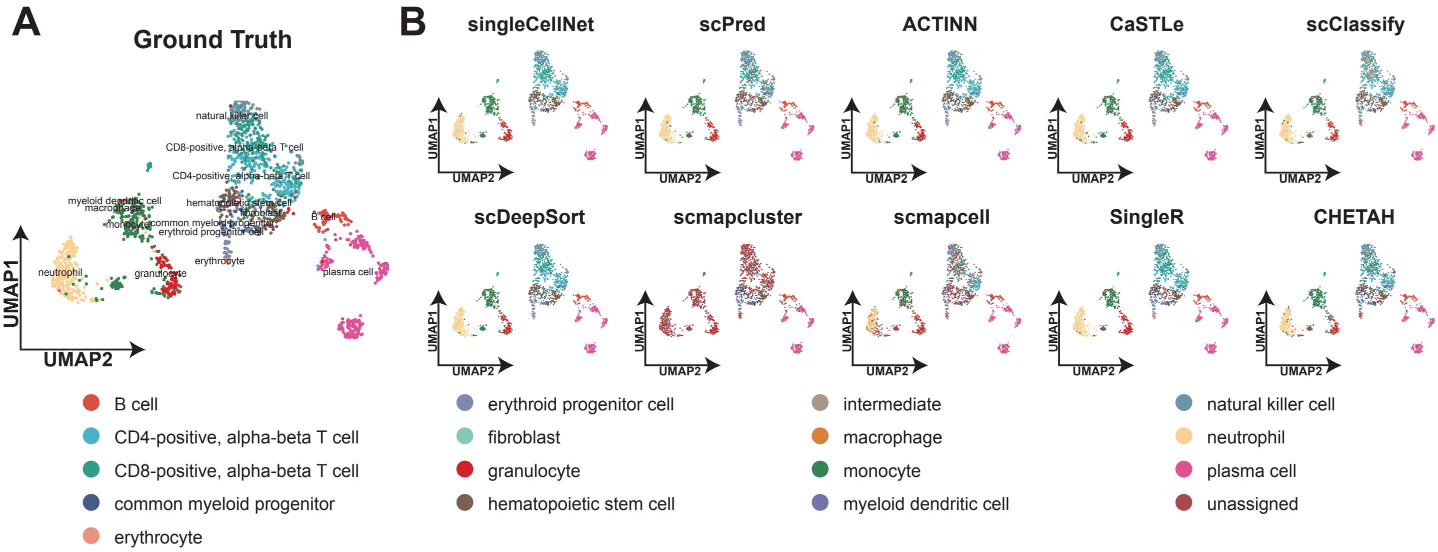

Supplementary Figure S12. (**A**) Ground Truth UMAP for the Smart-seq 1 query dataset. (**B**) UMAP comparison of method-specific annotations on the Smart-seq 1 query dataset.

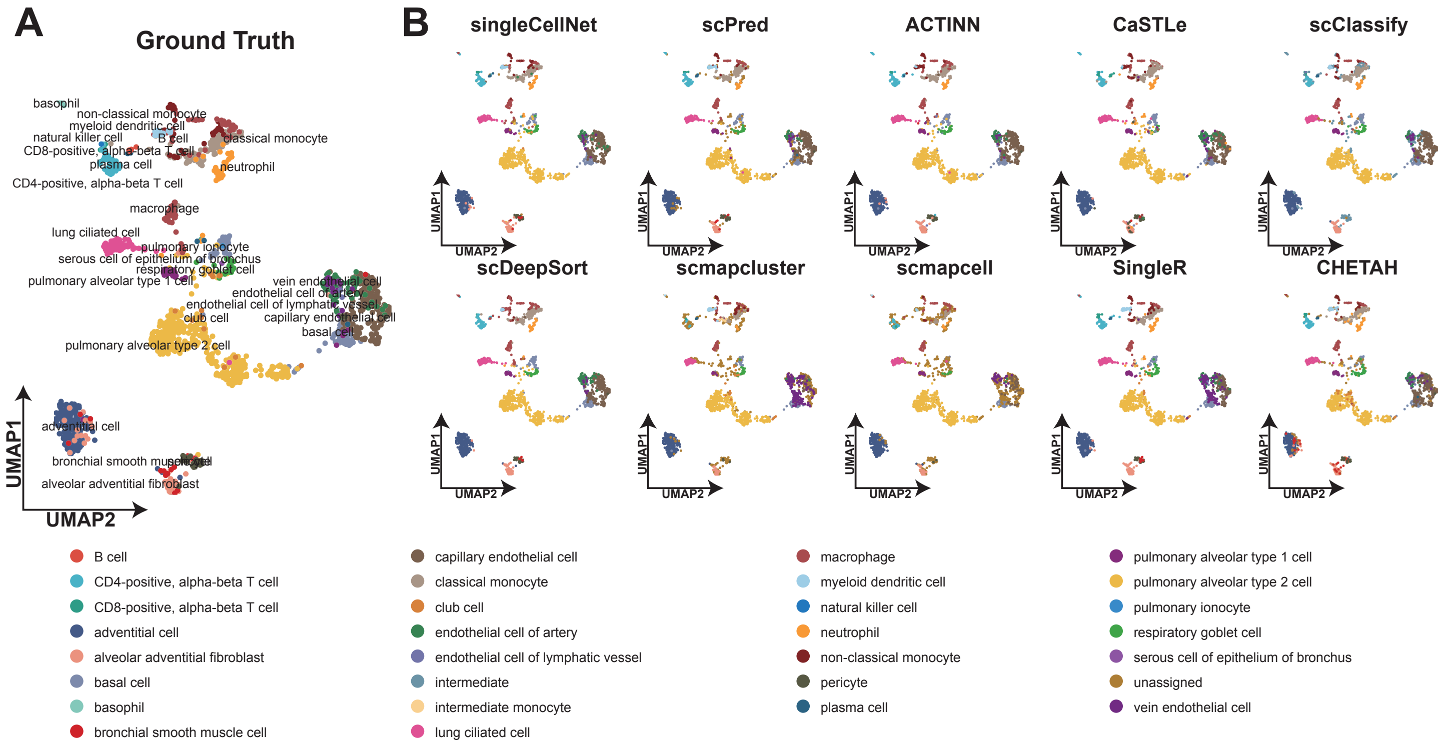

Supplementary Figure S13. (**A**) Ground Truth UMAP for the Smart-seq 2 query dataset. (**B**) UMAP comparison of method-specific annotations on the Smart-seq 2 query dataset.

Supplementary Table S2. Datasets for Same Platform Benchmarking

Datasets	#celltype	#cell	#gene	Tissue	Species	Condition	Protocol	Reference
10x 1(ref)	7	24094	18442	salivary gland	human	health	10x	[16]
10x 1(query)	7	12047	18442	salivary gland	human	health	10x	[16]
10x 2(ref)	7	17945	18442	large intestine	human	disease	10x	[16]
10x 2(query)	7	17946	18442	large intestine	human	disease	10x	[16]
Drop-seq 1(ref)	45	15262	56239	lung	human	health	Drop-seq	[17]
Drop-seq 1(query)	41	15263	56239	lung	human	health	Drop-seq	[17]
Drop-seq 2(ref)	38	8763	56239	lung	human	disease	Drop-seq	[17]
Drop-seq 2(query)	36	8763	56239	lung	human	disease	Drop-seq	[17]
inDrop 1(ref)	10	16446	45454	lung	human	disease	inDrop	[18]
inDrop 1(query)	10	16447	45454	lung	human	disease	inDrop	[18]
inDrop 2(ref)	7	6801	45454	lung	human	health	inDrop	[18]
inDrop 2(query)	6	6802	45454	lung	human	health	inDrop	[18]
microwell-seq 1(ref)	33	21738	26405	adrenal gland	human	health	microwell-seq	[19]
microwell-seq 1(query)	29	21738	26405	adrenal gland	human	health	microwell-seq	[19]
microwell-seq 2(ref)	20	14250	26405	liver	human	health	microwell-seq	[19]
microwell-seq 2(query)	20	14251	26405	liver	human	health	microwell-seq	[19]
Seq-Well 1(ref)	23	4008	56239	lung	human	health	Seq-Well	[17]
Seq-Well 1(query)	20	4008	56239	lung	human	health	Seq-Well	[17]
Seq-Well 2(ref)	17	3801	56239	nose	human	health	Seq-Well	[17]
Seq-Well 2(query)	16	3802	56239	nose	human	health	Seq-Well	[17]
Smart-seq2 1(ref)	13	1565	61759	bone marrow	human	health	Smart-seq2	[20]
Smart-seq2 1(query)	15	1566	61759	bone marrow	human	health	Smart-seq2	[20]
Smart-seq2 2(ref)	30	1446	61759	lung	human	health	Smart-seq2	[20]
Smart-seq2 2(query)	27	1447	61759	lung	human	health	Smart-seq2	[20]

Supplementary Table S3. Method Performance on Same-Platform Benchmarks

		10x 1	10x 2	Drop_seq 1	Drop_seq 2	inDrop 1	inDrop 2	microwell_seq 1	microwell_seq 2	Seq_Well 1	Seq_Well 2	Smart_seq2 1	Smart_seq2 2
Acc	singleCellNet	0.9836	0.9943	0.7352	0.7477	0.9553	0.9922	0.8913	0.8613	0.6707	0.6887	0.8723	0.8362
	scPred	0.9896	0.9981	0.6404	0.6435	0.9613	0.9930	0.9143	0.8190	0.4742	0.5554	0.7905	0.8196
	ACTINN	0.9905	0.9993	0.6976	0.6961	0.9656	0.9943	0.9260	0.8527	0.6191	0.6500	0.8946	0.8300
	CaSTLe	0.9853	0.9974	0.7241	0.7366	0.9668	0.9943	0.9206	0.8754	0.6612	0.6664	0.8748	0.8100
	scClassify	0.9833	0.9957	0.4259	0.3728	0.9432	0.9961	0.7475	0.4771	0.4587	0.4115	0.7963	0.7554
	scDeepSort	0.0000	0.0000	0.7500	0.7282	0.9720	0.9946	0.9319	0.8983	0.0000	0.6967	0.9036	0.8459
	scampcluster	0.4795	0.3505	0.0501	0.0025	0.1591	0.2563	0.0005	0.0002	0.0024	0.0000	0.2478	0.5473
	scmapcell	0.9202	0.9028	0.1247	0.0993	0.6138	0.7573	0.3937	0.3835	0.2341	0.0987	0.4642	0.5301
	SingleR	0.9576	0.9923	0.5898	0.5177	0.9045	0.9963	0.6908	0.7255	0.6236	0.5287	0.8225	0.8314
	CHETAH	0.9175	0.8793	0.4385	0.5332	0.7888	0.9569	0.7491	0.7079	0.4116	0.5046	0.7886	0.7547
F1-score	singleCellNet	0.9836	0.9943	0.7352	0.7477	0.9553	0.9922	0.8913	0.8613	0.6707	0.6887	0.8723	0.8362
	scPred	0.9896	0.9981	0.6404	0.6435	0.9613	0.9930	0.9143	0.8190	0.4742	0.5554	0.7905	0.8196
	ACTINN	0.9905	0.9993	0.6976	0.6961	0.9656	0.9943	0.9260	0.8527	0.6191	0.6500	0.8946	0.8300
	CaSTLe	0.9853	0.9974	0.7241	0.7366	0.9668	0.9943	0.9206	0.8754	0.6612	0.6664	0.8748	0.8100
	scClassify	0.9833	0.9957	0.4259	0.3728	0.9432	0.9961	0.7475	0.4771	0.4587	0.4115	0.7963	0.7554
	scDeepSort	0.0000	0.0000	0.7500	0.7282	0.9720	0.9946	0.9319	0.8983	0.0000	0.6967	0.9036	0.8459
	scampcluster	0.4795	0.3505	0.0501	0.0025	0.1591	0.2563	0.0005	0.0002	0.0024	0.0000	0.2478	0.5473
	scmapcell	0.9202	0.9028	0.1247	0.0993	0.6138	0.7573	0.3937	0.3835	0.2341	0.0987	0.4642	0.5301
	SingleR	0.9576	0.9923	0.5898	0.5177	0.9045	0.9963	0.6908	0.7255	0.6236	0.5287	0.8225	0.8314
	CHETAH	0.9175	0.8793	0.4385	0.5332	0.7888	0.9569	0.7491	0.7079	0.4116	0.5046	0.7886	0.7547
MCC	singleCellNet	0.9783	0.9922	0.6900	0.6858	0.9438	0.9850	0.8339	0.8276	0.5046	0.5830	0.8561	0.8156
	scPred	0.9862	0.9974	0.6270	0.6044	0.9517	0.9865	0.8742	0.7832	0.3792	0.5125	0.7762	0.8027
	ACTINN	0.9874	0.9991	0.6468	0.6250	0.9568	0.9890	0.8878	0.8174	0.4249	0.5223	0.8816	0.8087
	CaSTLe	0.9806	0.9965	0.6766	0.6726	0.9583	0.9890	0.8793	0.8452	0.4891	0.5469	0.8591	0.7857
	scClassify	0.9781	0.9942	0.4507	0.4504	0.9298	0.9925	0.6566	0.4647	0.3468	0.3658	0.7815	0.7399
	scDeepSort	0.0000	0.0000	0.7109	0.6672	0.9650	0.9896	0.8971	0.8740	0.0000	0.5873	0.8914	0.8265
	scampcluster	0.4800	0.4522	0.0792	0.0235	0.2784	0.4440	0.0186	0.0109	0.0279	0.0000	0.3312	0.5449
	scmapcell	0.8999	0.8780	0.2162	0.1809	0.6165	0.6887	0.3659	0.4001	0.2118	0.1436	0.5033	0.5416
	SingleR	0.9444	0.9894	0.5960	0.5449	0.8834	0.9928	0.6113	0.6861	0.4913	0.4527	0.8027	0.8135
	CHETAH	0.8949	0.8519	0.4298	0.5337	0.7598	0.9217	0.6602	0.6715	0.3210	0.4176	0.7678	0.7344
NMI	singleCellNet	0.9444	0.9759	0.6389	0.6176	0.8969	0.9508	0.7201	0.7283	0.3529	0.4590	0.7940	0.7994
	scPred	0.9668	0.9939	0.6349	0.5983	0.9333	0.9630	0.8109	0.6909	0.3026	0.4765	0.7388	0.7950
	ACTINN	0.9679	0.9966	0.6134	0.5660	0.9366	0.9622	0.8042	0.7246	0.2865	0.3941	0.8332	0.8084
	CaSTLe	0.9510	0.9877	0.6296	0.6015	0.9249	0.9663	0.7821	0.7462	0.3347	0.4062	0.7984	0.7728
	scClassify	0.9502	0.9860	0.4990	0.5531	0.9114	0.9766	0.6827	0.4844	0.3532	0.3599	0.7561	0.7418
	scDeepSort	0.0000	0.0000	0.6692	0.6148	0.9456	0.9736	0.8181	0.7888	0.0000	0.4569	0.8438	0.8227
	scampcluster	0.4976	0.5910	0.1672	0.0113	0.2775	0.6629	0.0033	0.0006	0.0260	0.0000	0.4484	0.6273
	scmapcell	0.8635	0.8686	0.1966	0.1524	0.6439	0.7014	0.3126	0.3772	0.1663	0.0909	0.5191	0.5863
	SingleR	0.8873	0.9734	0.6314	0.5815	0.8331	0.9747	0.5846	0.6538	0.3862	0.4087	0.7624	0.8140
	CHETAH	0.8412	0.8143	0.5423	0.5455	0.7556	0.8799	0.5774	0.6200	0.3250	0.3422	0.7171	0.7266
ARI	singleCellNet	0.9515	0.9856	0.4258	0.4304	0.9397	0.9820	0.8384	0.7378	0.3242	0.4179	0.7776	0.8180
	scPred	0.9736	0.9980	0.3864	0.3635	0.9720	0.9900	0.9255	0.7386	0.3047	0.4072	0.6866	0.8179
	ACTINN	0.9701	0.9988	0.3762	0.3548	0.9666	0.9862	0.9115	0.6958	0.2593	0.3478	0.8120	0.8257
	CaSTLe	0.9554	0.9939	0.4021	0.4117	0.9623	0.9898	0.8901	0.7742	0.3221	0.3705	0.7814	0.8147
	scClassify	0.9531	0.9932	0.2121	0.2812	0.9600	0.9928	0.8295	0.3631	0.3264	0.2501	0.7123	0.7358
	scDeepSort	0.0000	0.0000	0.4576	0.4041	0.9734	0.9935	0.9219	0.8098	0.0000	0.4207	0.8291	0.8411
	scampcluster	0.3337	0.3469	−0.0160	−0.0019	0.1084	0.7159	0.0009	0.0001	0.0068	0.0000	0.1451	0.4812
	scmapcell	0.8795	0.8768	0.0254	0.0071	0.4621	0.5865	0.0926	0.1547	0.0540	0.0285	0.1880	0.4004
	SingleR	0.9114	0.9827	0.3768	0.2083	0.8300	0.9925	0.5638	0.5648	0.3492	0.3273	0.7050	0.8253
	CHETAH	0.8768	0.7705	0.2619	0.2128	0.7655	0.9280	0.7057	0.5669	0.2108	0.2471	0.6638	0.7101

5 Supplementary Note 5: Supplementary for ”Benchmarking Annotation Methods in Cross-Platform Scenarios”

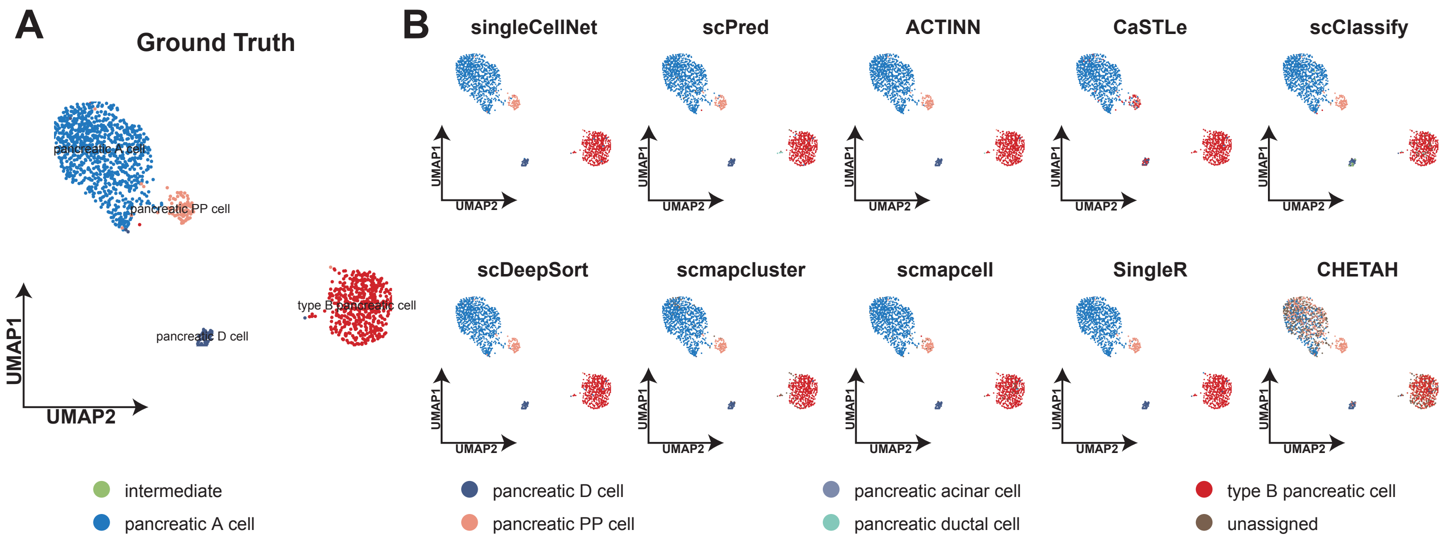

Supplementary Figure S14. (**A**) Ground truth UMAP of the Xin (SMARTer) query dataset. (**B**) UMAP of the Xin (SMARTer) query showing method-specific predicted cell type annotations using Muraro (CEL-seq2) as the reference.

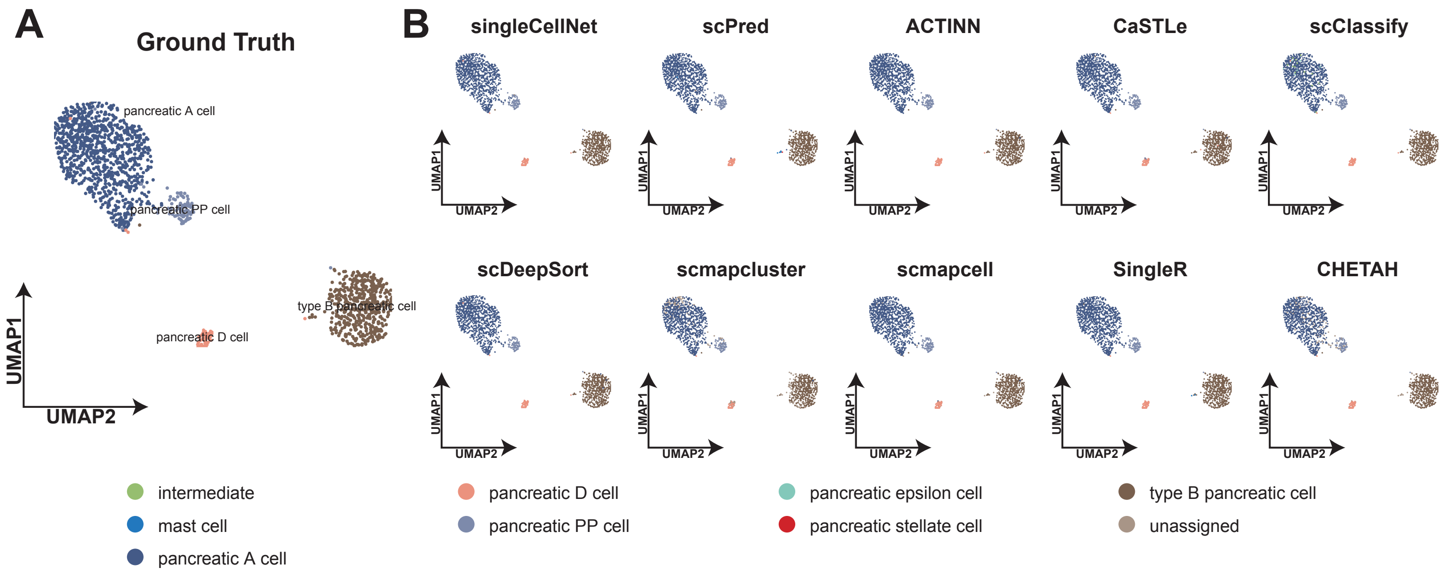

Supplementary Figure S15. (**A**) Ground truth UMAP of the Xin (SMARTer) query dataset. (**B**) UMAP of the Xin (SMARTer) query showing method-specific predicted cell type annotations using Segerstolpe (Smart-seq2) as the reference.

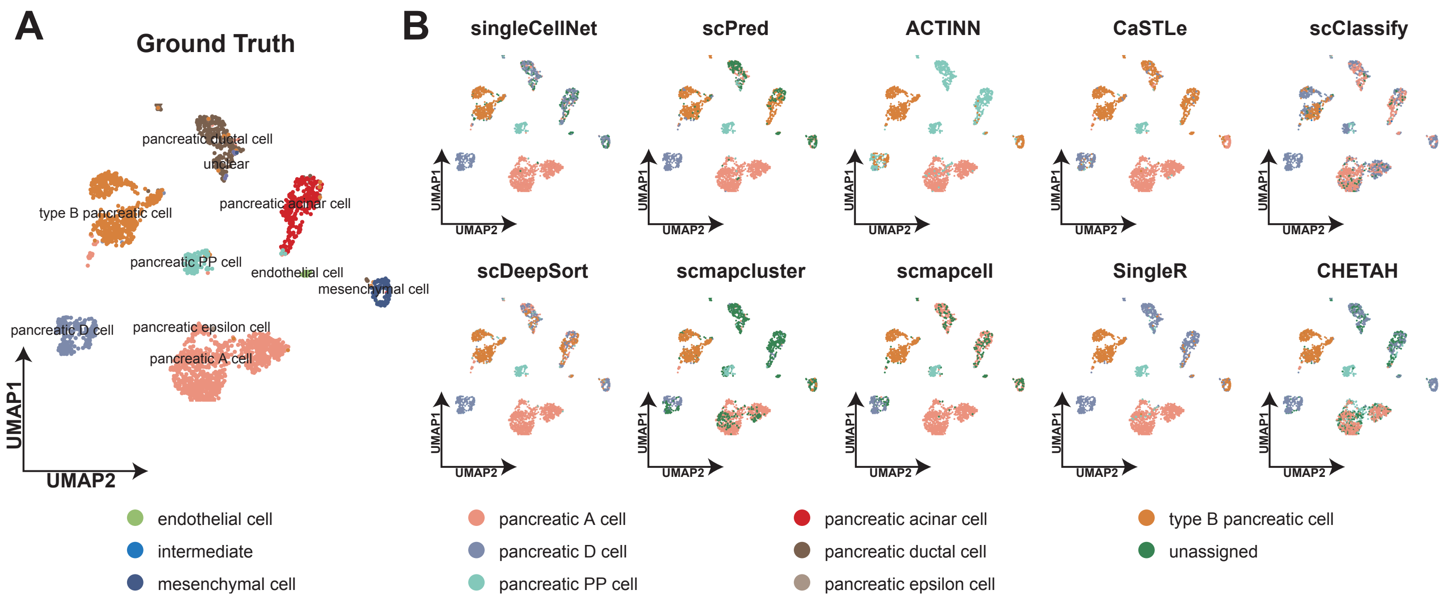

Supplementary Figure S16. (**A**) Ground truth UMAP of the Muraro (CEL-seq2) query dataset. (**B**) UMAP of the Muraro (CEL-seq2) query showing method-specific predicted cell type annotations using Xin (SMARTer) as the reference.

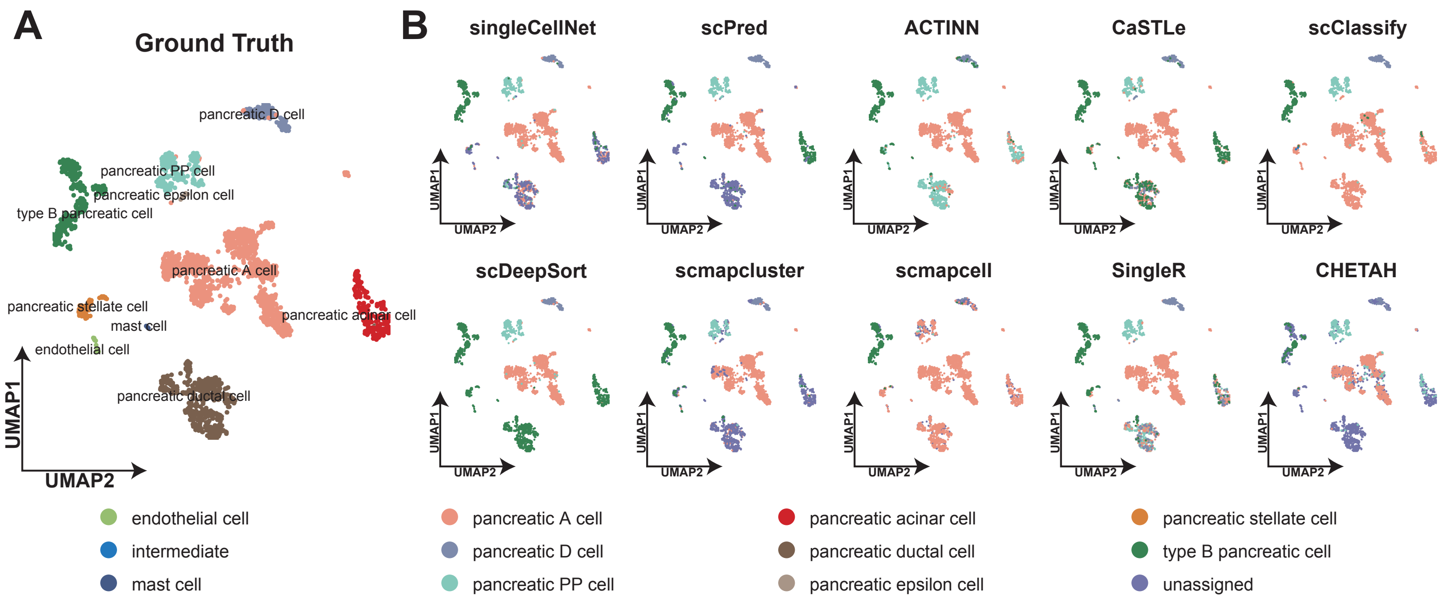

Supplementary Figure S17. (**A**) Ground truth UMAP of the Segerstolpe (Smart-seq2) query dataset. (**B**) UMAP of the Segerstolpe (Smart-seq2) query showing method-specific predicted cell type annotations using Xin (SMARTer) as the reference.

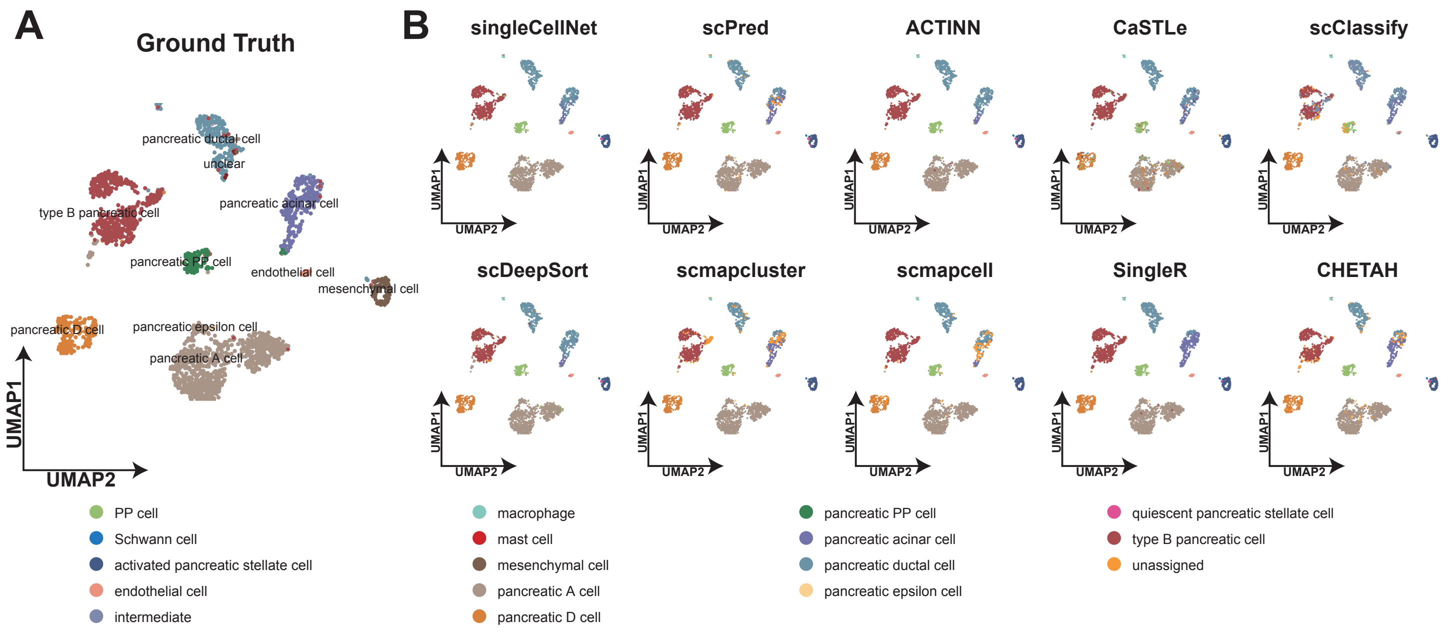

Supplementary Figure S18. (**A**) Ground truth UMAP of the Muraro (CEL-seq2) query dataset. (**B**) UMAP of the Muraro (CEL-seq2) query showing method-specific predicted cell type annotations using hBaron (inDrop) as the reference.

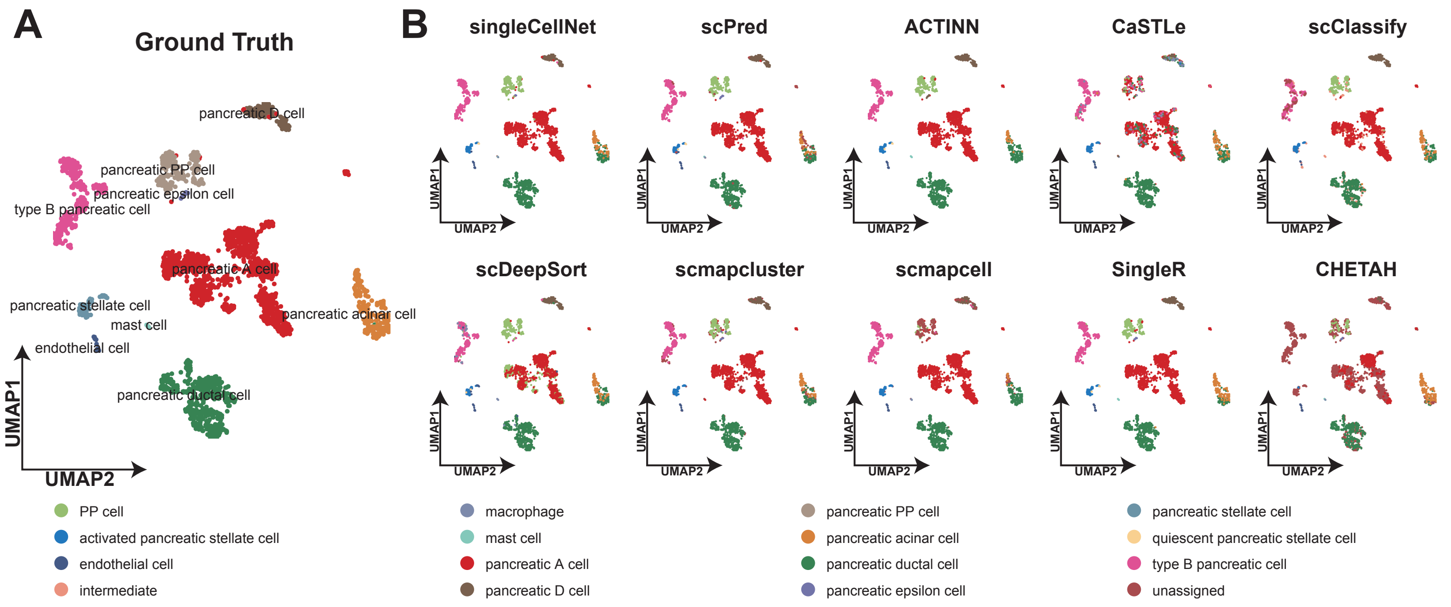

Supplementary Figure S19. (**A**) Ground truth UMAP of the Segerstolpe (Smart-seq2) query dataset. (**B**) UMAP of the Segerstolpe (Smart-seq2) query showing method-specific predicted cell type annotations using hBaron (inDrop) as the reference.

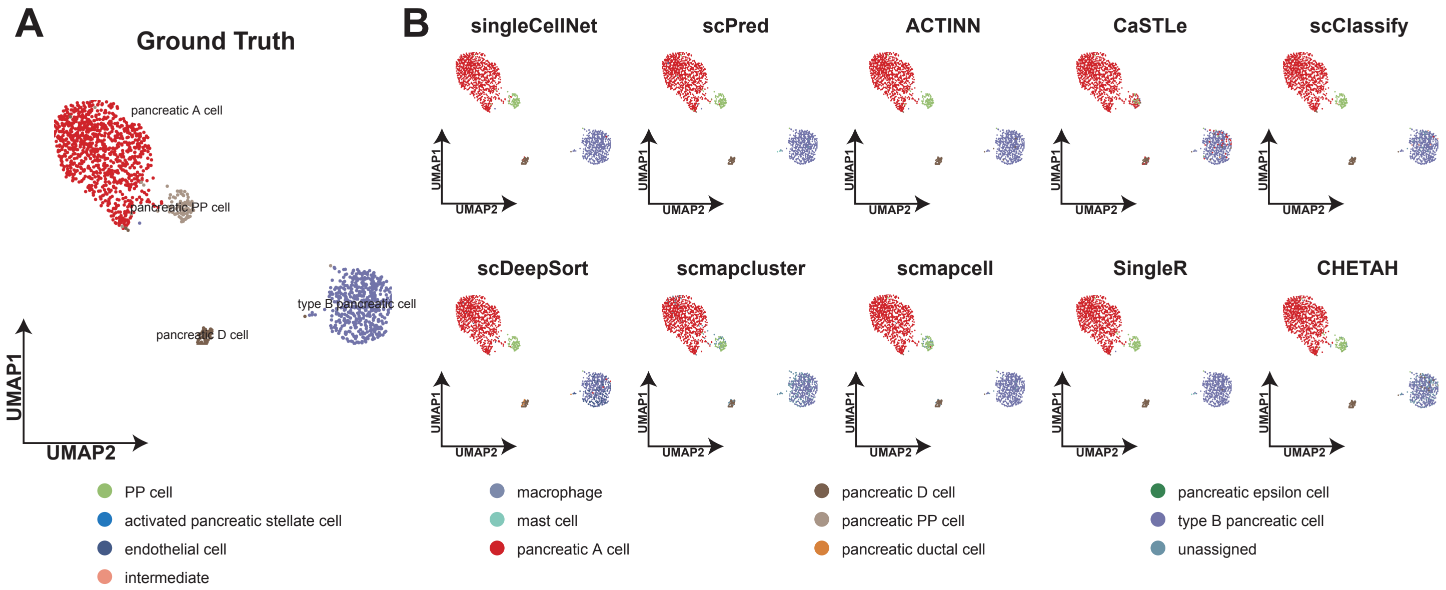

Supplementary Figure S20. (**A**) Ground truth UMAP of the Xin (SMARTer) query dataset. (**B**) UMAP of the Xin (SMARTer) query showing method-specific predicted cell type annotations using hBaron (inDrop) as the reference.

Supplementary Table S4. Datasets for Cross Platform Benchmarking

**Datasets**	**#celltype**	**#cell**	**#gene**	**Tissue**	**Species**	**Condition**	**Protocol**	**Reference**
hBaron	14	8569	18343	pancreas	human	health	inDrop	[21]
Muraro	10	2126	16494	pancreas	human	health	CEL-seq2	[22]
Segerstolpe	10	2122	21795	pancreas	human	health	Smart-seq2	[23]
Xin	4	1600	28255	pancreas	human	health	SMARTer	[24]

Supplementary Table S5. Method Performance on Cross-Platform Benchmarks

	method	hBaron(r) Muraro(q)	hBaron(r) Segerstolpe(q)	hBaron(r) Xin(q)	Muraro(r) Segerstolpe(q)	Muraro(r) Xin(q)	Segerstolpe(r) Muraro(q)	Segerstolpe(r) Xin(q)	Xin(r) Muraro(q)	Xin(r) Segerstolpe(q)
Acc	singleCellNet	0.8001	0.8242	0.9390	0.9571	0.9879	0.9229	0.9946	0.6816	0.6843
	scPred	0.8166	0.8087	0.9330	0.9345	0.9779	0.9102	0.9886	0.6957	0.6786
	ACTINN	0.7879	0.8058	0.9383	0.9477	0.9906	0.9233	0.9953	0.6091	0.6640
	CaSTLe	0.7418	0.7097	0.9035	0.8322	0.9169	0.8739	0.9879	0.6905	0.6838
	scClassify	0.6458	0.7328	0.9296	0.9350	0.9658	0.8617	0.9866	0.3316	0.6810
	scDeepSort	0.7817	0.6706	0.8713	0.9463	0.9940	0.8885	0.9960	0.6877	0.6852
	scampcluster	0.8222	0.7733	0.7802	0.9449	0.9383	0.8942	0.8311	0.5720	0.6329
	scmapcell	0.7766	0.7828	0.9323	0.8930	0.9692	0.8932	0.9792	0.6580	0.5386
	SingleR	0.8650	0.8492	0.9390	0.9618	0.9913	0.9214	0.9953	0.7051	0.6871
	CHETAH	0.7789	0.3035	0.8177	0.1923	0.4504	0.8598	0.8820	0.4619	0.4072
F1-score	singleCellNet	0.8001	0.8242	0.9390	0.9571	0.9879	0.9229	0.9946	0.6816	0.6843
	scPred	0.8166	0.8087	0.9330	0.9345	0.9779	0.9102	0.9886	0.6957	0.6786
	ACTINN	0.7879	0.8058	0.9383	0.9477	0.9906	0.9233	0.9953	0.6091	0.6640
	CaSTLe	0.7418	0.7097	0.9035	0.8322	0.9169	0.8739	0.9879	0.6905	0.6838
	scClassify	0.6458	0.7328	0.9296	0.9350	0.9658	0.8617	0.9866	0.3316	0.6810
	scDeepSort	0.7817	0.6706	0.8713	0.9463	0.9940	0.8885	0.9960	0.6877	0.6852
	scampcluster	0.8222	0.7733	0.7802	0.9449	0.9383	0.8942	0.8311	0.5720	0.6329
	scmapcell	0.7766	0.7828	0.9323	0.8930	0.9692	0.8932	0.9792	0.6580	0.5386
	SingleR	0.8650	0.8492	0.9390	0.9618	0.9913	0.9214	0.9953	0.7051	0.6871
	CHETAH	0.7789	0.3035	0.8177	0.1923	0.4504	0.8598	0.8820	0.4619	0.4072
MCC	singleCellNet	0.7531	0.7805	0.8925	0.9440	0.9778	0.9026	0.9901	0.6140	0.6097
	scPred	0.7721	0.7625	0.8831	0.9155	0.9593	0.8871	0.9790	0.6217	0.6231
	ACTINN	0.7390	0.7584	0.8923	0.9315	0.9827	0.9030	0.9914	0.5557	0.6048
	CaSTLe	0.6778	0.6164	0.8198	0.7850	0.8475	0.8424	0.9777	0.6094	0.6050
	scClassify	0.5841	0.6687	0.8767	0.9154	0.9387	0.8312	0.9759	0.2379	0.5998
	scDeepSort	0.7313	0.6339	0.7810	0.9299	0.9889	0.8581	0.9926	0.6041	0.6436
	scampcluster	0.7809	0.7209	0.6649	0.9288	0.8951	0.8687	0.7491	0.5347	0.5824
	scmapcell	0.7255	0.7284	0.8804	0.8650	0.9446	0.8664	0.9623	0.5695	0.3881
	SingleR	0.8314	0.8130	0.8932	0.9503	0.9840	0.9011	0.9914	0.6528	0.6075
	CHETAH	0.7317	0.3730	0.7097	0.2725	0.4480	0.8295	0.8190	0.4331	0.4150
NMI	singleCellNet	0.8393	0.9085	0.9573	0.9469	0.9322	0.8969	0.9698	0.5939	0.6217
	scPred	0.8358	0.8986	0.9468	0.9211	0.9036	0.8700	0.9451	0.6302	0.7563
	ACTINN	0.8455	0.9194	0.9645	0.9329	0.9513	0.8880	0.9718	0.5983	0.6336
	CaSTLe	0.6577	0.5736	0.6772	0.7726	0.7242	0.7872	0.9334	0.5518	0.5645
	scClassify	0.7291	0.8003	0.9421	0.9169	0.8914	0.8064	0.9602	0.1787	0.6295
	scDeepSort	0.8337	0.7707	0.8451	0.9140	0.9630	0.8292	0.9736	0.5180	0.7481
	scampcluster	0.8425	0.8124	0.7156	0.9431	0.8430	0.8653	0.7018	0.5178	0.6433
	scmapcell	0.8134	0.8471	0.9217	0.8905	0.8975	0.8597	0.9351	0.5586	0.3994
	SingleR	0.8786	0.9413	0.9661	0.9695	0.9523	0.8937	0.9729	0.6684	0.5757
	CHETAH	0.7915	0.4532	0.8167	0.5682	0.4640	0.8412	0.7808	0.4809	0.4309
ARI	singleCellNet	0.8773	0.9186	0.9786	0.9669	0.9661	0.9363	0.9869	0.6637	0.6233
	scPred	0.8844	0.9143	0.9788	0.9511	0.9533	0.9221	0.9762	0.6638	0.7901
	ACTINN	0.8661	0.9168	0.9862	0.9404	0.9760	0.9254	0.9872	0.6014	0.6551
	CaSTLe	0.7145	0.5683	0.7796	0.8080	0.8434	0.8700	0.9751	0.5640	0.5950
	scClassify	0.7642	0.8464	0.9778	0.9530	0.9507	0.8337	0.9712	0.0298	0.3995
	scDeepSort	0.8662	0.6153	0.8985	0.9505	0.9808	0.8310	0.9867	0.5749	0.6797
	scampcluster	0.8910	0.8597	0.7938	0.9644	0.9000	0.9143	0.7278	0.4957	0.6603
	scmapcell	0.8710	0.8831	0.9764	0.9350	0.9557	0.9224	0.9778	0.5038	0.3430
	SingleR	0.9161	0.9547	0.9870	0.9778	0.9768	0.9297	0.9905	0.7127	0.6261
	CHETAH	0.8414	0.1682	0.8737	0.3658	0.3061	0.8719	0.7920	0.3389	0.2184

6 Supplementary Note 6: Supplementary for ”Benchmarking Annotation Methods in Cross-Species Scenarios”

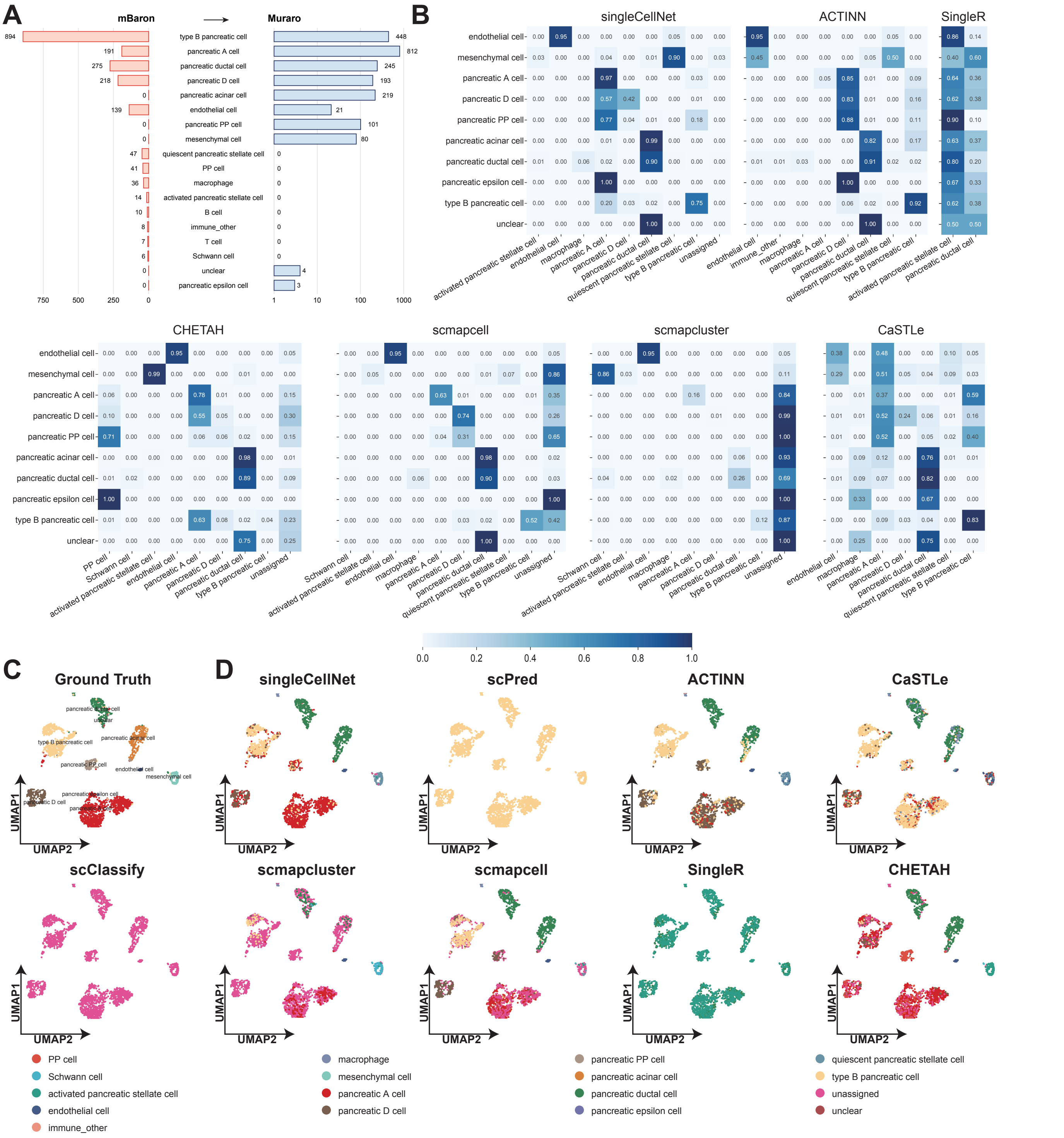

Supplementary Figure S21. (**A**) Cell-type composition of the mBaron reference and Muraro query datasets, reporting the number of cells per type in each dataset. (**B**) Comparison of method-specific annotations against the ground truth on the Muraron query using mBaron as the reference. (**C**) Ground truth UMAP of the Muraro query dataset. (**D**) UMAP of the Muraro query showing method-specific predicted cell type annotations using mBaron as the reference.

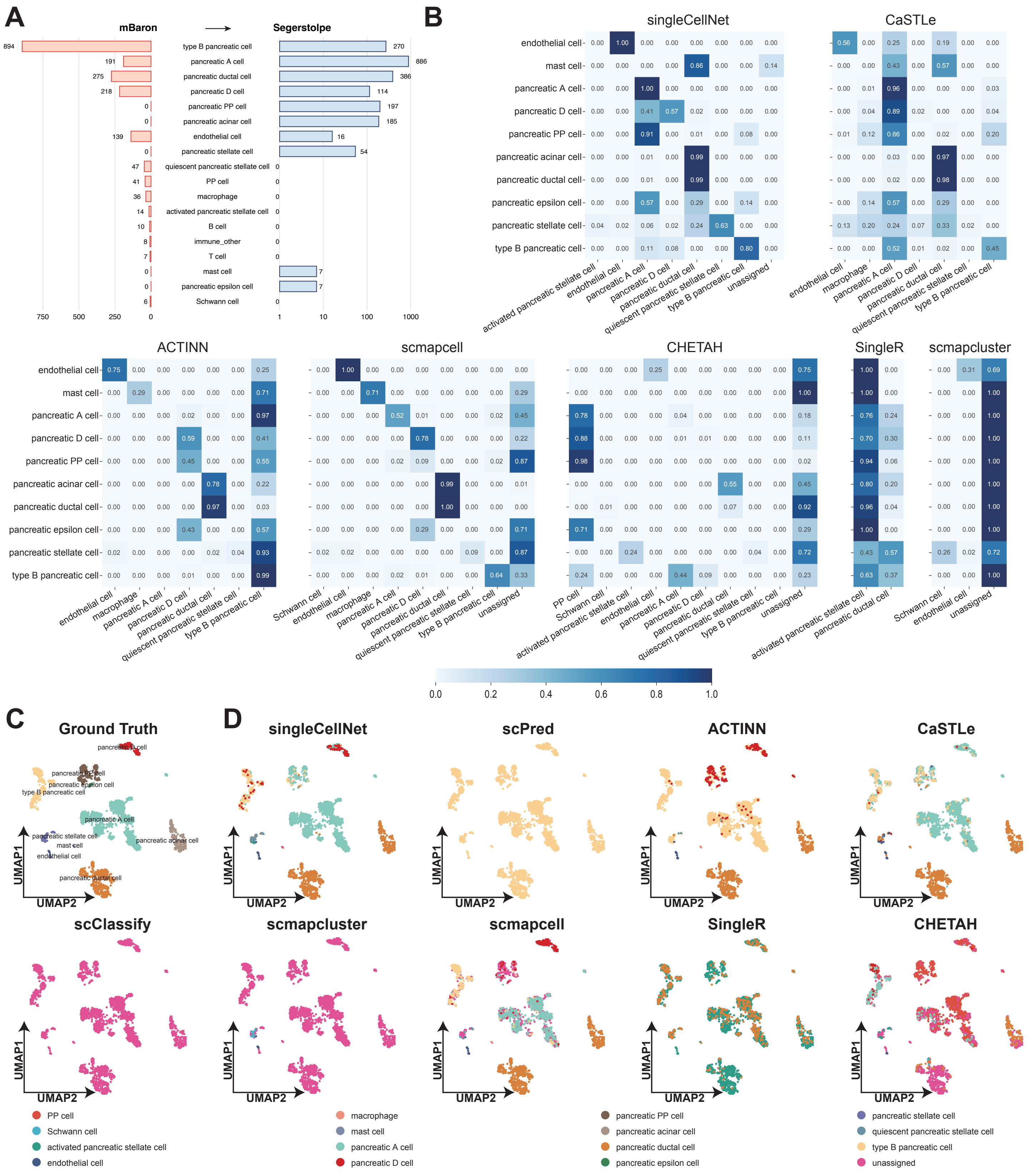

Supplementary Figure S22. (**A**) Cell-type composition of the mBaron reference and Segerstolpe query datasets, reporting the number of cells per type in each dataset. (**B**) Comparison of method-specific annotations against the ground truth on the Segerstolpe query using mBaron as the reference. (**C**) Ground truth UMAP of the Segerstolpe query dataset. (**D**) UMAP of the Segerstolpe query showing method-specific predicted cell type annotations using mBaron as the reference.

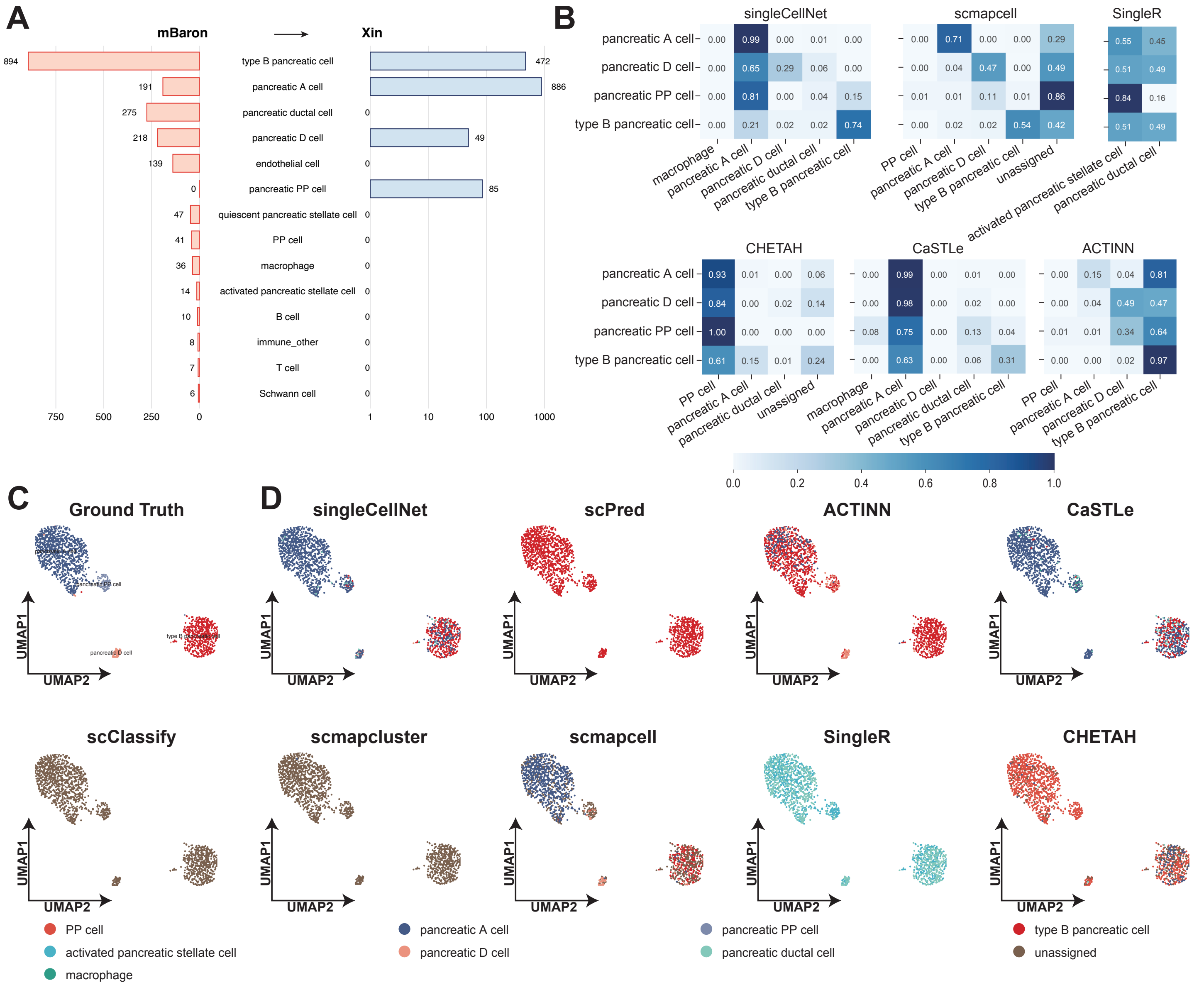

Supplementary Figure S23. (**A**) Cell-type composition of the mBaron reference and Xin query datasets, reporting the number of cells per type in each dataset. (**B**) Comparison of method-specific annotations against the ground truth on the Xin query using mBaron as the reference. (**C**) Ground truth UMAP of the Xin query dataset. (**D**) UMAP of the Xin query showing method-specific predicted cell type annotations using mBaron as the reference.

Supplementary Table S6. Datasets for Cross Species Benchmarking

Datasets	#celltype	#cell	#gene	Tissue	Species	Condition	Protocol	Reference
mBaron	13	1886	13491	pancreas	mouse	health	inDrop	[21]
hBaron	14	8569	18343	pancreas	human	health	inDrop	[21]
hMuraro	10	2126	16494	pancreas	human	health	CEL-seq2	[22]
hSegerstolpe	10	2122	21795	pancreas	human	health	Smart-seq2	[23]
hXin	4	1600	28255	pancreas	human	health	SMARTer	[24]

Supplementary Table S7. Method Performance on Cross-Species Benchmarks

	method	Muraro	Segerstolpe	Xin	hBaron
Acc	singleCellNet	0.6802	0.7380	0.8331	0.7150
	scPred	0.2107	0.1272	0.3164	0.2947
	ACTINN	0.4036	0.3398	0.4135	0.6158
	CaSTLe	0.4389	0.6404	0.6823	0.6262
	scClassify	0.0000	0.0000	0.0000	0.0000
	scmapcluster	0.1242	0.0024	0.0007	0.2838
	scmapcell	0.5310	0.5325	0.6052	0.5093
	SingleR	0.0230	0.0080	0.0000	0.0378
	CHETAH	0.4229	0.0311	0.0060	0.1747
F1-score	singleCellNet	0.6802	0.7380	0.8331	0.7150
	scPred	0.2107	0.1272	0.3164	0.2947
	ACTINN	0.4036	0.3398	0.4135	0.6158
	CaSTLe	0.4389	0.6404	0.6823	0.6262
	scClassify	0.0000	0.0000	0.0000	0.0000
	scmapcluster	0.1242	0.0024	0.0007	0.2838
	scmapcell	0.5310	0.5325	0.6052	0.5093
	SingleR	0.0230	0.0080	0.0000	0.0378
	CHETAH	0.4229	0.0311	0.0060	0.1747
MCC	singleCellNet	0.5869	0.6523	0.6853	0.6585
	scPred	0.0000	0.0000	0.0000	0.0000
	ACTINN	0.3789	0.3361	0.2330	0.5441
	CaSTLe	0.2941	0.5067	0.3790	0.5577
	scClassify	0.0000	0.0000	0.0000	0.0000
	scmapcluster	0.1875	0.0196	0.0101	0.3293
	scmapcell	0.4928	0.5001	0.4980	0.4845
	SingleR	−0.0281	−0.0602	0.0000	−0.0207
	CHETAH	0.2858	−0.0159	−0.0621	0.1444
NMI	singleCellNet	0.6236	0.6818	0.4776	0.6572
	scPred	0.0000	0.0000	0.0000	0.0000
	ACTINN	0.5520	0.4812	0.1232	0.5649
	CaSTLe	0.3460	0.4758	0.1914	0.4882
	scClassify	0.0000	0.0000	0.0000	0.0000
	scmapcluster	0.2727	0.0429	0.0007	0.3646
	scmapcell	0.5737	0.6096	0.4231	0.5705
	SingleR	0.0178	0.0462	0.0141	0.0462
	CHETAH	0.4802	0.4098	0.1121	0.4078
ARI	singleCellNet	0.5627	0.6304	0.5632	0.6323
	scPred	0.0000	0.0000	0.0000	0.0000
	ACTINN	0.4903	0.3990	0.0299	0.4095
	CaSTLe	0.2677	0.4539	0.1983	0.4902
	scClassify	0.0000	0.0000	0.0000	0.0000
	scmapcluster	0.0448	0.0114	−0.0007	0.1312
	scmapcell	0.4157	0.4229	0.3898	0.4453
	SingleR	−0.0019	0.0048	−0.0005	0.0364
	CHETAH	0.3545	0.3123	0.1279	0.2780

7 Supplementary Note 7: Supplementary for "Benchmarking Annotation Methods for Unknown Cell Type Detection"

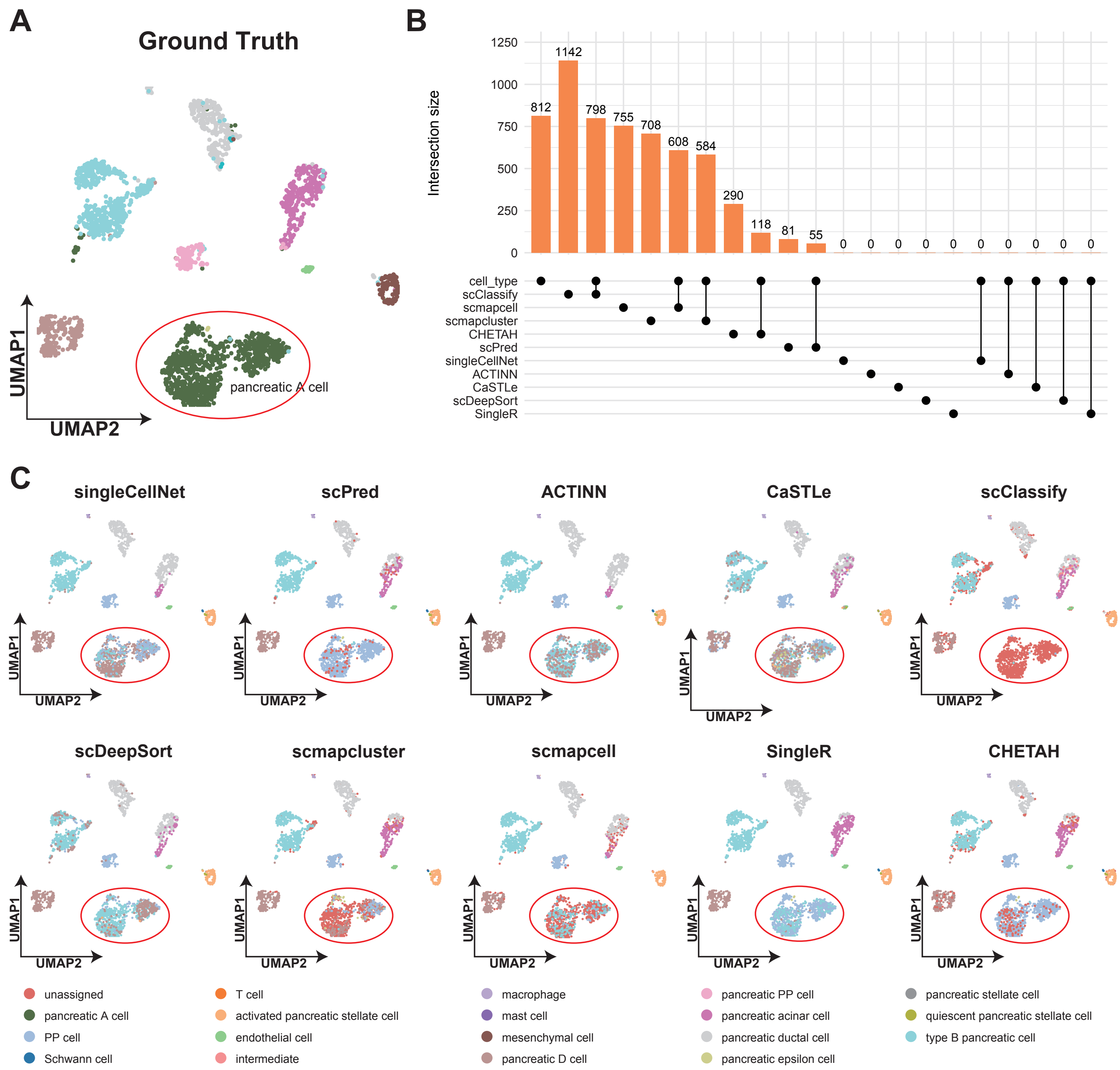

Supplementary Figure S24. **(A)** Ground truth of the Muraro query dataset **(B)** Label-level behavior in the missing-class setting further quantified on the Muraro dataset by partitioning pancreatic A cells into unassigned abstentions and reassigned labels and examining cooccurrence patterns across methods. **(C)** Method-specific predicted cell type annotations of the Muraro query dataset with differences in counts localized to the pancreatic A cell region.

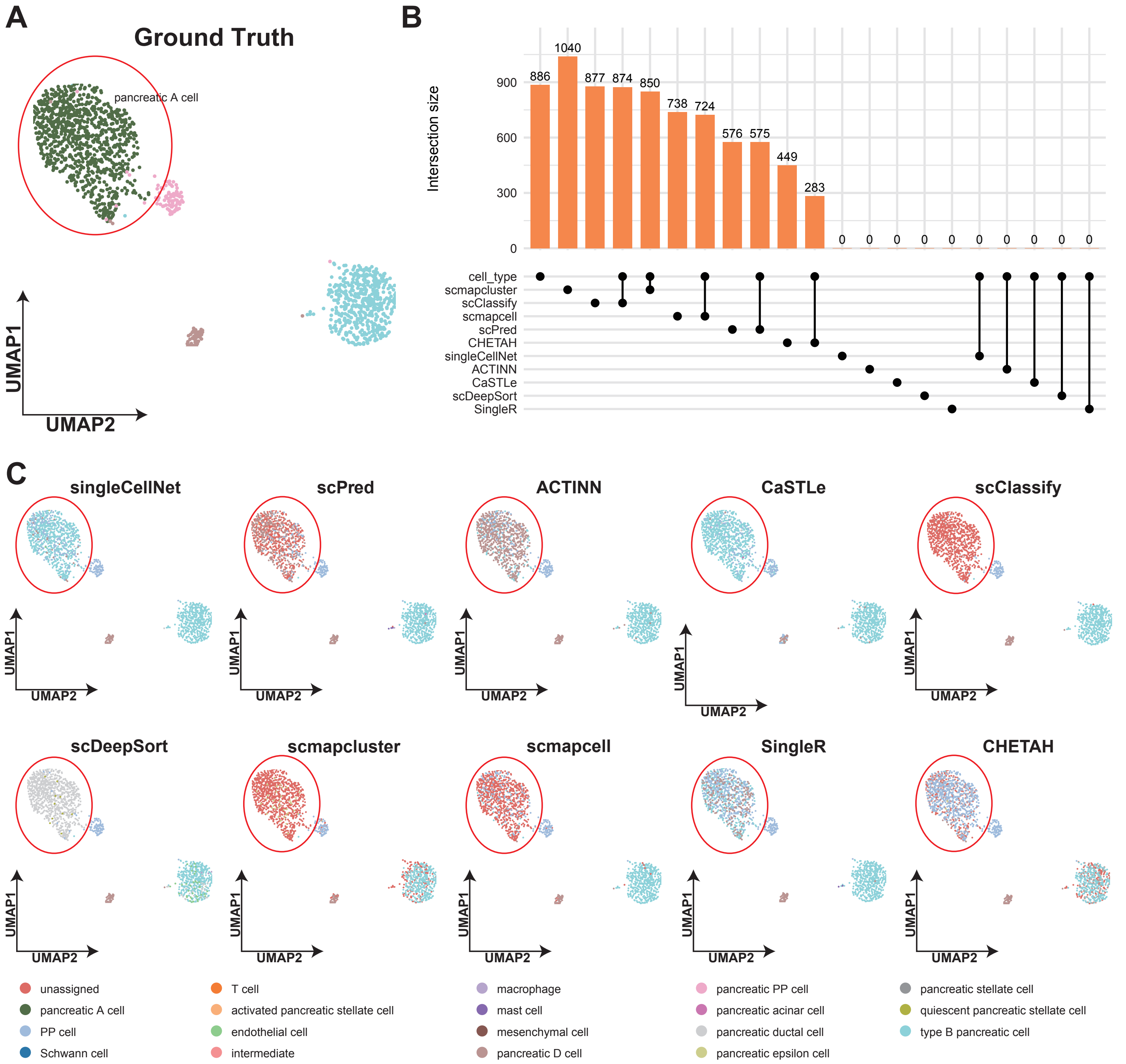

Supplementary Figure S25. **(A)** Ground truth of the Xin query dataset **(B)** Label-level behavior in the missing-class setting further quantified on the Xin dataset by partitioning pancreatic A cells into unassigned abstentions and reassigned labels and examining cooccurrence patterns across methods. **(C)** Method-specific predicted cell type annotations of the Xin query dataset with differences in counts localized to the pancreatic A cell region.

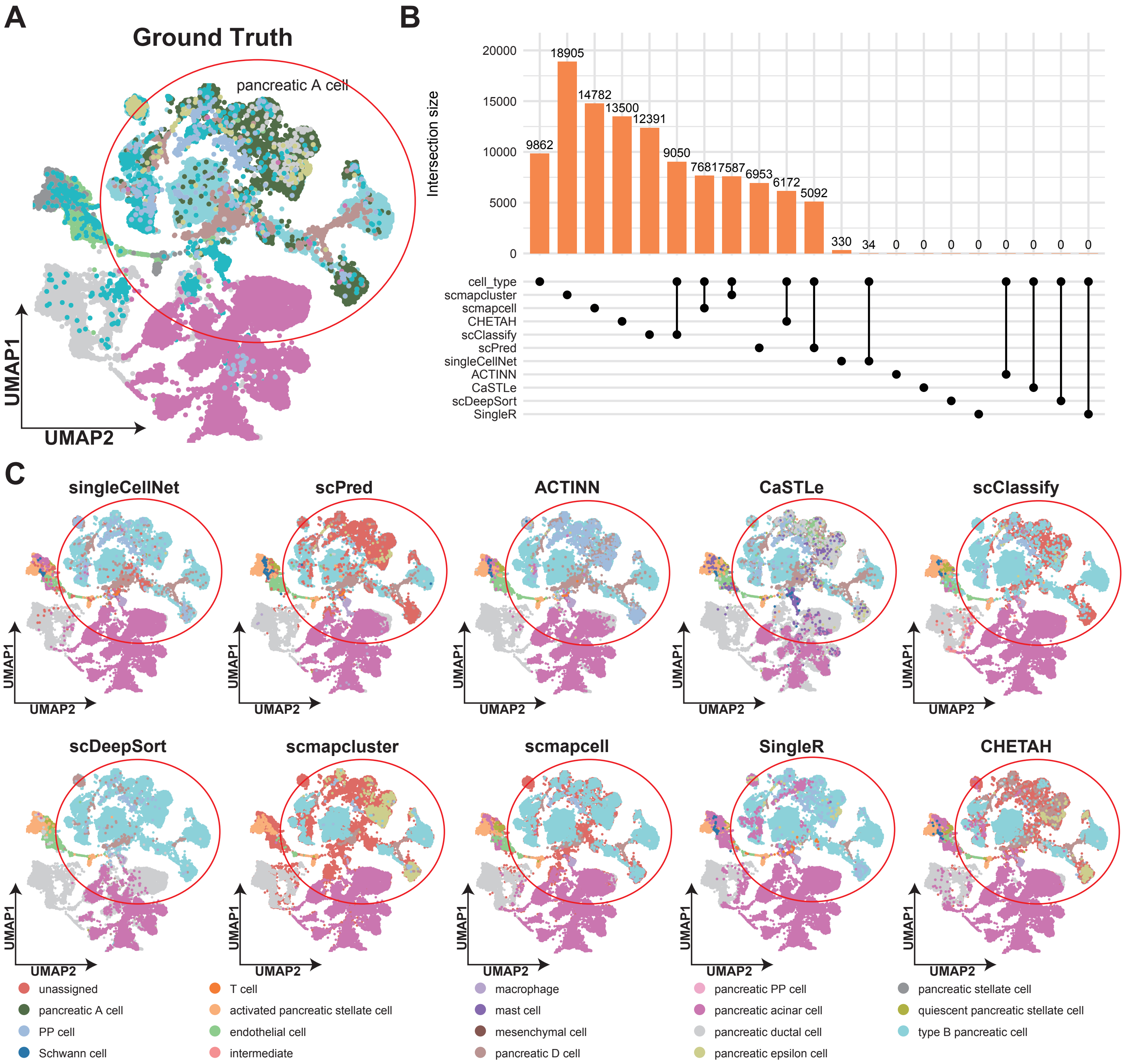

Supplementary Figure S26. **(A)** Ground truth of the Fasolino query dataset **(B)** Label-level behavior in the missing-class setting further quantified on the Fasolino dataset by partitioning pancreatic A cells into unassigned abstentions and reassigned labels and examining cooccurrence patterns across methods. **(C)** Method-specific predicted cell type annotations of the Fasolino query dataset with differences in counts localized to the pancreatic A cell region.

Supplementary Table S8. Datasets for Unknown Benchmarking

Datasets	#celltype	#cell	#gene	Tissue	Species	Condition	Protocol	Reference
hBaron	13	6243	18343	pancreas	human	health	inDrop	[21]
Muraro	10	2126	16494	pancreas	human	health	CEL-seq2	[22]
Segerstolpe	10	2122	21795	pancreas	human	health	Smart-seq2	[23]
Xin	4	1600	28255	pancreas	human	health	SMARTer	[24]
Fasolino	10	47245	26099	health	human	health	10x	[25]

Supplementary Table S9. Method Performance on Unknown Cell Type Detection

	method	Muraro	Segerstolpe	Xin	Fasolino
Acc	singleCellNet	0.4102	0.4053	0.3485	0.6353
	scPred	0.4459	0.4015	0.3438	0.6117
	ACTINN	0.4158	0.3897	0.3452	0.5910
	CaSTLe	0.3688	0.3412	0.3311	0.4299
	scClassify	0.2940	0.3534	0.3472	0.6112
	scDeepSort	0.3725	0.3831	0.3016	0.4455
	scampcluster	0.4454	0.3723	0.2332	0.5112
	scmapcell	0.3956	0.3662	0.3418	0.5620
	SingleR	0.4944	0.4336	0.3465	0.6273
	CHETAH	0.4102	0.2083	0.2306	0.5318
F1-score	singleCellNet	0.4102	0.4053	0.3485	0.6353
	scPred	0.4459	0.4015	0.3438	0.6117
	ACTINN	0.4158	0.3897	0.3452	0.5910
	CaSTLe	0.3688	0.3412	0.3311	0.4299
	scClassify	0.2940	0.3534	0.3472	0.6112
	scDeepSort	0.3725	0.3831	0.3016	0.4455
	scampcluster	0.4454	0.3723	0.2332	0.5112
	scmapcell	0.3956	0.3662	0.3418	0.5620
	SingleR	0.4944	0.4336	0.3465	0.6273
	CHETAH	0.4102	0.2083	0.2306	0.5318
MCC	singleCellNet	0.3982	0.4200	0.2150	0.5817
	scPred	0.4827	0.4477	0.3841	0.5681
	ACTINN	0.3754	0.3912	0.3584	0.5282
	CaSTLe	0.3325	0.3347	0.1533	0.3856
	scClassify	0.3398	0.4020	0.4497	0.5792
	scDeepSort	0.3299	0.3880	0.3909	0.3738
	scampcluster	0.4703	0.4195	0.3293	0.5038
	scmapcell	0.4047	0.3792	0.3950	0.5342
	SingleR	0.5233	0.5081	0.2870	0.5677
	CHETAH	0.4446	0.3248	0.2749	0.4979
NMI	singleCellNet	0.6407	0.6551	0.3625	0.6472
	scPred	0.7646	0.7655	0.6641	0.6481
	ACTINN	0.6179	0.6481	0.5526	0.5849
	CaSTLe	0.4222	0.4500	0.2354	0.5088
	scClassify	0.6498	0.7740	0.9502	0.6724
	scDeepSort	0.5309	0.5960	0.8347	0.5240
	scampcluster	0.6733	0.7327	0.5860	0.5766
	scmapcell	0.6355	0.6658	0.6569	0.5606
	SingleR	0.7362	0.8024	0.3368	0.6096
	CHETAH	0.6948	0.4663	0.4825	0.5531
ARI	singleCellNet	0.5006	0.4144	0.1798	0.5855
	scPred	0.7437	0.5894	0.5850	0.6627
	ACTINN	0.4085	0.3819	0.5449	0.4868
	CaSTLe	0.2390	0.2677	0.1473	0.4210
	scClassify	0.5313	0.7795	0.9723	0.7197
	scDeepSort	0.3254	0.3353	0.8808	0.3478
	scampcluster	0.5758	0.6614	0.5552	0.4841
	scmapcell	0.5422	0.5029	0.6791	0.5463
	SingleR	0.6336	0.7085	0.1443	0.5536
	CHETAH	0.6434	0.2752	0.3741	0.5378

8 Supplementary Note 8: Supplementary for "Benchmarking Annotation Methods Across Marker Database"

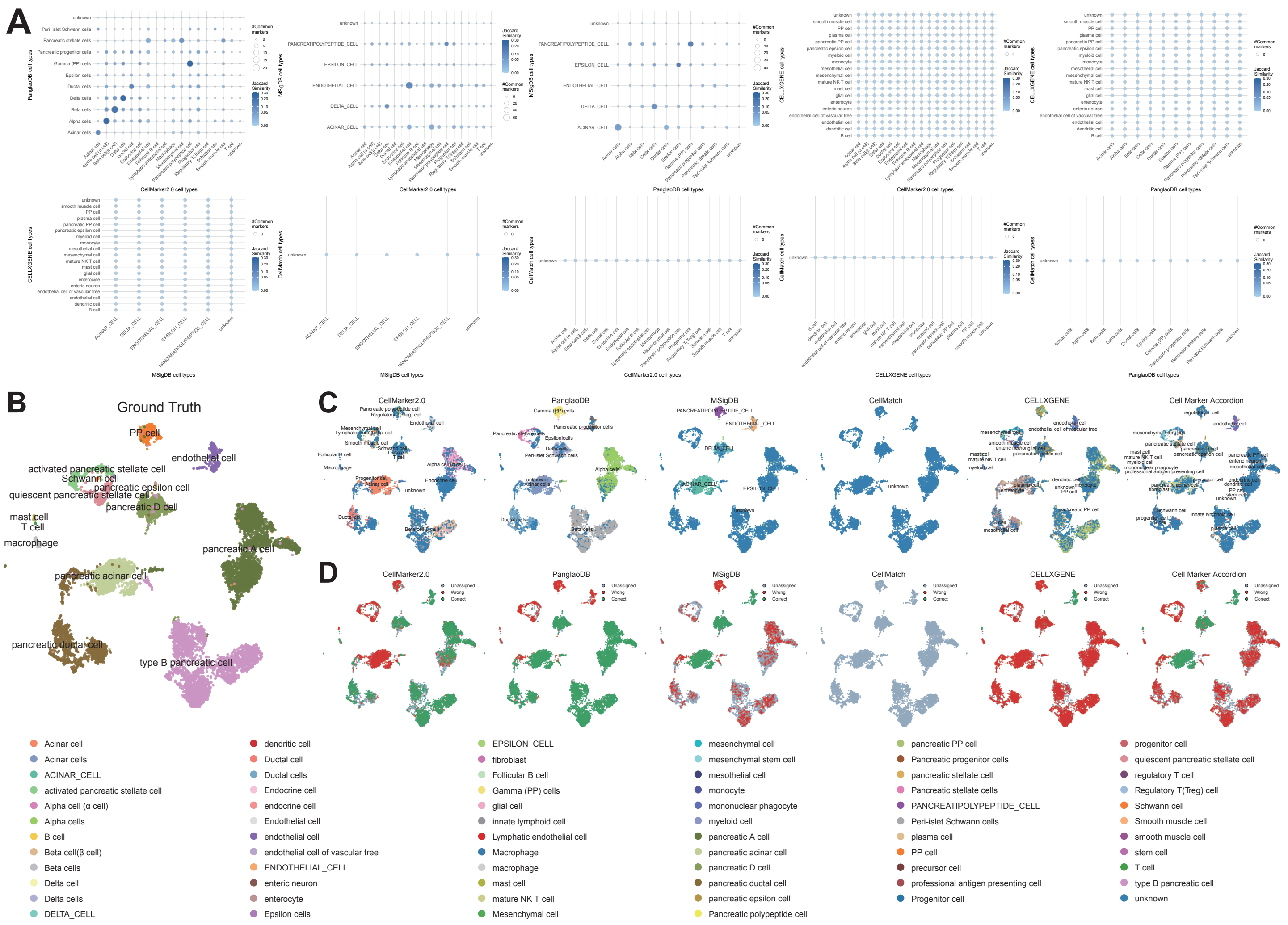

Supplementary Figure S27. (**A**) Overlap of marker genes across databases for hBaron cell types annotated by SCINA with different marker resources, in which dot color encodes the Jaccard similarity and dot size indicates the number of shared markers for each cell-type pair. (**B**) Ground truth UMAP of the hBaron query dataset. (**C**) UMAPs of the hBaron query showing SCINA-predicted annotations using different marker gene sets. (**D**) UMAPs comparing SCINA annotations from different marker gene sets with ground truth.

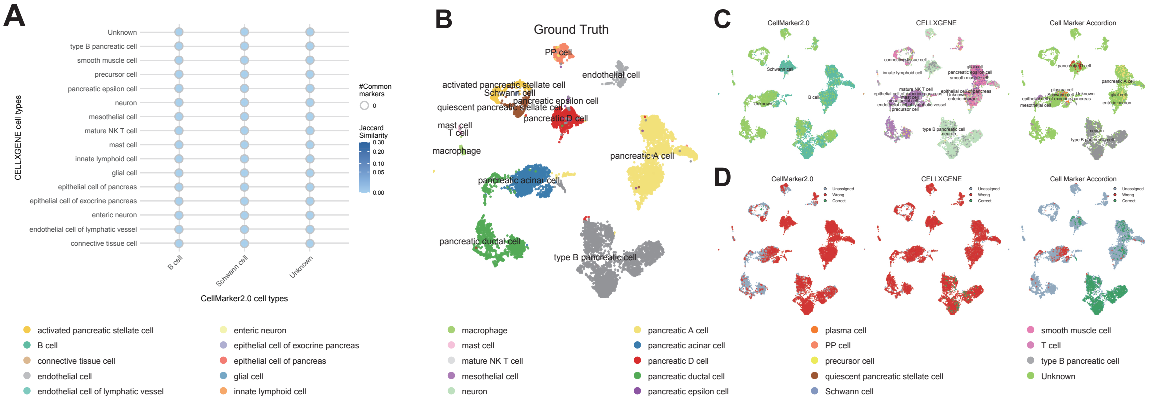

Supplementary Figure S28. (**A**) Overlap of marker genes across databases for hBaron cell types annotated by Garnett with different marker resources, in which dot color encodes the Jaccard similarity and dot size indicates the number of shared markers for each cell-type pair. (**B**) Ground truth UMAP of the hBaron query dataset. (**C**) UMAPs of the hBaron query showing Garnett-predicted annotations using different marker gene sets. (**D**) UMAPs comparing Garnett annotations from different marker gene sets with ground truth.

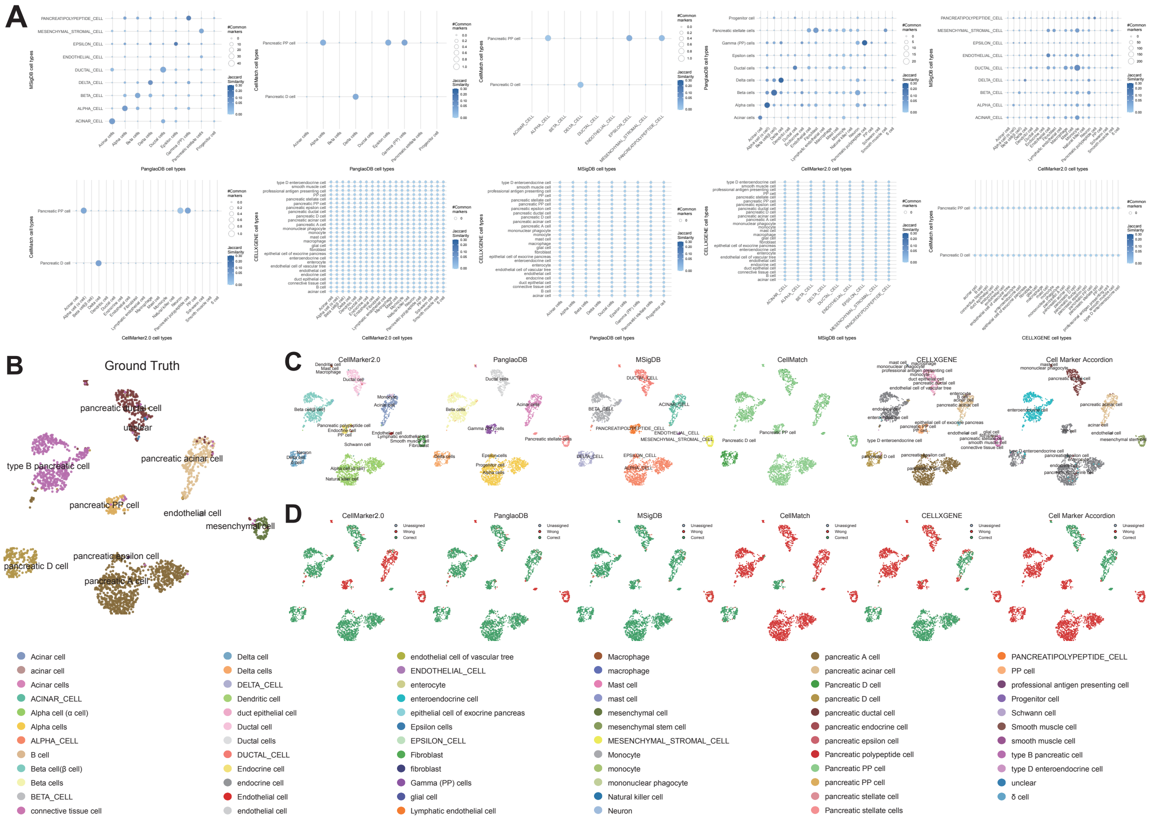

Supplementary Figure S29. (**A**) Overlap of marker genes across databases for hMuraro cell types annotated by CellAssign with different marker resources, in which dot color encodes the Jaccard similarity and dot size indicates the number of shared markers for each cell-type pair. (**B**) Ground truth UMAP of the hMuraro query dataset. (**C**) UMAPs of the hMuraro query showing CellAssign-predicted annotations using different marker gene sets. (**D**) UMAPs comparing CellAssign annotations from different marker gene sets with ground truth.

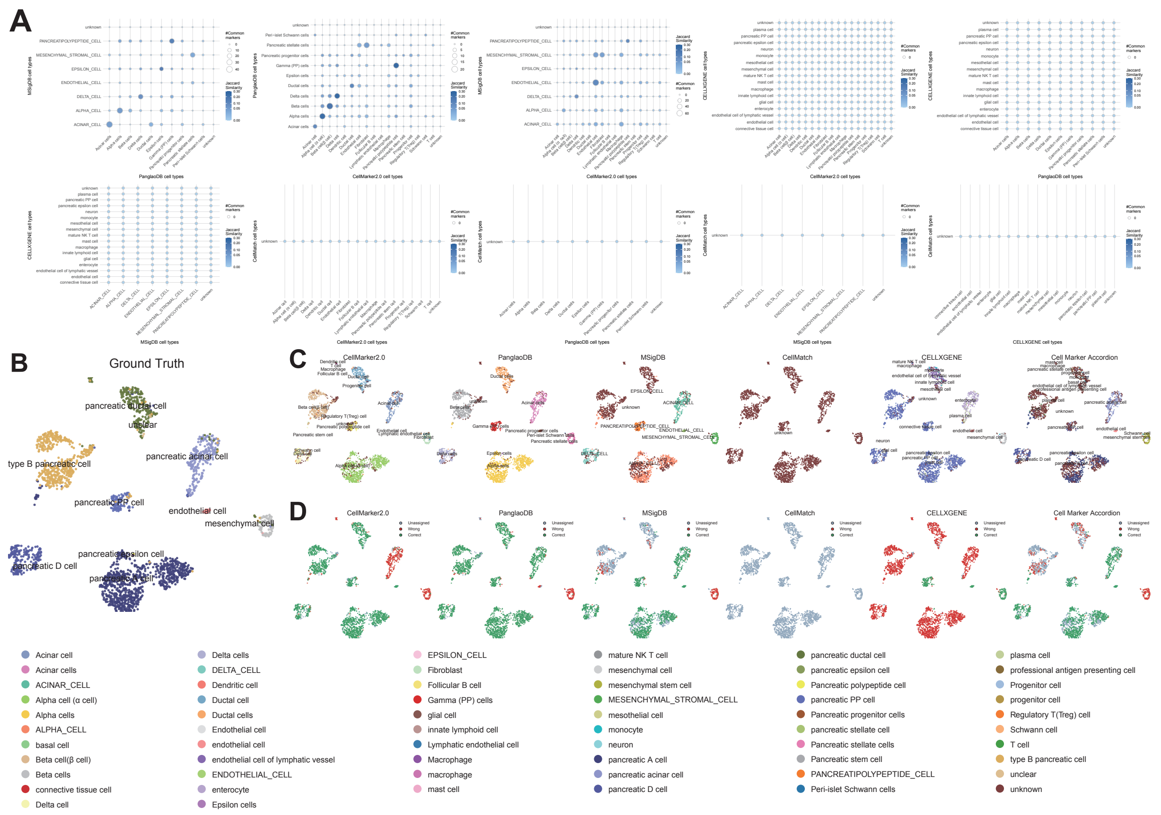

Supplementary Figure S30. (**A**) Overlap of marker genes across databases for hMuraro cell types annotated by SCINA with different marker resources, in which dot color encodes the Jaccard similarity and dot size indicates the number of shared markers for each cell-type pair. (**B**) Ground truth UMAP of the hMuraro query dataset. (**C**) UMAPs of the hMuraro query showing SCINA-predicted annotations using different marker gene sets. (**D**) UMAPs comparing SCINA annotations from different marker gene sets with ground truth.

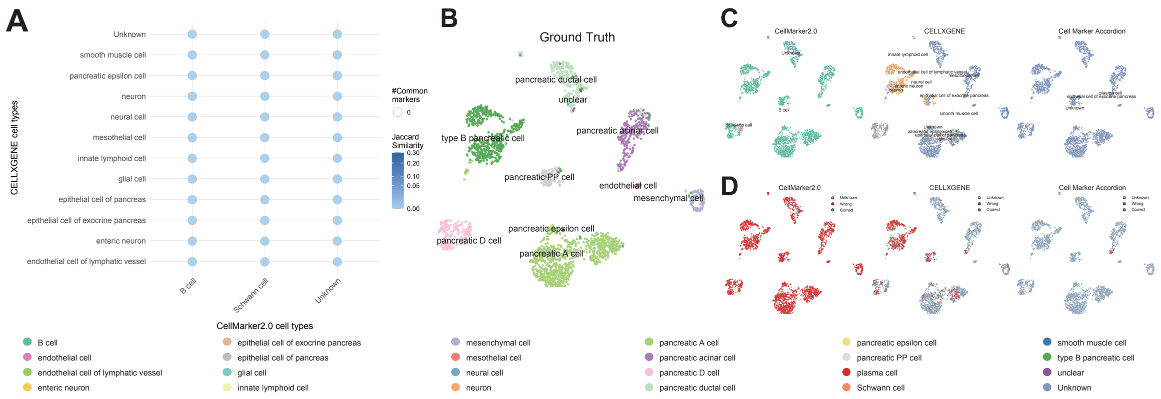

Supplementary Figure S31. (**A**) Overlap of marker genes across databases for hMuraro cell types annotated by Garnett with different marker resources, in which dot color encodes the Jaccard similarity and dot size indicates the number of shared markers for each cell-type pair. (**B**) Ground truth UMAP of the hMuraro query dataset. (**C**) UMAPs of the hMuraro query showing Garnett-predicted annotations using different marker gene sets. (**D**) UMAPs comparing Garnett annotations from different marker gene sets with ground truth.

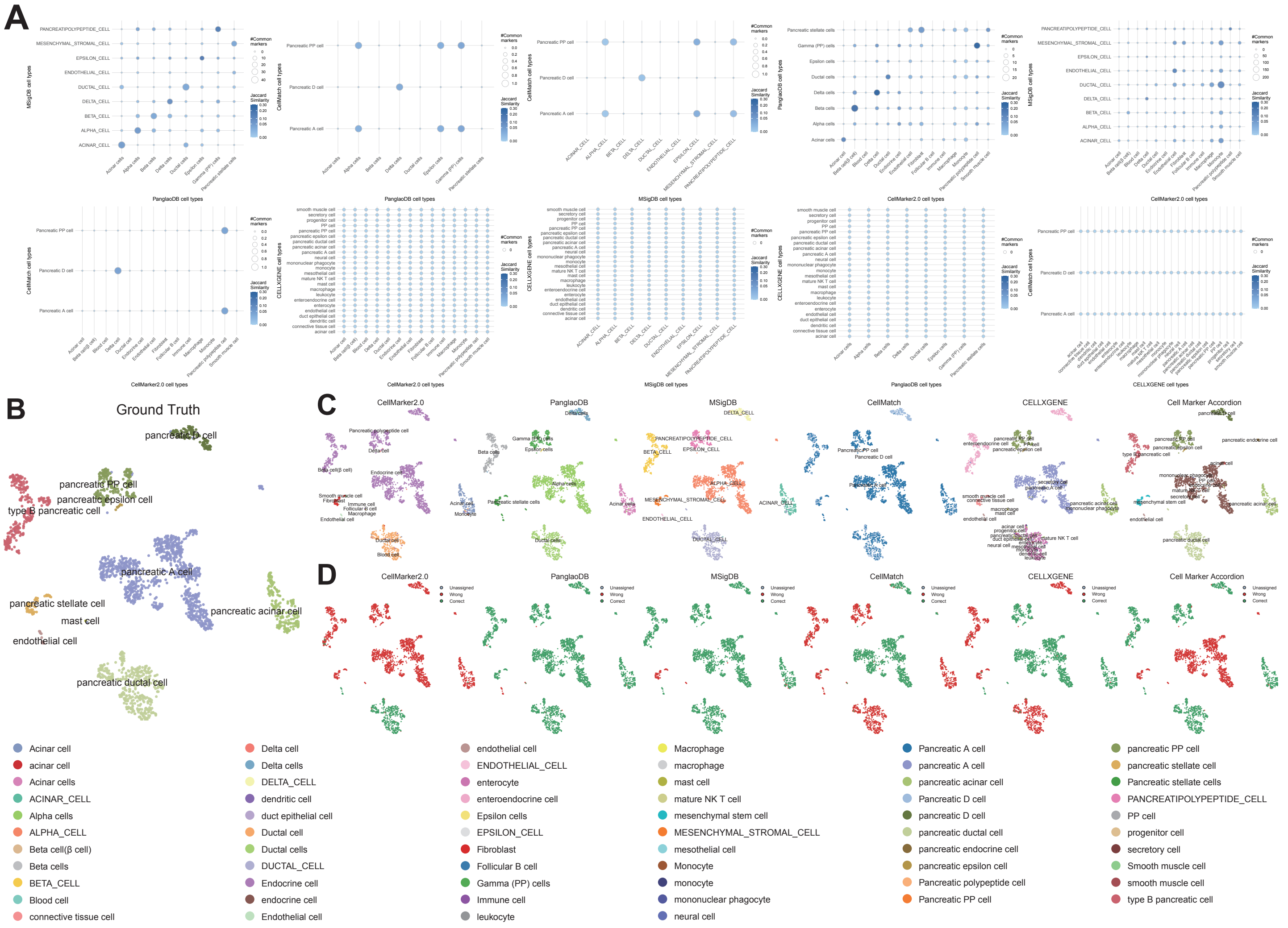

Supplementary Figure S32. (**A**) Overlap of marker genes across databases for hSegerstolpe cell types annotated by CellAssign with different marker resources, in which dot color encodes the Jaccard similarity and dot size indicates the number of shared markers for each cell-type pair. (**B**) Ground truth UMAP of the hSegerstolpe query dataset. (**C**) UMAPs of the hSegerstolpe query showing CellAssign-predicted annotations using different marker gene sets. (**D**) UMAPs comparing CellAssign annotations from different marker gene sets with ground truth.

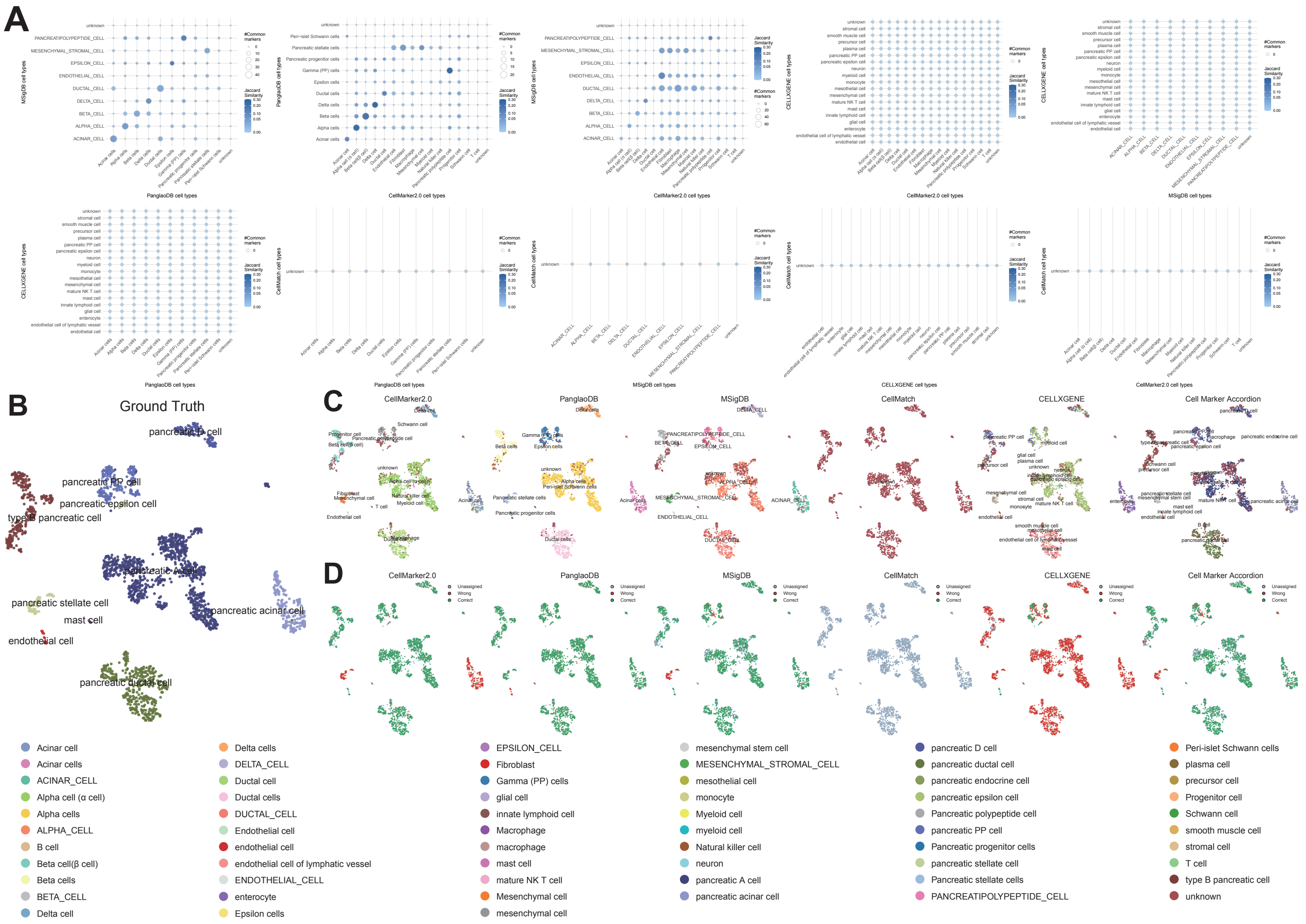

Supplementary Figure S33. (**A**) Overlap of marker genes across databases for hSegerstolpe cell types annotated by SCINA with different marker resources, in which dot color encodes the Jaccard similarity and dot size indicates the number of shared markers for each cell-type pair. (**B**) Ground truth UMAP of the hSegerstolpe query dataset. (**C**) UMAPs of the hSegerstolpe query showing SCINA-predicted annotations using different marker gene sets. (**D**) UMAPs comparing SCINA annotations from different marker gene sets with ground truth.

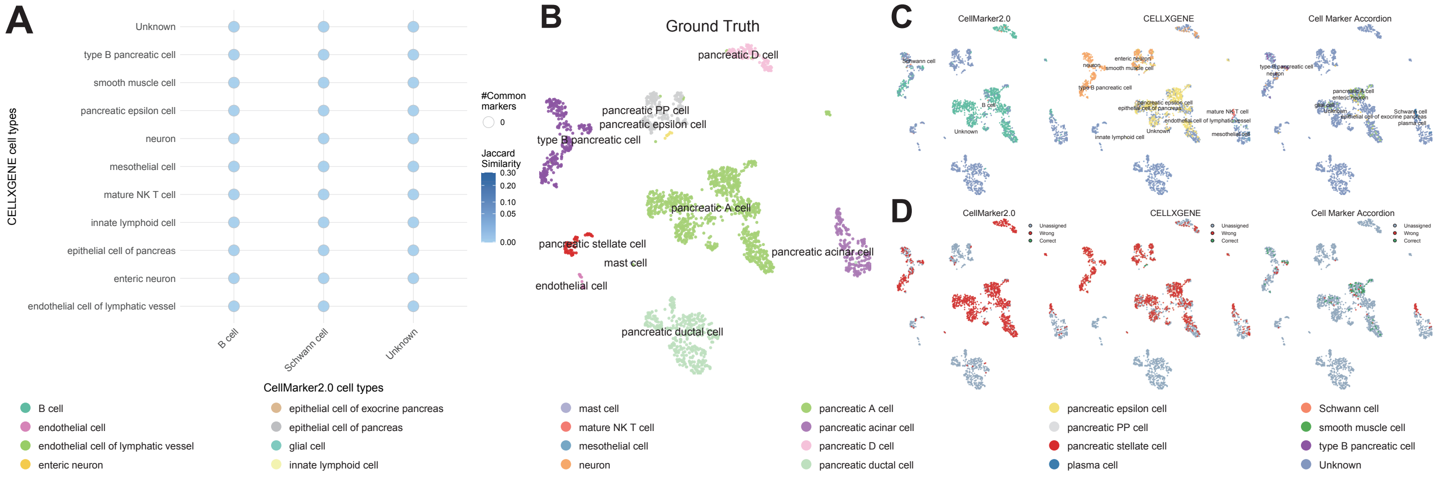

Supplementary Figure S34. (**A**) Overlap of marker genes across databases for hSegerstolpe cell types annotated by Garnett with different marker resources, in which dot color encodes the Jaccard similarity and dot size indicates the number of shared markers for each cell-type pair. (**B**) Ground truth UMAP of the hSegerstolpe query dataset. (**C**) UMAPs of the hSegerstolpe query showing Garnett-predicted annotations using different marker gene sets. (**D**) UMAPs comparing Garnett annotations from different marker gene sets with ground truth.

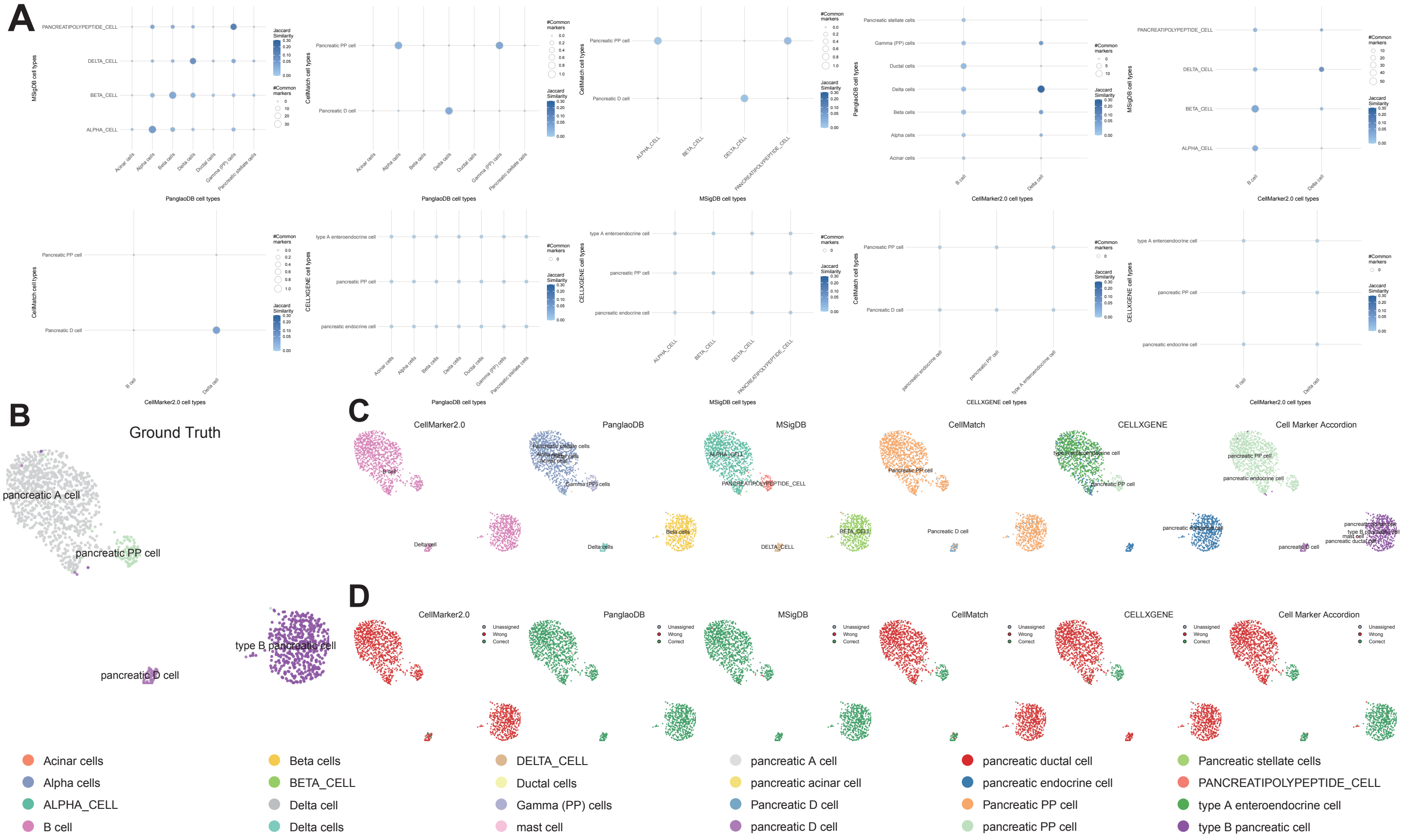

Supplementary Figure S35. (**A**) Overlap of marker genes across databases for hXin cell types annotated by CellAssign with different marker resources, in which dot color encodes the Jaccard similarity and dot size indicates the number of shared markers for each cell-type pair. (**B**) Ground truth UMAP of the hXin query dataset. (**C**) UMAPs of the hXin query showing CellAssign-predicted annotations using different marker gene sets. (**D**) UMAPs comparing CellAssign annotations from different marker gene sets with ground truth.

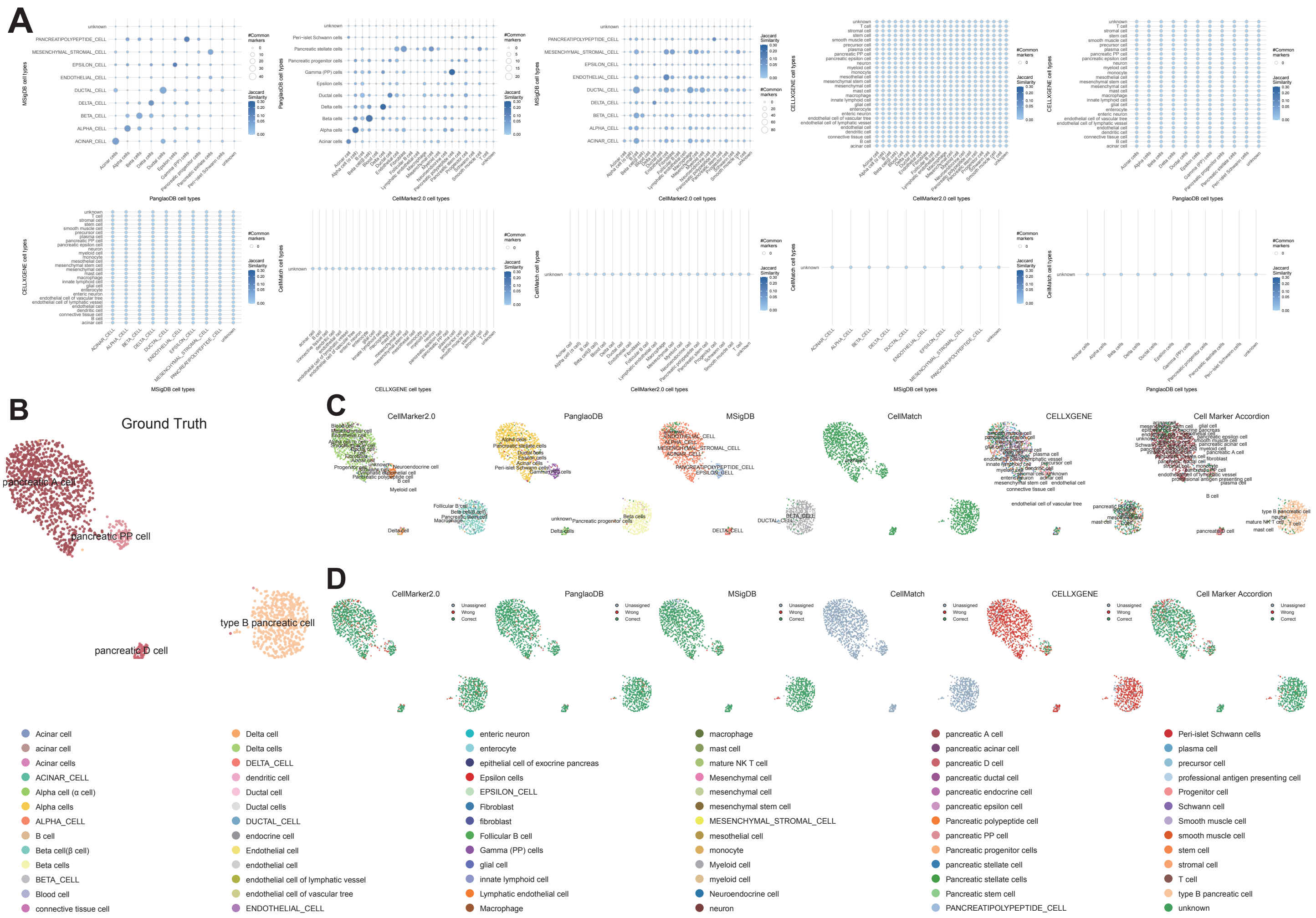

Supplementary Figure S36. (**A**) Overlap of marker genes across databases for hXin cell types annotated by SCINA with different marker resources, in which dot color encodes the Jaccard similarity and dot size indicates the number of shared markers for each cell-type pair. (**B**) Ground truth UMAP of the hXin query dataset. (**C**) UMAPs of the hXin query showing SCINA-predicted annotations using different marker gene sets. (**D**) UMAPs comparing SCINA annotations from different marker gene sets with ground truth.

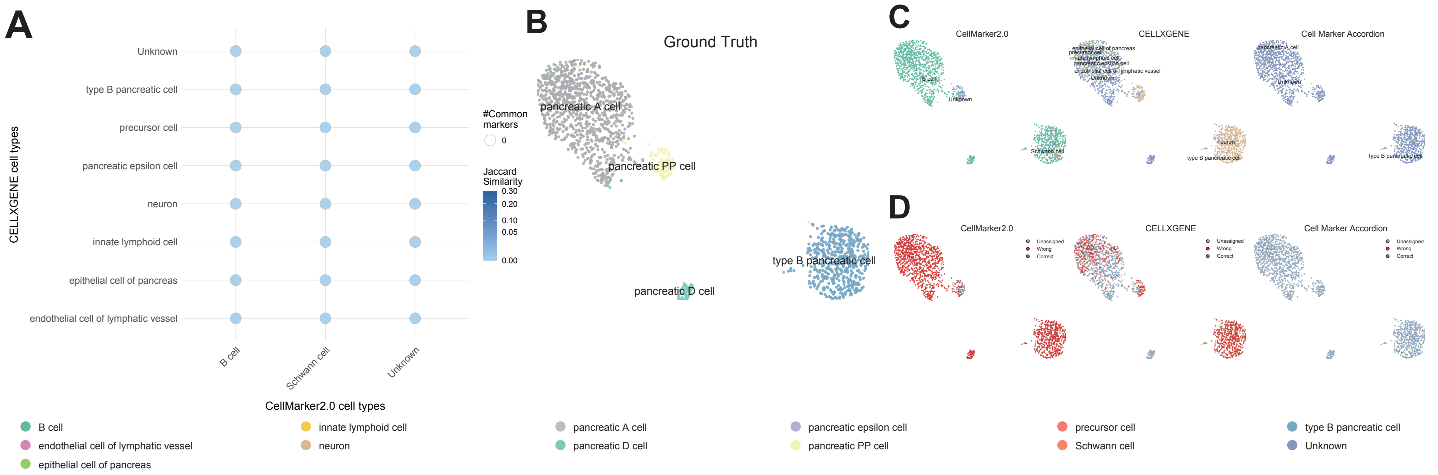

Supplementary Figure S37. (**A**) Overlap of marker genes across databases for hXin cell types annotated by Garnett with different marker resources, in which dot color encodes the Jaccard similarity and dot size indicates the number of shared markers for each cell-type pair. (**B**) Ground truth UMAP of the hXin query dataset. (**C**) UMAPs of the hXin query showing Garnett-predicted annotations using different marker gene sets. (**D**) UMAPs comparing Garnett annotations from different marker gene sets with ground truth.

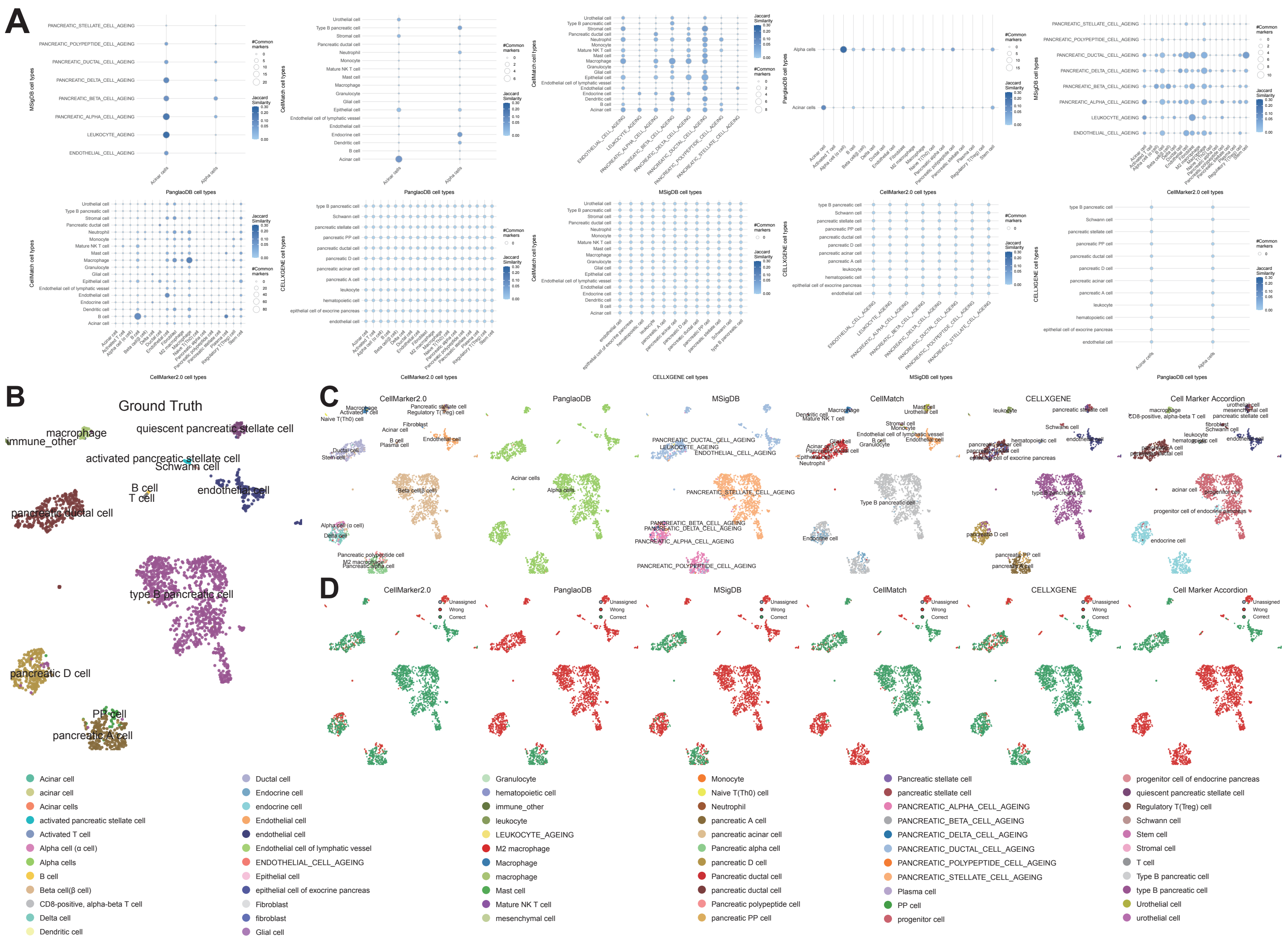

Supplementary Figure S38. (**A**) Overlap of marker genes across databases for mBaron cell types annotated by CellAssign with different marker resources, in which dot color encodes the Jaccard similarity and dot size indicates the number of shared markers for each cell-type pair. (**B**) Ground truth UMAP of the mBaron query dataset. (**C**) UMAPs of the mBaron query showing CellAssign-predicted annotations using different marker gene sets. (**D**) UMAPs comparing CellAssign annotations from different marker gene sets with ground truth.

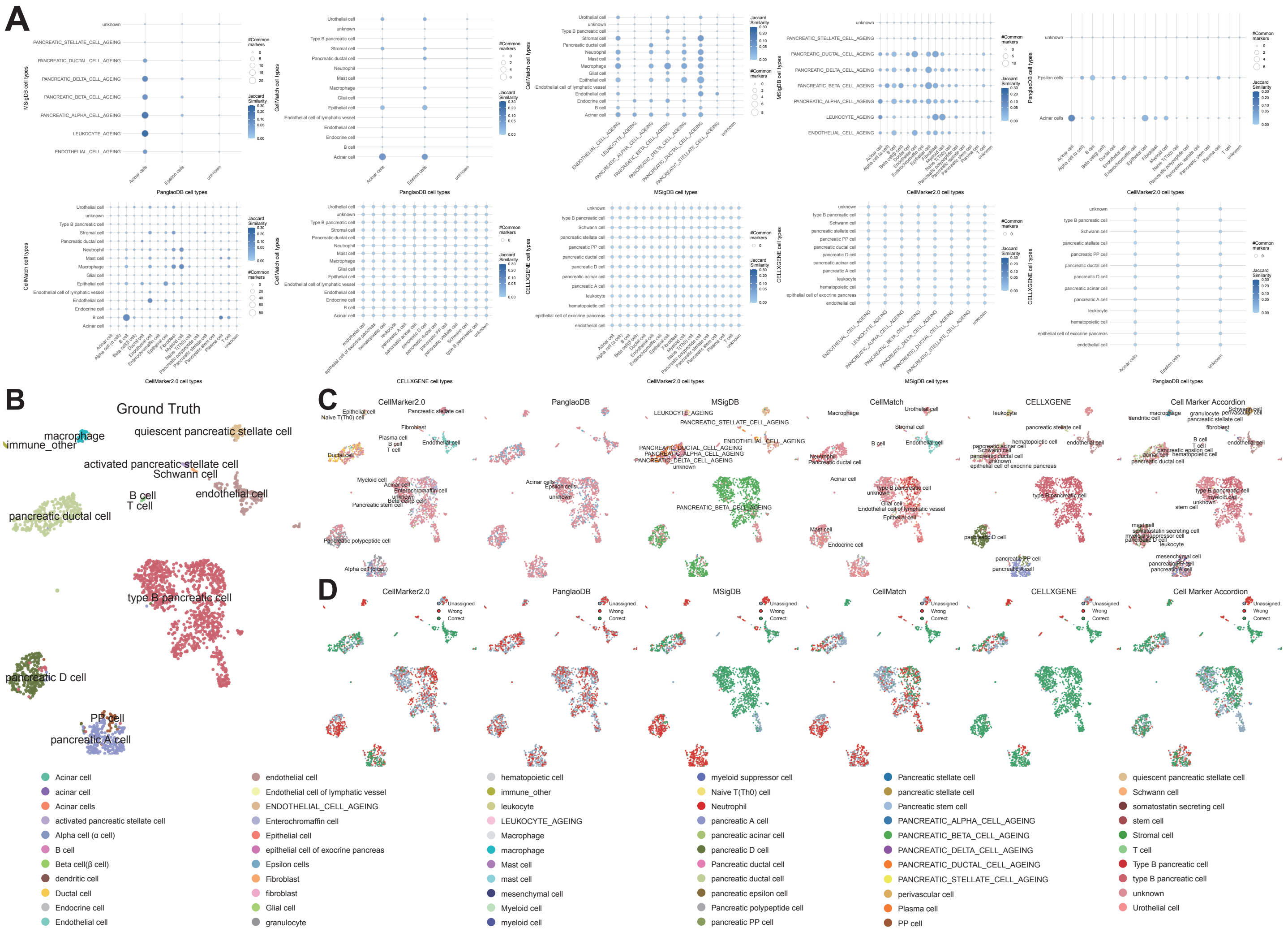

Supplementary Figure S39. (**A**) Overlap of marker genes across databases for mBaron cell types annotated by SCINA with different marker resources, in which dot color encodes the Jaccard similarity and dot size indicates the number of shared markers for each cell-type pair. (**B**) Ground truth UMAP of the mBaron query dataset. (**C**) UMAPs of the mBaron query showing SCINA-predicted annotations using different marker gene sets. (**D**) UMAPs comparing SCINA annotations from different marker gene sets with ground truth.

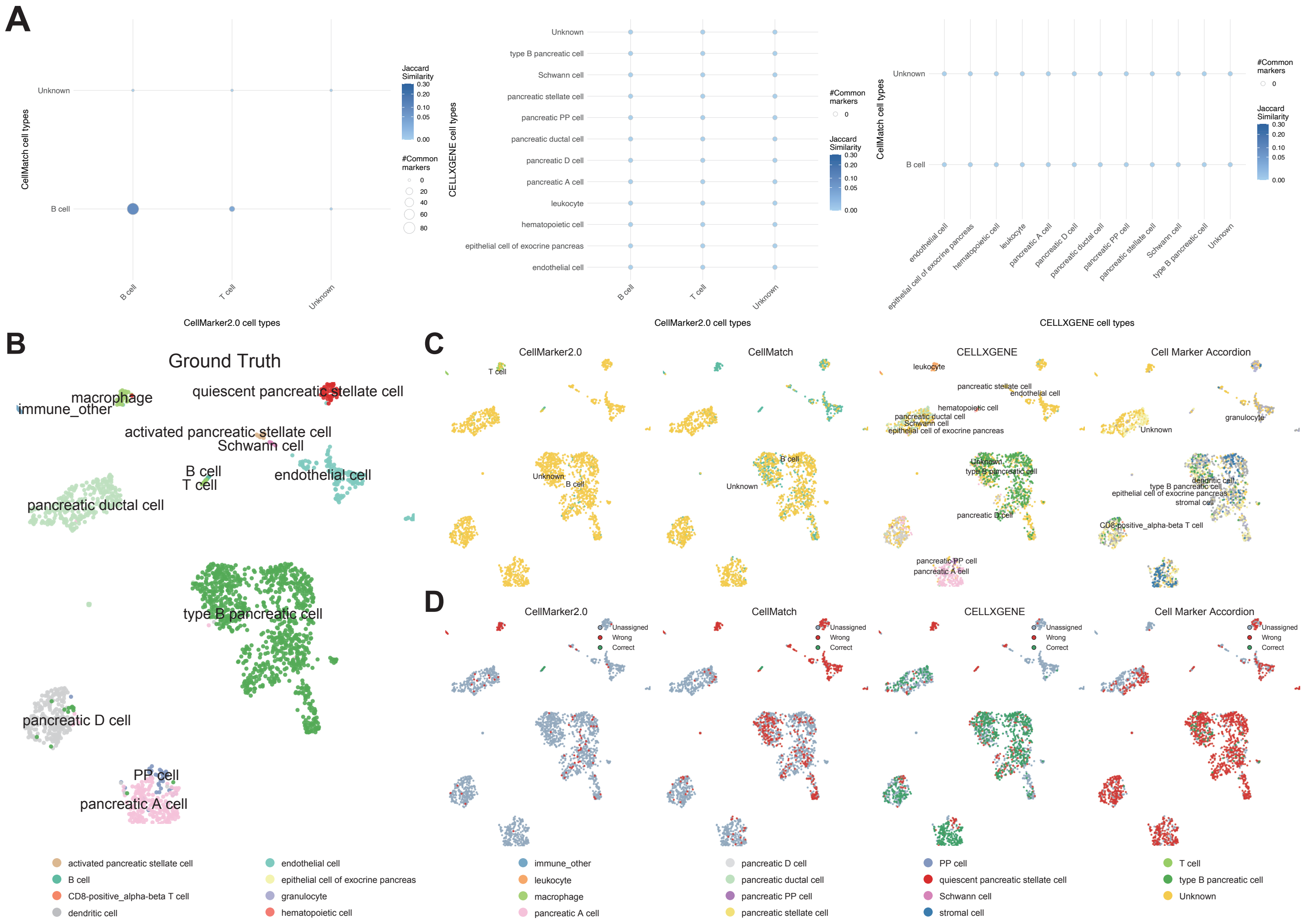

Supplementary Figure S40. (**A**) Overlap of marker genes across databases for mBaron cell types annotated by Garnett with different marker resources, in which dot color encodes the Jaccard similarity and dot size indicates the number of shared markers for each cell-type pair. (**B**) Ground truth UMAP of the mBaron query dataset. (**C**) UMAPs of the mBaron query showing Garnett-predicted annotations using different marker gene sets. (**D**) UMAPs comparing Garnett annotations from different marker gene sets with ground truth.

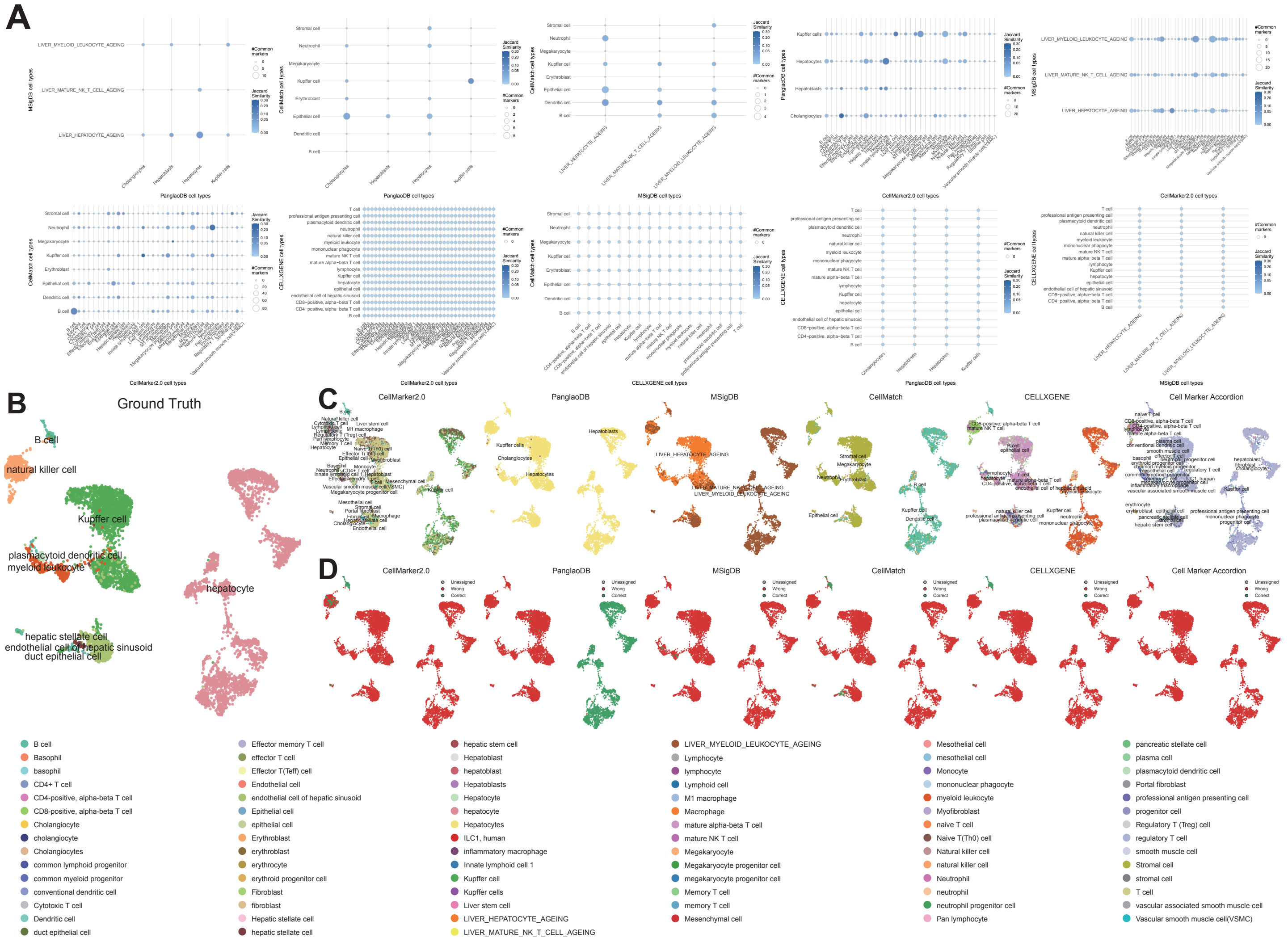

Supplementary Figure S41. (**A**) Overlap of marker genes across databases for TM_Liver cell types annotated by CellAssign with different marker resources, in which dot color encodes the Jaccard similarity and dot size indicates the number of shared markers for each cell-type pair. (**B**) Ground truth UMAP of the TM_Liver query dataset. (**C**) UMAPs of the TM_Liver query showing CellAssign-predicted annotations using different marker gene sets. (**D**) UMAPs comparing CellAssign annotations from different marker gene sets with ground truth.

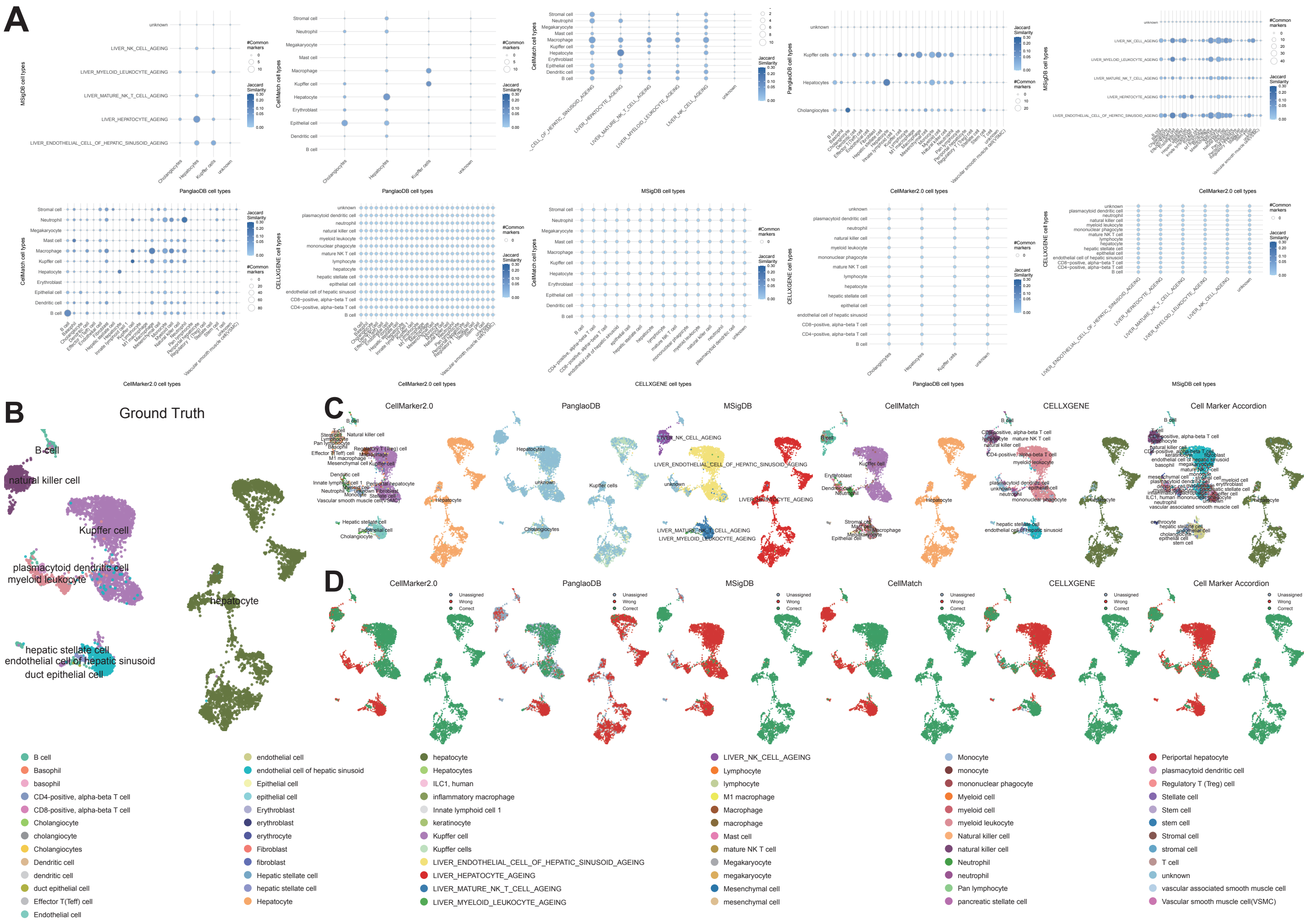

Supplementary Figure S42. (**A**) Overlap of marker genes across databases for TM_Liver cell types annotated by SCINA with different marker resources, in which dot color encodes the Jaccard similarity and dot size indicates the number of shared markers for each cell-type pair. (**B**) Ground truth UMAP of the TM_Liver query dataset. (**C**) UMAPs of the TM_Liver query showing SCINA-predicted annotations using different marker gene sets. (**D**) UMAPs comparing SCINA annotations from different marker gene sets with ground truth.

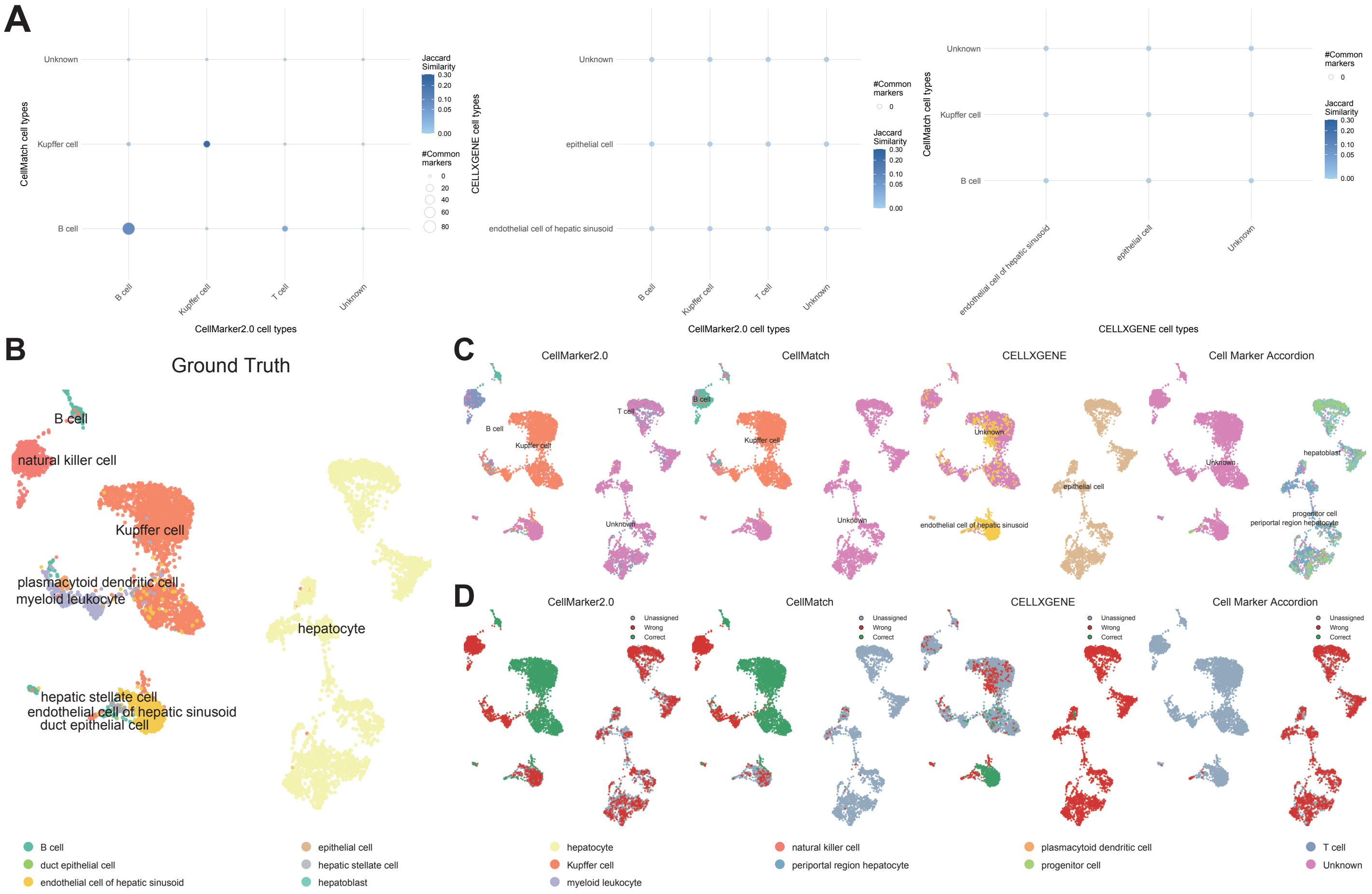

Supplementary Figure S43. (**A**) Overlap of marker genes across databases for TM_Liver cell types annotated by Garnett with different marker resources, in which dot color encodes the Jaccard similarity and dot size indicates the number of shared markers for each cell-type pair. (**B**) Ground truth UMAP of the TM_Liver query dataset. (**C**) UMAPs of the TM_Liver query showing Garnett-predicted annotations using different marker gene sets. (**D**) UMAPs comparing Garnett annotations from different marker gene sets with ground truth.

Supplementary Table S10. Datasets for Marker Benchmarking

Datasets	#celltype	#cell	#gene	Tissue	Species	Condition	Protocol	Reference
hBaron	14	8569	18343	pancreas	human	health	inDrop	[21]
hMuraro	10	2126	16494	pancreas	human	health	CEL-seq2	[22]
hSegerstolpe	10	2122	21795	pancreas	human	health	Smart-seq2	[23]
hXin	4	1600	28255	pancreas	human	health	SMARTer	[24]
mBaron	13	1886	13491	pancreas	mouse	health	inDrop	[21]
TM_Liver	9	7294	17984	liver	mouse	health	10x	[26]

Supplementary Table S11. Method Performance Across Marker Database

	method	hBaron	hMuraro	hSegerstolpe	hXin	mBaron	TM_Liver
Acc	CellAssign	0.1614	0.2183	0.5495	0.3975	0.2370	0.0000
	CellAssign_PanglaoDB	0.8351	0.9064	0.9826	0.9966	0.1007	0.3998
	CellAssign_CellMarker2.0	0.7039	0.7691	0.1857	0.0034	0.8489	0.0208
	CellAssign_MSigDB	0.3210	0.9299	0.9656	0.9960	0.2291	0.0016
	CellAssign_CELLXGENE	0.3157	0.5503	0.6065	0.0570	0.8325	0.0158
	CellAssign_CellMatch	0.0292	0.1308	0.4632	0.0784	0.6872	0.0093
	Garnett	0.1845	0.0000	0.0391	0.0067	0.0255	0.0000
	Garnett_CellMarker2.0	0.0000	0.0000	0.0000	0.0000	0.0074	0.3617
	Garnett_CellMatch					0.0053	0.3581
	Garnett_CELLXGENE	0.0098	0.0005	0.0019	0.0007		0.0925
	SCINA	0.1528	0.4365	0.7121	0.7909	0.4226	0.5313
	SCINA_PanglaoDB	0.7925	0.8688	0.9331	0.9330	0.0000	0.1067
	SCINA_CellMarker2.0	0.4106	0.7352	0.7719	0.8445	0.2036	0.7582
	SCINA_MSigDB	0.1894	0.4812	0.7526	0.9464	0.5228	0.4788
	SCINA_CELLXGENE	0.0263	0.0922	0.0429	0.0214	0.7625	0.5510
	SCINA_CellMatch	0.0000	0.0000	0.0000	0.0000	0.1607	0.7534
F1-score	CellAssign	0.1614	0.2183	0.5495	0.3975	0.2370	0.0000
	CellAssign_PanglaoDB	0.8351	0.9064	0.9826	0.9966	0.1007	0.3998
	CellAssign_CellMarker2.0	0.7039	0.7691	0.1857	0.0034	0.8489	0.0208
	CellAssign_MSigDB	0.3210	0.9299	0.9656	0.9960	0.2291	0.0016
	CellAssign_CELLXGENE	0.3157	0.5503	0.6065	0.0570	0.8325	0.0158
	CellAssign_CellMatch	0.0292	0.1308	0.4632	0.0784	0.6872	0.0093
	Garnett	0.1845	0.0000	0.0391	0.0067	0.0255	0.0000
	Garnett_CellMarker2.0	0.0011	0.0000	0.0000	0.0000	0.0074	0.3617
	Garnett_CellMatch					0.0053	0.3581
	Garnett_CELLXGENE	0.0098	0.0005	0.0019	0.0007		0.0925
	SCINA	0.1528	0.4365	0.7121	0.7909	0.4226	0.5313
	SCINA_PanglaoDB	0.7925	0.8688	0.9331	0.9330	0.0000	0.1067
	SCINA_CellMarker2.0	0.4106	0.7352	0.7719	0.8445	0.2036	0.7582
	SCINA_MSigDB	0.1894	0.4812	0.7526	0.9464	0.5228	0.4788
	SCINA_CELLXGENE	0.0263	0.0922	0.0429	0.0214	0.7625	0.5510
	SCINA_CellMatch	0.0000	0.0000	0.0000	0.0000	0.1607	0.7534
MCC	CellAssign	0.2002	0.2674	0.6293	0.5089	0.2933	−0.0021
	CellAssign_PanglaoDB	0.8015	0.8825	0.9771	0.9939	−0.0057	0.0045
	CellAssign_CellMarker2.0	0.6631	0.7219	0.2542	0.0538	0.7902	−0.1179
	CellAssign_MSigDB	0.3623	0.9118	0.9554	0.9926	0.2401	−0.3805
	CellAssign_CELLXGENE	0.3508	0.5014	0.5514	0.0982	0.7749	−0.0144
	CellAssign_CellMatch	−0.1201	0.1605	0.2270	0.0936	0.5657	−0.0122
	Garnett	0.2478	0.0000	0.0787	0.0451	0.0098	0.0000
	Garnett_CellMarker2.0	0.0017	0.0000	0.0000	0.0000	0.0244	0.3092
	Garnett_CellMatch					0.0073	0.3385
	Garnett_CELLXGENE	0.0066	0.0009	0.0016	0.0008		0.1212
	SCINA	0.1806	0.4261	0.6745	0.6877	0.4145	0.4439
	SCINA_PanglaoDB	0.7540	0.8379	0.9152	0.8844	0.0000	−0.0583
	SCINA_CellMarker2.0	0.3901	0.6867	0.7222	0.7539	0.2402	0.6795
	SCINA_MSigDB	0.2591	0.4793	0.7186	0.9074	0.3250	0.4001
	SCINA_CELLXGENE	0.0322	0.0921	0.0417	0.0235	0.6985	0.5098
	SCINA_CellMatch	0.0000	0.0000	0.0000	0.0000	0.1731	0.6661
NMI	CellAssign	0.7514	0.7355	0.9338	0.8466	0.8000	0.3451
	CellAssign_PanglaoDB	0.8463	0.8676	0.9647	0.9874	0.0031	0.0095
	CellAssign_CellMarker2.0	0.8285	0.8761	0.6631	0.0239	0.7677	0.3907
	CellAssign_MSigDB	0.6892	0.9037	0.9762	0.9764	0.6040	0.4591
	CellAssign_CELLXGENE	0.8584	0.8653	0.8880	0.6539	0.8554	0.5246
	CellAssign_CellMatch	0.0918	0.1779	0.1536	0.0917	0.6663	0.3667
	Garnett	0.2431	0.0151	0.0968	0.0121	0.1726	0.2323
	Garnett_CellMarker2.0	0.2019	0.0737	0.3486	0.1692	0.1137	0.5010
	Garnett_CellMatch					0.1387	0.6441
	Garnett_CELLXGENE	0.3115	0.2105	0.4159	0.4207		0.6659
	SCINA	0.3932	0.5500	0.6873	0.5592	0.5043	0.7123
	SCINA_PanglaoDB	0.7955	0.8369	0.8979	0.8109	0.0135	0.1204
	SCINA_CellMarker2.0	0.5587	0.7693	0.7950	0.6203	0.4456	0.7817

	SCINA_MSigDB	0.4504	0.6257	0.7443	0.8514	0.4357	0.8396
	SCINA_CELLXGENE	0.4232	0.5073	0.5595	0.1249	0.7810	0.7355
	SCINA_CellMatch	0.0000	0.0000	0.0000	0.0000	0.3640	0.7769
ARI	CellAssign	0.5575	0.6509	0.9395	0.8544	0.8176	0.1737
	CellAssign_PanglaoDB	0.8937	0.9109	0.9823	0.9920	0.0011	0.0039
	CellAssign_CellMarker2.0	0.8254	0.9176	0.4389	0.0105	0.7937	0.3884
	CellAssign_MSigDB	0.4423	0.9414	0.9848	0.9904	0.7216	0.5227
	CellAssign_CELLXGENE	0.9090	0.9151	0.9089	0.6039	0.9007	0.5692
	CellAssign_CellMatch	0.0364	0.1387	0.1113	0.0755	0.5839	0.4454
	Garnett	0.0452	0.0046	−0.0124	0.0015	0.0921	0.0194
	Garnett_CellMarker2.0	0.1650	0.0317	0.3350	0.1323	0.0298	0.5107
	Garnett_CellMatch					0.0464	0.6949
	Garnett_CELLXGENE	0.2812	0.1210	0.2131	0.3909		0.6872
	SCINA	0.1718	0.3442	0.5718	0.6553	0.3007	0.7984
	SCINA_PanglaoDB	0.8593	0.8914	0.9222	0.8903	0.0270	0.0896
	SCINA_CellMarker2.0	0.3434	0.8157	0.8309	0.7342	0.2871	0.9018
	SCINA_MSigDB	0.2275	0.4130	0.6321	0.9068	0.3758	0.9103
	SCINA_CELLXGENE	0.2755	0.3339	0.5687	0.0665	0.8485	0.8353
	SCINA_CellMatch	0.0000	0.0000	0.0000	0.0000	0.1210	0.9221

9 Supplementary Note 9: Benchmarking Annotation Methods on Pathology Datasets

Pathology cohorts differ fundamentally from healthy atlases: lesions introduce abnormal and transitional states, reference coverage is often incomplete for disease-relevant populations, and platform/center effects amplify domain shift. Forcing diseased cells into healthy lineages can inflate apparent accuracy while degrading biological validity, especially when malignant programs or therapy-induced states are present. Robust annotation requires disease-aware references, explicit handling of Unknown classes, and transparent provenance of reference resources. To support pathology-centered single-cell analysis under these constraints, scSuperAnnotator aggregates a broad collection of disease datasets (curated, versioned, and context-tagged by tissue, indication, and health status) that can serve as either references or queries. Leveraging this resource, we performed disease-to-disease benchmarks of reference-based annotators under a unified pipeline and report quantitative and qualitative readouts to guide method selection in pathology settings. The detailed information of the datasets used is shown in Supplementary Table S12.

Across the combined evaluation in Supplementary Fig.S44A, ACTINN achieved the strongest overall performance, followed by scPred, CaSTLe, and singleCellNet, which also showed consistently high scores. SingleR and scClassify were intermediate. scDeepSort ranked lower overall, consistent with its label space being constrained to a limited set of tissue categories, which impaired annotation in several disease settings. The cross-dataset score distributions in Supplementary Fig.S44B support this ordering: top-performing methods maintain higher medians with tight dispersion across metrics, whereas lower-ranked approaches show lower central tendencies and greater variability.

To examine method behavior in context, we visualized predictions for a liver cancer query. The ground-truth labels (Supplementary Fig.S44C) and method predictions (Supplementary Fig. S44D) show that high-performing approaches such as ACTINN and singleCellNet closely reproduce the observed structure and preserve clear boundaries between malignant and epithelial compartments. In contrast, scmapcluster, scmapcell, and CHETAH leave a substantial fraction of cells unassigned, and scClassify sometimes assigns cells to intermediate hierarchical categories rather than terminal types, reducing cluster purity. These qualitative patterns align with the quantitative rankings.

Extending this analysis to nine additional disease datasets (Supplementary Figs S45–53) yielded the same qualitative ordering. ACTINN, scPred, and CaSTLe were the reliable across diseases, whereas nearest-neighbor mapping approaches (scmapcluster, scmapcell) and hierarchical schemes (scClassify, CHETAH) were less robust to disease-associated heterogeneity, typically producing higher unassigned rates or confusion among closely related subsets.

In summary, when disease datasets are used as references for independent disease queries, methods with strong discriminative capacity and without restrictive label spaces (exemplified by ACTINN, scPred, and CaSTLe) deliver more consistent annotation quality across metrics and cohorts. Approaches based on nearest-neighbor transfer or hierarchical taxonomies are more sensitive to disease heterogeneity, often increasing the proportion of unassigned labels and reducing cluster purity. These results provide practical guidance for method selection within scSuperAnnotator for pathology-focused single-cell annotation.

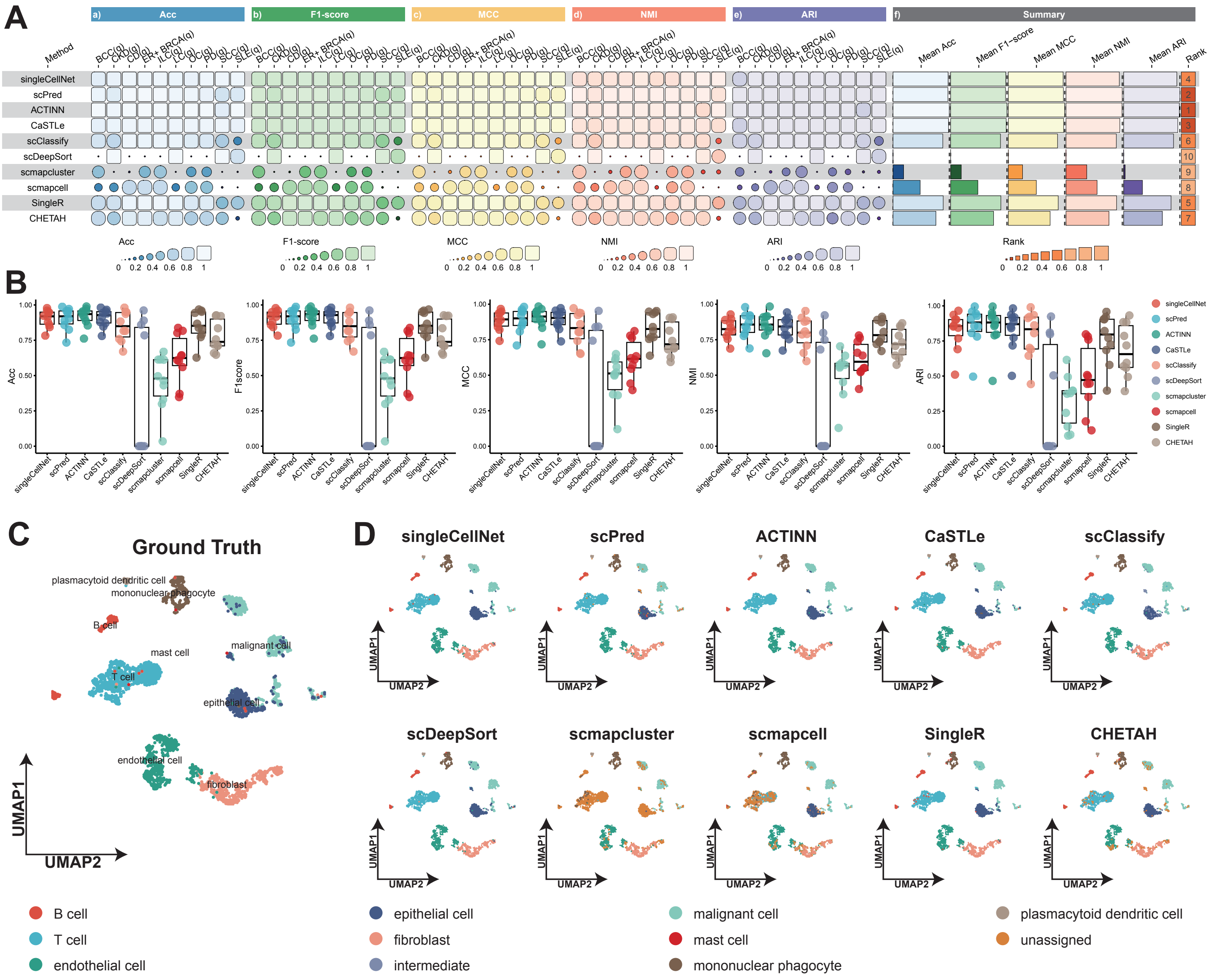

Supplementary Figure S44. (**A**) Summary of method performance on pathology datasets with per-dataset comparisons across five evaluation metrics for each method. (BCC: basel cell carcinoma; CKD: chronic kidney disease; CD: Crohn's disease; ER+ BRCA: estrogen receptor positive breast cancer; ILC: invasive lobular breast carcinoma; LC: liver cancer; OC: ovarian cancer; PD: periodontitis; SCC: squamous cell lung carcinoma; SLE: systemic lupus erythematosus.) (**B**) Distributional comparison of the five evaluation metrics for each method across pathology datasets. (**C**) Ground truth UMAP of the liver cancer dataset. (**D**) UMAPs showing method-specific annotations for the liver cancer dataset.

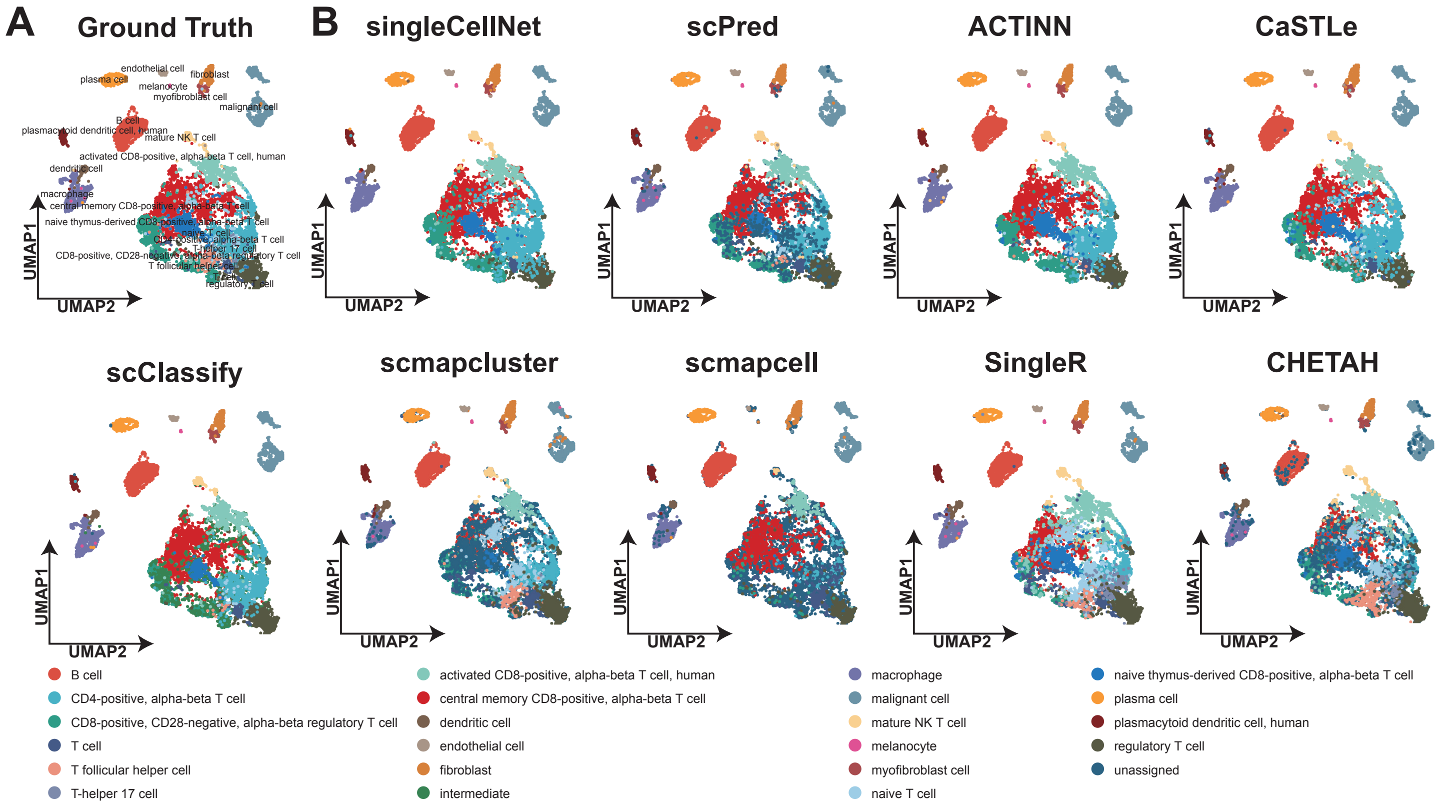

Supplementary Figure S45. (**A**) Ground truth UMAP of the basel cell carcinoma dataset. (**B**) UMAPs showing method-specific annotations for the basel cell carcinoma dataset.

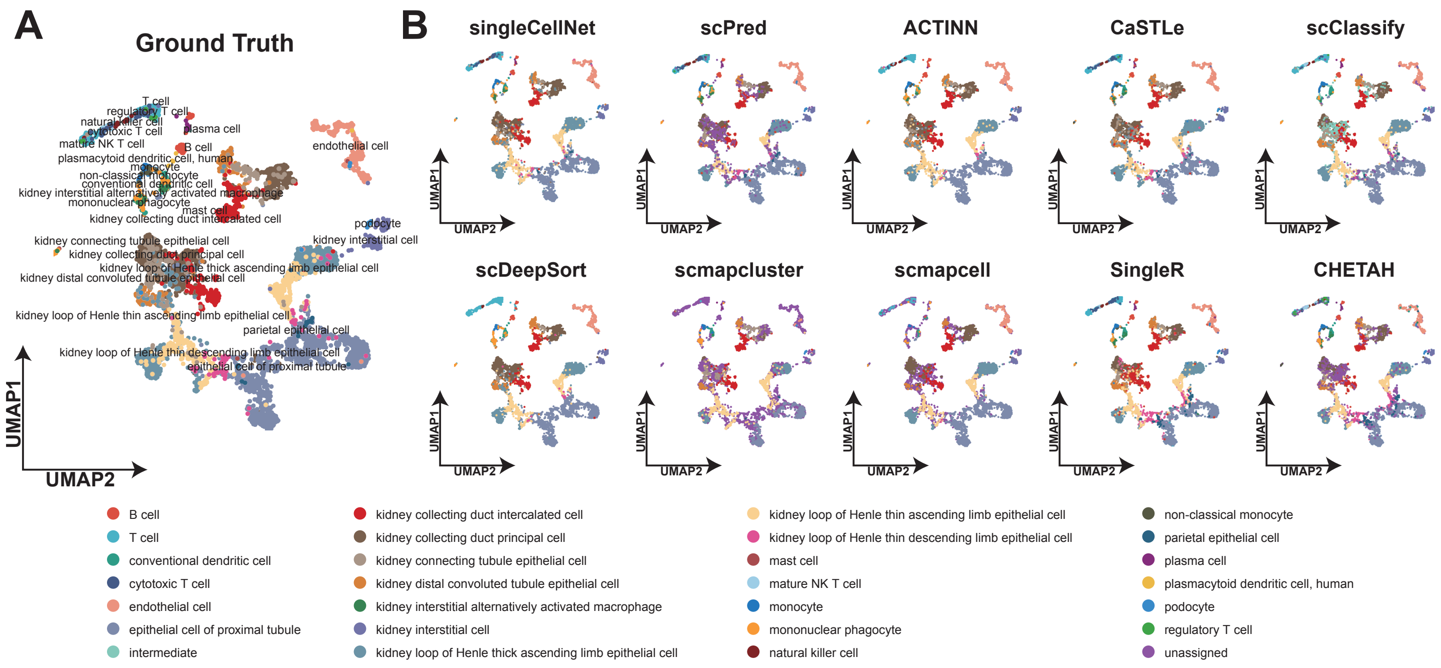

Supplementary Figure S46. (**A**) Ground truth UMAP of the chronic kidney disease dataset. (**B**) UMAPs showing method-specific annotations for the chronic kidney disease dataset.

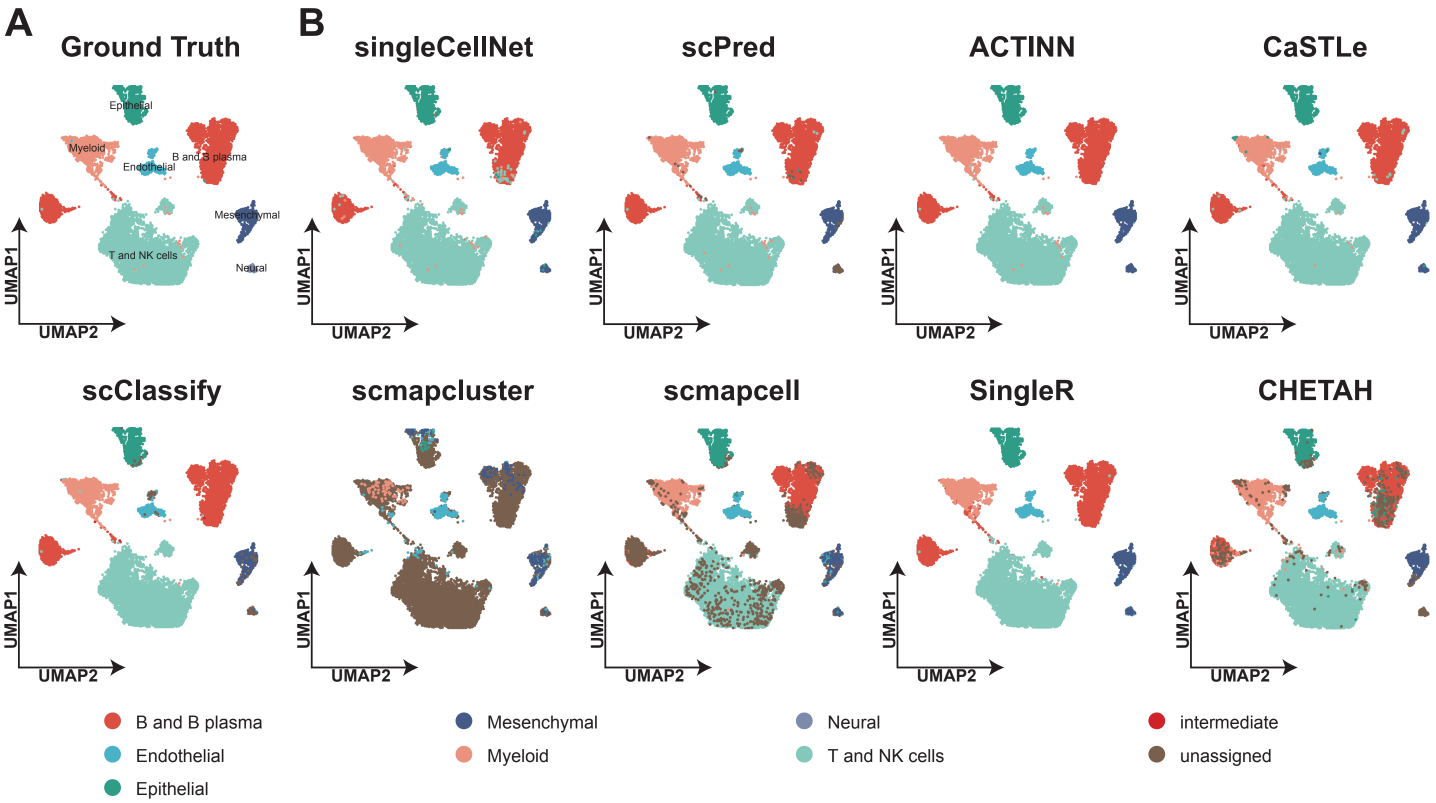

Supplementary Figure S47. (**A**) Ground truth UMAP of the Crohn's disease dataset. (**B**) UMAPs showing method-specific annotations for the Crohn's disease dataset.

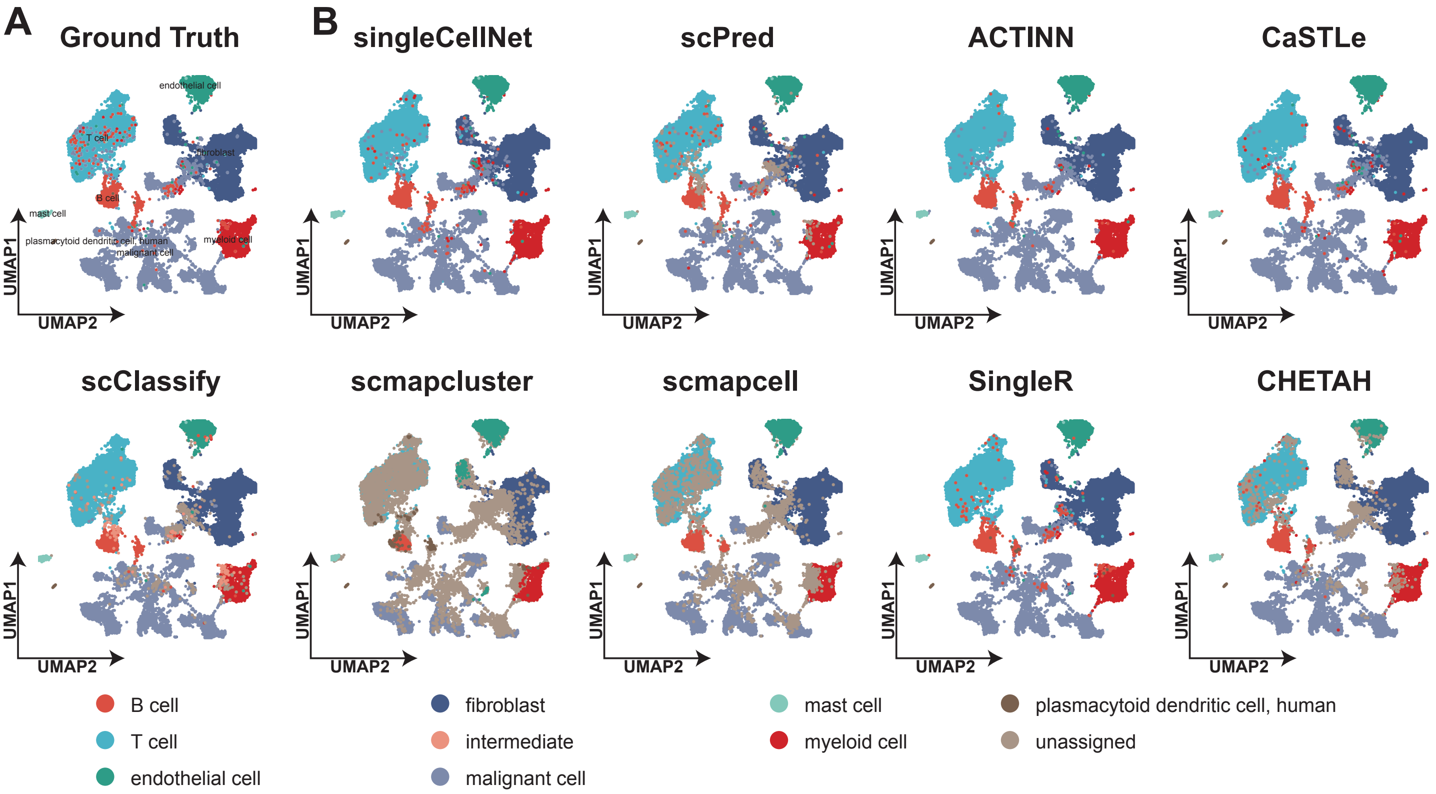

Supplementary Figure S48. (**A**) Ground truth UMAP of the estrogen receptor positive breast cancer dataset. (**B**) UMAPs showing method-specific annotations for the estrogen receptor positive breast cancer dataset.

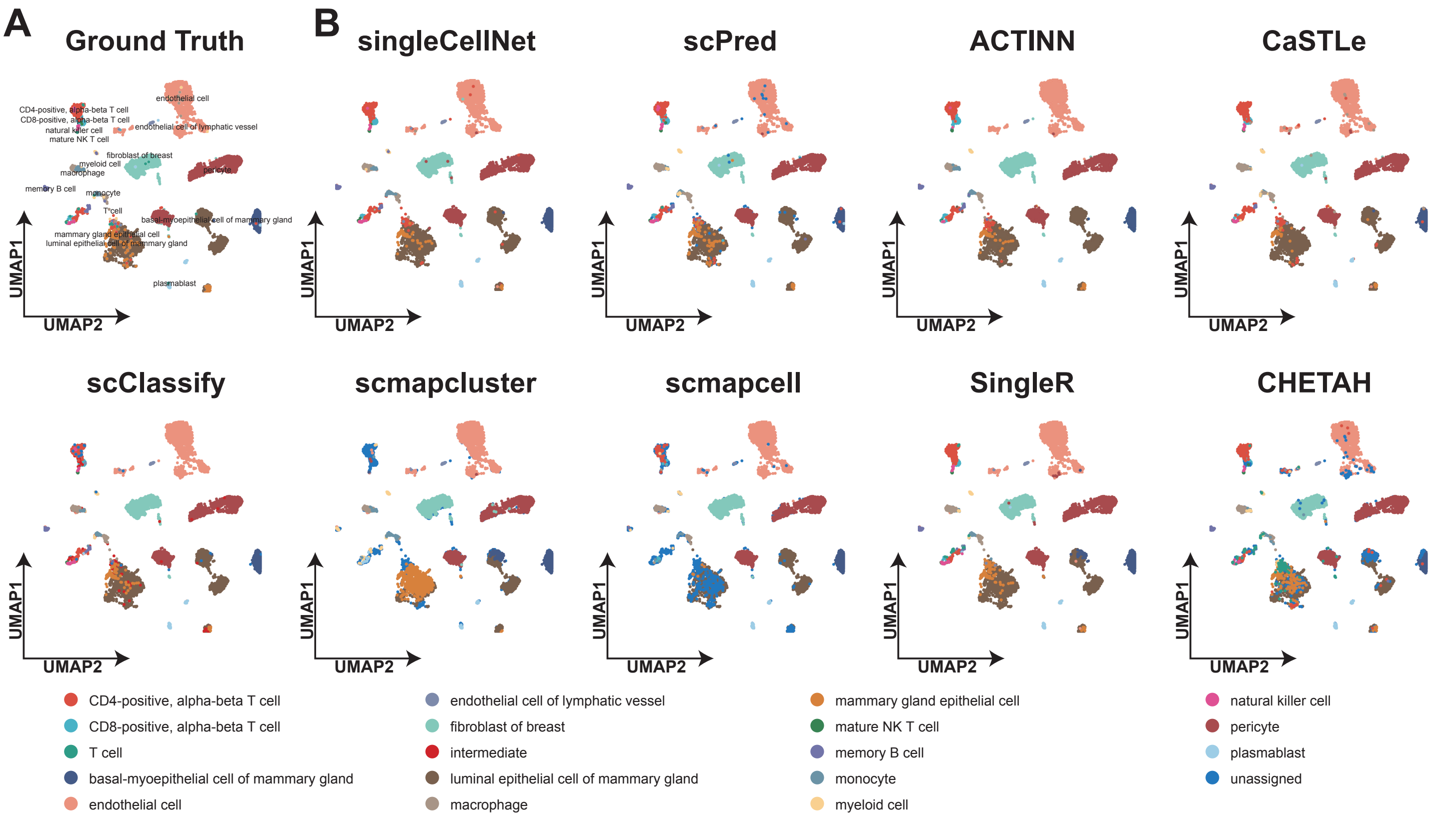

Supplementary Figure S49. (**A**) Ground truth UMAP of the invasive lobular breast carcinoma dataset. (**B**) UMAPs showing method-specific annotations for the invasive lobular breast carcinoma dataset.

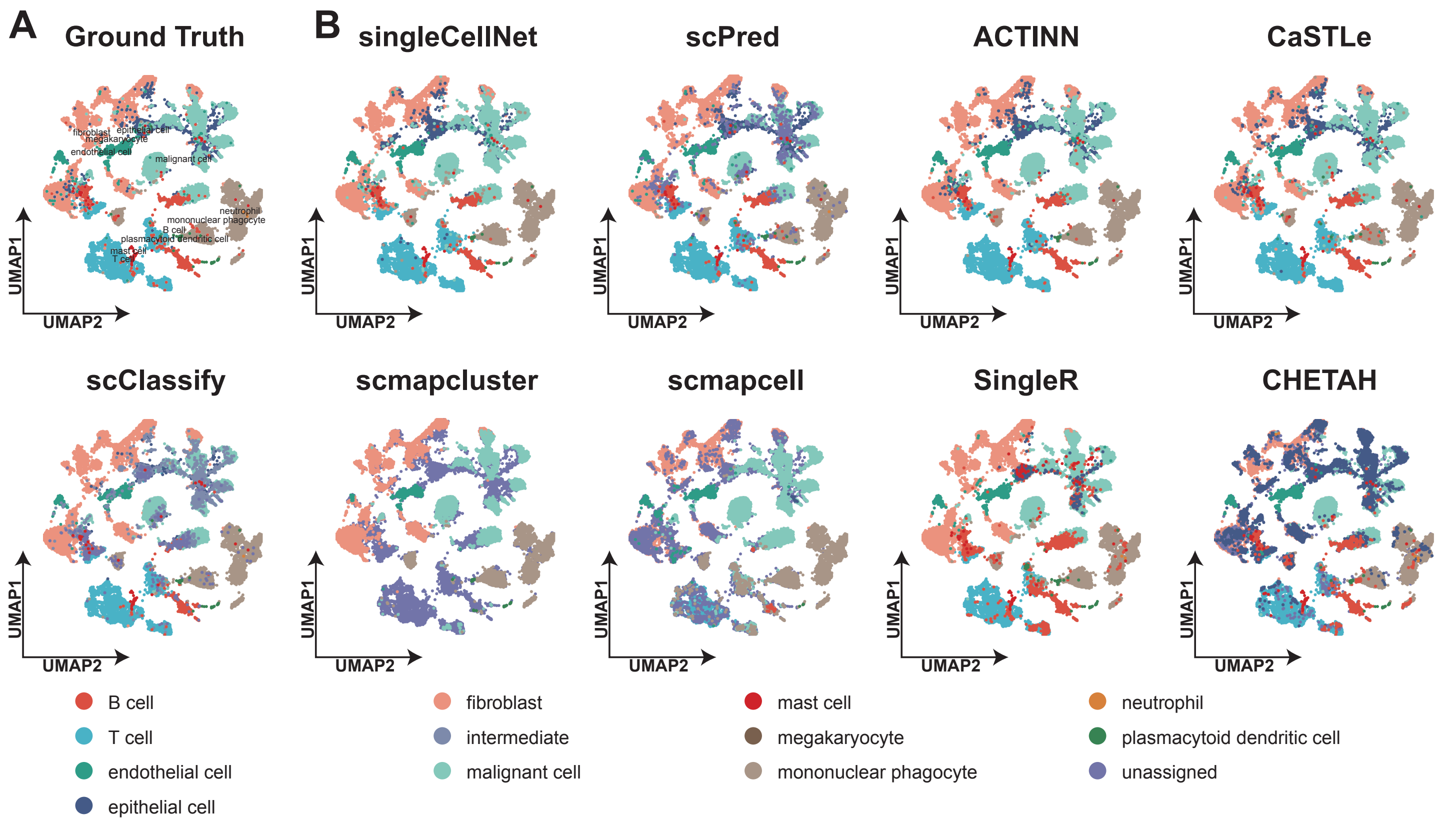

Supplementary Figure S50. (**A**) Ground truth UMAP of the ovarian cancer dataset. (**B**) UMAPs showing method-specific annotations for the ovarian cancer dataset.

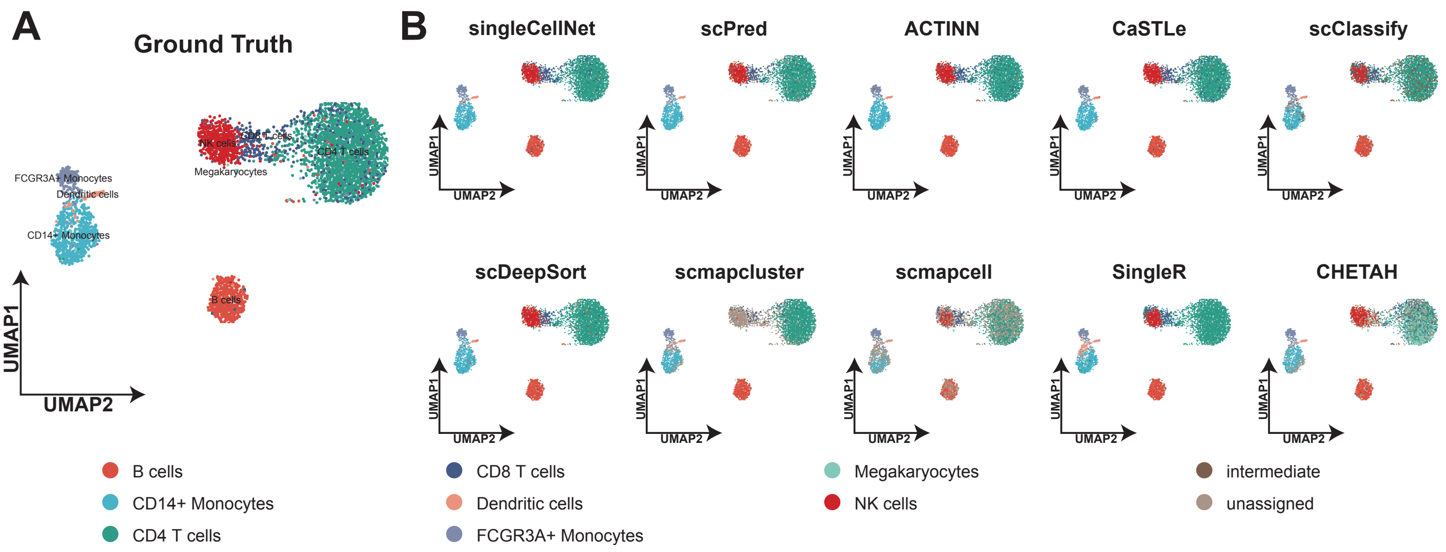

Supplementary Figure S51. (**A**) Ground truth UMAP of the systemic lupus erythematosus dataset. (**B**) UMAPs showing method-specific annotations for the systemic lupus erythematosus dataset.

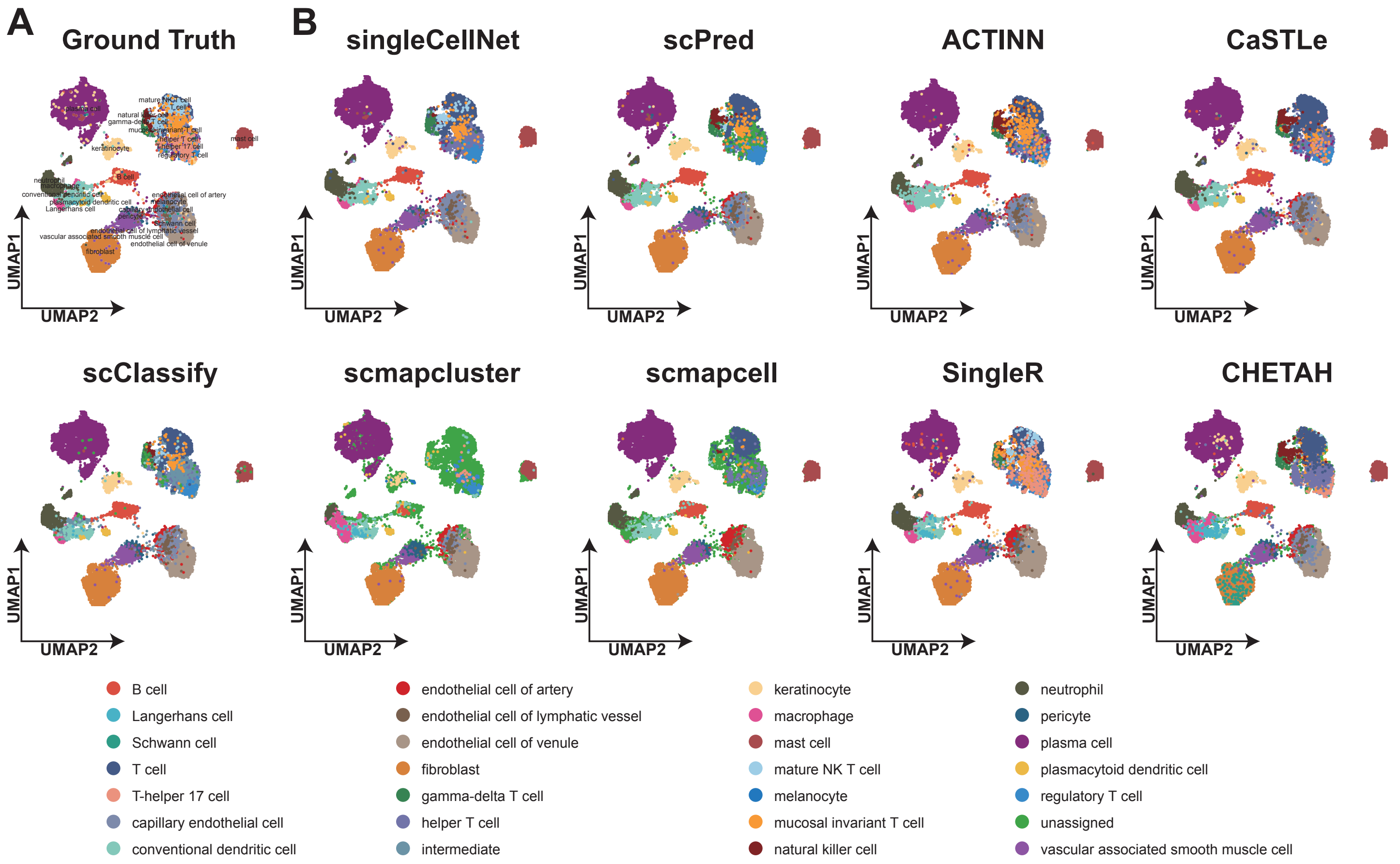

Supplementary Figure S52. (**A**) Ground truth UMAP of the periodontitis dataset. (**B**) UMAPs showing method-specific annotations for the periodontitis dataset.

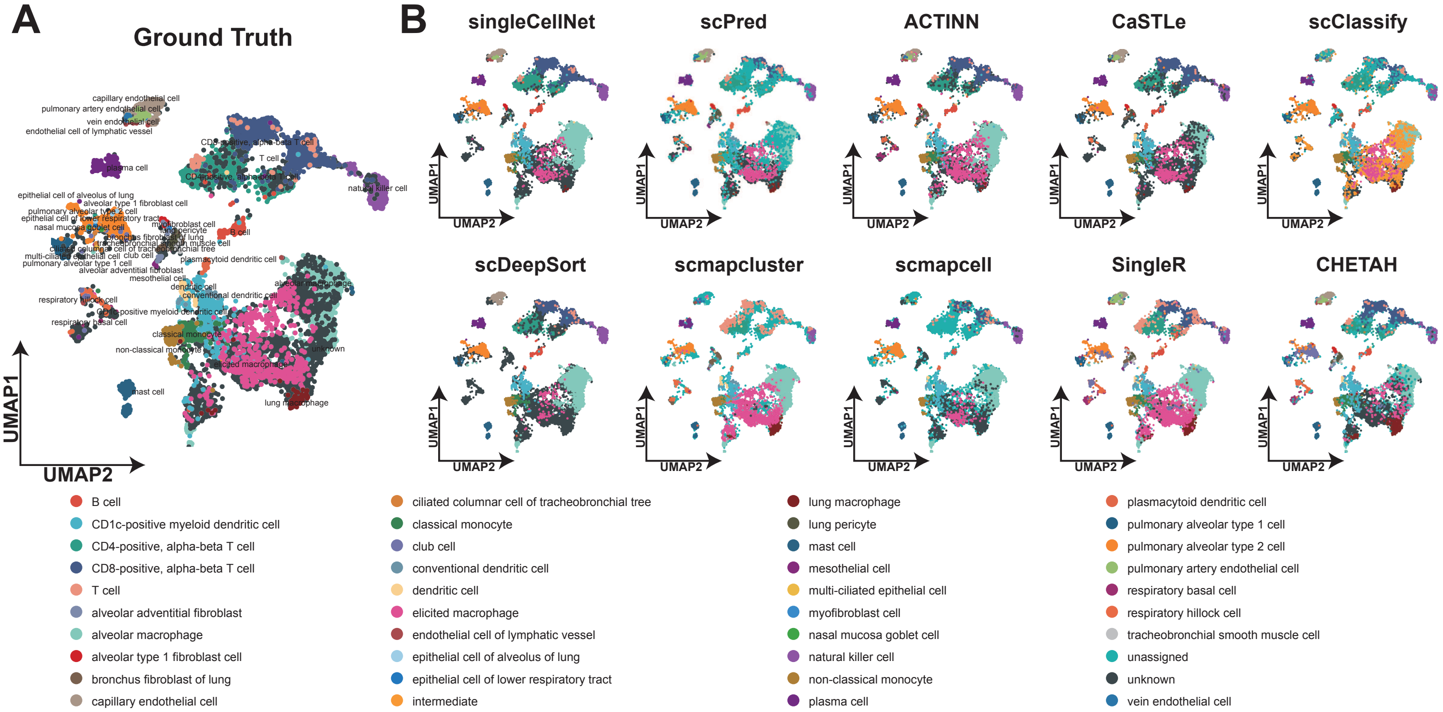

Supplementary Figure S53. (**A**) Ground truth UMAP of the squamous cell lung carcinoma dataset. (**B**) UMAPs showing method-specific annotations for the squamous cell lung carcinoma dataset.

Supplementary Table S12. Datasets for Pathology Benchmarking

Datasets	#celltype	#cell	#gene	Tissue	Species	Condition	Protocol	Reference
BCC(ref)	21	9180	22781	skin	human	disease	10x	[27]
BCC(query)	21	9180	22781	skin	human	disease	10x	[27]
CKD(ref)	26	5185	27235	kidney	human	disease	10x	[28]
CKD(query)	26	5186	27235	kidney	human	disease	10x	[28]
CD(ref)	6	19018	18442	intestine	human	disease	10x	[16]
CD(query)	7	14089	18442	intestine	human	disease	10x	[16]
ER+ BRCA(ref)	8	21912	22781	breast	human	disease	10x	[27]
ER+ BRCA(query)	8	21912	22781	brease	human	disease	10x	[27]
ILC(ref)	17	6285	29067	breast	human	disease	10x	[29]
ILC(query)	17	6285	29067	breast	human	disease	10x	[29]
LC(ref)	9	3330	45454	liver	human	disease	10x	[18]
LC(query)	9	3331	45454	liver	human	disease	10x	[18]
OC(ref)	11	28891	45454	ovary	human	disease	10x	[18]
OC(query)	11	28892	45454	ovary	human	disease	10x	[18]
PD(ref)	26	19260	36298	gingiva	human	disease	10x	[30]
PD(query)	26	19260	36298	gingiva	human	disease	10x	[30]
SCC(ref)	36	10315	56239	lung	human	disease	10x	[17]
SCC(query)	38	10316	56239	lung	human	disease	10x	[17]
SLE(ref)	8	3637	32738	blood	human	disease	10x	[31]
SLE(query)	8	4217	32738	blood	human	disease	10x	[31]

Supplementary Table S13. Method Performance on Pathology Bechmarks

	method	BCC	CKD	CD	ER+ BRCA	ILC	LC	OC	PD	SCC	SLE
Acc	singleCellNet	0.8587	0.8427	0.9778	0.9604	0.9473	0.9496	0.9163	0.8784	0.7826	0.9223
	scPred	0.8888	0.8255	0.9875	0.9717	0.9572	0.9577	0.9367	0.8579	0.7338	0.9020
	ACTINN	0.9072	0.8544	0.9891	0.9740	0.9545	0.9586	0.9447	0.8843	0.7617	0.9261
	CaSTLe	0.8704	0.8463	0.9816	0.9663	0.9442	0.9553	0.9399	0.8788	0.7803	0.9165
	scClassify	0.8161	0.7594	0.9810	0.9516	0.9367	0.9496	0.8833	0.8162	0.6700	0.7431
	scDeepSort	0.0000	0.8596	0.0000	0.0000	0.0000	0.9610	0.0000	0.0000	0.7851	0.8895
	scmapcluster	0.6122	0.2993	0.0346	0.6140	0.6691	0.4308	0.4956	0.4647	0.3301	0.6130
	scmapcell	0.3709	0.5600	0.8170	0.8379	0.8019	0.6443	0.6398	0.6031	0.3477	0.6085
	SingleR	0.7722	0.7912	0.9679	0.9599	0.9434	0.9517	0.8671	0.8277	0.6271	0.8376
	CHETAH	0.7563	0.7113	0.9269	0.9241	0.8353	0.9216	0.7211	0.7098	0.6474	0.6589
F1-score	singleCellNet	0.8587	0.8427	0.9778	0.9604	0.9473	0.9496	0.9163	0.8784	0.7826	0.9223
	scPred	0.8888	0.8255	0.9875	0.9717	0.9572	0.9577	0.9367	0.8579	0.7338	0.9020
	ACTINN	0.9072	0.8544	0.9891	0.9740	0.9545	0.9586	0.9447	0.8843	0.7617	0.9261
	CaSTLe	0.8704	0.8463	0.9816	0.9663	0.9442	0.9553	0.9399	0.8788	0.7803	0.9165
	scClassify	0.8161	0.7594	0.9810	0.9516	0.9367	0.9496	0.8833	0.8162	0.6700	0.7431
	scDeepSort	0.0000	0.8596	0.0000	0.0000	0.0000	0.9610	0.0000	0.0000	0.7851	0.8895
	scampcluster	0.6122	0.2993	0.0346	0.6140	0.6691	0.4308	0.4956	0.4647	0.3301	0.6130
	scmapcell	0.3709	0.5600	0.8170	0.8379	0.8019	0.6443	0.6398	0.6031	0.3477	0.6085
	SingleR	0.7722	0.7912	0.9679	0.9599	0.9434	0.9517	0.8671	0.8277	0.6271	0.8376
	CHETAH	0.7563	0.7113	0.9269	0.9241	0.8353	0.9216	0.7211	0.7098	0.6474	0.6589
MCC	singleCellNet	0.8393	0.8198	0.9671	0.9481	0.9360	0.9391	0.8966	0.8653	0.7416	0.8893
	scPred	0.8777	0.8081	0.9815	0.9632	0.9484	0.9492	0.9231	0.8465	0.7098	0.8620
	ACTINN	0.8946	0.8333	0.9840	0.9660	0.9448	0.9500	0.9318	0.8717	0.7173	0.8947
	CaSTLe	0.8526	0.8242	0.9728	0.9558	0.9322	0.9460	0.9259	0.8655	0.7380	0.8809
	scClassify	0.8029	0.7416	0.9719	0.9377	0.9236	0.9395	0.8603	0.8036	0.6501	0.6547
	scDeepSort	0.0000	0.8395	0.0000	0.0000	0.0000	0.9530	0.0000	0.0000	0.7431	0.8423
	scampcluster	0.6184	0.3652	0.1203	0.6050	0.6489	0.4971	0.5191	0.5056	0.3594	0.5584
	scmapcell	0.4414	0.5714	0.7634	0.8109	0.7784	0.6338	0.6101	0.6210	0.3955	0.5451
	SingleR	0.7548	0.7684	0.9525	0.9477	0.9313	0.9417	0.8398	0.8112	0.6374	0.7679
	CHETAH	0.7419	0.6887	0.8955	0.9044	0.8137	0.9077	0.6819	0.6940	0.6255	0.5928
NMI	singleCellNet	0.7763	0.7550	0.9285	0.8678	0.8956	0.8892	0.8060	0.8355	0.6874	0.8137
	scPred	0.8423	0.7765	0.9797	0.9145	0.9206	0.9216	0.8716	0.8270	0.6888	0.7928
	ACTINN	0.8331	0.7803	0.9841	0.9089	0.9120	0.9159	0.8698	0.8434	0.6548	0.8133
	CaSTLe	0.7835	0.7535	0.9425	0.8848	0.8930	0.9051	0.8558	0.8301	0.6739	0.7835
	scClassify	0.7871	0.7088	0.9530	0.8761	0.8839	0.9053	0.8086	0.7900	0.6570	0.5992
	scDeepSort	0.0000	0.7985	0.0000	0.0000	0.0000	0.9222	0.0000	0.0000	0.6809	0.7488
	scampcluster	0.6375	0.3660	0.1288	0.5733	0.7038	0.5874	0.5535	0.5624	0.4512	0.5734
	scmapcell	0.5287	0.5511	0.7369	0.7621	0.7800	0.6389	0.5492	0.6580	0.4029	0.5214
	SingleR	0.7330	0.7372	0.9116	0.8730	0.8897	0.8971	0.7591	0.8056	0.6702	0.7192
	CHETAH	0.7060	0.6644	0.8482	0.8382	0.7784	0.8666	0.6182	0.7317	0.6389	0.5734
ARI	singleCellNet	0.6915	0.7689	0.9652	0.9030	0.9364	0.9009	0.8441	0.8526	0.5098	0.8591
	scPred	0.8063	0.7813	0.9957	0.9451	0.9588	0.9284	0.9075	0.8525	0.4962	0.8568
	ACTINN	0.7965	0.7900	0.9954	0.9353	0.9498	0.9191	0.8983	0.8650	0.4654	0.8559
	CaSTLe	0.7178	0.7730	0.9824	0.9184	0.9372	0.9154	0.8864	0.8532	0.5015	0.8412
	scClassify	0.7009	0.6900	0.9905	0.9133	0.9346	0.9226	0.8621	0.8053	0.4428	0.6447
	scDeepSort	0.0000	0.8097	0.0000	0.0000	0.0000	0.9244	0.0000	0.0000	0.5047	0.7980
	scampcluster	0.3738	0.0748	0.0823	0.3962	0.6150	0.3777	0.3944	0.2375	0.1405	0.4932
	scmapcell	0.1771	0.3507	0.7820	0.7596	0.7976	0.4976	0.5074	0.4435	0.1132	0.4459
	SingleR	0.5792	0.6847	0.9582	0.9050	0.9215	0.9039	0.7711	0.8212	0.3961	0.7256
	CHETAH	0.5586	0.5942	0.9318	0.8836	0.7860	0.8841	0.5697	0.7183	0.3921	0.4937

10 Supplementary Note 10: Benchmarking of scTPC and scDFN with Cell Population Identification Methods

To strengthen our platform's coverage of upstream clustering paradigms that impact downstream annotation quality, we selected scTPC and scDFN for focused comparison.

- scTPC ([7]) is a semi-supervised deep clustering method for single-cell RNA sequencing data. It couples constraint-based metric learning, which encodes prior cell relationships as triplet or pairwise constraints, with a denoising autoencoder pretrained under a zero-inflated negative binomial (ZINB) model to address the extreme sparsity and class imbalance typical of single-cell data. By injecting weak biological priors through these constraints and optimizing the ZINB objective, scTPC improves clustering accuracy and robustness on sparse single-cell datasets.

- scDFN ([8]) is a deep fusion network for clustering that integrates attribute learning and topological learning through a dual encoder design. One encoder models cell-wise feature representations from gene expression, while a second, graph-based encoder captures cell–cell topology. The two representations are optimized jointly under multiple complementary objectives. This dual encoder, multi-loss formulation preserves complex intercellular structure and delivers state-of-the-art clustering accuracy and stability on scRNA seq datasets.

To evaluate the effectiveness of scTPC and scDFN, we used a unified benchmarking framework to systematically compare them against 14 representative cell type annotation tools. Performance was assessed on four publicly available, well-annotated healthy pancreatic scRNA seq datasets: hMuraro (22), hSegerstolpe (23), hBaron (21), and Tabula Sapiens Pancreas (20). For the reference-based methods in the benchmark, hMuraro, hSegerstolpe, and Tabula Sapiens Pancreas were annotated using hBaron as the reference, while hBaron was annotated using Fasolino (25) as the reference.
To comprehensively assess clustering performance, we used two standard metrics: NMI and ARI. Supplementary Fig. S54A summarizes results across the four datasets. scTPC achieves, or comes very close to, the best scores on both metrics; scDFN typically ranks in the upper tier, and in several datasets, these two methods form the leading group. By contrast, supervised methods show greater cross-dataset variability, with occasional sharp drops not seen for the clustering approaches, consistent with sensitivity to reference-to-query mismatch. Overall, without relying on labeled references, scTPC and scDFN deliver competitive clustering performance and exhibit greater stability across datasets.
To complement the quantitative results, we performed a unified qualitative assessment across the four datasets using UMAP visualizations, confusion matrix heatmaps comparing true and predicted labels, and Sankey plots tracing correspondences from true labels to predicted labels. In Supplementary Fig. S54B for the hBaron dataset, scTPC produces compact, contiguous, well-separated clusters whose geometry closely follows the ground truth layout. scDFN yields similarly coherent manifolds but systematically partitions large populations into finer, internally consistent subclusters. The Sankey plots in Supplementary Fig. S55 corroborate these patterns: for scTPC, cells from a given ground truth type predominantly map to a single predicted cluster, whereas for scDFN, high abundance types such as pancreatic A cells split across two or more clusters, reflecting the finer granularity visible in the UMAP visualizations.
In contrast, several reference-based annotation methods show within-cluster inconsistency. For example, the type B pancreatic region in CaSTLe and the pancreatic A region in scClassify display marked heterogeneity, consistent with many-to-one and one-to-many flows in the Sankey plots. These qualitative observations align with the trends in Supplementary Fig. S54A. By preserving local boundaries and limiting cross-cluster contamination, the clustering approaches of scTPC and scDFN deliver higher and more stable clustering quality. From an annotation standpoint, scDFN does not attach semantic labels and therefore requires downstream labeling, and the labels produced by scTPC show only limited concordance with canonical ground truth. Accordingly, the confusion matrix heatmaps in Supplementary Fig. S56 show no pronounced diagonal for scTPC and scDFN, whereas most reference-based methods show a clear diagonal. CellAssign, Garnett, and scCATCH are exceptions to this pattern.
Extending the analysis to the remaining three datasets in Supplementary Figs S57–62 shows a consistent pattern. scTPC again reconstructs clusters that closely track the ground truth, with concentrated flows in the Sankey diagrams. scDFN preserves coherent structure while subdividing large populations into finer, internally consistent subclusters. Reference-based annotation methods vary by dataset and by the chosen reference; because assignments depend on that choice, label maps can differ across references, and within-cluster consistency is often lower than for the clustering approaches. This dependence manifests as occasional merging and the presence of off-type cells within clusters on the UMAPs, as increased off-diagonal mass in the confusion matrix heatmaps, and as greater branching in the Sankey plots when the reference is not well matched. Nevertheless, when judged by semantic label accuracy, reference-based methods typically yield higher annotation concordance and overall label quality than scTPC and scDFN.
Overall, the comparative evidence shows that scTPC emphasizes boundary preservation with minimal merging, scDFN emphasizes fine-grained yet pure segmentation, and reference-based annotation methods deliver more accurate semantic labels when the reference and query are well matched. A practical advantage of scTPC and scDFN is that neither relies on external reference atlases or predefined marker gene sets to guide clustering. This independence from external labels makes them broadly applicable across biological conditions, improving utility for novel or poorly characterized samples. Although these algorithms do not themselves assign explicit cell type names, the accurate and stable clusters they produce provide

a strong foundation for downstream cell type annotation. In settings where reference data or well-established markers are limited, such reliable de novo clustering is particularly valuable for interpreting new single cell datasets.

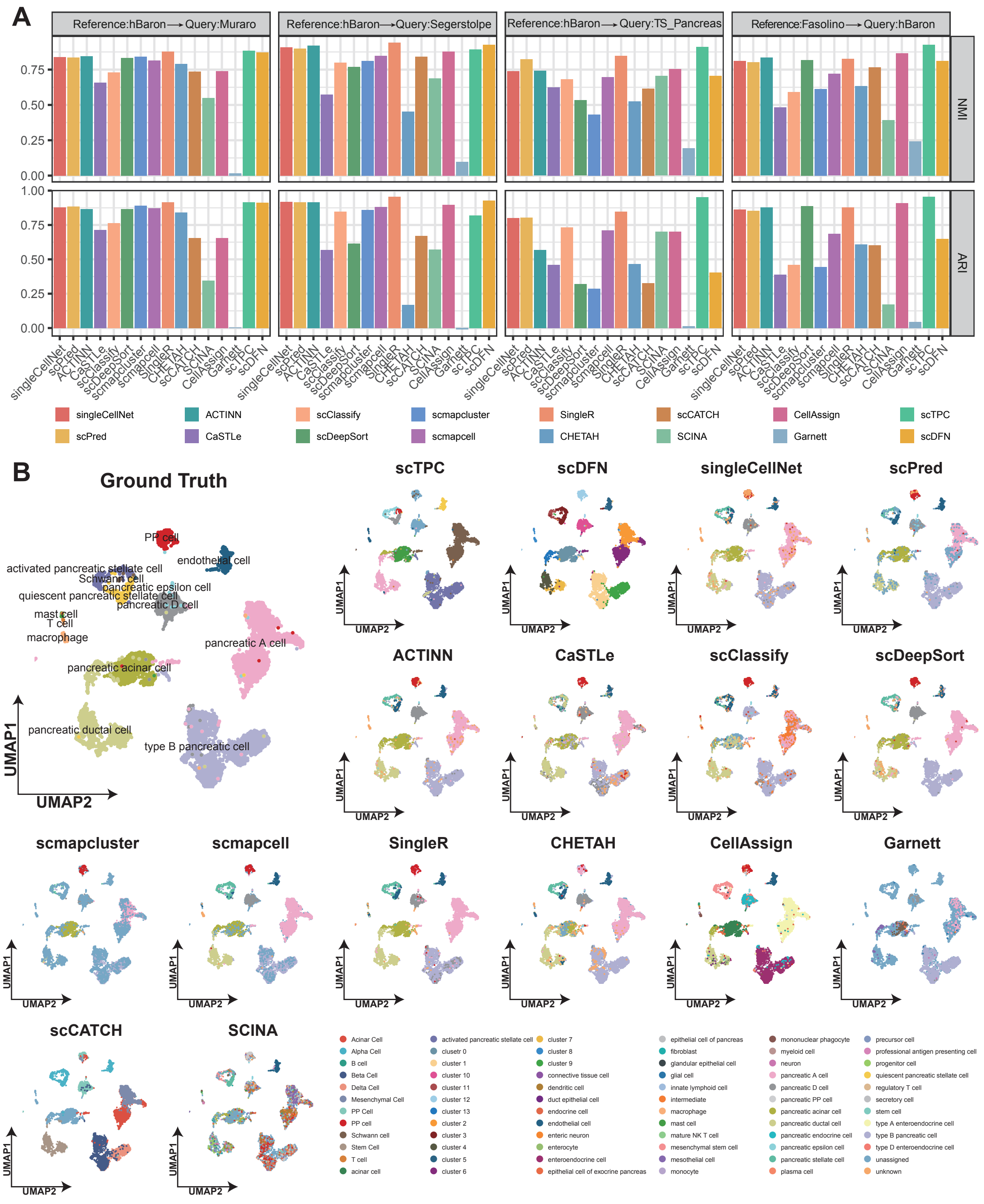

Supplementary Figure S54. (**A**) Comparison of NMI and ARI scores for scTPC and scDFN versus representative reference-based annotation methods in scSuperAnnotator across four healthy pancreatic scRNA-seq datasets (hMuraro, hSegerstolpe, Tabula Sapiens Pancreas, and hBaron). (**B**) UMAP of the hBaron dataset comparing ground truth, scTPC, scDFN, and reference-based annotation methods used for comparison, with the Fasolino dataset serving as the reference for the reference-based annotation methods.

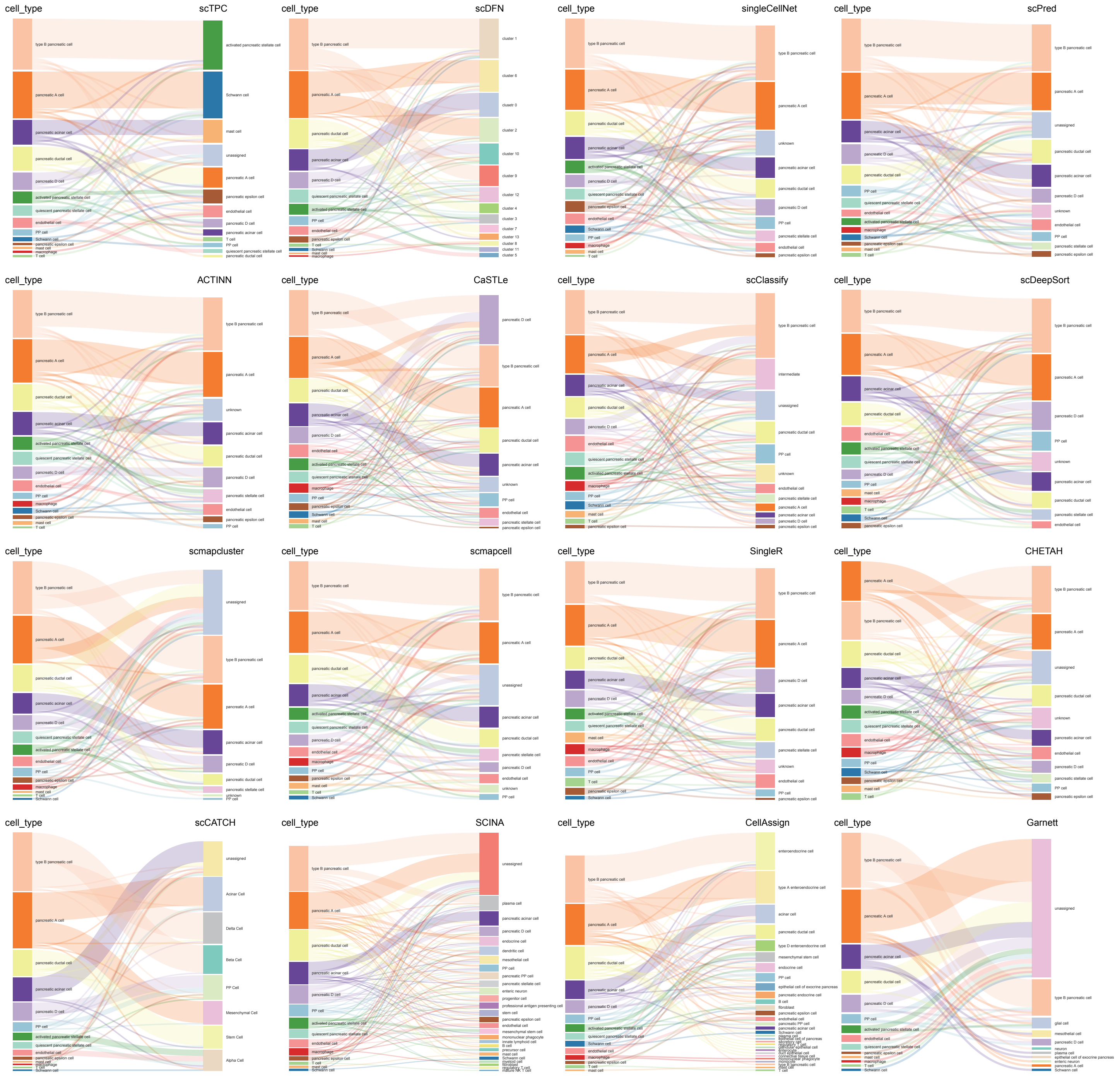

Supplementary Figure S55. Sankey plot illustrating the correspondence between the ground truth of the hBaron dataset and the annotation results from each method, with the Fasolino dataset serving as the reference for the reference-based annotation methods.

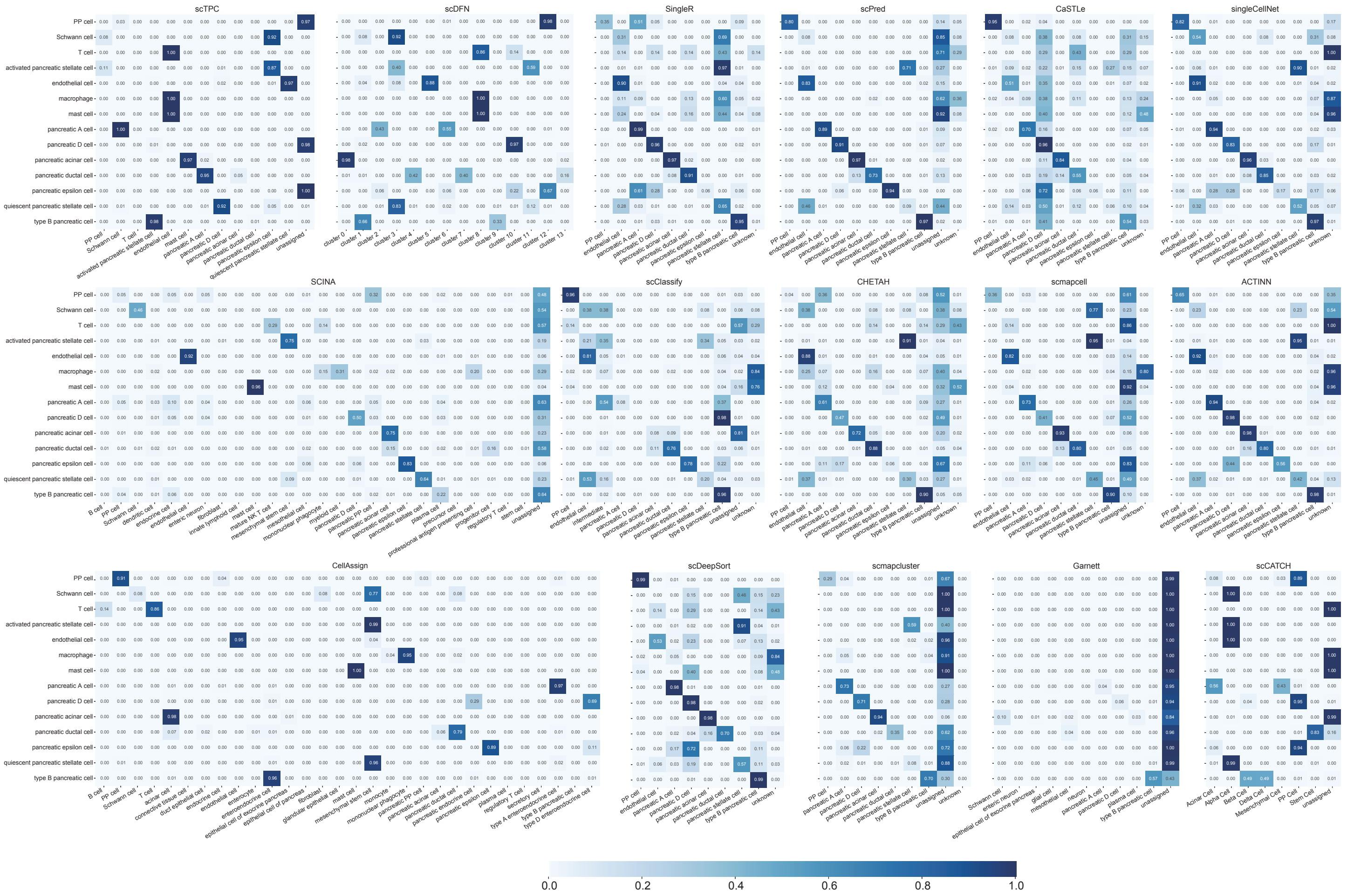

Supplementary Figure S56. Heatmap showing correlations between the ground truth of the hBaron dataset and the annotation results from each method, with the Fasolino dataset serving as the reference for the reference-based annotation methods. The y-axis represents the ground truth of the query dataset, and the x-axis represents the annotations produced by each method.

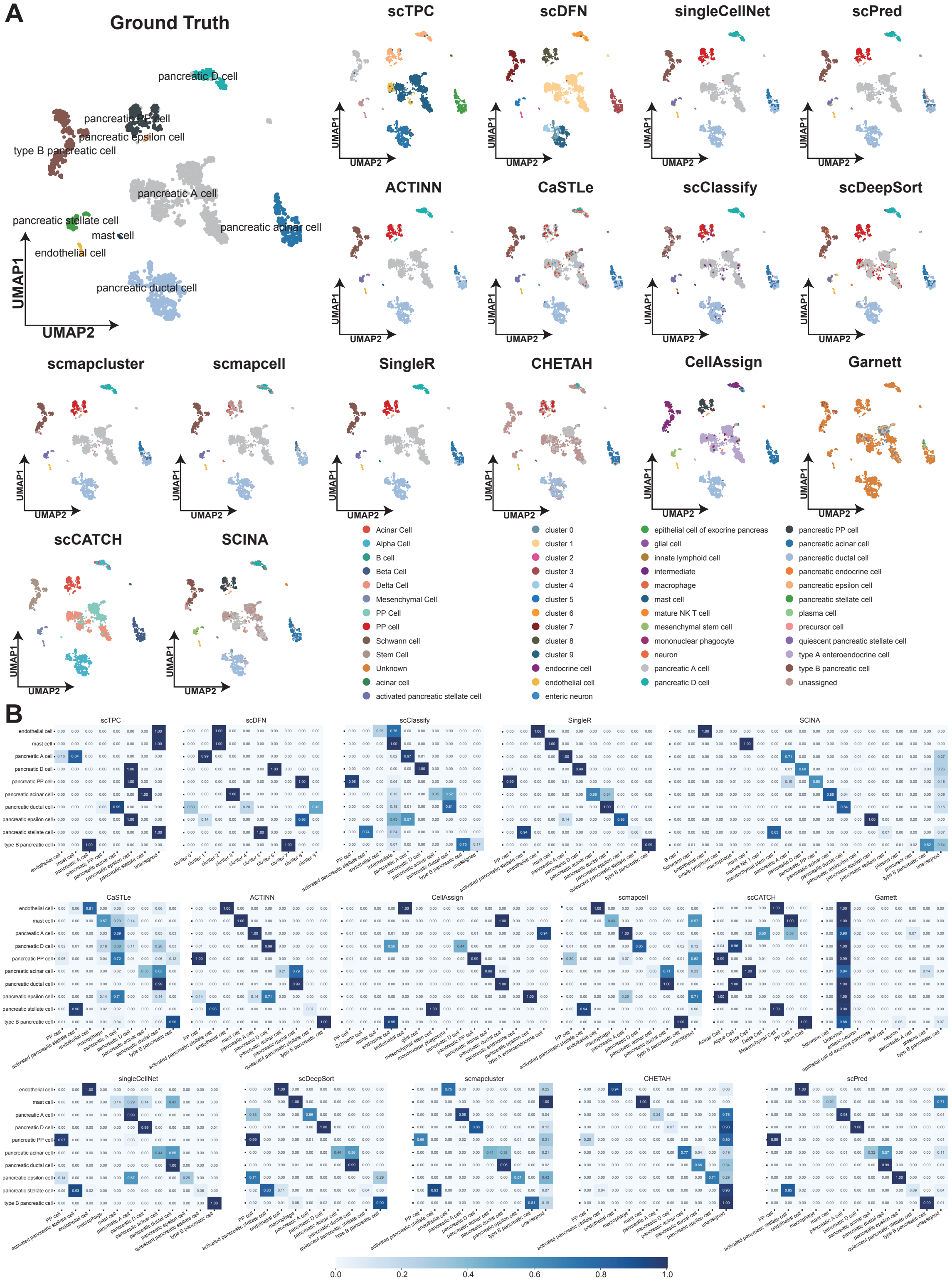

Supplementary Figure S57. (**A**) UMAP of the Segerstolpe dataset comparing ground truth, scTPC, scDFN, and reference-based annotation methods used for comparison, with the hBaron dataset serving as the reference for the reference-based annotation methods. (**B**) Heatmap showing correlations between the ground truth of the Segerstolpe dataset and the annotation results from each method, with the hBaron dataset serving as the reference for the reference-based annotation methods. The y-axis represents the ground truth of the query dataset, and the x-axis represents the annotations produced by each method.

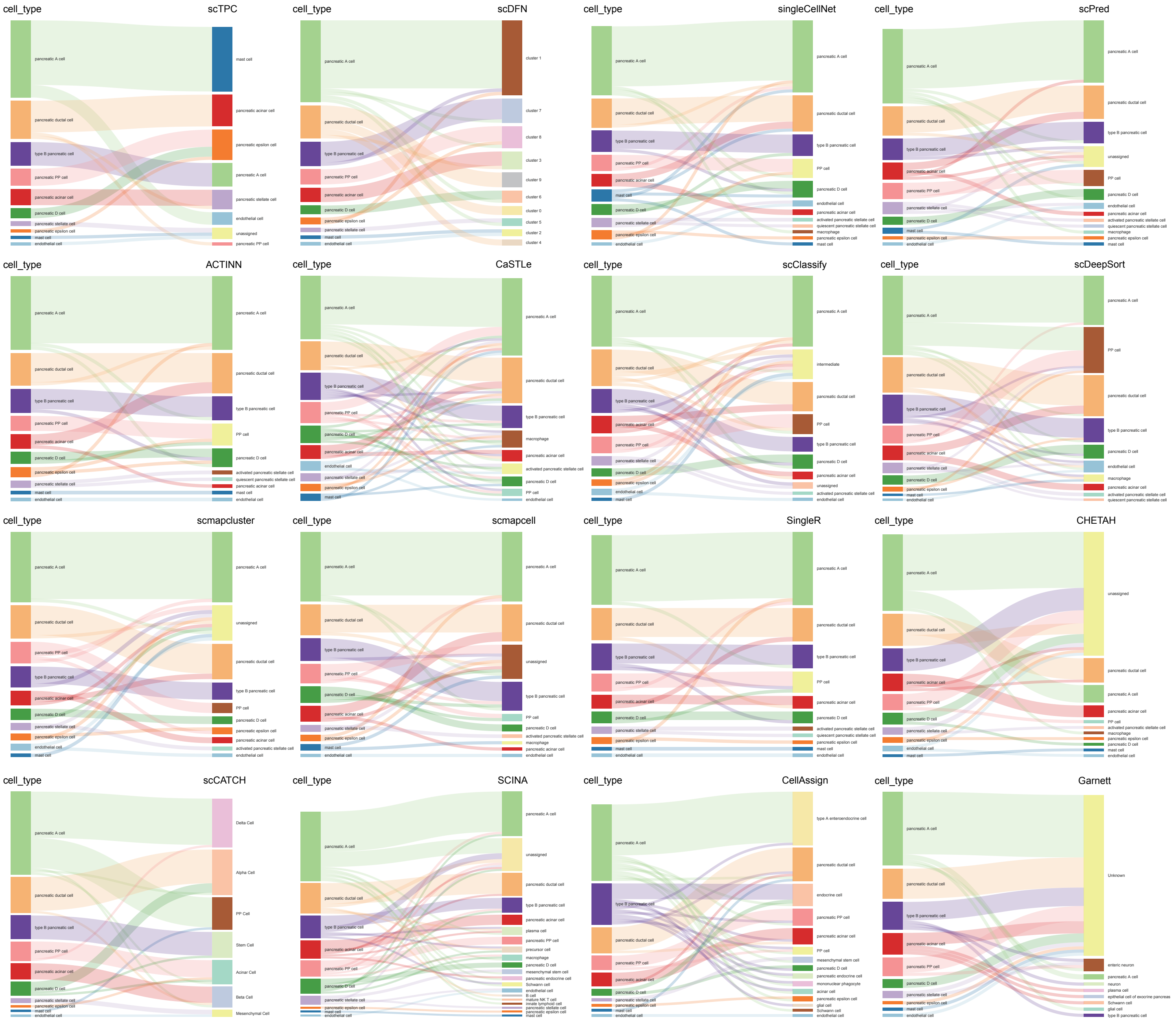

Supplementary Figure S58. Sankey plot illustrating the correspondence between the ground truth of the Segerstolpe dataset and the annotation results from each method, with the hBaron dataset serving as the reference for the reference-based annotation methods.

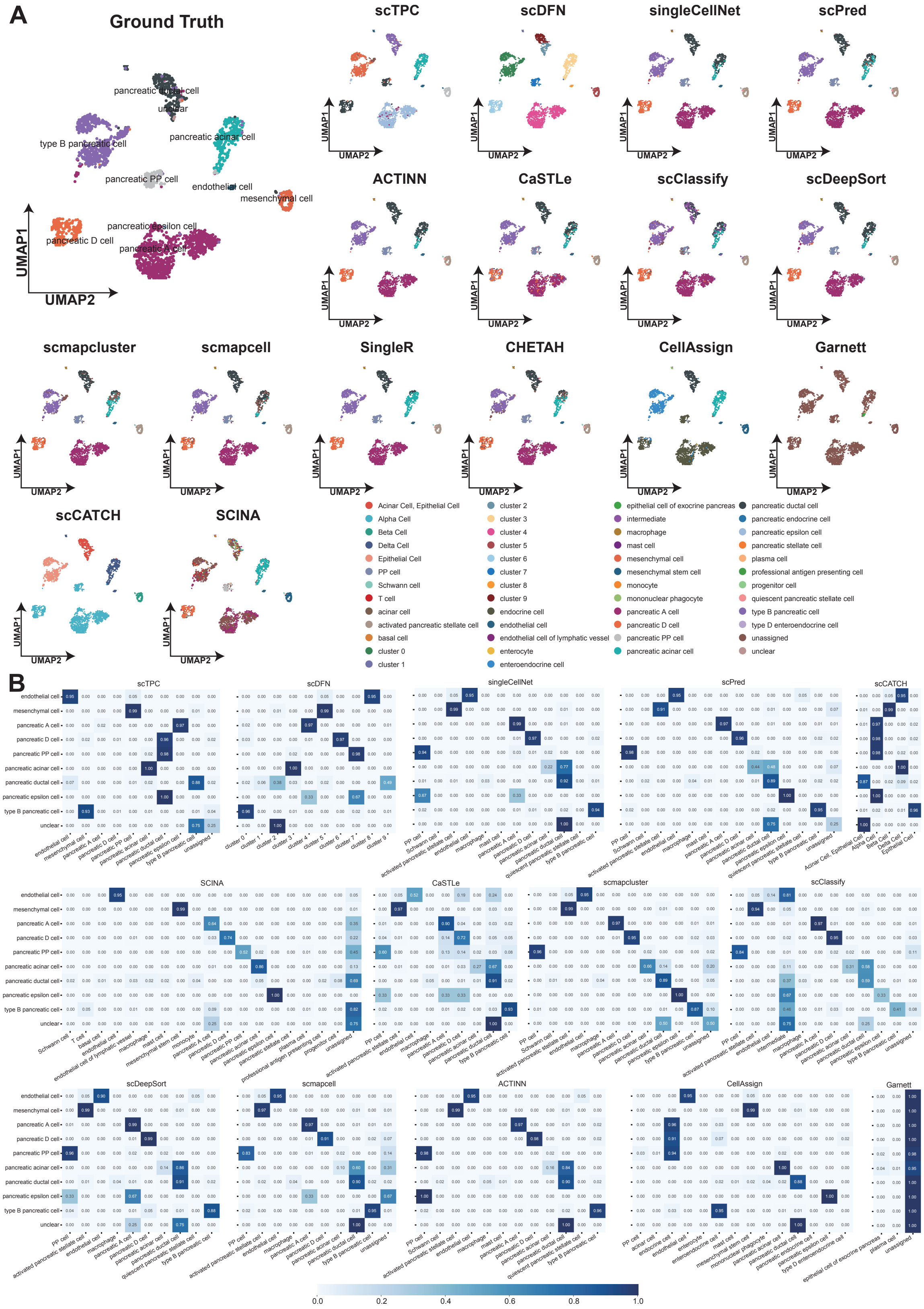

Supplementary Figure S59. (**A**) UMAP of the Muraro dataset comparing ground truth, scTPC, scDFN, and reference-based annotation methods used for comparison, with the hBaron dataset serving as the reference for the reference-based annotation methods. (**B**) Heatmap showing correlations between the ground truth of the Muraro dataset and the annotation results from each method, with the hBaron dataset serving as the reference for the reference-based annotation methods. The y-axis represents the ground truth of the query dataset, and the x-axis represents the annotations produced by each method.

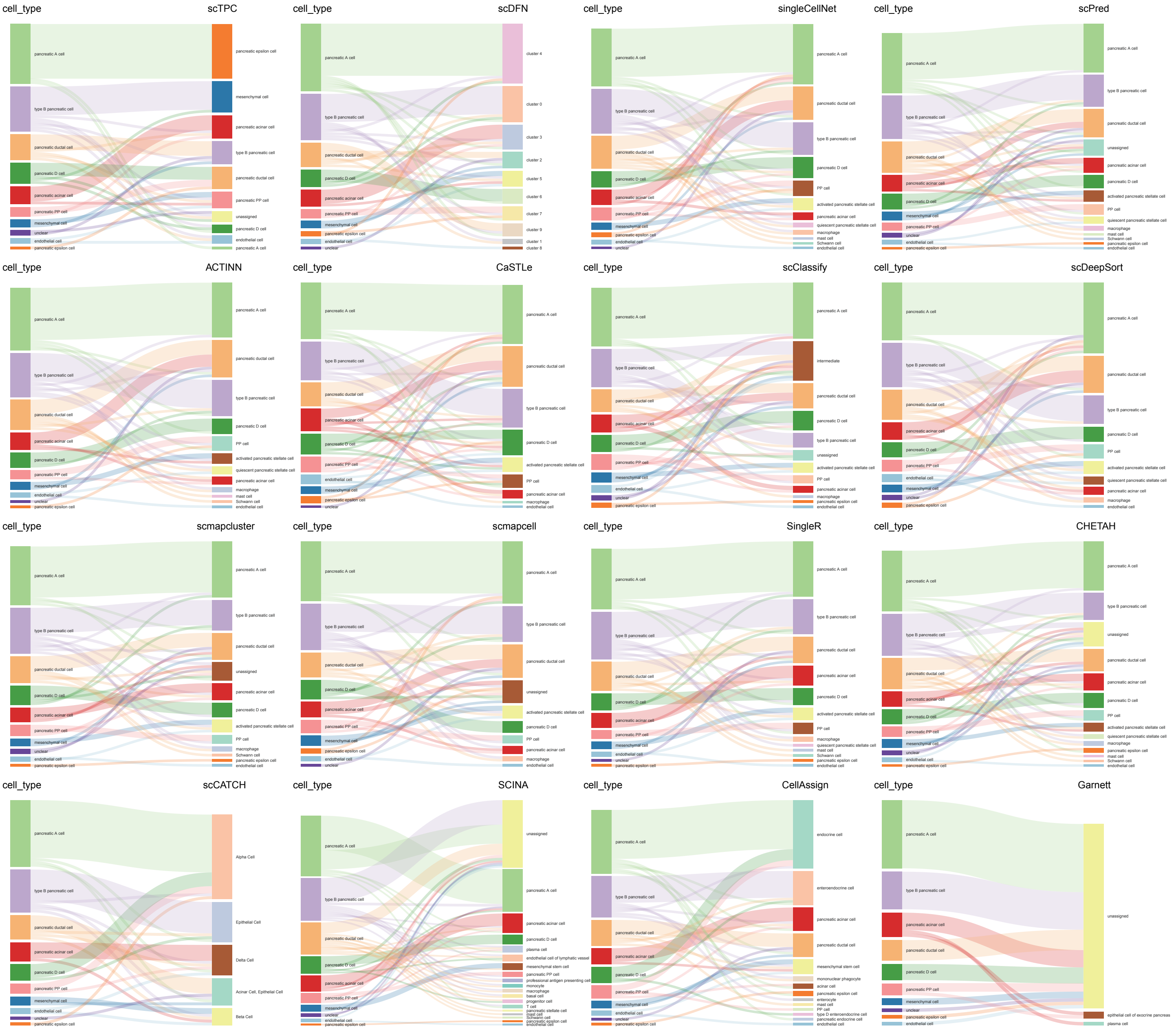

Supplementary Figure S60. Sankey plot illustrating the correspondence between the ground truth of the Muraro dataset and the annotation results from each method, with the hBaron dataset serving as the reference for the reference-based annotation methods.

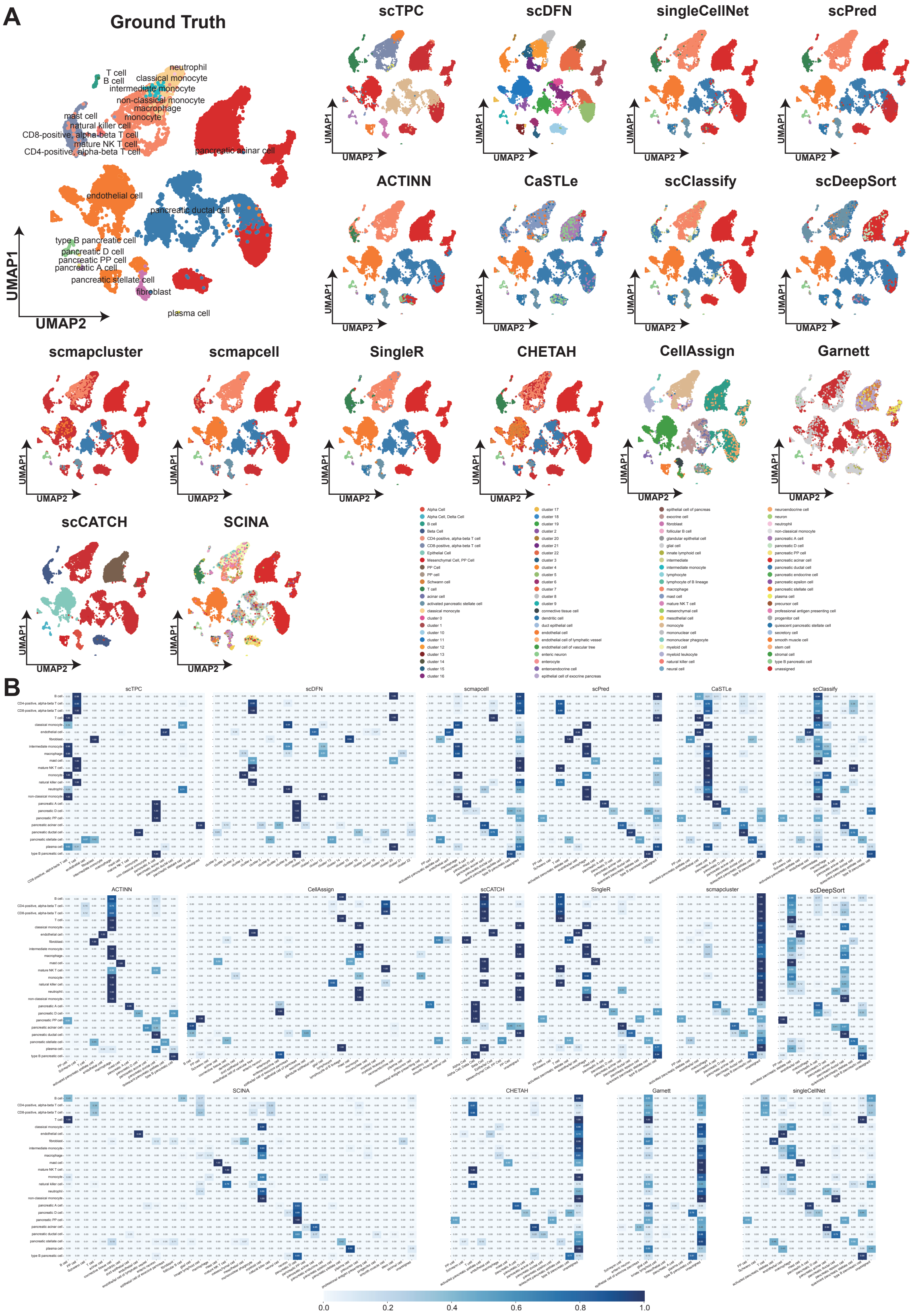

Supplementary Figure S61. (**A**) UMAP of the Tabula Sapiens Pancreas dataset comparing ground truth, scTPC, scDFN, and reference-based annotation methods used for comparison, with the hBaron dataset serving as the reference for the reference-based annotation methods. (**B**) Heatmap showing correlations between the ground truth of the Tabula Sapiens Pancreas dataset and the annotation results from each method, with the hBaron dataset serving as the reference for the reference-based annotation methods. The y-axis represents the ground truth of the query dataset, and the x-axis represents the annotations produced by each method.

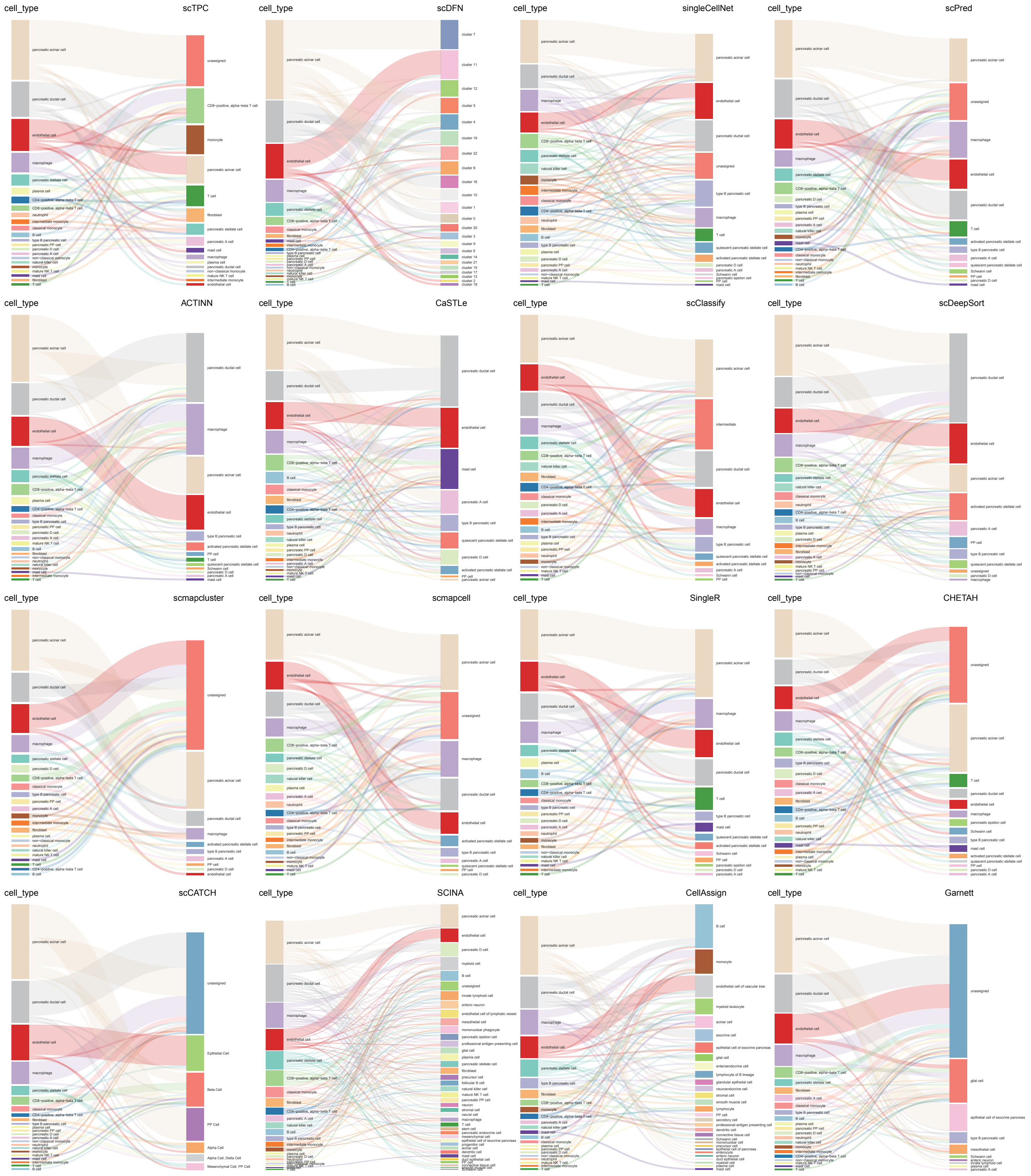

Supplementary Figure S62. Sankey plot illustrating the correspondence between the ground truth of the Tabula Sapiens Pancreas dataset and the annotation results from each method, with the hBaron dataset serving as the reference for the reference-based annotation methods.

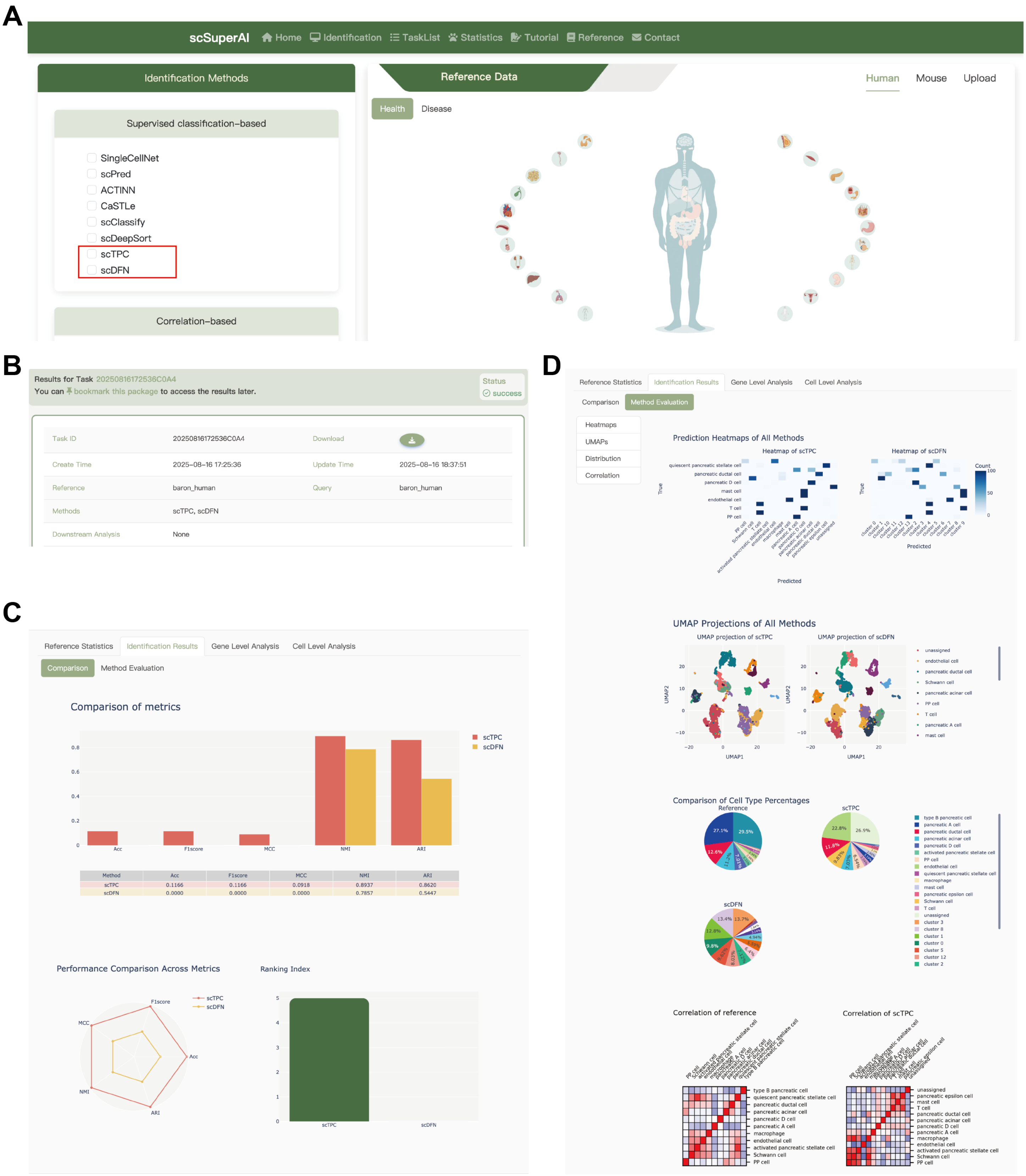

Supplementary Figure S63. Integration and evaluation of scTPC and scDFN within the scSuperAnnotator. (**A**) The Identification page of the scSuperAnnotator, where scTPC and scDFN have been added (highlighted with red boxes). (**B**) Task information for running scTPC and scDFN on the hBaron dataset. (**C**) Comparison results from the task under Identification Results. (**D**) Method Evaluation results from the task under Identification Results.

Supplementary Table S14. Method Performance

	method	hBaron(r)-Muraro(q)	hBaron(r)-Segerstolpe(q)	hBaron(r)-TS Pancreas(q)	Fasolino(r)-hBaron(q)
	singleCellNet	0.8393	0.9085	0.7400	0.8105
NMI	scPred	0.8358	0.8986	0.8248	0.8019
	ACTINN	0.8455	0.9194	0.7411	0.8344
	CaSTLe	0.6577	0.5736	0.6251	0.4827
	scClassify	0.7291	0.8003	0.6809	0.5917
	scDeepSort	0.8337	0.7707	0.5330	0.8168
	scmapcluster	0.8425	0.8124	0.4309	0.6125
	scmapcell	0.8134	0.8471	0.6972	0.7200
	SingleR	0.8786	0.9413	0.8490	0.8270
	CHETAH	0.7915	0.4532	0.5251	0.6345
	scCATCH	0.7371	0.8406	0.6149	0.7667
	SCINA	0.5500	0.6873	0.7047	0.3932
	CellAssign	0.7390	0.8771	0.7530	0.8649
	Garnett	0.0151	0.0968	0.1925	0.2431
	scTPC	0.8828	0.8936	0.9107	0.9251
	scDFN	0.8724	0.9253	0.7075	0.8107
ARI	singleCellNet	0.8773	0.9186	0.8004	0.8631
	scPred	0.8844	0.9143	0.8027	0.8526
	ACTINN	0.8661	0.9168	0.5684	0.8799
	CaSTLe	0.7145	0.5683	0.4588	0.3890
	scClassify	0.7642	0.8464	0.7331	0.4599
	scDeepSort	0.8662	0.6153	0.3205	0.8879
	scmapcluster	0.8910	0.8597	0.2868	0.4435
	scmapcell	0.8710	0.8831	0.7122	0.6865
	SingleR	0.9161	0.9547	0.8467	0.8787
	CHETAH	0.8414	0.1682	0.4663	0.6076
	scCATCH	0.6545	0.6709	0.3284	0.6040
	SCINA	0.3442	0.5718	0.7008	0.1718
	CellAssign	0.6564	0.8975	0.7019	0.9083
	Garnett	0.0046	−0.0124	0.0127	0.0452
	scTPC	0.9159	0.8202	0.9533	0.9558
	scDFN	0.9134	0.9286	0.4043	0.6478

11 Supplementary Note 11: Supplementary for ”Identification of potential disease-associated cell populations”

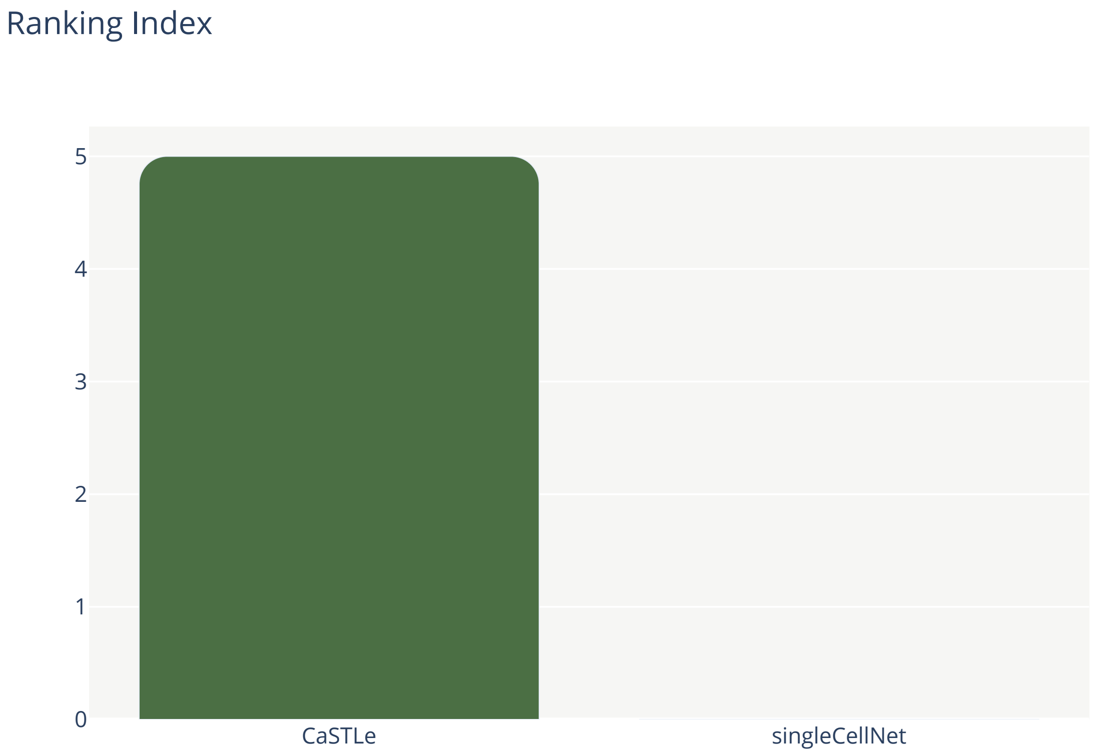

Supplementary Figure S64. Ranking index for CaSTLe and singleCellNet.

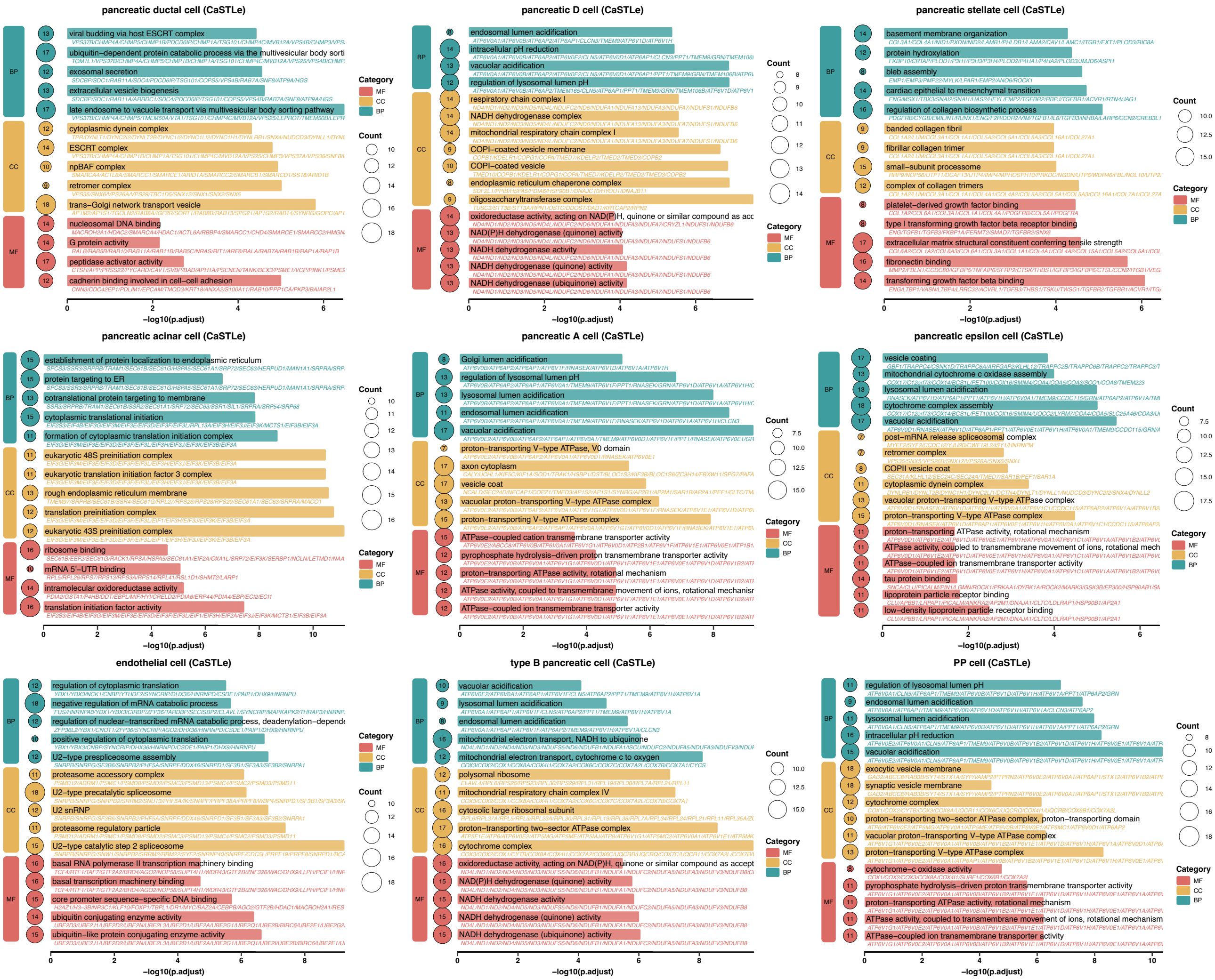

Supplementary Figure S65. GO enrichment of cell types identified by CaSTLe across biological processes, cellular components and molecular functions.

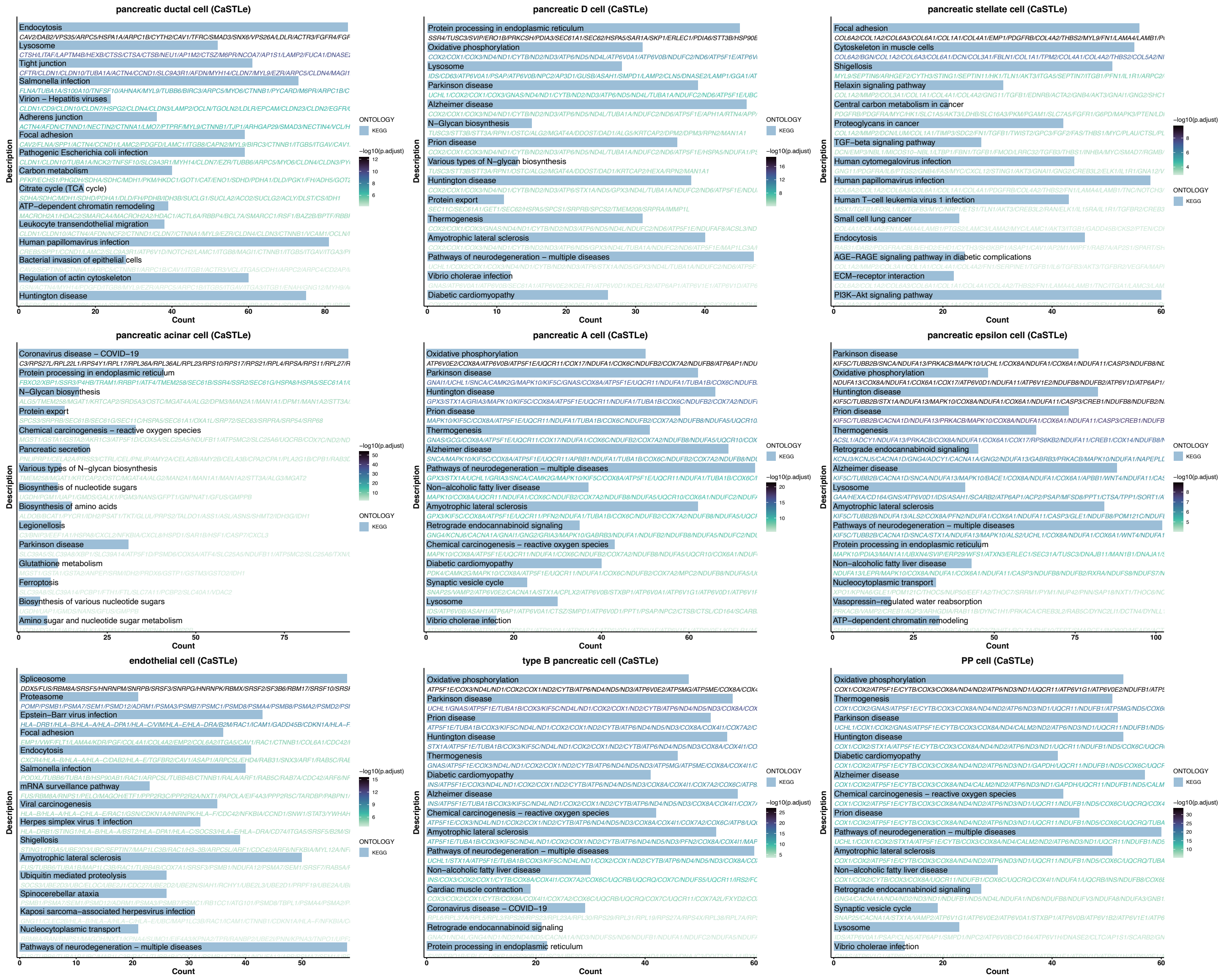

Supplementary Figure S66. KEGG pathways enriched in cell types identified by CaSTLe.

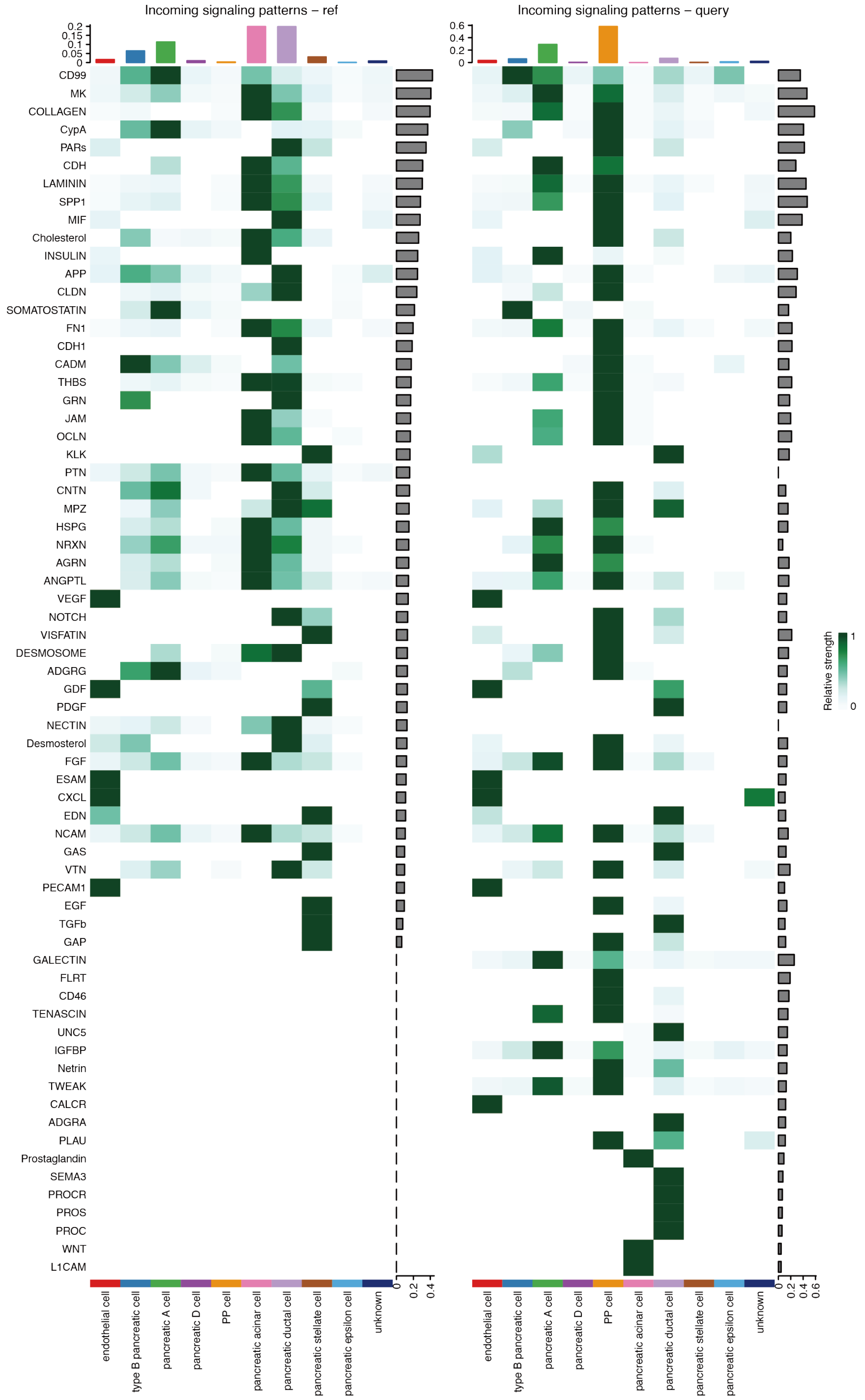

Supplementary Figure S67. Comparison of incoming signaling patterns between the healthy reference dataset (ref) and the T1D dataset identified by CaSTLe (query).

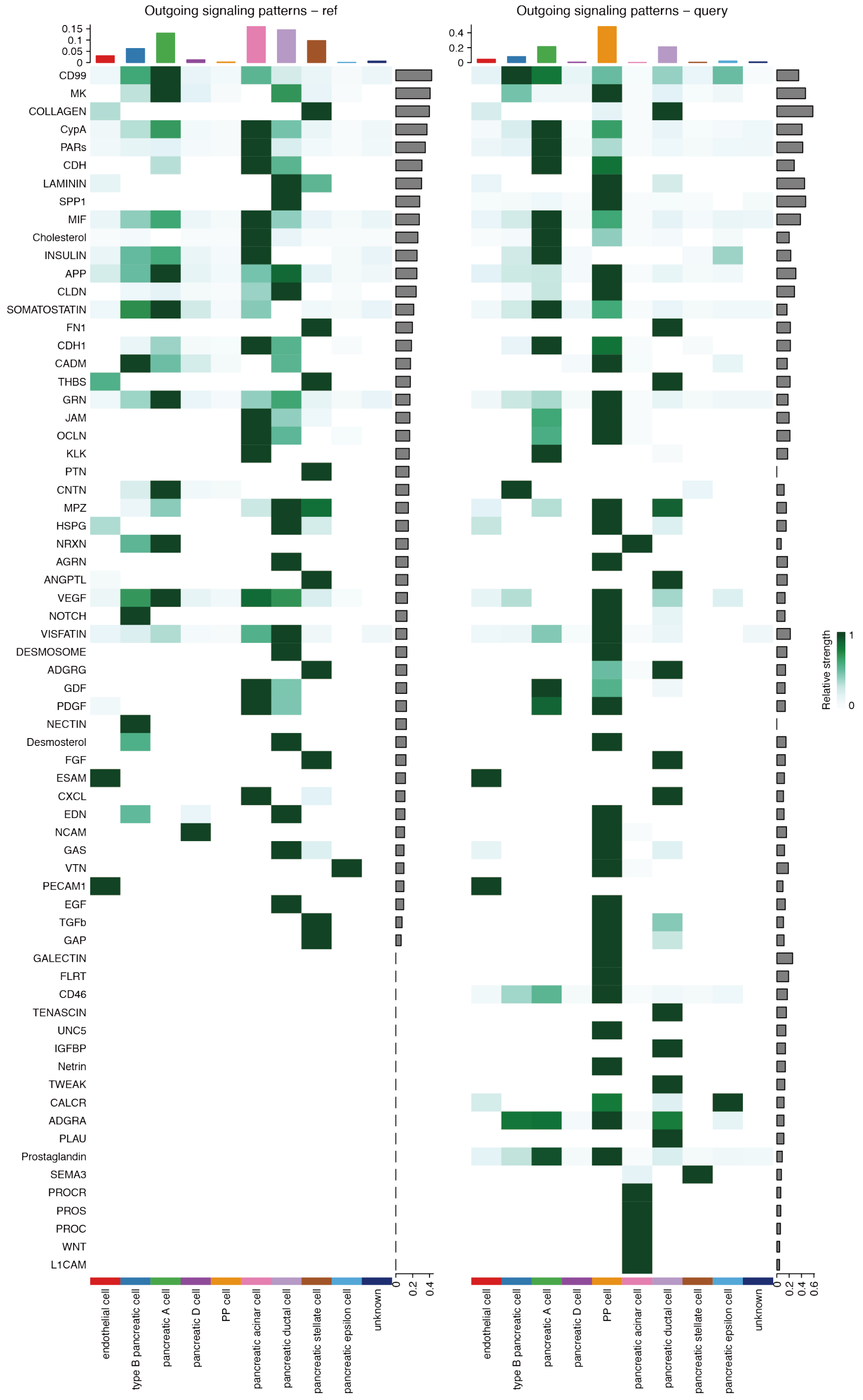

Supplementary Figure S68. Comparison of outgoing signaling patterns between the healthy reference dataset (ref) and the T1D dataset identified by CaSTLe (query).

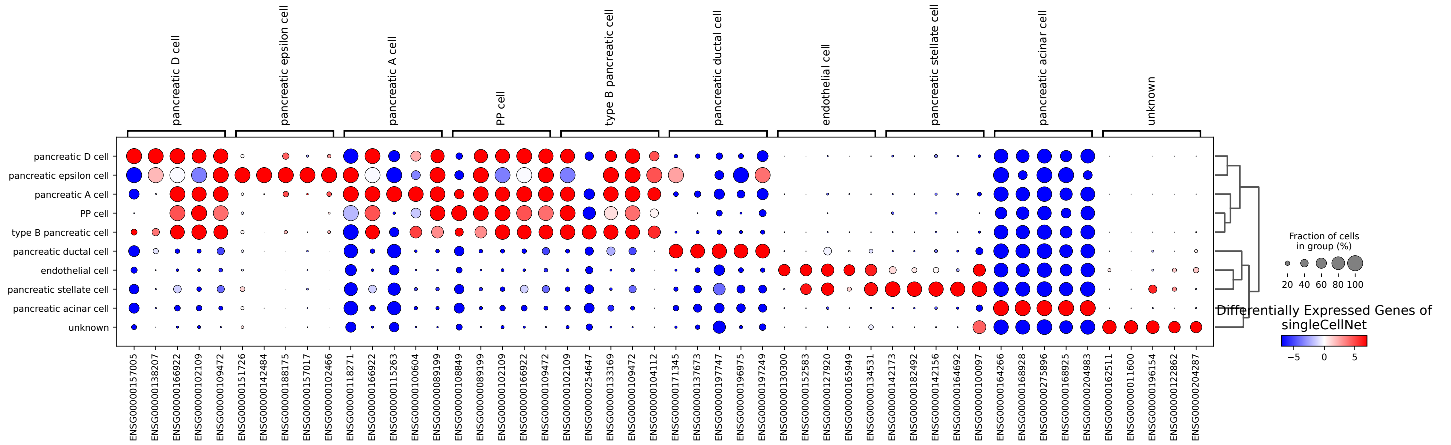

Supplementary Figure S69. Differential expression genes across various cell types identified by singleCellNet.

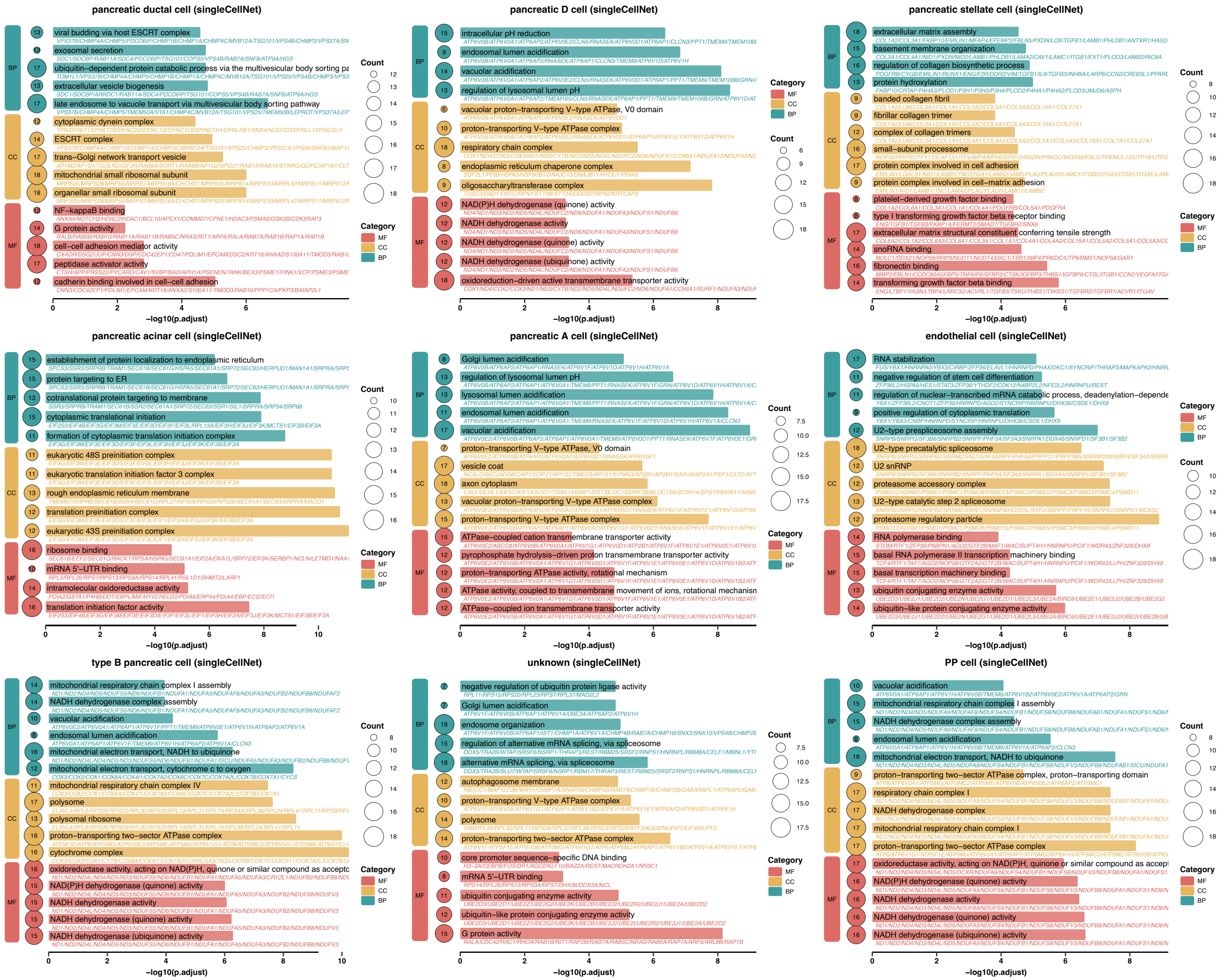

Supplementary Figure S70. GO enrichment of cell types identified by singleCellNet across biological processes, cellular components and molecular functions.

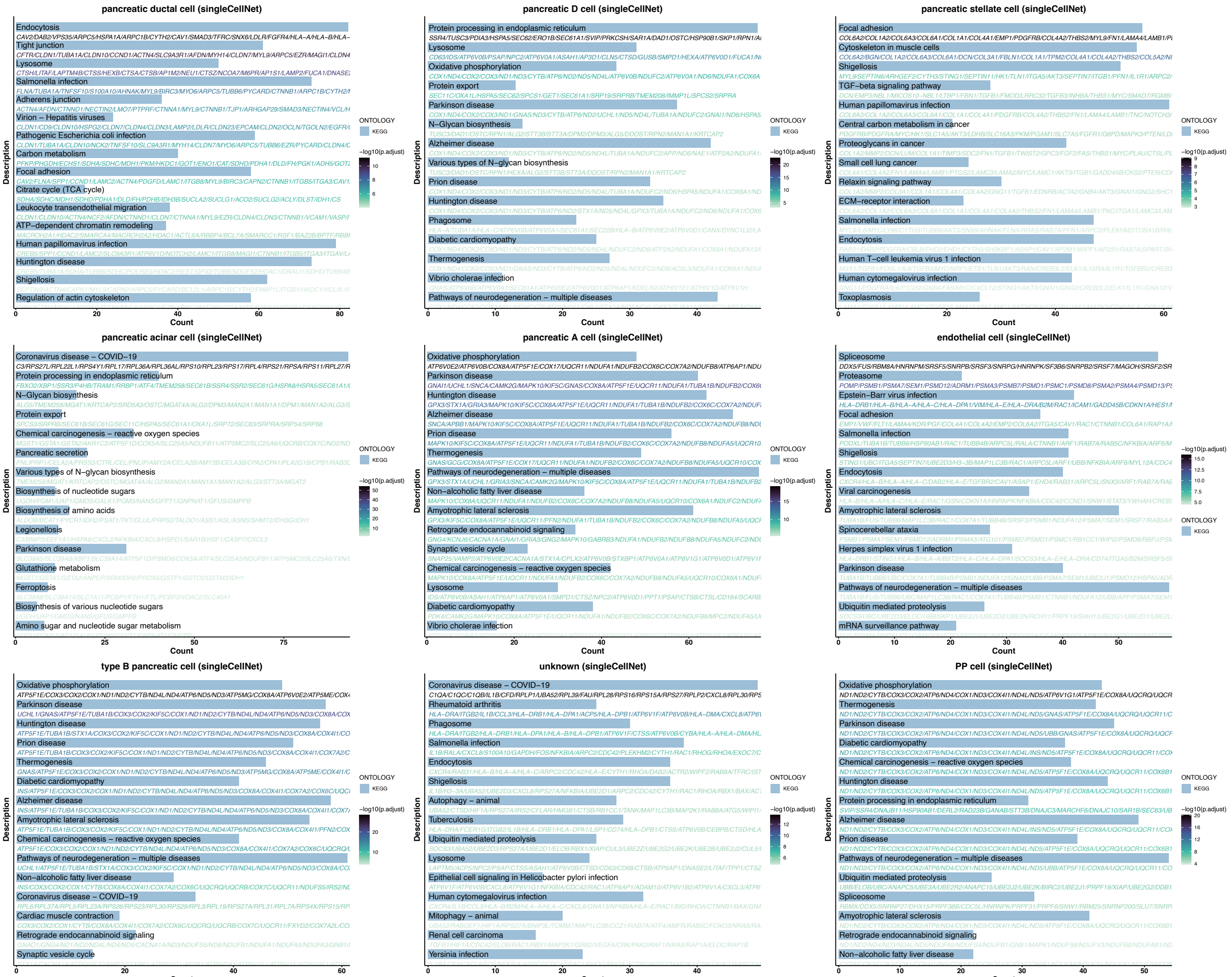

Supplementary Figure S71. KEGG pathways enriched in cell types identified by singleCellNet.

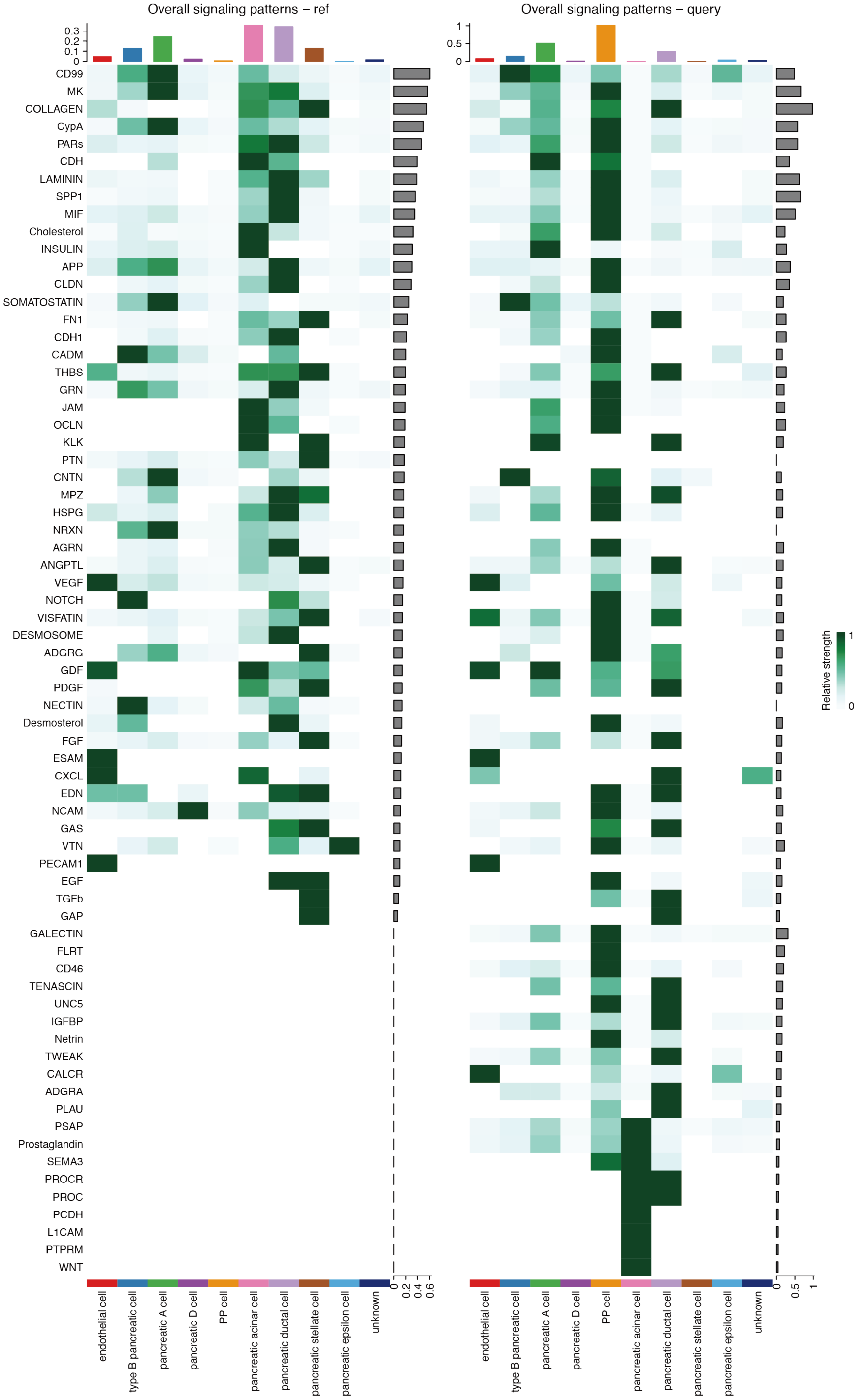

Supplementary Figure S72. Comparison of overall signaling patterns between the healthy reference dataset (ref) and the T1D dataset identified by singleCellNet (query).

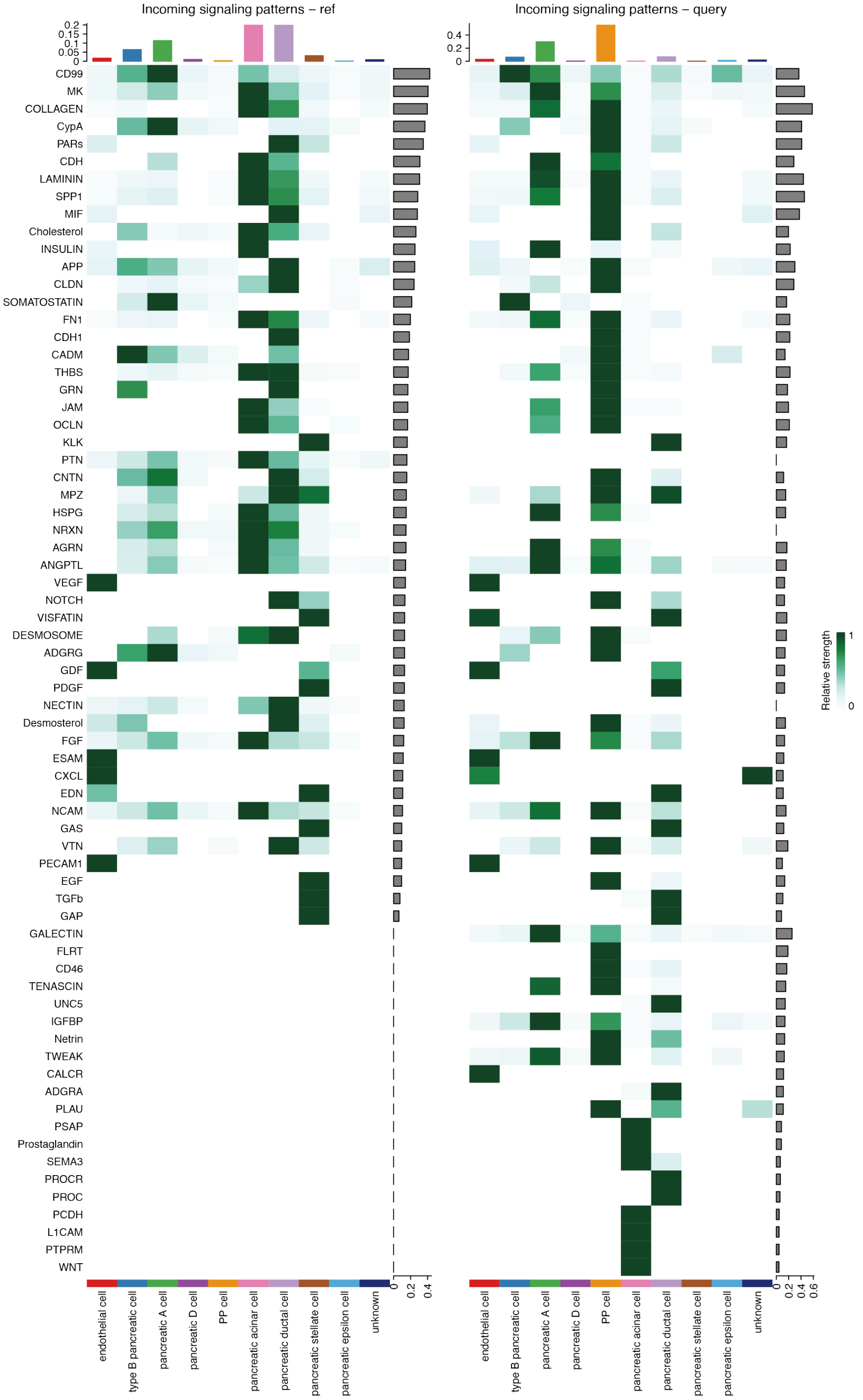

Supplementary Figure S73. Comparison of incoming signaling patterns between the healthy reference dataset (ref) and the T1D dataset identified by singleCellNet (query).

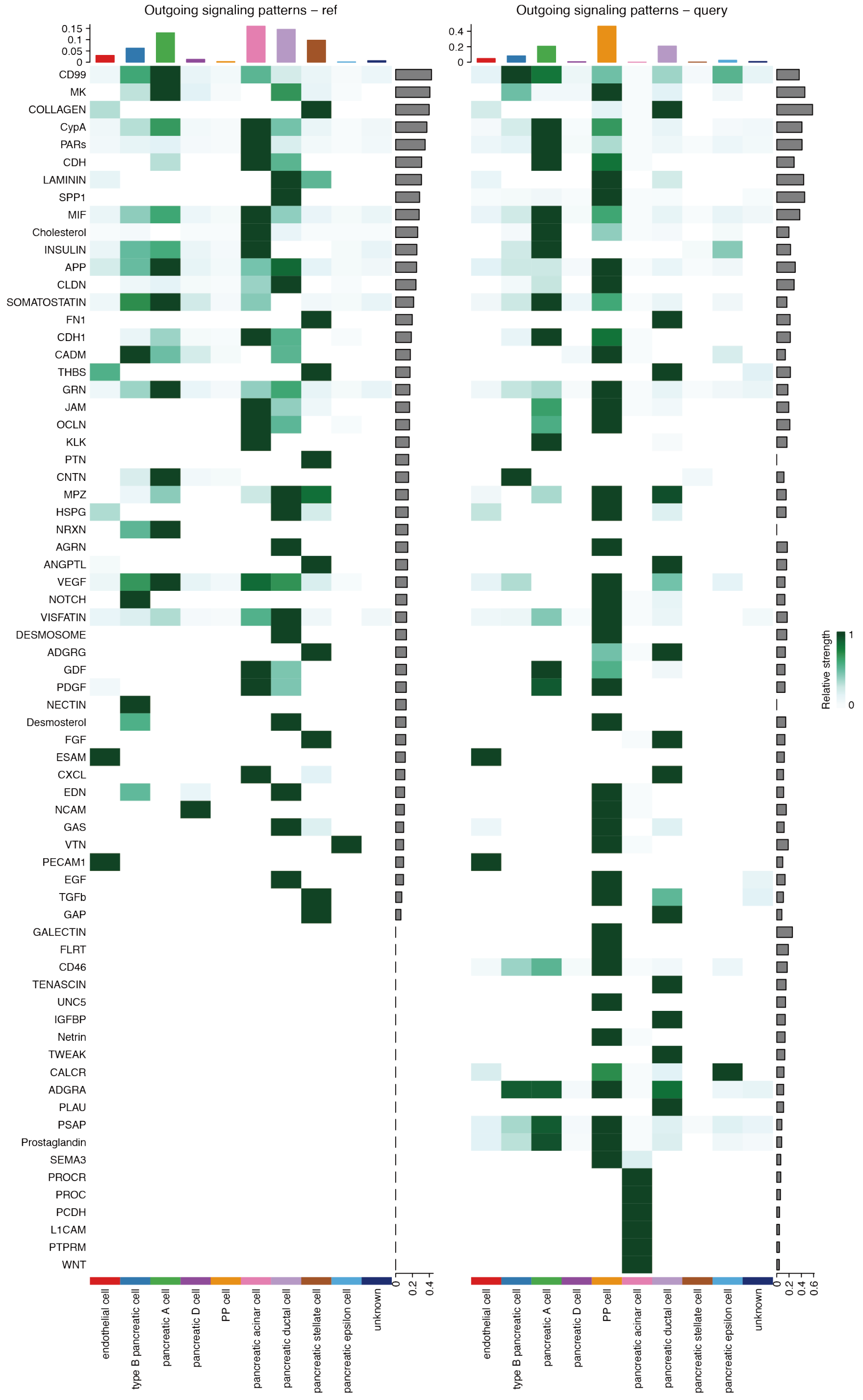

Supplementary Figure S74. Comparison of outgoing signaling patterns between the healthy reference dataset (ref) and the T1D dataset identified by singleCellNet (query).

12 Supplementary Note 12: Supplementary for "Deciphering disease biology through cell signaling and trajectory analysis"

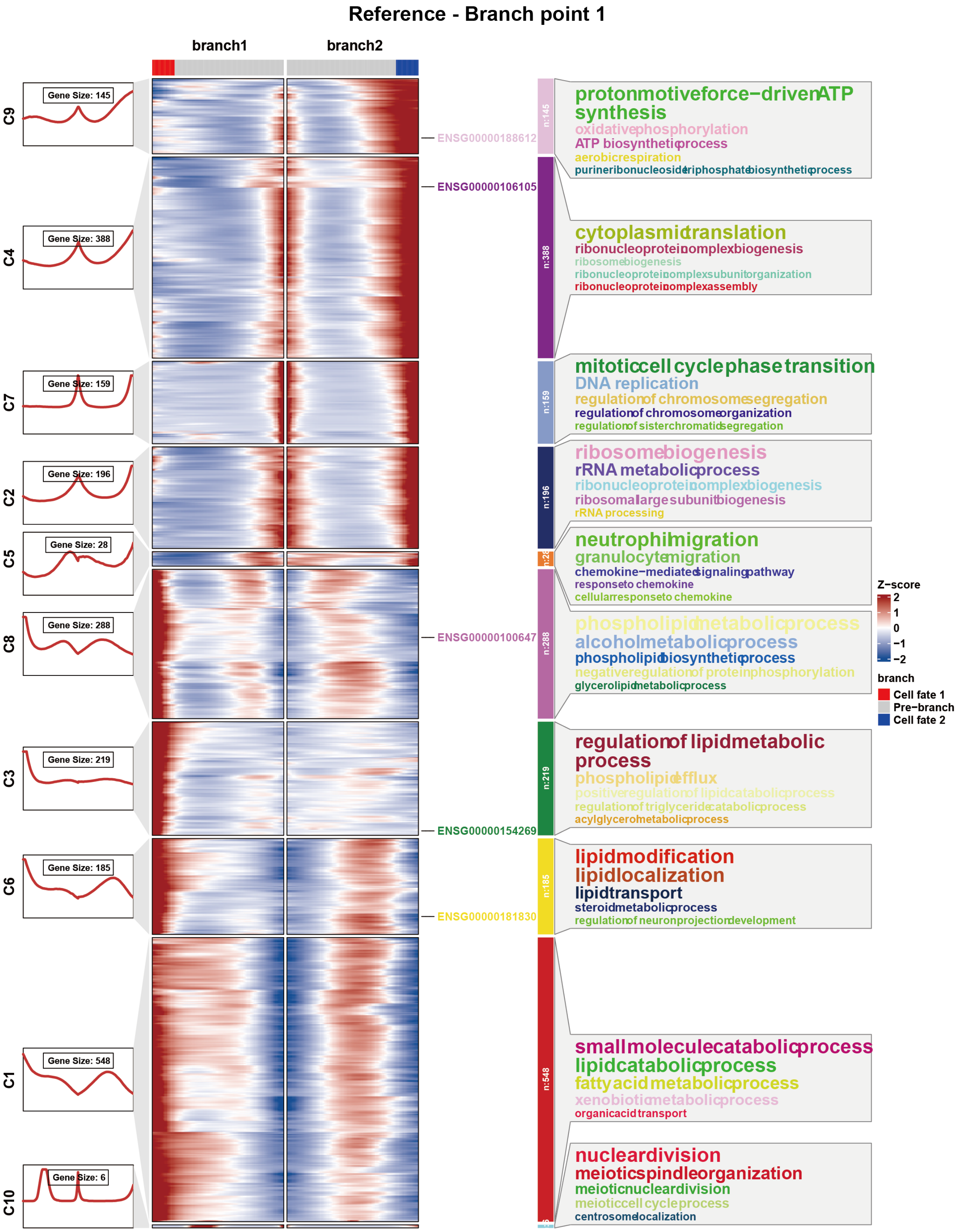

Supplementary Figure S75. BEAM analysis of reference at branch point 1.

CaSTLe - Branch point 1

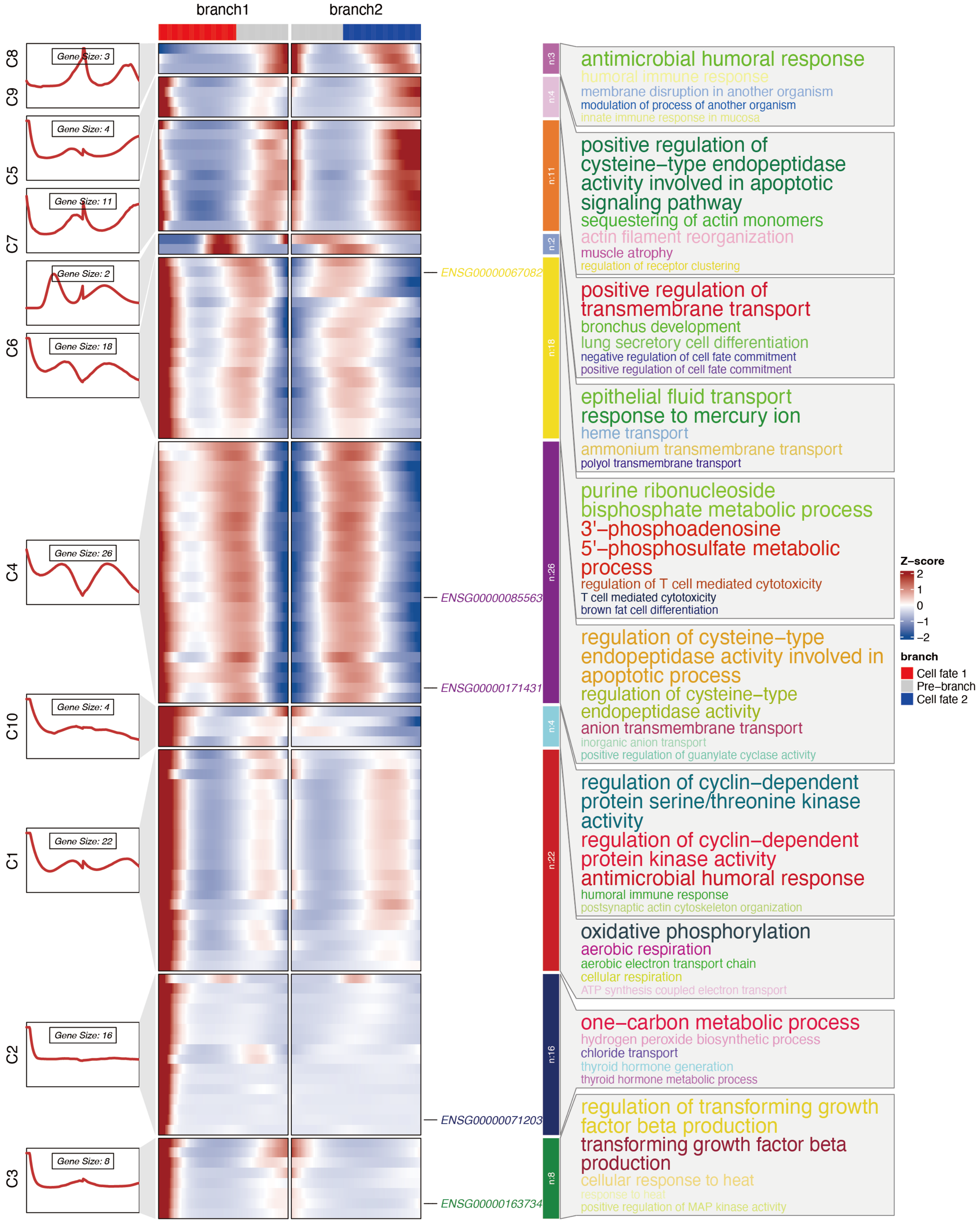

Supplementary Figure S76. BEAM analysis of CaSTLe results at branch point 1.

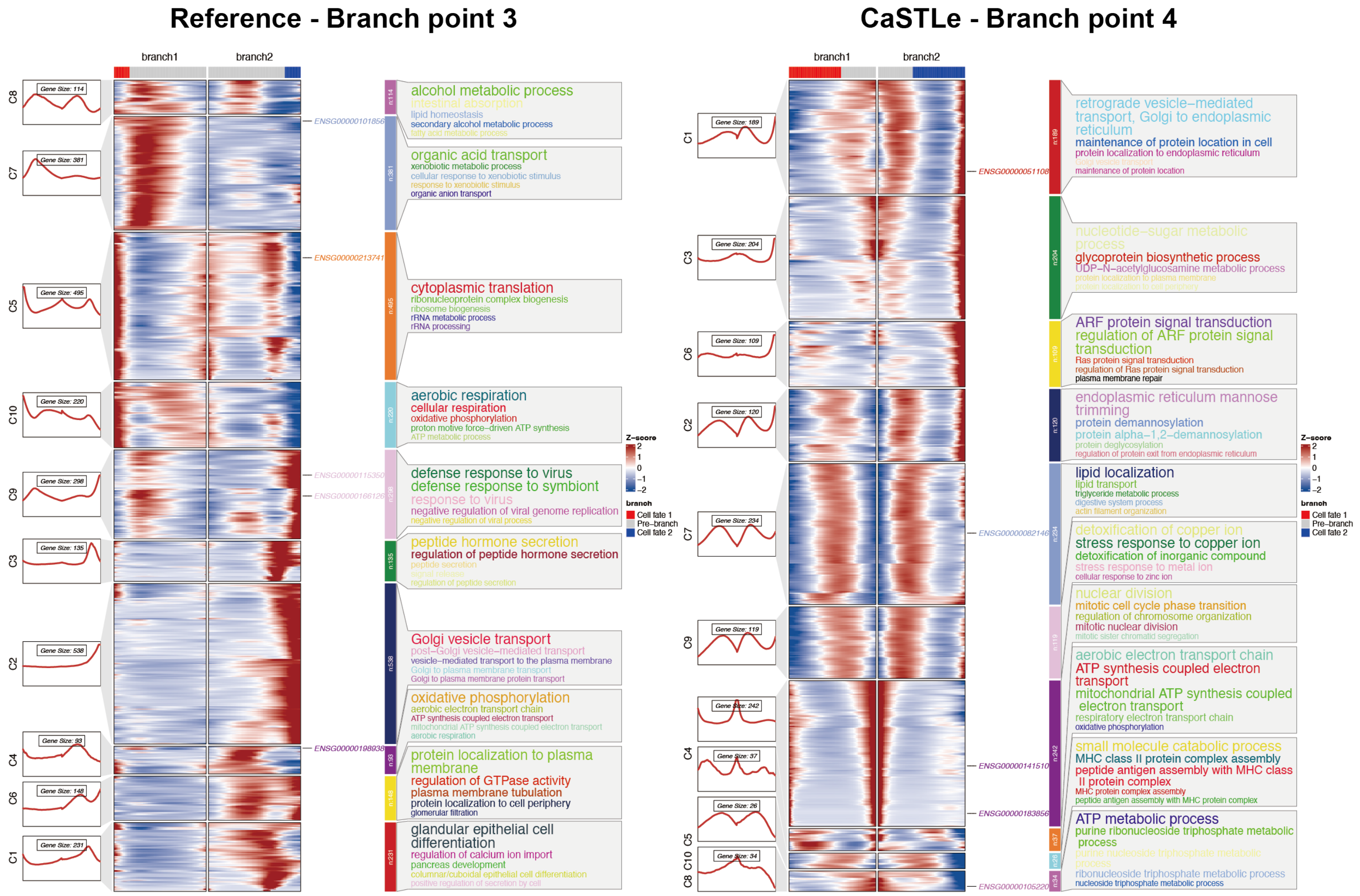

Supplementary Figure S77. Comparison of BEAM analysis between branch point 3 of reference and branch point 4 of CaSTLe results.

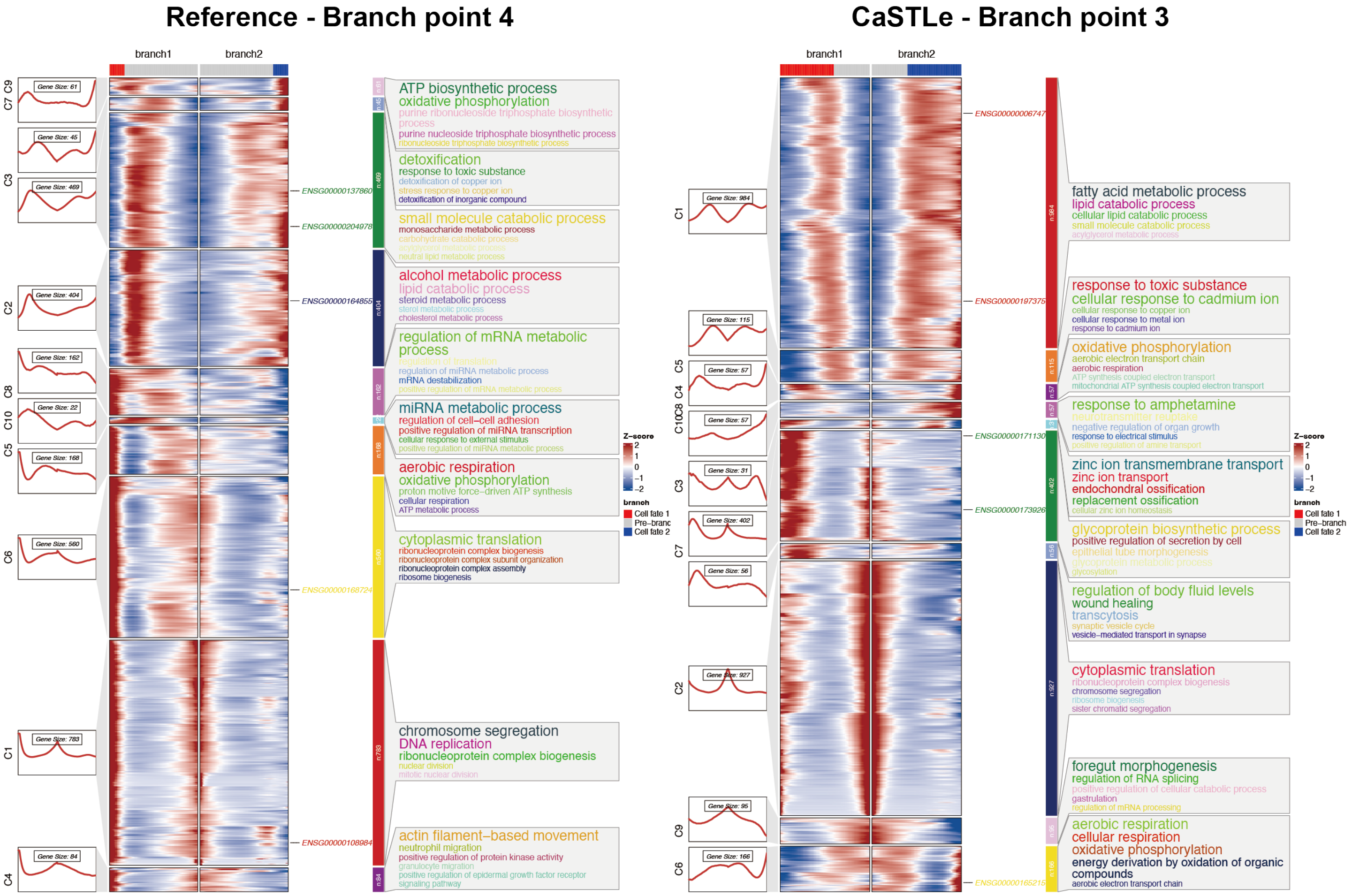

Supplementary Figure S78. Comparison of BEAM analysis between branch point 4 of reference and branch point 3 of CaSTLe results.

13 Supplementary Note 13: Example Outputs from the Web Server

Accurate cell type identification is critical for interpreting single-cell data and uncovering its biological significance. Different computational methods have distinct strengths and limitations, making rigorous benchmarking across multiple metrics essential for identifying the optimal approach and ensuring reliable and reproducible results. Here, we utilized fourteen cell identification methods (singleCellNet, scPred, ACTINN, CaSTLe, scClassify, scDeepSort, scmapcluster, scmapcell, SingleR, CHETAH, scCATCH, SCINA, CellAssign, and Garnett) to identify cell types in the archived human Segerstolpe dataset, using the archived human Muraro dataset as the reference. In addition to evaluating the performance of algorithms on specific datasets, the platform provides insights into their robustness across diverse datasets and conditions, sensitivity to data complexity and batch effects, computational efficiency, and scalability, offering comprehensive guidance for algorithm selection and optimization. By combining quantitative and qualitative comparisons from multiple perspectives, we demonstrate the scSuperAnnotator's capability for algorithmic evaluation, offering insights into performance, robustness, sensitivity to data complexity and batch effects, computational efficiency, and scalability, thereby providing comprehensive guidance for algorithm selection and optimization. Supplementary Fig. S79A and B present the performance of the compared methods across Acc, F1-Score, MCC, NMI, and ARI. The results highlight that supervised classification-based methods, such as singleCellNet, scPred, CaSTLe, and scClassify, consistently achieve strong predictive performance across all metrics. Correlation-based methods, including scmapcluster and SingleR, also demonstrate relatively good performance. In contrast, marker gene-based methods, such as CellAssign, SCINA, scCATCH, and Garnett, exhibit weaker performance across the evaluated metrics. Supplementary Fig. S79C highlights the relative performance of cell type identification methods, with supervised classification-based approaches, including singleCellNet, scPred, scClassify, and CaSTLe, occupying the largest areas in the radar plot, reflecting their robust performance across all metrics. In contrast, correlation-based methods, such as scmapcluster and SingleR, show moderate performance, while marker gene-based methods, including CellAssign, SCINA, scCATCH, and Garnett, consistently underperform across the evaluated metrics. Supplementary Fig. S79D further illustrates the Ranking Index (RI) of these methods, summarizing their overall performance. Supervised classification-based methods rank the highest, with a score of 5, followed by correlation-based approaches with ranking index close to 5. ACTINN exhibits moderate performance, with ranking index between 4 and 5. Marker gene-based methods rank the lowest, likely due to discrepancies between cell type names in the marker gene sets and query dataset labels, as well as the limited number of marker genes. These results underscore the reliability and superior predictive power of supervised classification-based methods compared to marker gene-based approaches.

We further evaluated the performance of each method, with Supplementary Fig. S79E comparing the accuracy of cell type identification by visualizing the agreement between true and predicted cell types. Supervised classification-based methods showed strong diagonal patterns, indicating high accuracy, while marker gene-based methods exhibited diffuse patterns, reflecting weaker performance, particularly when gene expression poorly matched predefined marker sets. Correlation-based methods demonstrated moderate accuracy with some misclassifications. Supplementary Fig. S79F uses UMAP plots to assess cluster distinctness and compactness, revealing that supervised methods produced well-separated, compact clusters consistent with their superior performance, whereas marker gene-based methods displayed overlapping, diffuse clusters, aligning with their lower accuracy. The UMAP plots also highlighted variability in cell type classification, with tight clusters indicating easier classification and diffuse clusters posing greater challenges. Together, these results confirm the superior reliability and accuracy of supervised classification-based methods for consistent cell type identification across diverse datasets.

Each identification method exhibits distinct strengths and limitations, with no single approach standing out as universally superior. Notably, the primary objective of this analysis is to showcase the platform's ability to visualize and evaluate results from multiple perspectives, rather than to endorse specific algorithms. scSuperAnnotator serves as a valuable resource, providing users with comprehensive insights to guide the selection of the most suitable method for their specific needs.

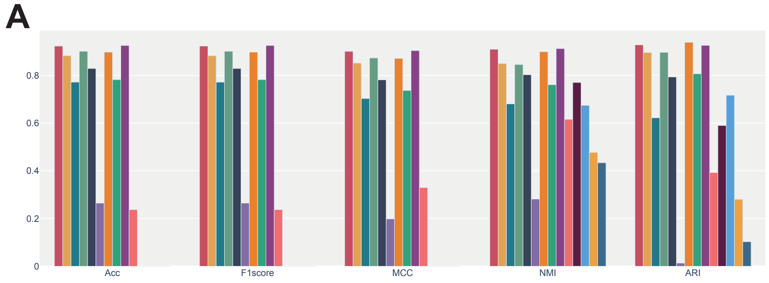

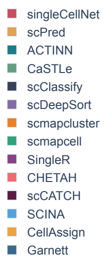

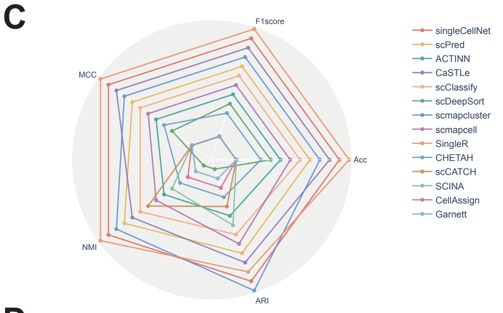

B

Method	Acc	F1score	MCC	NMI	ARI
singleCellNet	0.9230	0.9230	0.9010	0.9098	0.9278
scPred	0.8828	0.8828	0.8517	0.8497	0.8959
ACTINN	0.7718	0.7718	0.7030	0.6807	0.6224
CaSTLe	0.9013	0.9013	0.8730	0.8455	0.8966
scClassify	0.8289	0.8289	0.7815	0.8029	0.7933
scDeepSort	0.2648	0.2648	0.1985	0.2817	0.0134
scmapcluster	0.8977	0.8977	0.8712	0.8994	0.9389
scmapcell	0.7823	0.7823	0.7368	0.7611	0.8069
SingleR	0.9253	0.9253	0.9040	0.9124	0.9258
CHETAH	0.2372	0.2372	0.3296	0.6160	0.3924
scCATCH	0.0000	0.0000	0.0000	0.7702	0.5899
SCINA	0.0000	0.0000	0.0000	0.6745	0.7170
CellAssign	0.0000	0.0000	0.0000	0.4773	0.2806
Garnett	0.0000	0.0000	0.0000	0.4338	0.1027

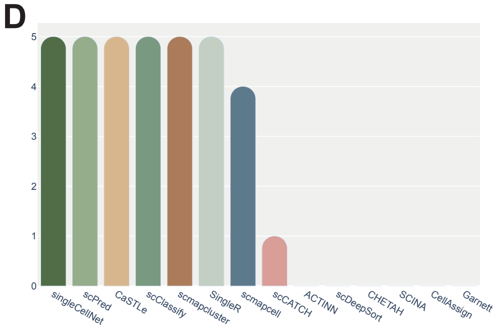

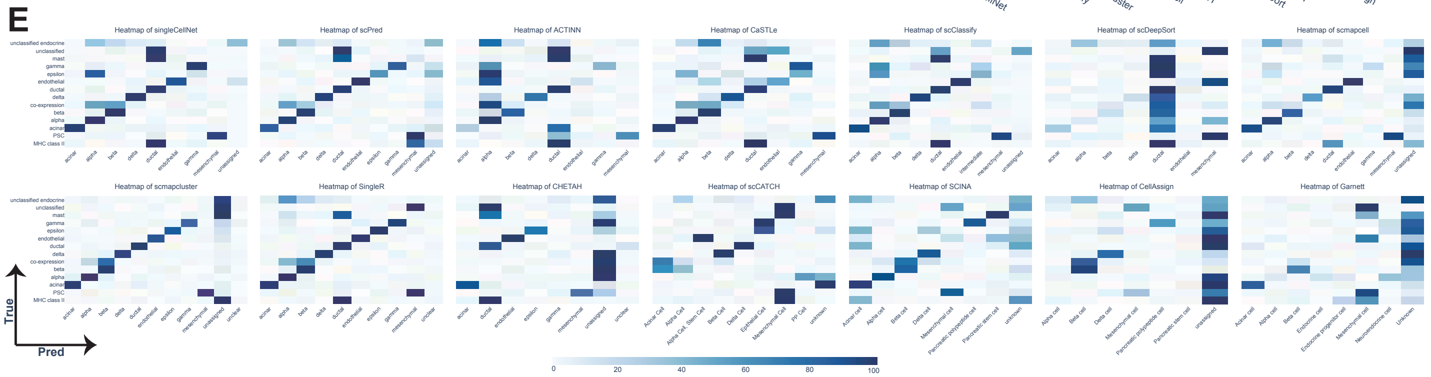

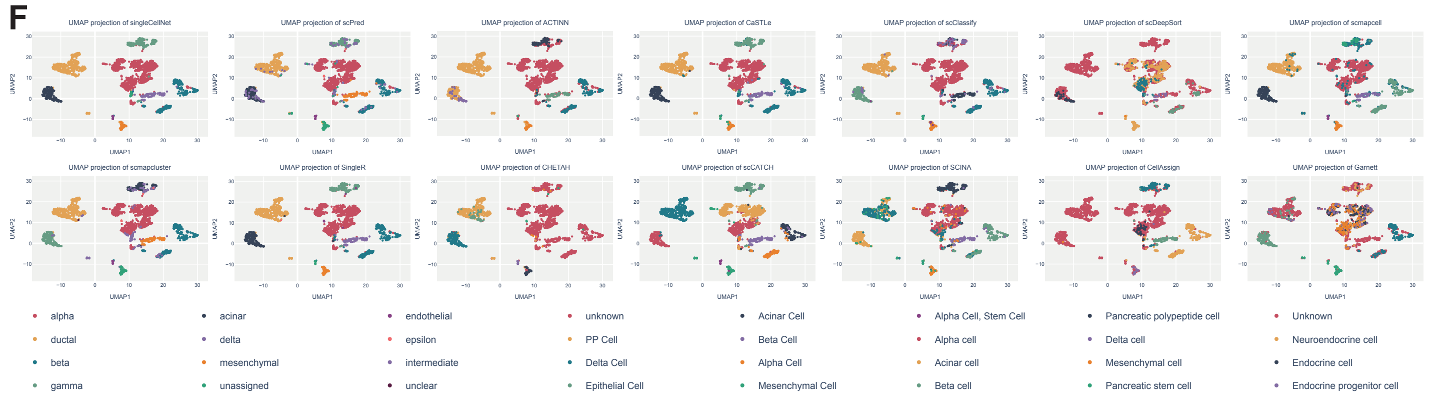

Supplementary Figure S79. (**A**) Comparison of performance metrics across different algorithms trained on the Muraro reference dataset and annotated on the Segerstolpe dataset. (**B**) Table detailing the performance metrics for each algorithm. (**C**) Overview of general performance for selected algorithms. (**D**) Comparison of Ranking index. (**E**) Heatmaps illustrating the correspondence between predicted(x-axis) and true(y-axis) cell types across selected methods. (**F**) UMAP visualizations of annotation results for selected methods.

14 Supplementary Note 14: Web Server Overview and Screenshot

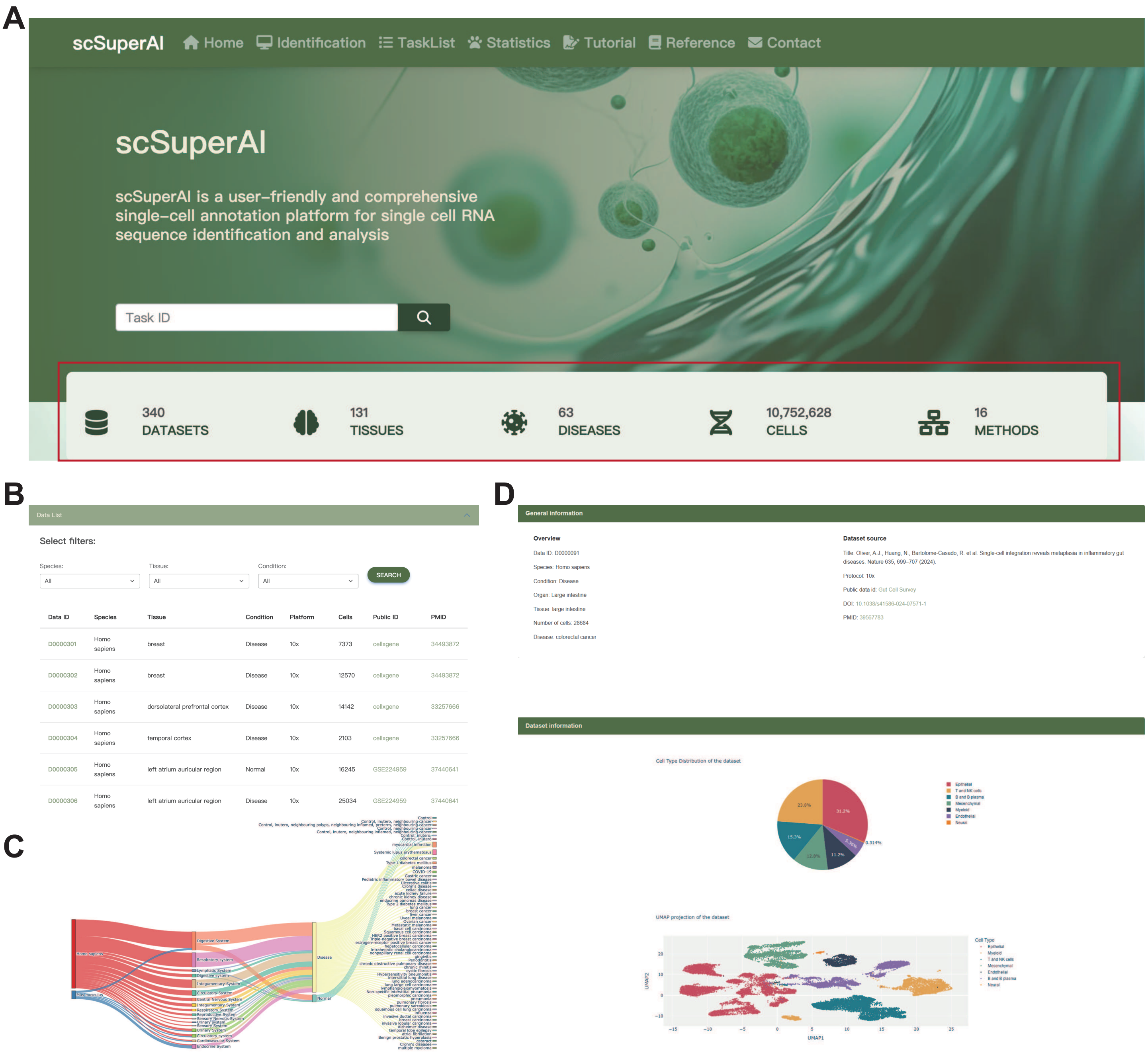

Supplementary Figure S80. Overview of the platform interface and dataset organization. (**A**) Home page showing the scSuperAnnotator's dataset statistics. (**B**) Statistics page listing all datasets with fields for Tissue, Condition, Platform, and data source. (**C**) Hierarchical organization of all datasets by species (Homo sapiens, Mus musculus), physiological system, and health status (Normal vs. Disease), with the terminal level specifying disease type. (**D**) Per-dataset summary combining general information with the cell-type composition and the proportions of each cell type; the detailed dataset page is accessed by clicking the Data ID in (**B**).

CaSTLe

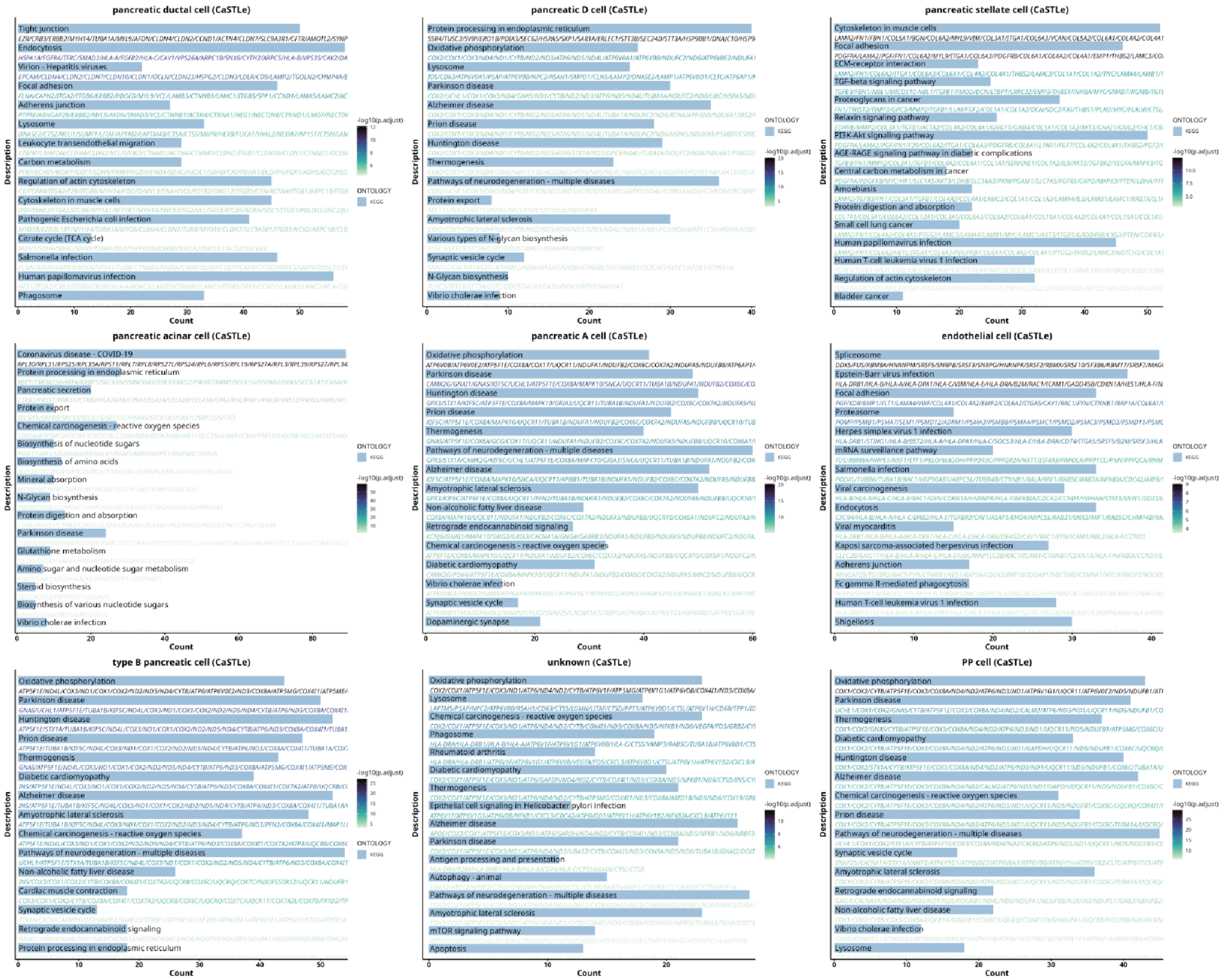

Supplementary Figure S81. KEGG pathway enrichment interface in the Task Details view of scSuperAnnotator.

References

[1] Yuqi Tan and Patrick Cahan. Singlecellnet: a computational tool to classify single cell rna-seq data across platforms and across species. *Cell systems*, 9(2):207–213, 2019.

[2] Jose Alquicira-Hernandez, Anuja Sathe, Hanlee P Ji, Quan Nguyen, and Joseph E Powell. scpred: accurate supervised method for cell-type classification from single-cell rna-seq data. *Genome biology*, 20:1–17, 2019.

[3] Feiyang Ma and Matteo Pellegrini. Actinn: automated identification of cell types in single cell rna sequencing. *Bioinformatics*, 36(2):533–538, 2020.

[4] Yuval Lieberman, Lior Rokach, and Tal Shay. Castle–classification of single cells by transfer learning: harnessing the power of publicly available single cell rna sequencing experiments to annotate new experiments. *PloS one*, 13(10):e0205499, 2018.

[5] Yingxin Lin, Yue Cao, Hani Jieun Kim, Agus Salim, Terence P Speed, David M Lin, Pengyi Yang, and Jean Yee Hwa Yang. scclassify: sample size estimation and multiscale classification of cells using single and multiple reference. *Molecular systems biology*, 16(6):e9389, 2020.

[6] Xin Shao, Haihong Yang, Xiang Zhuang, Jie Liao, Penghui Yang, Junyun Cheng, Xiaoyan Lu, Huajun Chen, and Xiaohui Fan. scdeepsort: a pre-trained cell-type annotation method for single-cell transcriptomics using deep learning with a weighted graph neural network. *Nucleic acids research*, 49(21):e122–e122, 2021.

[7] Yushan Qiu, Lingfei Yang, Hao Jiang, and Quan Zou. sctpc: a novel semisupervised deep clustering model for scrna-seq data. *Bioinformatics*, 40(5):btae293, 2024.

[8] Tianxiang Liu, Cangzhi Jia, Yue Bi, Xudong Guo, Quan Zou, and Fuyi Li. scdfn: enhancing single-cell rna-seq clustering with deep fusion networks. *Briefings in Bioinformatics*, 25(6):bbae486, 2024.

[9] Vladimir Yu Kiselev, Andrew Yiu, and Martin Hemberg. scmap: projection of single-cell rna-seq data across data sets. *Nature methods*, 15(5):359–362, 2018.

[10] Dvir Aran, Agnieszka P Looney, Leqian Liu, Esther Wu, Valerie Fong, Austin Hsu, Suzanna Chak, Ram P Naikawadi, Paul J Wolters, Adam R Abate, et al. Reference-based analysis of lung single-cell sequencing reveals a transitional profibrotic macrophage. *Nature immunology*, 20(2):163–172, 2019.

[11] Jurrian K De Kanter, Philip Lijnzaad, Tito Candelli, Thanasis Margaritis, and Frank CP Holstege. Chetah: a selective, hierarchical cell type identification method for single-cell rna sequencing. *Nucleic acids research*, 47(16):e95–e95, 2019.

[12] Xin Shao, Jie Liao, Xiaoyan Lu, Rui Xue, Ni Ai, and Xiaohui Fan. sccatch: automatic annotation on cell types of clusters from single-cell rna sequencing data. *Iscience*, 23(3), 2020.

[13] Ze Zhang, Danni Luo, Xue Zhong, Jin Huk Choi, Yuanqing Ma, Stacy Wang, Elena Mahrt, Wei Guo, Eric W Stawiski, Zora Modrusan, et al. Scina: a semi-supervised subtyping algorithm of single cells and bulk samples. *Genes*, 10(7):531, 2019.

[14] Allen W Zhang, Ciara O'Flanagan, Elizabeth A Chavez, Jamie LP Lim, Nicholas Ceglia, Andrew McPherson, Matt Wiens, Pascale Walters, Tim Chan, Brittany Hewitson, et al. Probabilistic cell-type assignment of single-cell rna-seq for tumor microenvironment profiling. *Nature methods*, 16(10):1007–1015, 2019.

[15] Hannah A Pliner, Jay Shendure, and Cole Trapnell. Supervised classification enables rapid annotation of cell atlases. *Nature methods*, 16(10):983–986, 2019.

[16] Amanda J Oliver, Ni Huang, Raquel Bartolome-Casado, Ruoyan Li, Simon Koplev, Hogne R Nilsen, Madelyn Moy, Batuhan Cakir, Krzysztof Polanski, Victoria Gudiño, et al. Single-cell integration reveals metaplasia in inflammatory gut diseases. *Nature*, 635(8039):699–707, 2024.

[17] Lisa Sikkema, Ciro Ramírez-Suástegui, Daniel C Strobl, Tessa E Gillett, Luke Zappia, Elo Madissoon, Nikolay S Markov, Laure-Emmanuelle Zaragosi, Yuge Ji, Meshal Ansari, et al. An integrated cell atlas of the lung in health and disease. *Nature medicine*, 29(6):1563–1577, 2023.

[18] Gabriela Rapozo Guimarães, Giovanna Resk Maklouf, Cristiane Esteves Teixeira, Leandro de Oliveira Santos, Nayara Gusmão Tessarollo, Nayara Evelin de Toledo, Alessandra Freitas Serain, Cristóvão Antunes de Lanna, Marco Antônio Pretti, Jéssica Gonçalves Vieira da Cruz, et al. Single-cell resolution characterization of myeloid-derived cell states with implication in cancer outcome. *Nature Communications*, 15(1):5694, 2024.

[19] Xiaoping Han, Ziming Zhou, Lijiang Fei, Huiyu Sun, Renying Wang, Yao Chen, Haide Chen, Jingjing Wang, Huanna Tang, Wenhao Ge, et al. Construction of a human cell landscape at single-cell level. *Nature*, 581(7808):303–309, 2020.

[20] The Tabula Sapiens Consortium*, Robert C Jones, Jim Karkanias, Mark A Krasnow, Angela Oliveira Pisco, Stephen R Quake, Julia Salzman, Nir Yosef, Bryan Bulthaup, Phillip Brown, et al. The tabula sapiens: A multiple-organ, single-cell transcriptomic atlas of humans. *Science*, 376(6594):eabl4896, 2022.

[21] Maayan Baron, Adrian Veres, Samuel L Wolock, Aubrey L Faust, Renaud Gaujoux, Amedeo Vetere, Jennifer Hyoje Ryu, Bridget K Wagner, Shai S Shen-Orr, Allon M Klein, et al. A single-cell transcriptomic map of the human and mouse pancreas reveals inter-and intra-cell population structure. *Cell systems*, 3(4):346–360, 2016.

[22] Mauro J Muraro, Gitanjali Dharmadhikari, Dominic Grün, Nathalie Groen, Tim Dielen, Erik Jansen, Leon Van Gurp, Marten A Engelse, Francoise Carlotti, Eelco Jp De Koning, et al. A single-cell transcriptome atlas of the human pancreas. *Cell systems*, 3(4):385–394, 2016.

[23] Åsa Segerstolpe, Athanasia Palasantza, Pernilla Eliasson, Eva-Marie Andersson, Anne-Christine Andréasson, Xiaoyan Sun, Simone Picelli, Alan Sabirsh, Maryam Clausen, Magnus K Bjursell, et al. Single-cell transcriptome profiling of human pancreatic islets in health and type 2 diabetes. *Cell metabolism*, 24(4):593–607, 2016.

[24] Yurong Xin, Jinrang Kim, Haruka Okamoto, Min Ni, Yi Wei, Christina Adler, Andrew J Murphy, George D Yancopoulos, Calvin Lin, and Jesper Gromada. Rna sequencing of single human islet cells reveals type 2 diabetes genes. *Cell metabolism*, 24(4):608–615, 2016.

[25] Maria Fasolino, Gregory W Schwartz, Abhijeet R Patil, Aanchal Mongia, Maria L Golson, Yue J Wang, Ashleigh Morgan, Chengyang Liu, Jonathan Schug, Jinping Liu, et al. Single-cell multi-omics analysis of human pancreatic islets reveals novel cellular states in type 1 diabetes. *Nature metabolism*, 4(2):284–299, 2022.

[26] A single-cell transcriptomic atlas characterizes ageing tissues in the mouse. *Nature*, 583(7817):590–595, 2020.

[27] Mahnoor N Gondal, Marcin Cieslik, and Arul M Chinnaiyan. Integrated cancer cell-specific single-cell rna-seq datasets of immune checkpoint blockade-treated patients. *Scientific Data*, 12(1):139, 2025.

[28] Blue B Lake, Rajasree Menon, Seth Winfree, Qiwen Hu, Ricardo Melo Ferreira, Kian Kalhor, Daria Barwinska, Edgar A Otto, Michael Ferkowicz, Dinh Diep, et al. An atlas of healthy and injured cell states and niches in the human kidney. *Nature*, 619(7970):585–594, 2023.

[29] Sunny Z Wu, Ghamdan Al-Eryani, Daniel Lee Roden, Simon Junankar, Kate Harvey, Alma Andersson, Aatish Thennavan, Chenfei Wang, James R Torpy, Nenad Bartonicek, et al. A single-cell and spatially resolved atlas of human breast cancers. *Nature genetics*, 53(9):1334–1347, 2021.

[30] Quinn T Easter, Bruno Fernandes Matuck, Germán Beldorati Stark, Catherine L Worth, Alexander V Predeus, Brayon Fremin, Khoa Huynh, Vaishnavi Ranganathan, Zhi Ren, Diana Pereira, et al. Single-cell and spatially resolved interactomics of tooth-associated keratinocytes in periodontitis. *Nature Communications*, 15(1):5016, 2024.

[31] Hyun Min Kang, Meena Subramaniam, Sasha Targ, Michelle Nguyen, Lenka Maliskova, Elizabeth McCarthy, Eunice Wan, Simon Wong, Lauren Byrnes, Cristina M Lanata, et al. Multiplexed droplet single-cell rna-sequencing using natural genetic variation. *Nature biotechnology*, 36(1):89–94, 2018.
